# Supplementary material for: Subtype-selective agonists of plant hormone co-receptor COI1-JAZs identified from the stereoisomers of coronatine
Source: Commun Biol. 2023 Mar 25;6:320. doi: 10.1038/s42003-023-04709-1 (PMC10039919; doi:10.1038/s42003-023-04709-1)
Supplement: Supplementary file 2 — Supplementary Information [file 42003_2023_4709_MOESM2_ESM.pdf]

Supplemental Materials for

Title:

Subtype-selective agonists of plant hormone co-receptor COI1-JAZs identified from the stereoisomers of coronatine

Authors:

Kengo Hayashi,[1] Nobuki Kato,[1] Khurram Bashir,[2,3] Haruna Nomoto,[1] Misuzu Nakayama,[1] Andrea Chini,[4] Satoshi Takahashi,[2] Hiroaki Saito,[5] Raku Watanabe,[6] Yousuke Takaoka,[1] Maho Tanaka,[2] Atsushi J. Nagano,[7,8] Motoaki Seki,[2] Roberto Solano,[4] Minoru Ueda[1,6]*

Affiliation:

[1]Department of Chemistry, Graduate School of Science, Tohoku University, Sendai 980-8578, Japan

[2]Plant Genomic Network Research Team, RIKEN Center for Sustainable Resource Science, Yokohama 230-0045, Japan

[3]Department of Life Sciences, SBA School of Science and Engineering, Lahore University of Management Sciences, 54792, Lahore, Pakistan

[4]Plant Molecular Genetics Department, National Centre for Biotechnology (CNB), Consejo Superior de Investigaciones Cientificas (CSIC), Campus University Autonoma, 28049 Madrid, Spain

[5]Faculty of Pharmaceutical Sciences, Hokuriku University, Kanazawa, 920-1181, Japan

[6]Department of Molecular and Chemical Life Sciences, Graduate School of Life Sciences, Tohoku University, Sendai 980-8578, Japan.

[7]Faculty of Agriculture, Ryukoku University, Shiga 520-2194, Japan

[8]Institute for Advanced Biosciences, Keio University, Yamagata, 997-0017, Japan

*Corresponding author e-mail: minoru.ueda.d2@tohoku.ac.jp

Supplementary Methods

Chemical Synthesis

1. General

All chemical reagents and solvents were obtained from commercial suppliers (Kanto Chemical Co. Ltd., Wako Pure Chemical Industries Co. Ltd., Nacalai Tesque Co. Ltd., Tokyo Chemical Industry Co. Ltd., Sigma-Aldrich Co. LLC., GE Healthcare) and used without further purification. All anhydrous solvents were either dried by standard techniques and freshly distilled before use or purchased in anhydrous form and used as supplied. Reversed-phase high–performance liquid chromatography (HPLC) was carried out on a PU–4180 plus pump equipped with UV–4075 and MD–4010 detectors (JASCO, Tokyo, Japan). ^{1}H and ^{13}C NMR spectra were recorded on a JNM–ECS–400 spectrometers (JEOL, Tokyo, Japan) in deuterated chloroform using TMS as an internal standard. Fourier transforms infrared (FT/IR) spectra were recorded on an FT/IR–4100 (JASCO, Tokyo, Japan). High–resolution (HR) electrospray ionization (ESI)–mass spectrometry (MS) analyses were conducted using a microTOF II (Bruker Daltonics Inc., Billerica, MA). Optical rotations were measured using a JASCO P–2200 polarimeter (JASCO, Tokyo, Japan). Flash chromatography was performed on an Isolera system (Biotage Ltd., North Carolina, US). TLC analyses were performed on Silica gel F254 (0.25 mm or 0.5 mm, MERCK, Germany) or RP–18F254S (0.25 mm, MERCK). $SiO_2/K_2CO_3/H_2O$ is a homogeneous mixture of 400 g of SiO_2, 40 g of K_2CO_3, and 120 mL of water. All reactions were carried out under air unless stated otherwise.

2. Synthesis of (+)-C6-*epi*-CFA (3b) and (-)-C6-*epi*-CFA (3c)

2. 1. Synthesis of the diastereomeric mixture of CFAs (+)-9.

To a suspension of **3**[1] (520 mg, 2.50 mmol) and K_2CO_3 (390 mg, 2.83 mmol) in DMF (4.6 mL) was added iodomethane (320 μL, 5.14 mmol) under argon atmosphere. After stirring the reaction mixture for 3 h, the reaction mixture was diluted with hexane and quenched with H_2O. The mixture was extracted with hexane, and the combined organic layers were washed with saturated NaCl aq., dried over Na_2SO_4, and filtered. The reaction mixture was concentrated under reduced pressure to afford the methyl ester (539 mg mixture). The crude product was used for the next reaction without further purification. To a solution of the mixture (539 mg, mixture) in benzene (4.7 mL) was added DBU (330 μL, 2.21 mmol) under an argon atmosphere. After the reaction mixture was stirred at

reflux temperature for 23 h, the reaction mixture was quenched with 1 M HCl aq. The mixture was extracted with EtOAc, and the combined organic layers were washed with saturated NaCl aq., dried over Na_2SO_4, and filtered. The reaction mixture was concentrated under reduced pressure to afford the diastereomeric mixture of methyl esters (539 mg, mixture). The crude product was used for the next reaction without further purification. A mixture suspension (539 mg, mixture) in 3 M HCl aq. (40 mL) was refluxed for 6 h. After the reaction mixture was quenched with H_2O, the mixture was extracted with EtOAc. The combined organic layers were washed with saturated NaCl aq., dried over Na_2SO_4, and filtered. After evaporation, the crude product was purified by medium-pressure chromatography (Isolera, eluent: 0.1:90:10 AcOH/*n*-hexane/EtOAc to 0.1:20:80 AcOH/*n*-hexane/EtOAc) to afford the diastereomeric mixture of CFAs (+)-**9**. (443 mg, 6*R*:6*S*=3:2, 85% in 3 steps) was obtained as a pale yellow solid.

2. 2. Synthesis of mixed anhydride (+)-10.

PivCl (260 μL, 2.13 mmol) was added to a solution of the diastereomeric mixture (+)-**9** (267 mg, 1.28 mmol) and Et_3N (940 μL, 6.74 mmol) in CH_2Cl_2 (5 mL) and the mixture was stirred for 12 h. The reaction was quenched with 1 M HCl aq. and the mixture was extracted with CH_2Cl_2. The combined organic layers were washed with brine, dried over Na_2SO_4, and concentrated under reduced pressure. The crude product was purified by medium-pressure chromatography (Isolera, eluent: 0.1:95:5 AcOH/*n*-hexane/EtOAc) to afford (+)-**10** (86.6 mg, 25%) as a colorless oil and 6*R*-isomer (119 mg, 34%) as a colorless oil.

(+)-**10**: $[\alpha]_D^{21}$ +127.4 (*c* 0.50, $CHCl_3$); ^1H NMR (400 MHz, $CDCl_3$) δ_H: 7.04 (dd, *J* = 2.4, 1.0 Hz, 1H), 3.33-3.26 (m, 1H), 2.51 (ddd, *J* = 12.0, 7.2, 1.0 Hz, 1H), 2.33 (td, *J* = 12.0, 8.0 Hz, 1H), 2.30 (d, *J* = 8.0 Hz, 1H), 2.18 (dqd, *J* = 15.0, 7.6, 1.6 Hz, 1H), 2.14-2.05 (m, 2H), 2.01 (td, *J* = 6.0, 4.4 Hz, 1H), 1.46-1.37 (m, 2H), 1.37 (dd, *J* = 15.0, 7.2 Hz, 1H), 1.30 (s, 9H), 0.98 (t, *J* = 7.6 Hz, 3H); ^{13}C NMR (100 MHz, $CDCl_3$) δ_C: 220.5, 174.2, 162.8, 149.5, 131.2, 45.8, 40.0, 36.6, 35.8, 35.0, 27.4, 26.6, 26.6, 25.2, 11.4; IR (film) cm^{-1}: 2967, 2936, 2877, 1796, 1738, 1634, 1480, 1461, 1400, 1368, 1278, 1213, 1144, 1089, 1065, 1041, 1018, 991, 943, 924, 887; HRMS (ESI, positive) *m/z* $[M+Na]^+$ Calcd. for $C_{17}H_{24}NaO_4$: 315.1567, Found: 315.1552

6*R*-isomer: $[\alpha]_D^{21}$ +100.8 (*c* 0.14, $CHCl_3$); ^1H NMR (400 MHz, $CDCl_3$) δ_H: 6.99 (s, 1H), 3.12 (dt, *J* = 11.4, 7.6 Hz, 1H), 2.64 (dt, *J* = 11.4, 7.6 Hz, 1H), 2.43 (dd, *J* = 19.2,

8.0 Hz, 1H), 2.36 (ddd, J = 16.0, 8.0, 4.4 Hz, 1H), 2.30-2.21 (m, 1H), 1.91 (dt, J = 13.6, 4.4 Hz, 1H), 1.69-1.41 (m, 3H), 1.31 (s, 9H), 1.31 (td, J = 13.6, 10.8 Hz, 1H), 0.99 (t, J = 7.6 Hz, 3H); ^{13}C NMR (100 MHz, $CDCl_3$) δ_C: 219.6, 174.2, 162.8, 148.3, 131.6, 46.4, 40.0, 38.1, 38.0, 36.1, 27.8, 27.7, 26.6, 25.6, 11.2; IR (film) cm^{-1}: 2967, 2937, 2875, 1796, 1742, 1636, 1479, 1463, 1226, 1215, 1144, 1092, 1069, 1043, 1010, 976, 750; HRMS (ESI, positive) *m/z* $[M+Na]^+$ Calcd. for $C_{17}H_{24}NaO_4$: 315.1567, Found: 315.1564

2. 3. Synthesis of 3b.

To a solution of (+)-**10** (86.6 mg, 296 μmol) in THF (3 mL) was added 1M LiOH aq. (900 μl, 900 μmol). After the reaction mixture was stirred for 11 h, the reaction mixture was diluted with EtOAc and acidified 1 M HCl aq. The mixture was extracted with EtOAc. The combined organic layers were washed with brine, dried over Na_2SO_4, and concentrated under reduced pressure. The crude product was purified by medium-pressure chromatography (Isolera, eluent: 0.1:90:10 AcOH/*n*-hexane/EtOAc to 0.1:20:80 AcOH/*n*-hexane/EtOAc) to afford **3b** (55.1 mg, 89%) as a colorless crystalline solid. All spectral data of **3b** were identical to those reported.[2]

2. 4. Synthesis of the diastereomeric mixture of CFAs (-)-9.

To a suspension of **3a**[1] (286 mg, 1.38 mmol) and K_2CO_3 (220 mg, 1.59 mmol) in DMF (3 mL) was added iodomethane (180 μL, 2.89 mmol) under argon atmosphere. After the reaction mixture was stirred for 2 h, the reaction mixture was diluted with hexane and quenched with H_2O. The mixture was extracted with hexane, and the combined organic layers were washed with saturated NaCl aq., dried over Na_2SO_4, and filtered. The reaction mixture was concentrated under reduced pressure to afford the methyl ester (299 mg, mixture). The crude product was used for the next reaction without further purification. To a solution of the mixture (299 mg, mixture) in benzene (2.7 mL) was added DBU (190 μL, 1.27 mmol) under an argon atmosphere. After the reaction mixture was stirred at reflux temperature for 20 h, the reaction mixture was quenched with 1 M HCl aq. The mixture was extracted with EtOAc, and the combined organic layers were washed with saturated NaCl aq., dried over Na_2SO_4, and filtered. The reaction mixture was concentrated under reduced pressure to afford the diastereomeric mixture of methyl esters (279 mg, mixture). The crude product was used for the next reaction without further purification. A mixture suspension (279 mg, mixture) in 3 M HCl aq. (20 mL) was

refluxed for 9 h. After the reaction mixture was quenched with H_2O, the mixture was extracted with EtOAc. The combined organic layers were washed with saturated NaCl aq., dried over Na_2SO_4, and filtered. After evaporation, the crude product was purified by medium-pressure chromatography (Isolera, eluent: 0.1:90:10 AcOH/*n*-hexane/EtOAc to 0.1:20:80 AcOH/*n*-hexane/EtOAc) to afford the diastereomeric mixture of CFAs (-)-**9**. (252 mg, 6*R*:6*S*=3:2, 88% in 3 steps) was obtained as a white solid.

2. 5. Synthesis of mixed anhydride (-)-10.

PivCl (30 μL, 246 μmol) was added to a solution of the diastereomeric mixture (-)-**9** (31.5 mg, 151 μmol) and Et_3N (110 μL, 798 μmol) in CH_2Cl_2 (1.2 mL) and the mixture was stirred for 2 h. The reaction was quenched with 1 M HCl aq. and the mixture was extracted with CH_2Cl_2. The combined organic layers were washed with brine, dried over Na_2SO_4, and concentrated under reduced pressure. The crude product was purified by medium-pressure chromatography (Isolera, eluent: 0.1:95:5 AcOH/*n*-hexane/EtOAc) to afford (-)-**10** (14.6 mg, 33%) as a colorless oil and 6*S*-isomer (19.6 mg, 44%) as a colorless oil.

(-)-**10**: $[\alpha]_D^{25}$ −123.6 (*c* 0.54, $CHCl_3$); δ_H: 7.05 (dd, *J* = 2.4, 0.8 Hz, 1H), 3.33-3.26 (m, 1H), 2.51 (ddd, *J* = 12.0, 6.8, 0.8 Hz, 1H), 2.33 (td, *J* = 12.0, 7.6 Hz, 1H), 2.30 (d, *J* = 7.6 Hz, 1H), 2.18 (dqd, *J* = 14.8, 7.6, 1.2 Hz, 1H), 2.14-2.05 (m, 2H), 2.01 (td, *J* = 6.4, 4.0 Hz, 1H) 1.47-1.36 (m, 2H), 1.37 (dd, *J* = 14.8, 6.8 Hz, 1H), 1.31 (s, 9H), 0.98 (t, *J* = 7.2 Hz, 3H); ^{13}C NMR (100 MHz, $CDCl_3$) δ_C: 220.5, 174.3, 162.8, 149.5, 131.2, 45.7, 40.0, 36.6, 35.8, 35.0, 27.4, 26.6, 26.6, 25.2, 11.4; IR (film) cm^{-1}: 2967, 2936, 2877, 1797, 1738, 1635, 1479, 1462, 1399, 1368, 1278, 1213, 1144, 1089, 1064, 1042, 1015, 991, 942, 924, 886; HRMS (ESI, positive) *m/z* $[M+Na]^+$ Calcd. for $C_{17}H_{24}NaO_4$: 315.1567, Found: 315.1584

6*S*-isomer: $[\alpha]_D^{21}$ −97.3 (*c* 0.46, $CHCl_3$); ^{1}H NMR (400 MHz, $CDCl_3$) δ_H: 6.99 (s, 1H), 3.12 (dt, *J* = 11.6, 6.8 Hz, 1H), 2.64 (dt, *J* = 11.6, 6.8 Hz, 1H), 2.42 (dd, *J* = 18.8, 8.4 Hz, 1H), 2.35 (ddd, *J* = 16.8, 8.4, 4.0 Hz, 1H), 2.30-2.22 (m, 1H), 1.91 (dt, *J* = 13.6, 4.0 Hz, 1H), 1.63 (qd, *J* = 12.6, 8.4 Hz, 1H), 1.59-1.39 (m, 2H), 1.31 (s, 9H), 1.12 (td, *J* = 13.6, 10.8 Hz, 1H), 0.99 (t, *J* = 7.6 Hz, 3H); ^{13}C NMR (100 MHz, $CDCl_3$) δ_C: 219.5, 174.1, 162.7, 148.3, 131.6, 46.3, 40.0, 38.1, 38.0, 36.1, 27.8, 27.6, 26.6, 25.6, 11.2; IR (film) cm^{-1}: 2967, 2938, 2876, 1796, 1744, 1635, 1479, 1463, 1228, 1216, 1141, 1092,

1067, 1044, 1010, 977, 750; HRMS (ESI, positive) *m/z* $[M+Na]^+$ Calcd. for $C_{17}H_{24}NaO_4$: 315.1567, Found: 315.1581

2. 6. Synthesis of 3c.

To a solution of (-)-**10** (7.3 mg, 35.1 µmol) in THF (500 mL) was added 1M LiOH aq. (150 µl, 150 µmol). After the reaction mixture was stirred for 12 h, the reaction mixture was diluted with EtOAc and acidified 1 M HCl aq. The mixture was extracted with EtOAc. The combined organic layers were washed with brine, dried over Na_2SO_4, and concentrated under reduced pressure. The crude product was purified by medium-pressure chromatography (Isolera, eluent: 0.1:90:10 AcOH/*n*-hexane/EtOAc to 0.1:20:80 AcOH/*n*-hexane/EtOAc) to afford **3c** (5.3 mg, quant.) as a white solid. All spectral data of **3c** were identical to those reported.[2]

3. Synthesis of coronatine (2) and the stereoisomers (2a–2o).

3. 1. General procedure A: Condensation between CMA and CFA stereoisomers.

For example, the synthesis of coronatine benzyl ester (**11**).

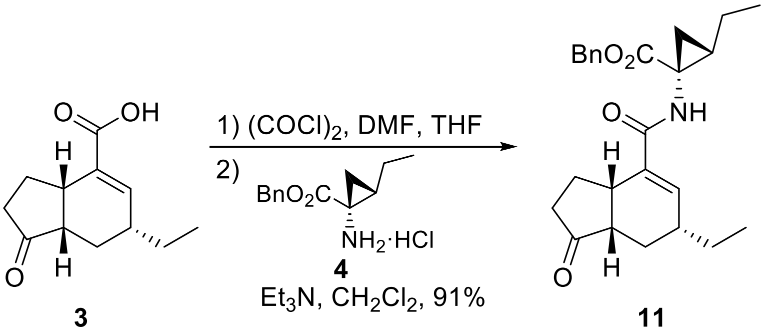

To a solution of **3** (34.1 mg, 164 µmol) and DMF (2 µL, 25.9 µmol) in THF (800 µL) was added oxalyl chloride (45 µL, 524 µmol) under an argon atmosphere. After stirring the reaction mixture at room temperature for 11 h, THF was removed *in vacuo*. The residue was solved in CH_2Cl_2 (2 mL), and then a solution of **4** (69.1 mg, 270 µmol) and Et_3N (350 µL, 2.50 mmol) in CH_2Cl_2 (1 mL) was added under an argon atmosphere. After the reaction mixture was stirred for 21 h, the reaction mixture was quenched with 1 M HCl aq., and the mixture was extracted with AcOEt. The combined organic layers were washed with brine, dried over Na_2SO_4, and concentrated under reduced pressure. The crude product was purified by medium-pressure chromatography (Isolera, eluent: $CHCl_3$ to 4:96 MeOH/$CHCl_3$) to afford **11** (61.1 mg, 91%) as a yellow oil.

3. 2. General procedure B: Condensation between CMA and CFA stereoisomers.

For example, the synthesis of **2g** benzyl ester (**11g**).

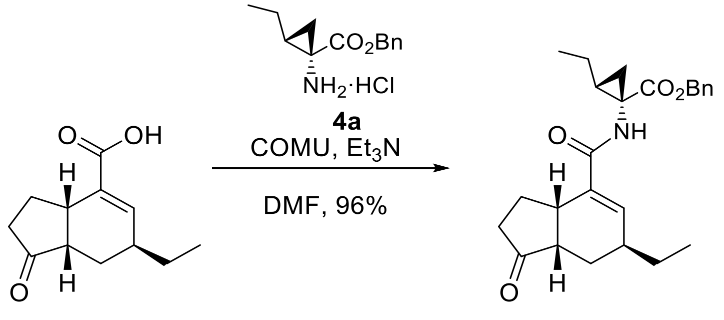

3b **11g**

To a solution of **3b** (16.4 mg, 51.3 μmol) in CH_2Cl_2 (200 μL) was added TFA (200 μL) at 0 °C under argon atmosphere. After being stirred for 30 min, the reaction mixture was evaporated, and then the residue was dissolved in DMF (300 μL) under an argon atmosphere. To the solution were added Et_3N (20 μL, 14.4 μmol), COMU (15.7 mg, 36.7 μmol), and **4a** (2.6 mg, 12.5 μmol). After stirring for 20 h, the reaction mixture was quenched with 1 M HCl aq. The mixture was extracted with EtOAc, and the resulting organic layer was washed with saturated aqueous NaCl, dried over Na_2SO_4, and filtered. After evaporation, the residue was purified by silica gel column chromatography (*n-hexane*/EtOAc = 16/1–1/1) to give the benzyl ester (4.9 mg, 12.0 μmol, 96%) a pale yellow solid.

3. 3. General procedure C: Deprotection of benzyl ester.

For example, the synthesis of coronatine (**2**).

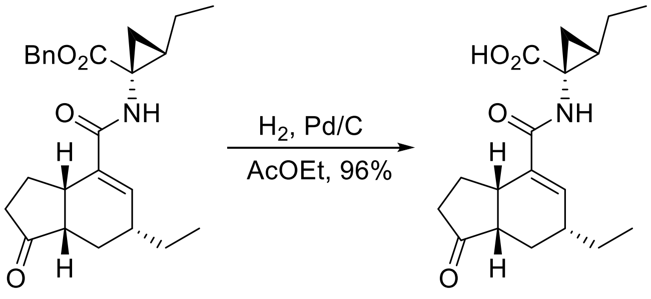

11 **2**

Benzyl ester **11** (61.1 mg, 149 μmol) was dissolved in EtOAc (5.0 mL) under argon and 5% Pd/C (35.0 mg, 16.4 μmol) was added. The atmosphere was displaced with hydrogen, and the reaction mixture was stirred for 80 min. After filtration with Celite, the

filtrate was evaporated to dryness. The residue was purified by medium-pressure chromatography (Isolera, eluent: 0.1:1:99 AcOH/MeOH/$CHCl_3$ to 0.1:8:92 AcOH/MeOH/$CHCl_3$) to afford **2** (46.1 mg, 96%) as a colorless oil.

3. 4. General procedure D: Deprotection of methyl ester.

For example, the synthesis of **2e**.

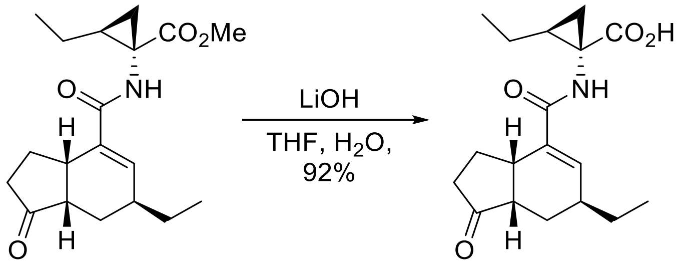

To a solution of **11e** (77.9 mg, 234 μmol) in THF (4.5 ml) was added 1M-LiOH solution (1.8 mL, 1.80 mmol), and the mixture was stirred for 20 h. The reaction mixture was quenched with 1 M HCl aq., and the mixture was extracted with AcOEt. The combined organic layers were washed with brine, dried over Na_2SO_4, and concentrated under reduced pressure. The crude product was purified by medium-pressure chromatography (Isolera, eluent: 0.1:1:99 AcOH/MeOH/$CHCl_3$ to 0.1:8:92 AcOH/MeOH/$CHCl_3$) to afford **2e** (69.4 mg, 92%) as a colorless oil.

3. 5. Synthesis of coronatine (2).

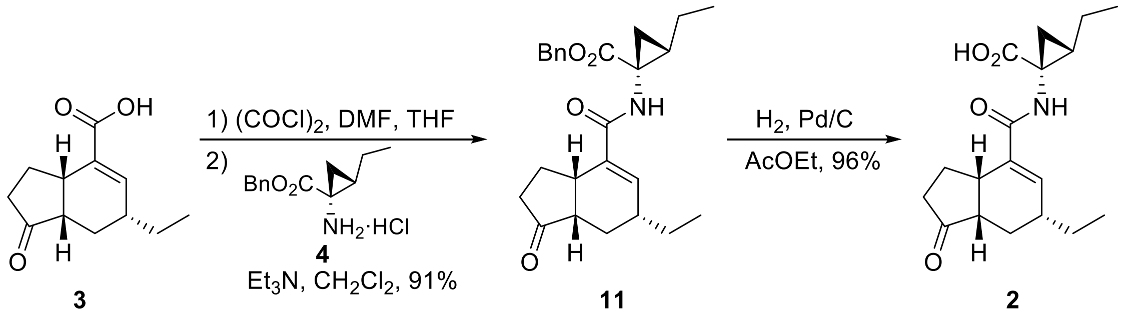

Compound 11

Prepared according to General Procedure A. $[\alpha]_D^{22}$ +51.5 (*c* 0.28, $CHCl_3$); ^1H NMR (400 MHz, $CDCl_3$) δ_H: 7.34–7.30 (m, 5H), 6.23 (dd, *J* = 3.4, 1.9 Hz, 1H), 5.17 (d, *J* = 12.2 Hz, 1H), 5.10 (d, *J* = 12.2 Hz, 1H), 3.36–3.31 (m, 1H), 2.46 (dd, *J* = 12.4, 5.0 Hz, 1H), 2.10–1.89 (m, 5H), 1.82–1.75 (m, 1H), 1.69–1.55 (m, 2H), 1.49 (dq, *J* = 9.1, 7.5 Hz

2H), 1.39–1.25 (m, 4H), 0.95 (t, J = 7.1 Hz, 3H), 0.92 (t, J = 7.5 Hz, 3H); ^{13}C NMR (100 MHz, $CDCl_3$) δ_C: 221.0, 171.0, 170.0, 137.6, 136.1, 135.7, 128.7, 128.5, 67.5, 46.4, 38.3, 36.5, 36.3, 33.9, 33.4, 28.1, 26.2, 25.8, 23.4, 20.5, 13.7, 11.5; IR (film) cm^{-1}: 3303, 2962, 2929, 2875, 1732, 1656, 1625, 1520, 1457, 1329, 1161, 751, 698; HRMS (ESI, positive) *m/z* $[M+Na]^+$ Calcd. for $C_{25}H_{31}NNaO_4$: 432.2151, Found: 432.2134.

Coronatine (2)

Prepared according to General Procedure C. $[\alpha]_D^{22}$ +97.6 (*c* 0.15, $CHCl_3$); All spectral data of **2** were identical to those reported.[3]

3. 6. Synthesis of 2a.

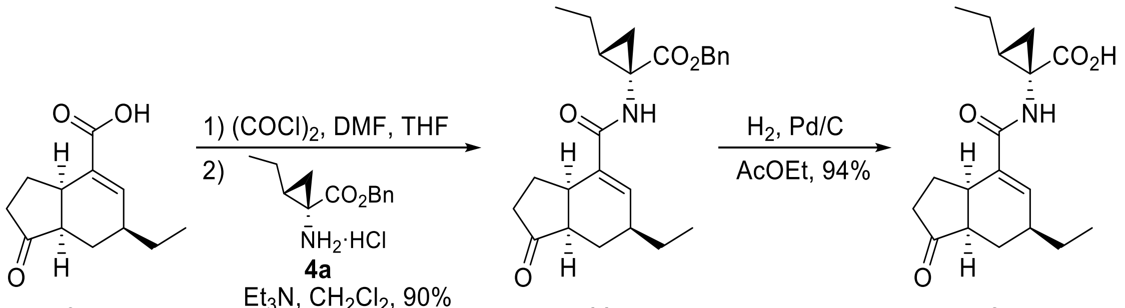

Compound 11a

Prepared according to General Procedure A. $[\alpha]_D^{22}$ −48.4 (*c* 0.34, $CHCl_3$); ^{1}H NMR (400 MHz, $CDCl_3$) δ_H: 7.34–7.30 (m, 5H), 6.23 (dd, J = 3.4, 1.9 Hz, 1H), 5.17 (d, J = 12.2 Hz, 1H), 5.10 (d, J = 12.2 Hz, 1H), 3.36–3.31 (m, 1H), 2.46 (dd, J = 12.4, 5.0 Hz, 1H), 2.10–1.89 (m, 5H), 1.82–1.75 (m, 1H), 1.69–1.55 (m, 2H), 1.49 (dq, J = 9.1, 7.5 Hz 2H), 1.39–1.25 (m, 4H), 0.95 (t, J = 7.1 Hz, 3H), 0.92 (t, J = 7.5 Hz, 3H); ^{13}C NMR (100 MHz, $CDCl_3$) δ_C: 221.0, 171.0, 170.0, 137.6, 136.1, 135.7, 128.7, 128.5, 67.5, 46.4, 38.3, 36.5, 36.3, 33.9, 33.4, 28.1, 26.2, 25.8, 23.4, 20.5, 13.7, 11.5; IR (film) cm^{-1}: 3303, 2962, 2929, 2875, 1732, 1656, 1625, 1520, 1457, 1329, 1161, 751, 698; HRMS (ESI, positive) *m/z* $[M+Na]^+$ Calcd. for $C_{25}H_{31}NNaO_4$: 432.2151, Found: 432.2177.

Compound 2a

Prepared according to General Procedure C. $[\alpha]_D^{22}$ −96.8 (*c* 0.20, $CHCl_3$); All spectral data of **2a** were identical to those reported.[3]

3. 7. Synthesis of 2b.

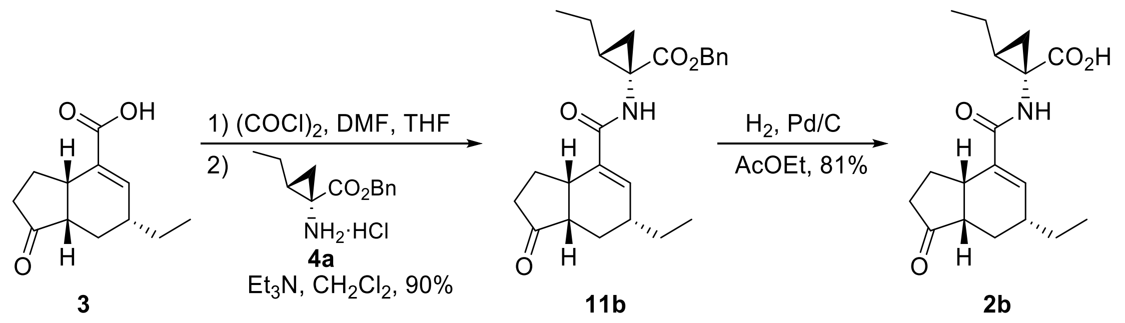

Compound 11b

Prepared according to General Procedure A. $[\alpha]_D^{22}$ −1.4 (*c* 0.335, $CHCl_3$): ^1H NMR (400 MHz, $CDCl_3$) δ_H: 7.34–7.30 (m, 5H), 6.27 (s, 1H), 6.22 (s, 1H), 5.16 (d, *J* = 12.4 Hz, 1H), 5.12 (d, *J* = 12.4 Hz, 1H), 3.15–3.07 (m, 1H), 2.39–2.16 (m, 1H), 2.14–2.06 (m, 1H), 1.86 (dt, *J* = 12.8, 4.8 Hz, 1H), 1.64 (ddd, *J* = 16.8, 7.2, 2.4 Hz, 1H), 1.64–1.58 (m, 2H), 1.48 (dq, *J* = 9.2, 7.2 Hz, 2H), 1.44–1.40 (m, 1H), 1.35 (ddq, *J* = 12.0, 7.6, 7.2 Hz, 1H), 1.35 (m, 1H), 1.02 (td, *J* = 13.6, 11.2 Hz, 1H), 0.97 (t, *J* = 7.2 Hz, 3H), 0.95 (t, *J* = 7.2 Hz, 3H); ^{13}C NMR (100 MHz, $CDCl_3$) δ_C: 220.4, 171.0, 169.2, 136.3, 136.0, 135.7, 128.5, 128.3, 128.2, 67.2, 46.3, 38.2, 37.2, 36.2, 33.4, 28.0, 27.6, 26.0, 23.3, 20.5, 13.5, 11.3; IR (film) cm^{-1}: 3328, 2958, 2931, 2870, 1732, 1658, 1628, 1512, 1454, 1380, 1319, 1107, 752, 698; HRMS (ESI, positive) *m/z* $[M+Na]^+$ Calcd. for $C_{25}H_{31}NNaO_4$: 432.2151, Found: 432.2137.

Compound 2b

Prepared according to General Procedure C. $[\alpha]_D^{22}$ -35.8 (*c* 0.53, $CHCl_3$); All spectral data of **2b** were identical to those reported.[3]

3. 8. Synthesis of 2c.

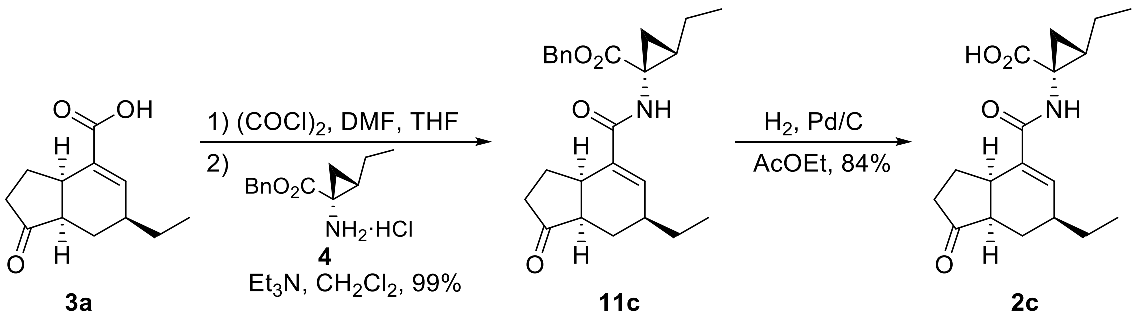

Compound 11c

Prepared according to General Procedure A. $[\alpha]_D^{22}$ +2.7 (*c* 0.22, $CHCl_3$); ^{1}H NMR (400 MHz, $CDCl_3$) δ_H: 7.34–7.30 (m, 5H), 6.27 (s, 1H), 6.22 (s, 1H), 5.16 (d, *J* = 12.4 Hz, 1H), 5.12 (d, *J* = 12.4 Hz, 1H), 3.15–3.07 (m, 1H), 2.39–2.16 (m, 1H), 2.14–2.06 (m, 1H), 1.86 (dt, *J* = 12.8, 4.8 Hz, 1H), 1.64 (ddd, *J* = 16.8, 7.2, 2.4 Hz, 1H), 1.64–1.58 (m, 2H), 1.48 (dq, *J* = 9.2, 7.2 Hz, 2H), 1.44–1.40 (m, 1H), 1.35 (ddq, *J* = 12.0, 7.6, 7.2 Hz, 1H), 1.35 (m, 1H), 1.02 (td, *J* = 13.6, 11.2 Hz, 1H), 0.97 (t, *J* = 7.2 Hz, 3H), 0.95 (t, *J* = 7.2 Hz, 3H); ^{13}C NMR (100 MHz, $CDCl_3$) δ_C: 220.4, 171.0, 169.2, 136.3, 136.0, 135.7, 128.5, 128.3, 128.2, 67.2, 46.3, 38.2, 37.2, 36.2, 33.4, 28.0, 27.6, 26.0, 23.3, 20.5, 13.5, 11.3; IR (film) cm^{-1}: 3328, 2958, 2931, 2870, 1732, 1658, 1628, 1512, 1454, 1380, 1319, 1107, 752, 698; HRMS (ESI, positive) *m/z* $[M+Na]^+$ Calcd. for $C_{25}H_{31}NNaO_4$: 432.2151, Found: 432.2136.

Compound 2c

Prepared according to General Procedure C. $[\alpha]_D^{22}$ +37.0 (*c* 0.60, $CHCl_3$); All spectral data of **2c** were identical to those reported.[3]

3. 9. Synthesis of 2d.

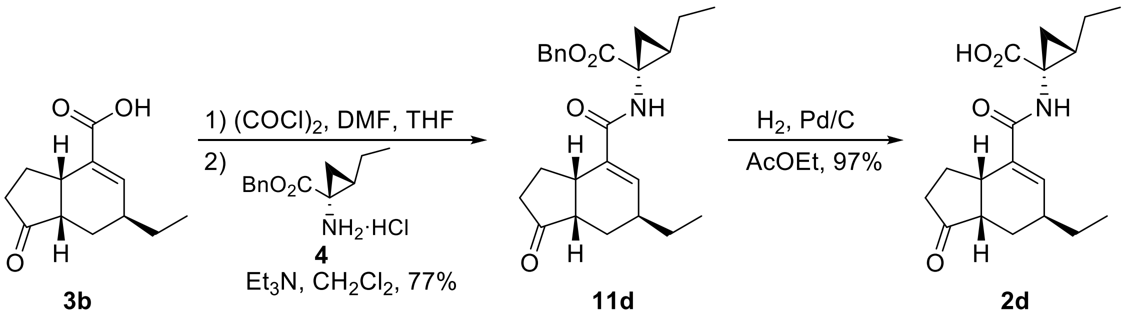

Compound 11d

Prepared according to General Procedure A. $[\alpha]_D^{22}$ +107.0 (*c* 0.31, $CHCl_3$); ^{1}H NMR (400 MHz, $CDCl_3$) δ_H: 7.34–7.30 (m, 5H), 6.23 (dd, *J* = 3.4, 1.9 Hz, 1H), 5.17 (d, *J* = 12.2 Hz, 1H), 5.10 (d, *J* = 12.2 Hz, 1H), 3.36–3.31 (m, 1H), 2.46 (dd, *J* = 12.4, 5.0 Hz, 1H), 2.10–1.89 (m, 5H), 1.82–1.75 (m, 1H), 1.69–1.55 (m, 2H), 1.49 (dq, *J* = 9.1, 7.5 Hz 2H), 1.39–1.25 (m, 4H), 0.95 (t, *J* = 7.1 Hz, 3H), 0.92 (t, *J* = 7.5 Hz, 3H); ^{13}C NMR (100 MHz, $CDCl_3$) δ_C: 221.0, 171.0, 170.0, 137.6, 136.1, 135.7, 128.7, 128.5, 67.5, 46.4, 38.3,

36.5, 36.3, 33.9, 33.4, 28.1, 26.2, 25.8, 23.4, 20.5, 13.7, 11.5; IR (film) cm^{-1}: 3303, 2962, 2929, 2875, 1732, 1656, 1625, 1520, 1457, 1329, 1161, 751, 698; HRMS (ESI, positive) *m/z* $[M+Na]^+$ Calcd. for $C_{25}H_{25}NNaO_4$: 432.2151, Found: 432.2144.

Compound 2d

Prepared according to General Procedure C. $[\alpha]_D^{22}$ +138.3 (*c* 0.23, $CHCl_3$); ^{1}H NMR (400 MHz, $CDCl_3$) δ_H: 6.38 (dd, *J* = 2.5, 1.0 Hz, 1H), 6.32 (brs, 1H), 3.38–3.32 (m, 1H), 2.52 (dd, *J* = 11.9, 6.2 Hz, 1H), 2.30–1.92 (m, 5H), 1.69–1.52 (m, 4H), 1.46 (dquint., *J* = 16.4, 7.7 Hz, 1H), 1.40–1.31 (m, 2H), 1.27 (dd, *J* = 9.4, 5.4 Hz, 1H), 1.26 (dd, *J* = 14.0, 7.2 Hz, 1H), 1.05 (t, *J* = 7.3 Hz, 3H), 0.96 (t, *J* = 7.5 Hz, 3H); ^{13}C NMR (100 MHz, $CDCl_3$) δ_C: 220.5, 172.8, 171.3, 139.1, 135.1, 46.1, 39.2, 36.7, 36.2, 34.3, 33.7, 28.0, 26.6, 25.7, 22.0, 21.0, 13.6, 11.6; IR (film) cm^{-1}: 3316, 2961, 2927, 2876, 1732, 1658, 1626, 1521, 1460, 1283, 1175, 756; HRMS (ESI, positive) *m/z* $[M+Na]^+$ Calcd. for $C_{18}H_{25}NNaO_4$: 342.1676, Found: 342.1681.

3. 10. Synthesis of 2e.

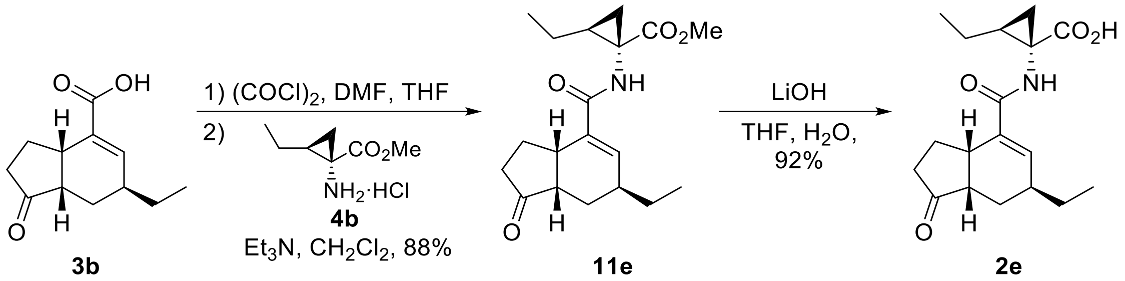

Compound 11e

Prepared according to General Procedure A. $[\alpha]_D^{22}$ +91.8 (*c* 0.23, $CHCl_3$); ^{1}H NMR (400 MHz, $CDCl_3$) δ_H: 6.35 (dd, *J* = 1.9, 1.0 Hz, 1H), 6.02 (brs, 1H), 3.69 (s, 3H), 3.42–3.36 (m, 1H), 2.52 (dd, *J* = 12.6, 4.6 Hz, 1H), 2.30–1.98 (m, 5H), 1.82 (tdd, *J* = 9.0, 7.7, 5.7 Hz, 1H), 1.73–1.62 (m, 2H), 1.48–1.15 (m, 5H), 1.06 (t, *J* = 7.2 Hz, 3H), 0.96 (t, *J* = 7.5 Hz, 3H), 0.90 (dd, *J* = 7.5, 5.1 Hz, 1H); ^{13}C NMR (100 MHz, $CDCl_3$) δ_C: 220.9, 173.0, 170.5, 137.4, 136.2, 52.6, 46.3, 38.0, 36.7, 36.4, 34.0, 30.1, 28.1, 26.3, 25.8, 22.7, 21.9, 13.7, 11.5; IR (film) cm^{-1}: 3312, 2962, 2926, 2877, 1735, 1657, 1627, 1512, 1437, 1336, 1259, 1195, 1163, 744; HRMS (ESI, positive) *m/z* $[M+Na]^+$ Calcd. for $C_{19}H_{27}NNaO_4$: 356.1838, Found: 356.1829.

Compound 2e

Prepared according to General Procedure D. $[\alpha]_D^{22}$ +96.8 (*c* 0.18, $CHCl_3$); ^1H NMR (400 MHz, $CDCl_3$) δ_H: 6.37 (q, *J* = 1.3 Hz, 1H), 6.07 (brs, 1H), 3.40–3.35 (m, 1H), 2.52 (dd, *J* = 12.5, 5.2 Hz, 1H), 2.29–1.97 (m, 5H), 1.85 (tdd, *J* = 9.1, 7.7, 5.3 Hz, 1H), 1.79 (dd, *J* = 9.3, 4.6 Hz, 1H), 1.68 (dsext., *J* = 13.8, 7.0 Hz, 1H), 1.47–1.17 (m, 5H), 1.06 (t, *J* = 7.2 Hz, 3H), 0.96 (t, *J* = 7.4 Hz, 3H), 0.92 (dd, *J* = 7.4, 4.7 Hz, 1H); ^{13}C NMR (100 MHz, $CDCl_3$) δ_C: 220.8, 176.8, 170.9, 138.2, 135.8, 46.2, 37.9, 36.7, 36.3, 34.1, 30.8, 28.0, 26.4, 25.7, 23.1, 21.9, 13.7, 11.5; IR (film) cm^{-1}: 3307, 2961, 2926, 2878, 2856, 1736, 1707, 1659, 1628, 1510, 1449, 1281, 1261, 1195, 1137, 760; HRMS (ESI, positive) *m/z* $[M+Na]^+$ Calcd. for $C_{18}H_{25}NNaO_4$: 342.1676, Found: 342.1674.

3. 11. Synthesis of 2f.

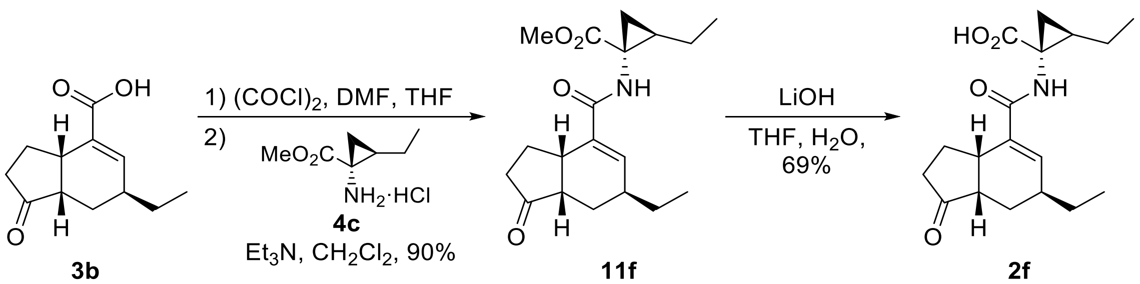

Compound 11f

Prepared according to General Procedure A. $[\alpha]_D^{22}$ +70.7 (*c* 0.20, $CHCl_3$); ^1H NMR (400 MHz, $CDCl_3$) δ_H: 6.30 (dd, *J* = 2.4, 1.0 Hz, 1H), 6.03 (brs, 1H), 3.69 (s, 3H), 3.44–3.39 (m, 1H), 2.52 (dd, *J* = 12.0, 5.8 Hz, 1H), 2.30–1.94 (m, 5H), 1.81 (dd, J = 9.2, 4.6 Hz, 1H), 1.79–1.71 (m, 1H), 1.66 (sept., *J* = 7.1 Hz, 1H), 1.48–1.23 (m, 5H), 1.07 (t, *J* = 7.3 Hz, 3H), 0.95 (t, *J* = 7.4 Hz, 3H), 0.92 (dd, *J* = 6.9, 4.6 Hz, 1H); ^{13}C NMR (100 MHz, $CDCl_3$) δ_C: 220.9, 173.0, 170.5, 137.3, 136.5, 52.6, 46.3, 37.8, 36.7, 36.4, 34.1, 30.2, 28.1, 26.3, 25.8, 23.0, 22.0, 13.7, 11.5; IR (film) cm^{-1}: 3323, 2962, 2927, 2878, 1733, 1654, 1625, 1516, 1436, 1335, 1258, 1196, 1163, 743; HRMS (ESI, positive) *m/z* $[M+Na]^+$ Calcd. for $C_{19}H_{27}NNaO_4$: 356.1838, Found: 356.1829.

Compound 2f

Prepared according to General Procedure D. $[\alpha]_D^{19}$ +66.5 (*c* 0.28, $CHCl_3$); ^1H NMR (400 MHz, $CDCl_3$) δ_H: 6.34 (dd, *J* = 2.3, 1.0 Hz, 1H), 6.07 (brs, 1H), 3.42–3.37 (m, 1H), 2.52 (dd, *J* = 12.3, 5.7 Hz, 1H), 2.29–1.94 (m, 5H), 1.86 (dd, *J* = 9.5, 4.9 Hz, 1H), 1.84–

1.77 (m, 1H), 1.68 (sept., J = 7.0 Hz, 1H), 1.48–1.23 (m, 5H), 1.08 (t, J = 7.3 Hz, 3H), 0.95 (t, J = 7.4 Hz, 3H), 0.95–0.92 (m, 1H); ^{13}C NMR (100 MHz, $CDCl_3$) δ_C: 220.8, 176.4, 170.8, 138.1, 136.1, 46.2, 37.7, 36.7, 36.3, 34.2, 30.8, 28.0, 26.4, 25.7, 23.4, 22.0, 13.7, 11.5; IR (film) cm^{-1}: 3319, 2962, 2925, 2876, 2855, 1732, 1706, 1656, 1626, 1515, 1457, 1282, 1255, 1183, 1139, 756; HRMS (ESI, positive) *m/z* $[M+Na]^+$ Calcd. for $C_{18}H_{25}NNaO_4$: 342.1676, Found: 342.1681.

3. 12. Synthesis of 2g.

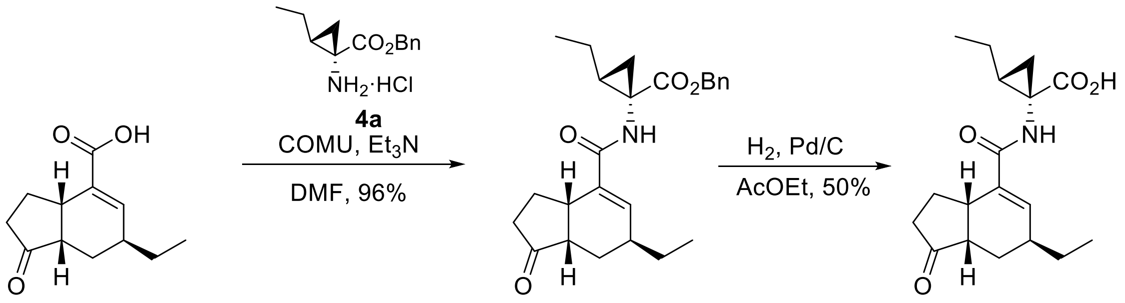

3b 11g 2g

Compound 11g

Prepared according to General Procedure B. $[\alpha]_D^{22}$ +51.7 (*c* 0.38, $CHCl_3$); ^{1}H NMR (400 MHz, $CDCl_3$) δ_H: 7.35–7.30 (m, 5H), 6.27 (t, J = 1.7 Hz, 1H), 6.21 (brs, 1H), 5.16 (d, J = 12.2 Hz, 1H), 5.10 (d, J = 12.2 Hz, 1H), 3.35–3.30 (m, 1H), 2.47 (dd, J = 12.4, 5.0 Hz, 1H), 2.13–1.90 (m, 5H), 1.86–1.79 (m, 1H), 1.66 (quint., J = 7.4 Hz, 1H), 1.65 (quint., J = 7.4 Hz, 1H), 1.63–1.58 (m, 1H), 1.50 (dq, J = 15.5, 7.4 Hz, 1H), 1.39–1.26 (m, 4H), 0.99 (t, J = 7.4 Hz, 3H), 0.91 (t, J = 7.4 Hz, 3H); ^{13}C NMR (100 MHz, $CDCl_3$) δ_C: 220.9, 170.9, 169.6, 137.5, 135.8, 128.5, 128.4, 128.3, 67.3, 46.2, 38.0, 36.4, 36.0, 33.7, 33.6, 27.8, 26.0, 25.6, 23.4, 20.4, 13.5, 11.3; IR (film) cm^{-1}: 3313, 2962, 2931, 2873, 1732, 1658, 1631, 1512, 1458, 1385, 1323, 1161, 752, 698; HRMS (ESI, positive) *m/z* $[M+Na]^+$ Calcd. for $C_{25}H_{31}NNaO_4$: 432.2151, Found: 432.2244.

Compound 2g

Prepared according to General Procedure C. $[\alpha]_D^{23}$ +17.6 (*c* 0.25, $CHCl_3$); ^{1}H NMR (400 MHz, $CDCl_3$) δ_H: 6.40 (dd, J = 2.5, 1.1 Hz, 1H), 6.34 (brs, 1H), 3.40–3.35 (m, 1H), 2.53 (dd, J = 12.8, 5.2 Hz, 1H), 2.31–1.98 (m, 5H), 1.93–1.87 (m, 1H), 1.65–1.49 (m, 4H), 1.47–1.32 (m, 3H), 1.22 (dd, J = 9.6, 5.5 Hz, 1H), 1.06 (t, J = 7.2 Hz, 3H), 0.96 (t, J = 7.5 Hz, 3H); ^{13}C NMR (100 MHz, $CDCl_3$) δ_C: 220.3, 172.2, 171.9, 140.4, 134.9,

46.1, 39.8, 36.6, 36.2, 34.3, 33.7, 27.9, 26.4, 25.6, 21.3, 21.1, 13.5, 11.5; IR (film) cm^{-1}: 3310, 2961, 2925, 2854, 1732, 1657, 1625, 1521, 1460, 1285, 1173, 756; HRMS (ESI, positive) *m/z* $[M+Na]^+$ Calcd. for $C_{18}H_{25}NNaO_4$: 342.1676, Found: 342.1672.

3. 13. Synthesis of 2h.

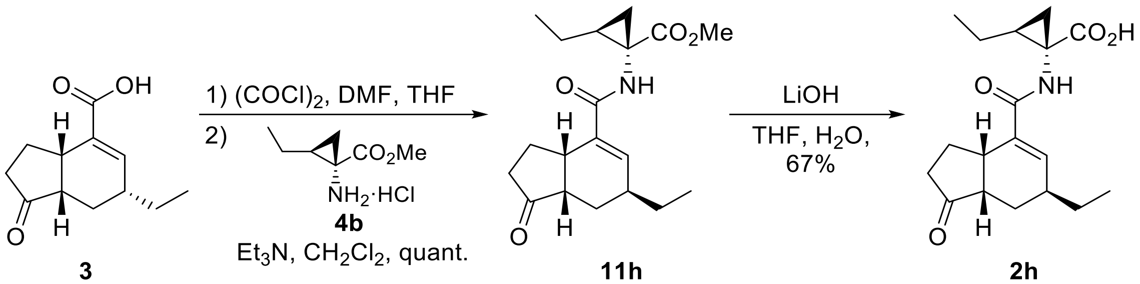

3 11h 2h

Compound 11h

Prepared according to General Procedure A. $[\alpha]_D^{22}$ +57.3 (*c* 0.12, $CHCl_3$); ^{1}H NMR (400 MHz, $CDCl_3$) δ_H: 6.30 (s, 1H), 6.07 (brs, 1H), 3.69 (s, 3H), 3.24–3.15 (m, 1H), 2.54–2.24 (m, 4H), 2.21–2.11 (m, 1H), 1.90 (dt, *J* = 13.6, 4.4 Hz, 1H), 1.85–1.56 (m, 3H), 1.63 (dd, *J* = 12.0, 3.6 Hz, 1H), 1.52 (dquintet, *J* = 8.8, 7.6 Hz, 1H), 1.38 (dquintet, *J* = 8.8, 7.6 Hz, 1H), 1.33–1.19 (m, 1H), 1.07 (td, *J* = 13.2, 10.4 Hz, 1H), 1.06 (t, *J* = 7.2 Hz, 3H), 0.98 (t, *J* = 7.2 Hz, 3H), 0.90 (dd, *J* = 7.6, 5.2 Hz, 1H); ^{13}C NMR (100 MHz, $CDCl_3$) δ_C: 220.5, 173.1, 169.9, 136.4, 136.3, 52.7, 46.5, 38.4, 38.0, 37.4, 36.5, 30.1, 28.3, 27.9, 26.2, 22.8, 21.9, 13.7, 11.6; IR (film) 3313, 2970, 2924, 2889, 1736, 1651, 1512, 1458, 1319, 1277, 945, 744; HRMS (ESI, positive) *m/z* $[M+Na]^+$ Calcd. for $C_{19}H_{27}NNaO_4$: 356.1838, Found: 356.1867.

Compound 2h

Prepared according to General Procedure D. $[\alpha]_D^{21}$ +40.1 (*c* 0.58, $CHCl_3$); ^{1}H NMR (400 MHz, $CDCl_3$) δ_H: 6.34 (s, 1H), 6.17 (brs, 1H), 3.22–3.12 (m, 1H), 2.47 (dt, *J* = 13.6, 7.2 Hz, 1H), 2.40 (dt, *J* = 18.4, 6.8 Hz, 1H), 2.40–2.24 (m, 2H), 2.22–2.11 (m, 1H), 1.90 (dt, *J* = 13.6, 4.4 Hz, 1H), 1.86–1.77 (m, 2H), 1.74–1.56 (m, 2H), 1.51 (dquintet, *J* = 8.6, 7.2 Hz, 1H), 1.37 (dquintet, *J* = 8.6, 7.2 Hz, 1H), 1.28–1.17 (m, 1H), 1.07 (td, *J* = 12.8, 10.8 Hz, 1H), 1.06 (t, *J* = 7.2 Hz, 3H), 0.98 (t, *J* = 7.2 Hz, 3H), 0.94 (dd, *J* = 6.4, 4.0 Hz, 1H); ^{13}C NMR (100 MHz, $CDCl_3$) δ_C: 220.3, 177.6, 170.2, 137.0, 135.8, 46.3, 38.2, 37.7, 37.2, 36.3, 30.7, 29.7, 28.1, 27.8, 25.9, 23.1, 21.8, 13.5, 11.4; IR (film) cm^{-1}: 3309, 2970,

2931, 2873, 1732, 1693, 1658, 1581, 1512, 1462, 1327, 1254, 960, 755; HRMS (ESI, positive) *m/z* $[M+Na]^+$ Calcd. for $C_{18}H_{25}NNaO_4$: 342.1676, Found: 342.1679.

3. 14. Synthesis of 2i.

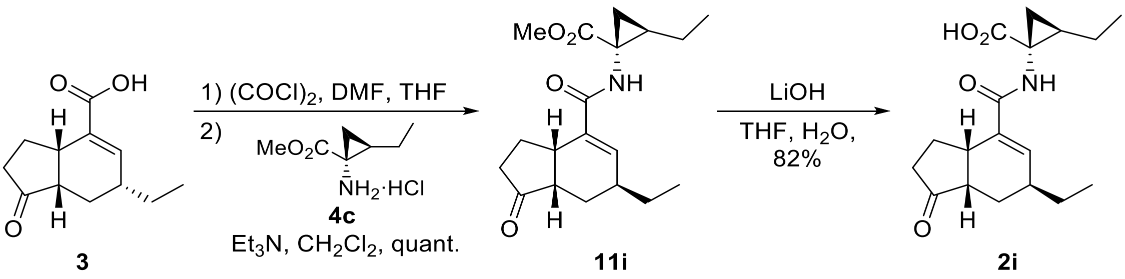

Compound 11i

Prepared according to General Procedure A. $[\alpha]_D^{22}$ +12.0 (*c* 0.17, $CHCl_3$); ^1H NMR (400 MHz, $CDCl_3$) δ_H: 6.30 (s, 1H), 6.09 (brs, 1H), 3.69 (s, 3H), 3.27–3.18 (m, 1H), 2.48–2.25 (m, 4H), 2.20–2.11 (m, 1H), 1.91 (dt, *J* = 12.8, 5.2 Hz, 1H), 1.82–1.75 (m, 1H), 1.70–1.56 (m, 3H), 1.51 (dquintet, *J* = 8.2, 7.2 Hz, 1H) , 1.39 (dquintet, *J* = 8.0, 7.2 Hz, 1H), 1.30–1.20 (m, 1H), 1.07 (td, *J* = 13.2, 11.8 Hz, 1H), 1.06 (t, *J* = 7.6 Hz, 3H), 0.99 (t, *J* = 7.6 Hz, 3H), 0.91 (d, *J* = 2.4 Hz, 1H); ^{13}C NMR (100 MHz, $CDCl_3$) δ_C: 220.5, 173.1, 169.7, 136.5, 136.4, 52.7, 46.4, 38.4, 37.9, 37.4, 36.4, 30.2, 28.3, 27.8, 26.2, 23.0, 22.0, 13.7, 11.5; IR (film) cm^{-1}: 3325, 2958, 2927, 2881, 1736, 1658, 1516, 1458, 1334, 1265, 1192, 1152, 837, 752; HRMS (ESI, positive) *m/z* $[M+Na]^+$ Calcd. for $C_{19}H_{27}NNaO_4$: 356.1838, Found: 356.1844.

Compound 2i

Prepared according to General Procedure D. $[\alpha]_D^{24}$ +18.0 (*c* 0.19, $CHCl_3$); ^1H NMR (400 MHz, $CDCl_3$) δ_H: 6.34 (s, 1H), 6.20 (brs, 1H), 3.23–3.17 (m, 1H), 2.48–2.25 (m, 4H), 2.20–2.11 (m, 1H), 1.90 (dt, *J* = 12.8, 4.8 Hz, 1H), 1.86–1.80 (m, 2H), 1.72–1.63 (m, 1H), 1.59 (td, *J* = 11.2, 3.2 Hz, 1H), 1.50 (sept, *J* = 7.2 Hz, 1H), 1.39 (sept, *J* = 7.2 Hz, 1H), 1.30–1.20 (m, 1H), 1.08 (td, *J* =13.0, 11.6 Hz, 1H), 1.07 (t, *J* = 7.6 Hz, 3H), 0.98 (t, *J* = 7.6 Hz, 3H), 0.95 (d, *J* = 2.4 Hz, 1H); ^{13}C NMR (100 MHz, $CDCl_3$) δ_C: 220.3, 177.6, 169.9, 136.9, 135.9, 46.2, 38.2, 37.6, 37.2, 36.1, 30.8, 29.7, 28.1, 27.7, 26.0, 23.4, 21.8, 13.5, 11.4; IR (film) cm^{-1}: 3313, 2962, 2935, 2877, 1732, 1658, 1520, 1269, 1172, 841, 756; HRMS (ESI, positive) *m/z* $[M+Na]^+$ Calcd. for $C_{18}H_{25}NNaO_4$: 342.1676, Found: 342.1682.

3. 15. Synthesis of 2j.

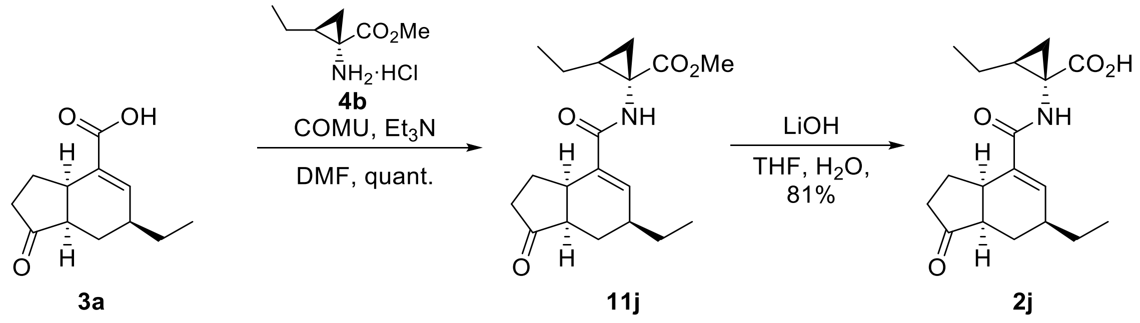

Compound 11j

Prepared according to General Procedure B. [α]$_D^{22}$ −11.5 (*c* 0.11, $CHCl_3$); ^1H NMR (400 MHz, $CDCl_3$) δ_H: 6.30 (s, 1H), 6.08 (brs, 1H), 3.69 (s, 3H), 3.28–3.18 (m, 1H), 2.49–2.25 (m, 4H), 2.20–2.10 (m, 1H), 1.90 (dt, *J* = 12.8, 4.8 Hz, 1H), 1.81–1.75 (m, 1H), 1.71–1.55 (m, 3H), 1.51 (dquintet, *J* = 8.2, 7.2 Hz, 1H), 1.39 (dquintet, *J* = 8.0, 7.2 Hz, 1H), 1.30–1.20 (m, 1H), 1.07 (td, *J* = 13.0, 11.6 Hz, 1H), 1.06 (t, *J* = 7.6 Hz, 3H), 0.99 (t, *J* = 7.6 Hz, 3H), 0.91 (d, *J* = 2.8 Hz, 1H); ^{13}C NMR (100 MHz, $CDCl_3$) δ_C: 220.4, 172.9, 169.6, 136.3, 136.2, 52.5, 46.3, 38.2, 37.8, 37.2, 36.2, 30.0, 28.2, 27.6, 26.0, 22.9, 21.8, 13.5, 11.4; IR (film) cm^{-1}: 3326, 2958, 2927, 2866, 1739, 1655, 1512, 1462, 1365, 1259, 1195, 1163, 837, 744; HRMS (ESI, positive) *m/z* $[M+Na]^+$ Calcd. for $C_{19}H_{27}NNaO_4$: 356.1838, Found: 356.1867.

Compound 2j

Prepared according to General Procedure D. [α]$_D^{24}$ –17.6 (*c* 0.26, $CHCl_3$); ^1H NMR (400 MHz, $CDCl_3$) δ_H: 6.30 (s, 1H), 6.08 (brs, 1H), 3.25–3.15 (m, 1H), 2.47–2.25 (m, 4H), 2.20–2.10 (m, 1H), 1.90 (dt, *J* = 13.2, 4.4 Hz, 1H), 1.86–1.80 (m, 2H), 1.73–1.63 (m, 1H), 1.58 (td, *J* = 12.0, 3.6 Hz, 1H), 1.50 (sept, *J* = 7.2 Hz, 1H), 1.39 (sept, *J* = 7.2 Hz, 1H), 1.30–1.20 (m, 1H), 1.08 (td, *J* =12.4, 11.4 Hz, 1H), 1.07 (t, *J* = 7.6 Hz, 3H), 0.98 (t, *J* = 7.6 Hz, 3H), 0.93 (d, *J* = 1.6 Hz, 1H); ^{13}C NMR (100 MHz, $CDCl_3$) δ_C: 220.2, 177.5, 170.0, 137.0, 135.9, 46.2, 38.2, 37.6, 37.2, 36.1, 30.8, 29.7, 28.1, 27.6, 26.0, 23.3, 21.8, 13.5, 11.3; IR (film) cm^{-1}: 3321, 2962, 2931, 2873, 1732, 1662, 1520, 1458, 1269, 1180, 841, 760; HRMS (ESI, positive) *m/z* $[M+Na]^+$ Calcd. for $C_{18}H_{25}NNaO_4$: 342.1676, Found: 342.1677.

3. 16. Synthesis of 2k.

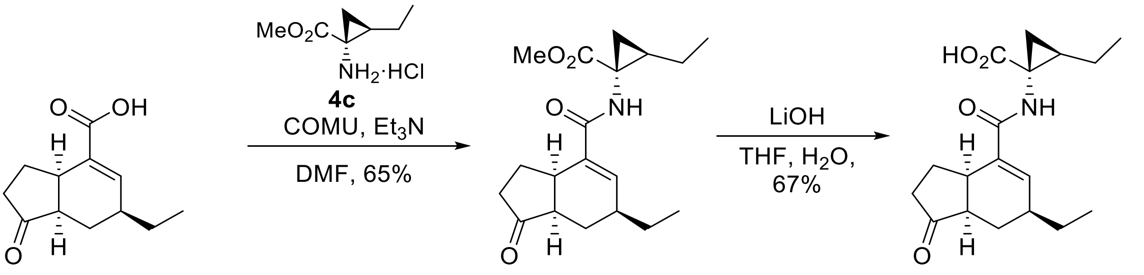

Compound 11k

Prepared according to General Procedure B. $[\alpha]_D^{22}$ −53.1 (*c* 0.12, $CHCl_3$); ^{1}H NMR (400 MHz, $CDCl_3$) δ_H: 6.30 (s, 1H), 6.08 (brs, 1H), 3.69 (s, 3H), 3.24–3.15 (m, 1H), 2.54–2.24 (m, 4H), 2.21–2.11 (m, 1H), 1.90 (dt, *J* = 13.6, 4.4 Hz, 1H), 1.85–1.58 (m, 3H), 1.63 (dd, *J* = 11.6, 2.8 Hz, 1H), 1.52 (dquintet, *J* = 8.6, 7.6 Hz, 1H), 1.39 (dquintet, *J* = 8.6, 7.6 Hz, 1H), 1.29–1.15 (m, 1H), 1.07 (td, *J* = 13.2, 10.8 Hz, 1H), 1.06 (t, *J* = 7.2 Hz, 3H), 0.98 (t, *J* = 7.2 Hz, 3H), 0.90 (dd, *J* = 7.6, 5.6 Hz, 1H); ^{13}C NMR (100 MHz, $CDCl_3$) δ_C: 220.4, 172.9, 169.8, 136.3, 136.1, 52.5, 46.3, 38.2, 37.8, 37.2, 36.4, 29.9, 28.2, 27.8, 26.0, 22.6, 21.7, 13.5, 11.4; IR (film) cm^{-1}: 3312, 2970, 2935, 2876, 1739, 1682, 1515, 1462, 1338, 1261, 833, 760; HRMS (ESI, positive) *m/z* $[M+Na]^+$ Calcd. for $C_{19}H_{27}NNaO_4$: 356.1838, Found: 356.1844.

Compound 2k

Prepared according to General Procedure D. $[\alpha]_D^{20}$ −41.7 (*c* 0.39, $CHCl_3$); ^{1}H NMR (400 MHz, $CDCl_3$) δ_H: 6.34 (s, 1H), 6.17 (brs, 1H), 3.22–3.14 (m, 1H), 2.47 (dt, *J* = 12.4, 8.0 Hz, 1H), 2.42–2.24 (m, 3H), 2.21–2.12 (m, 1H), 1.90 (dt, *J* = 13.2, 5.6 Hz, 1H), 1.86–1.78 (m, 2H), 1.74–1.56 (m, 2H), 1.51 (dquintet, *J* = 8.4, 7.2 Hz, 1H), 1.38 (dquintet, *J* = 8.4, 7.2 Hz, 1H), 1.28–1.17 (m, 1H), 1.07 (td, *J* =12.6, 10.6 Hz, 1H), 1.06 (t, *J* = 7.6 Hz, 3H), 0.98 (t, *J* = 7.6 Hz, 3H), 0.94 (dd, *J* = 6.4, 4.4 Hz, 1H); ^{13}C NMR (100 MHz, $CDCl_3$) δ_C: 220.2, 177.5, 170.2, 137.0, 135.8, 46.3, 38.2, 37.7, 37.2, 36.3, 30.7, 29.7, 28.1, 27.8, 26.0, 23.1, 21.7, 13.5, 11.4; IR (film) cm^{-1}: 3313, 2966, 2935, 2873, 1731, 1658, 1516, 1458, 1265, 1176, 841, 756; HRMS (ESI, positive) *m/z* $[M+Na]^+$ Calcd. for $C_{18}H_{25}NNaO_4$: 342.1676, Found: 342.1674.

3. 17. Synthesis of 2l.

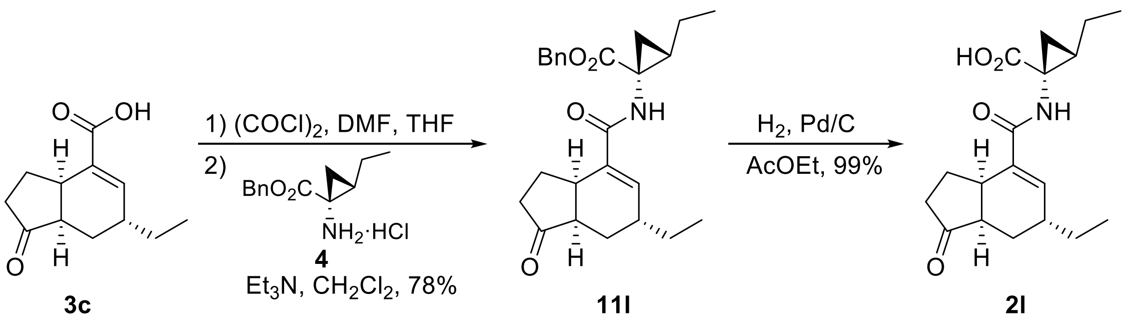

Compound 11l

Prepared according to General Procedure A. $[\alpha]_D^{22}$ −51.2 (*c* 0.28, $CHCl_3$); ^{1}H NMR (400 MHz, $CDCl_3$) δ_H: 7.36–7.30 (m, 5H), 6.28 (s, 1H), 6.23 (brs, 1H), 5.16 (d, *J* = 12.4 Hz, 1H), 5.10 (d, *J* = 12.4 Hz, 1H), 3.36–3.28 (m, 1H), 2.70, (dd, *J* = 12.4, 4.4 Hz, 1H), 2.13–1.89 (m, 5H), 1.85–1.78 (m, 1H), 1.66 (ddd, *J* = 14.8, 7.2, 1.6 Hz, 2H), 1.60 (dd, *J* = 8.0, 4.8 Hz, 1H), 1.50 (quintet, *J* = 7.8 Hz, 1H), 1.35–1.25 (m, 4H), 0.99 (t, *J* = 7.6 Hz, 3H), 0.90 (t, *J* = 7.6 Hz, 3H); ^{13}C NMR (100 MHz, $CDCl_3$) δ_C: 220.8, 170.9, 169.7, 137.6, 135.7, 135.5, 128.5, 128.4, 128.3, 46.2, 38.0, 36.4, 36.0, 33.7, 33.6, 27.9, 26.1, 25.6, 23.5, 20.4, 13.5, 11.3; IR (film) cm^{-1}: 3325, 2962, 2935, 2873, 1732, 1658, 1628, 1512, 1458, 1385, 1273, 1161, 841, 748; HRMS (ESI, positive) *m/z* $[M+Na]^+$ Calcd. for $C_{25}H_{31}NNaO_4$: 432.2151, Found: 432.2134.

Compound 2l

Prepared according to General Procedure C. $[\alpha]_D^{21}$ −18.1 (*c* 0.65, $CHCl_3$); ^{1}H NMR (400 MHz, $CDCl_3$) δ_H: 6.47 (s, 1H), 6.40 (brs, 1H), 3.40–3.32 (m, 1H), 2.53 (dd, *J* = 12.4, 4.8 Hz, 1H), 2.31–2.10 (m, 2H), 2.20–1.89 (m, 3H), 2.06 (dd, *J* = 18.8, 5.6 Hz, 1H), 1.62 (ddd, *J* = 14.0, 6.8, 4.0 Hz, 2H), 1.58 (dd, *J* = 8.0, 5.2 Hz, 1H), 1.53–1.24 (m, 5H), 1.05 (t, *J* = 7.6 Hz, 3H), 0.95 (t, *J* = 7.6 Hz, 3H); ^{13}C NMR (100 MHz, $CDCl_3$) δ_C: 220.5, 173.9, 171.4, 139.6, 134.9, 46.1, 39.0, 36.4, 36.0, 34.0, 33.8, 26.2, 25.5, 21.8, 20.9, 13.4, 11.4; IR (film) cm^{-1}: 3310, 2962, 2931, 2873, 1732, 1655, 1624, 1516, 1450, 1288, 1173, 953, 752; HRMS (ESI, positive) *m/z* $[M+Na]^+$ Calcd. For $C_{18}H_{25}NNaO_4$: 342.1676, Found: 342.1688.

3. 18. Synthesis of 2m.

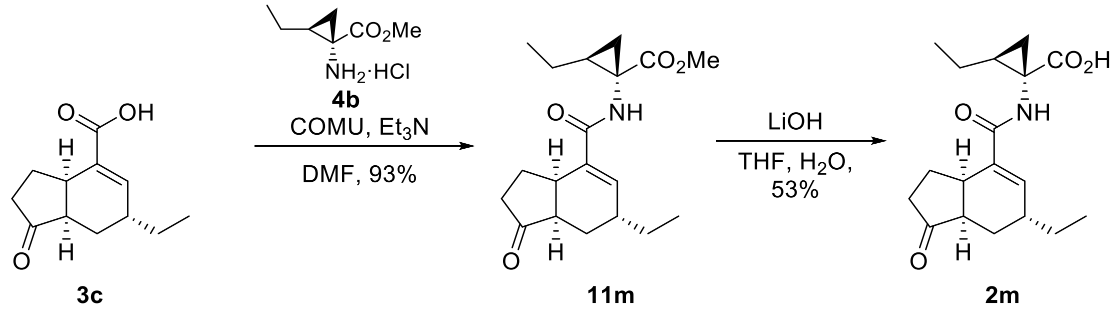

Compound 11m

Prepared according to General Procedure B. $[\alpha]_D^{22}$ −70.4 (*c* 0.16, $CHCl_3$); ^1H NMR (400 MHz, $CDCl_3$) δ_H: 6.30 (s, 1H), 6.04 (brs, 1H), 3.69 (s, 3H), 3.45–3.37 (m, 1H), 2.53 (dd, J = 12.2, 4.7 Hz, 1H), 2.31–1.97 (m, 5H), 1.84–1.70 (m, 2H), 1.66 (sept, J = 7.1 Hz, 1H), 1.48–1.16 (m, 5H), 1.07 (t, J = 7.4 Hz, 3H), 0.95 (t, J = 7.4 Hz, 3H), 0.92 (dd, J = 6.8, 4.4 Hz, 1H); ^{13}C NMR (100 MHz, $CDCl_3$) δ_C:220.9, 173.0, 170.4, 137.3, 136.4, 52.5, 46.2, 37.7, 36.6, 36.2, 33.9, 30.0, 27.9, 26.2, 25.7, 23.0, 21.9, 13.6, 11.4; IR (film) cm^{-1}: 3325, 2958, 2931, 2878, 1731, 1655, 1624, 1516, 1437, 1335, 1261, 1196, 1165, 744; HRMS (ESI, positive) *m/z* $[M+Na]^+$ Calcd. for $C_{19}H_{27}NNaO_4$: 356.1837, Found: 356.1857.

Compound 2m

Prepared according to General Procedure D. $[\alpha]_D^{24}$ −69.4 (*c* 0.52, $CHCl_3$); ^1H NMR (400 MHz, $CDCl_3$) δ_H: 6.34 (s, 1H), 6.17 (brs, 1H), 3.42–3.35 (m, 1H), 2.53 (dd, J = 12.0, 4.4 Hz, 1H), 2.30–2.10 (m, 3H), 2.06 (dd, J = 18.4, 5.6 Hz, 1H), 2.02–1.92 (m, 2H), 1.89–1.78 (m, 2H), 1.67 (sept, J = 7.0 Hz, 1H), 1.46–1.23 (m, 4H), 1.08 (t, J = 7.6 Hz, 3H), 0.98–0.95 (m, 1H), 0.95 (t, J = 7.6 Hz, 3H); ^{13}C NMR (100 MHz, $CDCl_3$) δ_C: 220.8, 177.3, 170.6, 137.9, 136.0, 46.1, 37.5, 36.5, 36.1, 34.0, 30.8, 29.7, 27.8, 26.2, 25.6, 21.8, 13.5, 11.4; IR (film) cm^{-1}: 3305, 2962, 2931, 2873, 1732, 1658, 1631, 1519, 1454, 1261, 1176, 1053, 837, 760; HRMS (ESI, positive) *m/z* $[M+Na]^+$ Calcd. for $C_{18}H_{25}NNaO_4$: 342.1676, Found: 342.1687.

3. 19. Synthesis of 2n.

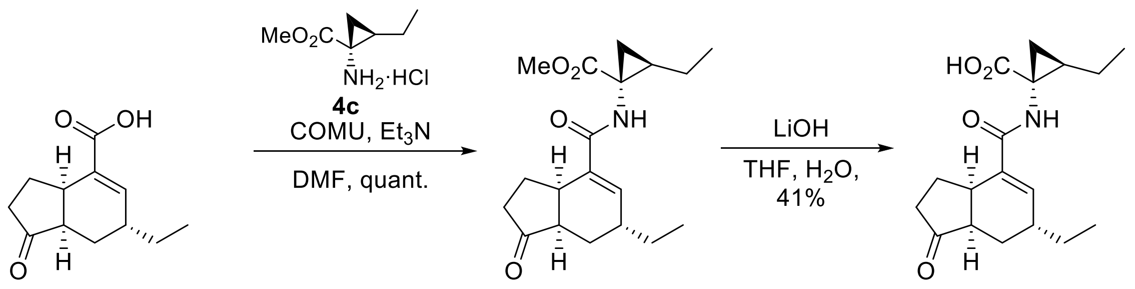

3c **11n** **2n**

Compound 11n

Prepared according to General Procedure B. [α]$_D^{22}$ −94.9 (*c* 0.24, $CHCl_3$); ^{1}H NMR (400 MHz, $CDCl_3$) δ_H: 6.35 (s, 1H), 6.02 (brs, 1H), 3.69 (s, 3H), 3.44–3.33 (m, 1H), 2.52 (dd, *J* = 12.3, 4.8 Hz, 1H), 2.32–1.95 (m, 6H), 1.80 (tdd, *J* = 9.3, 7.8, 5.7 Hz, 1H), 1.75–1.63 (m, 2H), 1.49–1.29 (m, 3H), 1.20 (sextetd, *J* = 8.3, 6.9 Hz, 1H), 1.05 (t, *J* = 7.4 Hz, 3H), 0.96 (t, *J* = 7.3 Hz, 3H), 0.89 (dd, *J* = 7.4, 4.9 Hz, 1H); ^{13}C NMR (100 MHz, $CDCl_3$) δ_C: 220.9, 172.9, 170.4, 137.3, 136.0, 52.5, 46.2, 37.9, 36.6, 36.2, 33.9, 30.0, 27.9, 26.2, 25.7, 22.6, 21.8, 13.6, 11.5; IR (film) cm^{-1}: 3313, 2954, 2935, 2877, 1732, 1658, 1628, 1512, 1335, 1261, 1195, 1161, 744 HRMS (ESI, positive) *m/z* [M+Na]$^+$ Calcd. for $C_{19}H_{27}NNaO_4$: 356.1838, Found: 356.1854.

Compound 2n

Prepared according to General Procedure D. [α]$_D^{20}$ −96.3 (*c* 0.17, $CHCl_3$); ^{1}H NMR (400 MHz, $CDCl_3$) δ_H: 6.38 (s, 1H), 6.16 (brs, 1H), 3.41–3.33 (m, 1H), 2.53 (dd, *J* = 12.0, 5.2 Hz, 1H), 2.31–2.11 (m, 3H), 2.07 (dd, *J* = 13.6, 7.6 Hz, 1H), 2.04–1.95 (m, 2H), 1.86 (qd, *J* = 7.6, 5.6 Hz, 1H), 1.78 (dd, *J* = 9.6, 5.2 Hz, 1H), 1.69 (sept, *J* = 6.4 Hz, 1H), 1.48–1.30 (m, 3H), 1.21 (sept, *J* = 6.4 Hz, 1H), 1.06 (t, *J* = 7.6 Hz, 3H), 0.96–0.93 (m, 1H), 0.95 (t, *J* = 7.6 Hz, 3H); ^{13}C NMR (100 MHz, $CDCl_3$) δ_C: 221.0, 177.6, 170.9, 138.2, 135.5, 46.1, 37.6, 36.4, 36.1, 33.9, 30.8, 27.8, 26.1, 25.5, 23.1, 21.7, 13.5, 11.4; IR (film) cm^{-1}: 3313, 2962, 2935, 2877, 1728, 1705, 1658, 1631, 1516, 1454, 1269, 1172, 840, 756; HRMS (ESI, positive) *m/z* [M+Na]$^+$ Calcd. for $C_{18}H_{25}NNaO_4$: 342.1676, Found: 342.1682.

3. 20. Synthesis of 2o.

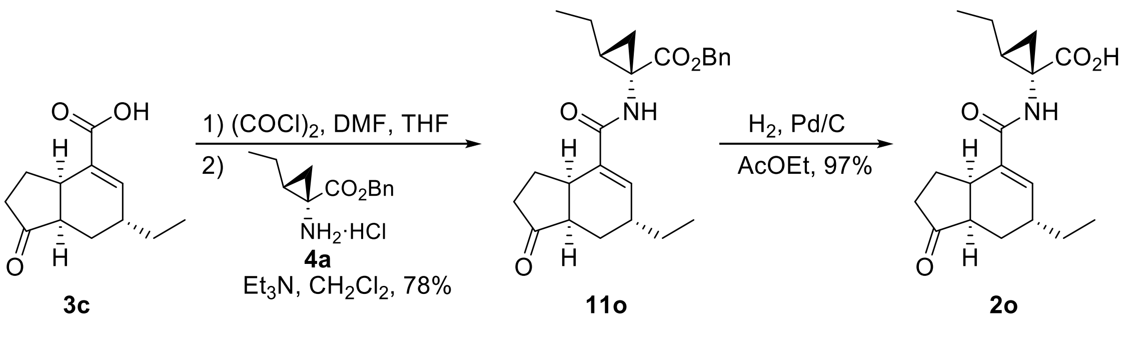

Compound 11o

Prepared according to General Procedure A. $[\alpha]_D^{22}$ −107.6 (*c* 0.465, $CHCl_3$); ^1H NMR (400 MHz, $CDCl_3$) δ_H: 7.36–7.28 (m, 5H), 6.27 (s, 1H), 6.23 (brs, 1H), 5.17 (d, *J* = 12.0 Hz, 1H), 5.10 (d, *J* = 12.0 Hz, 1H), 3.37–3.29 (m, 1H), 2.46, (dd, *J* = 12.4, 4.8 Hz, 1H), 2.11–1.87 (m, 5H), 1.82–1.74 (m, 1H), 1.65 (dd, *J* = 13.2, 7.2 Hz, 2H), 1.59 (dd, *J* = 12.8, 6.0 Hz, 1H), 1.55–1.46 (m, 1H), 1.38–1.24 (m, 4H), 0.94 (t, *J* = 6.8 Hz, 3H), 0.91 (t, *J* = 7.2 Hz, 3H); ^{13}C NMR (100 MHz, $CDCl_3$) δ_C: 221.0, 170.8, 169.8, 137.4, 136.0, 135.5, 128.5, 128.5, 128.5, 67.3, 46.3, 38.1, 36.3, 36.1, 33.7, 33.2, 27.9, 26.0, 25.6, 23.2, 20.3, 13.5, 11.3; IR (film) cm^{-1}: 3282, 2962, 2931, 2869, 1731, 1658, 1627, 1515, 1457, 1380, 1322, 1280, 1160, 1002, 794, 744; HRMS (ESI, positive) *m/z* $[M+Na]^+$ Calcd. for $C_{25}H_{31}NNaO_4$: 432.2151, Found: 432.2184.

Compound 2o

Prepared according to General Procedure C. $[\alpha]_D^{24}$ −134.2 (*c* 0.21, $CHCl_3$); ^1H NMR (400 MHz, $CDCl_3$) δ_H: 6.53 (s, 1H), 6.35 (brs, 1H), 3.40–3.30 (m, 1H), 2.53 (dd, *J* = 12.4, 4.8 Hz, 1H), 2.32–2.08 (m, 3H), 2.05 (dd, *J* = 13.2, 6.0 Hz, 1H), 2.01–1.91 (m, 2H), 1.68–1.59 (m, 3H), 1.54 (quintet, *J* = 7.6 Hz, 1H), 1.46–1.29 (m, 4H), 1.02 (t, *J* = 7.2 Hz, 3H), 0.95 (t, *J* = 7.6 Hz, 3H); ^{13}C NMR (100 MHz, $CDCl_3$) δ_C: 221.2, 175.5, 171.1, 138.9, 135.1, 46.1, 38.4, 36.4, 36.0, 33.9, 27.8, 26.1, 25.5, 23.0, 20.5, 13.4, 11.3; IR (film) cm^{-1}: 3290, 2962, 2935, 2873, 1727, 1658, 1628, 1520, 1458, 1288, 1168, 922, 756; HRMS (ESI, positive) *m/z* $[M+Na]^+$ Calcd. for $C_{18}H_{25}NNaO_4$: 342.1676, Found: 342.1654. ach stereoisomer. Two isomers in the same box are each enantiomer.

GFP-fused JAZ protein degradation assay Four-day-old seedlings on a half-strength MS plate were transferred to a half-strength MS liquid medium in the presence or absence of each compound. After treatment for 2 h, each seedling was flash-frozen, and homogenized with extraction buffer (50 μL; 50 mM Tris-HCl, pH 7.4, 100 mM NaCl, 10% glycerol, 0.1% Tween-20, 1 mM DTT, 1 mM phenylmethylsulfonyl fluoride (PMSF), cOmplete protease inhibitor (Roche) and 50 μM MG132). After centrifugation (15,000 *g*, 4 ºC, 10 min), each supernatant was collected. Extracts were subjected to SDS-PAGE and analyzed by western blotting. SDS-PAGE and western blotting were carried out using a Mini-Protean III electrophoresis apparatus (Bio-Rad, Hercules, CA). Chemiluminescence was observed on Amersham Imager 680 (Cytiva, US).

***In silico* MD simulation and docking analyses** The initial structure of the COI1-**2**-JAZ1 complex was obtained from the crystal structure (PDB ID: 3OGM). COI1-**2**-JAZ9 complex was obtained according to our previous report.[4] In order to model the complex structure of the COI1-**2**-$InsP_8$-JAZ9, the structures of inositol phosphates (PO_4) in the COI1-**2**-JAZ9 complex were removed, and then the $InsP_8$ structure extracted from the other protein structure (PDB ID: 3T9F) was docked to the removed sites of PO_4. The structure of COI1-**2e**-$InsP_8$-JAZ9 was prepared by replacing **2** with **2e** by docking simulation. DOCK 6.6 software was used for the docking of the $InsP_8$ and **2e**, and Amber99 force field parameters were assigned for the estimations of grid score. The space of the conformation search was defined in a 12 Å radius from the center of the binding sites of COI1. The best score pose was used for the subsequent MD simulations in a water solvent. Five independent 100 ns MD simulations with different initial velocities were performed to sample the equilibrated structures of COI1-**2**-$InsP_8$-JAZ9 and COI1-**2e**-$InsP_8$-JAZ9. A Parrinello-Rahman type thermostat[5] and a Nosé-Hoover type barostat[6] were adopted to control the system temperature (T = 300 K) and pressure (P = 1 atm). The force field parameters of CHARMM36[7], generalized charmm force field[8], and TIP3P water model[9] were assigned for the protein, ligand, and water molecule, respectively. The cutoff length for the actual space was set to 12 Å. The Particle mesh Ewald (PME) method[10] was used to treat long-range electrostatics. The integration time step of the system was 2 fs, and the MD structures were stored every 10 ps. All MD calculations were done using the GROMACS 2018 program package. Radial distribution functions (RDFs) were calculated to analyze the hydrogen bond networks between the compounds

and surrounding residues in the binding pocket of COI1. The RDF curves for possible hydrogen bond pairs were evaluated using a total 500 ns term MD simulation. All MD trajectory data (toral 500 ns) were used for the RDF analysis.

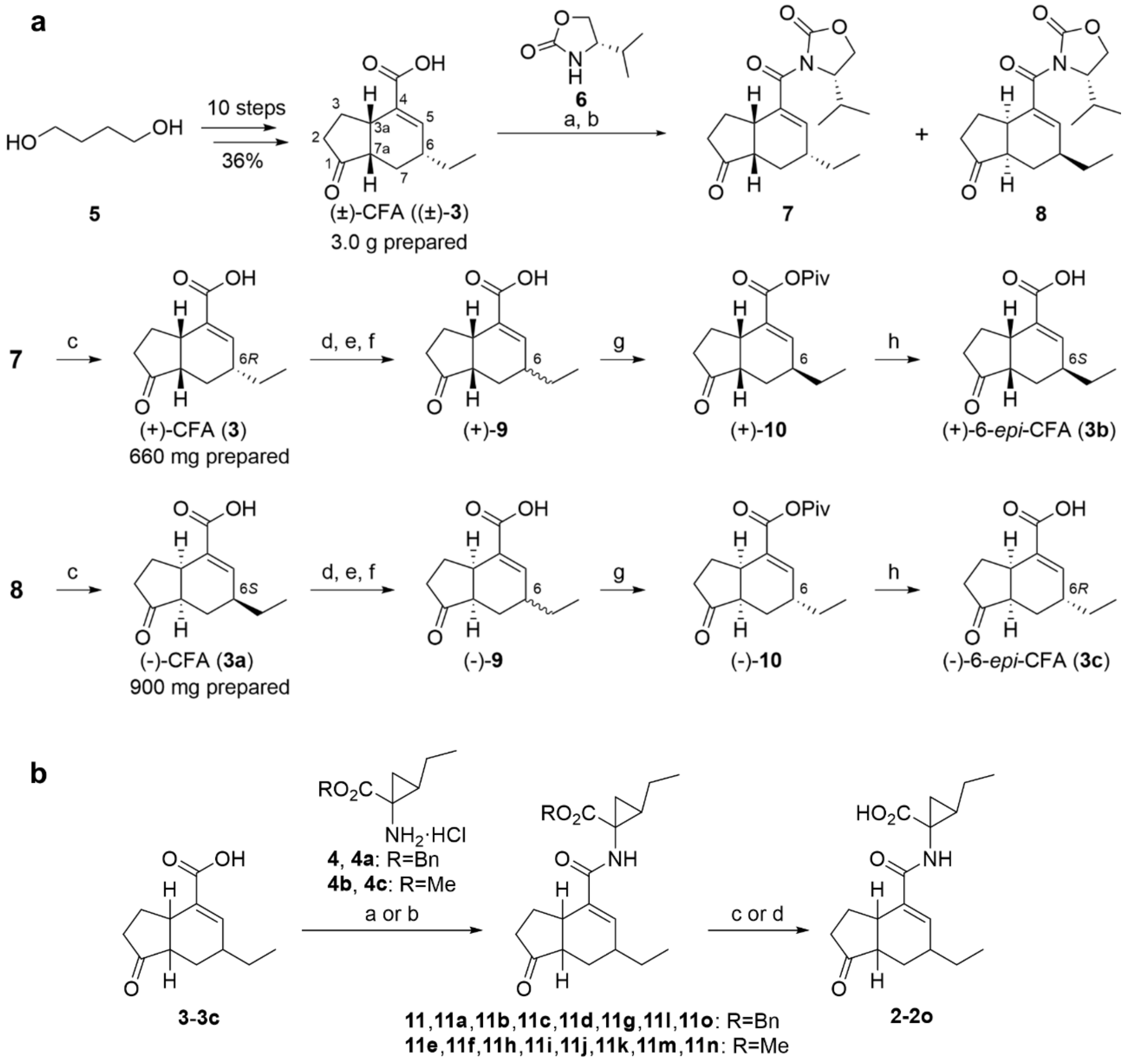

Supplementary Fig. 1. Chemical synthesis of 2-stereoisomers. We first prepared 3.0 g of a racemic mixture of (±)-**3** from 1,4-butanediol (**5**) according to the modified Watson's procedure.[1, 11] Coupling between (±)-**3** and the chiral auxiliary (*S*)-4-isopropyl-2-oxazolidinone (**6**) gave a diastereomer mixture of **7** and **8** [12] which was separated to give pure **7** and **8**. Separated **7** was treated with lithium hydroperoxide to afford 660 mg of optically pure (+)-**3**. Methyl ester (+)-**9** was subsequently isomerized with DBU, and the resulting diastereomer mixture was separated to afford pure diastereomers (+)-**10**, which was hydrolyzed to give 55.1 mg of optically pure (+)-6*epi*-CFA (**3b**). (-)-CFA (**3a**) and (-)-6*epi*-CFA (**3c**) were also obtained from **8** by the same procedure.

(**a**) Synthetic scheme of CFA and the stereoisomers (**3**-**3c**). Reagents and conditions: (a) **6**, PivCl, LiCl, Et_3N, DMAP, CH_2Cl_2; (b) SiO_2 chromatography, **7** (49%), **8** (48%); (c)

LiOH, H_2O_2, THF, H_2O, **3** (89%), **3a** (83%); (d) MeI, K_2CO_3, DMF; (e) DBU, benzene, reflux; (f) HCl, reflux, (+)-**9** (85% (3 steps), 6*R*:6*S*=3:2), (-)-**9** (88% (3 steps), 6*R*:6*S*=2:3); (g) PivCl, Et_3N, CH_2Cl_2, (+)-**10** (25%) + 6*R* isomer (34%), (-)-**10** (33%) + 6*S* isomer (44%); (h) LiOH, THF, H_2O, **3b** (89%), **3c** (quant.). (**b**) Synthetic scheme of 16 stereochemical isomers of **2**. Reagents and conditions: (a) $(COCl)_2$, DMF, THF; **4-4c**, Et_3N, CH_2Cl_2, **11** (91%), **11a** (90%), **11b** (90%), **11c** (99%), **11d** (77%), **11e** (88%), **11f** (90%), **11h** (quant.), **11i** (quant.), **11l** (78%), **11o** (78%); (b) COMU, Et_3N, DMF, **11g** (96%), **11j** (quant.), **11k** (65%), **11m** (93%), **11n** (quant.), (c) H_2, Pd/C, AcOEt, **2** (46.1 mg, 96%), **2a** (40.6 mg, 94%), **2b** (43.8 mg, 81%), **2c** (38.9 mg, 84%), **2d** (39.5 mg, 97%), **2g** (2.1 mg, 50%), **2l** (40.8 mg, 99%), **2o** (31.5 mg, 97%); (d) LiOH, THF, H_2O, **2e** (69.4 mg, 92%), **2f** (32.9 mg, 69%), **2h** (34.6 mg, 67%), **2i** (43.4 mg, 82%), **2j** (1.7 mg, 81%), **2k** (1.6 mg, 67%), **2m** (1.7 mg, 53%), **2n** (2.6 mg, 41%).

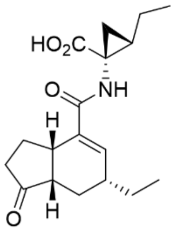

2

$[\alpha]_D^{22}$ +97.6 (*c* 0.15, $CHCl_3$)

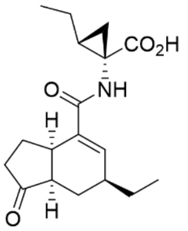

2a

$[\alpha]_D^{22}$ −96.8 (*c* 0.20, $CHCl_3$)

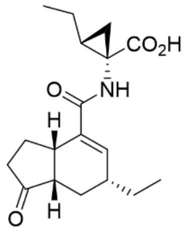

2b

$[\alpha]_D^{22}$ −35.8 (*c* 0.53, $CHCl_3$)

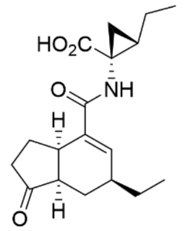

2c

$[\alpha]_D^{22}$ +37.0 (*c* 0.60, $CHCl_3$)

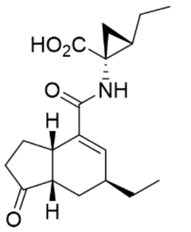

2d

$[\alpha]_D^{22}$ +138.3 (*c* 0.23, $CHCl_3$)

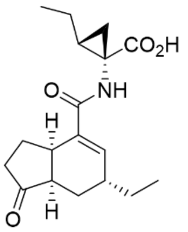

2o

$[\alpha]_D^{24}$ −134.2 (*c* 0.21, $CHCl_3$)

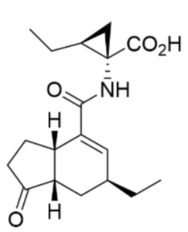

2e

$[\alpha]_D^{22}$ +96.8 (*c* 0.18, $CHCl_3$)

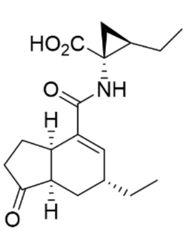

2n

$[\alpha]_D^{20}$ −96.3 (*c* 0.17, $CHCl_3$)

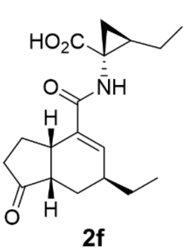

2f

$[\alpha]_D^{19}$ +66.5 (*c* 0.28, $CHCl_3$)

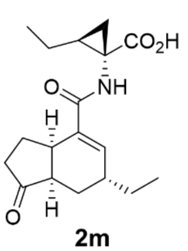

2m

$[\alpha]_D^{24}$ −69.4 (*c* 0.52, $CHCl_3$)

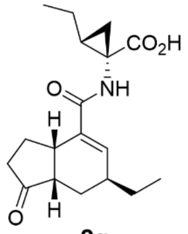

2g

$[\alpha]_D^{23}$ +17.6 (*c* 0.25, $CHCl_3$)

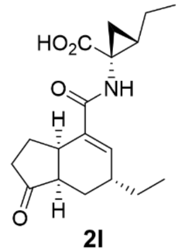

2l

$[\alpha]_D^{21}$ −18.1 (*c* 0.65, $CHCl_3$)

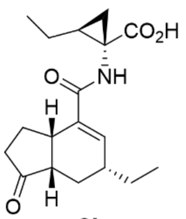

2h

$[\alpha]_D^{21}$ +40.1 (*c* 0.58, $CHCl_3$)

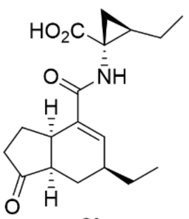

2k

$[\alpha]_D^{20}$ −41.7 (*c* 0.39, $CHCl_3$)

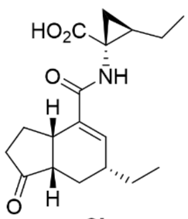

2i

$[\alpha]_D^{24}$ +18.0 (*c* 0.19, $CHCl_3$)

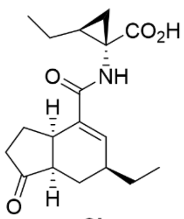

2j

$[\alpha]_D^{24}$ −17.6 (*c* 0.26, $CHCl_3$)

Supplementary Fig. 2. Specific optical rotations of 2-stereoisomers. Two isomers in the same box are each enantiomer. Opposite signs of the optical rotations were observed in each enantiomer.

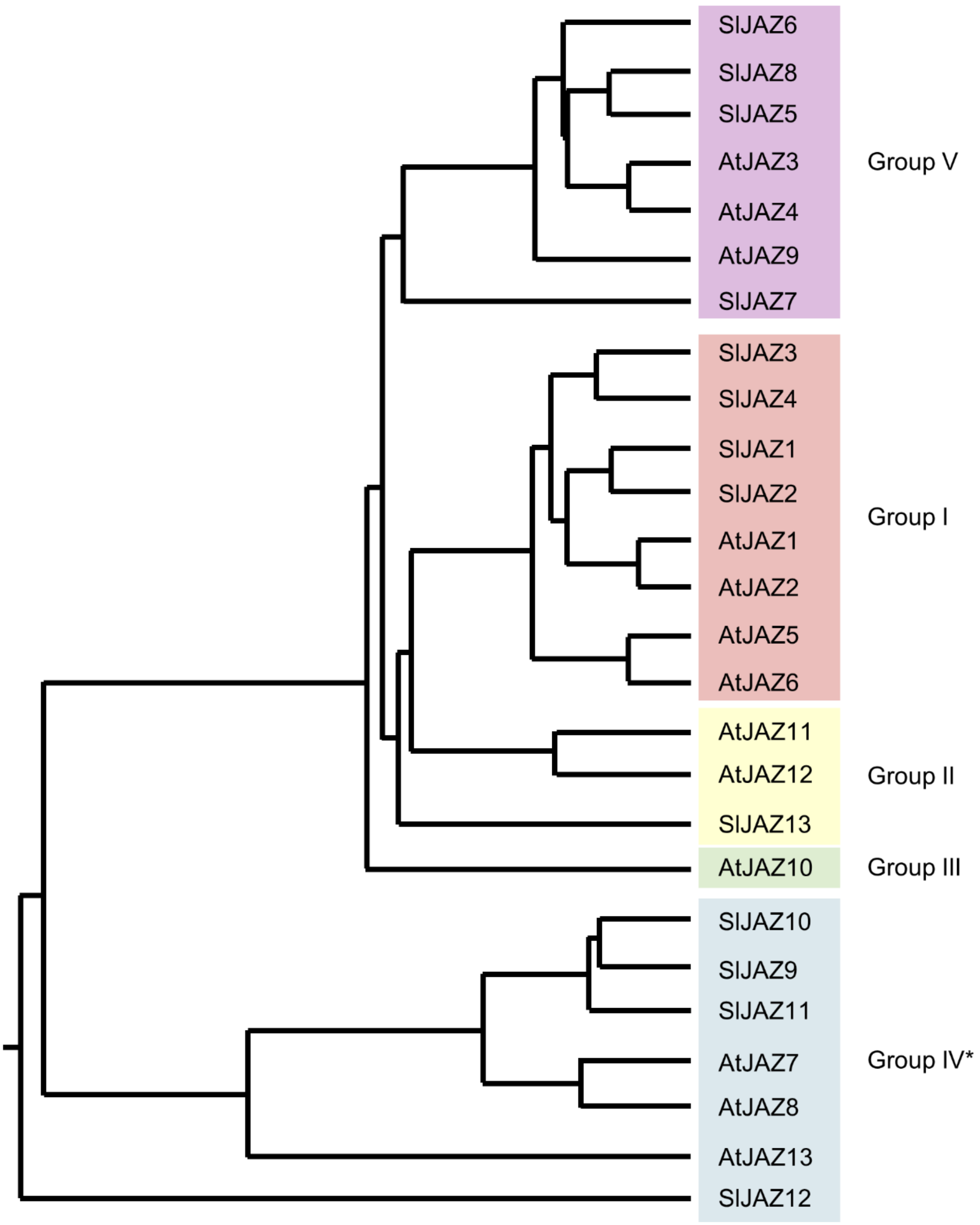

Supplementary Fig. 3. Phylogenetic tree of *At*JAZ and *Sl*JAZ. Asterisk: JAZs classified into Group IV are non-canonical JAZs that lack the short conserved canonical degron sequence. The phylogenetic analysis aligned the full-length protein sequences with Geneious (Tomy Digital Biology Co., Ltd.). The Pam250 program was used for

sequence alignment, and the Neighbor-Joining.[13] program was used for drawing the results trees.

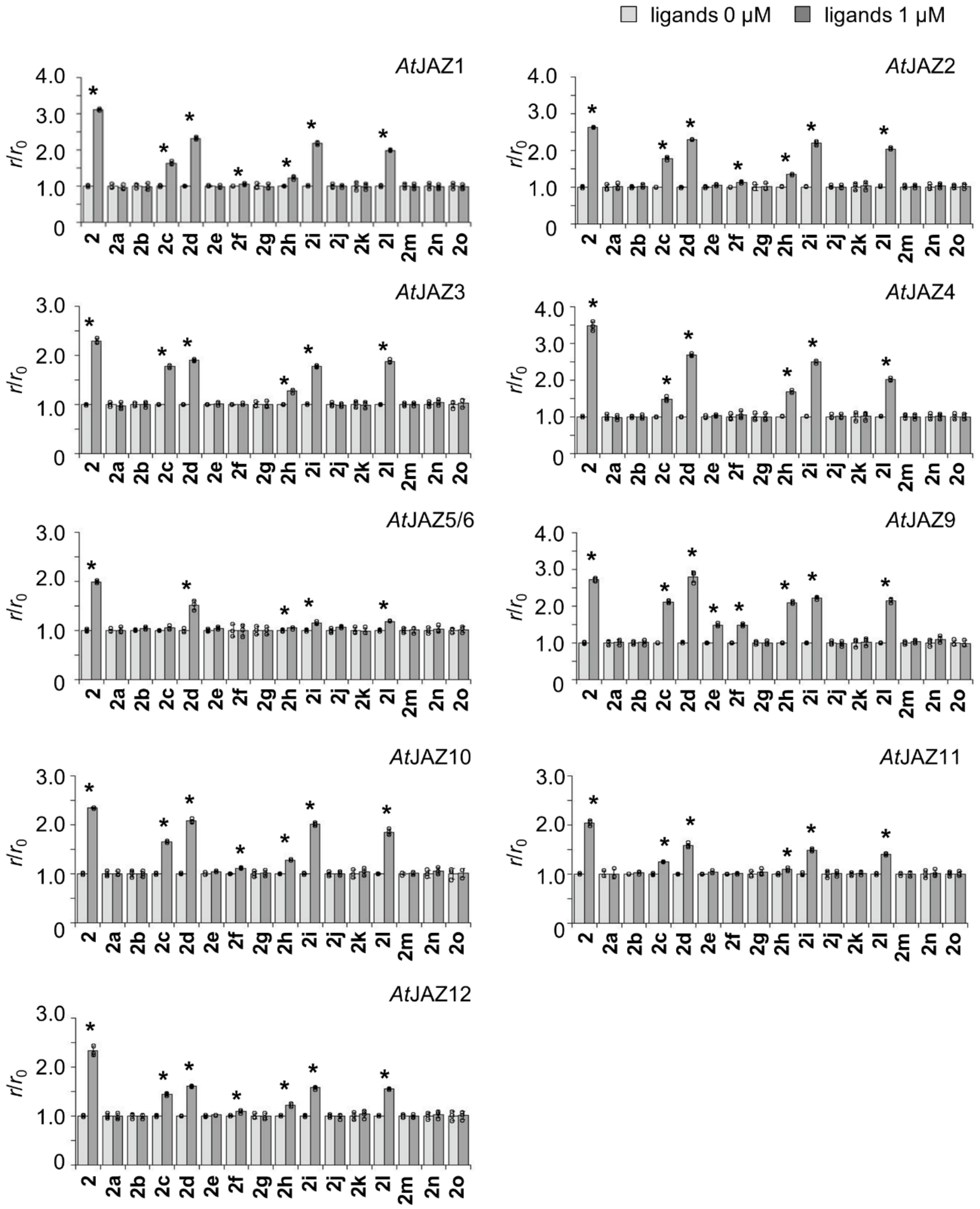

Supplementary Fig. 4. FA change (r/r_0) of Fl-*At*JAZPs (100 nM) with GST-*At*COI1 (100 nM) in the absence or presence of each stereoisomer (1 μM). Values are mean ± SD of three replicates. Significant differences between FA values in the absence or presence of the indicated ligands (shown as asterisk) were evaluated by a Student's t-test ($p < 0.05$).

*At*JAZ (nM)	**2**	**2a**	**2b**	**2c**	**2d**	**2e**	**2f**	**2g**	**2h**	**2i**	**2j**	**2k**	**2l**	**2m**	**2n**	**2o**
JAZ1	6.7	n.d.	n.d.	975.8	11.6	n.d.	19097	n.d.	3813	139.5	n.d.	n.d.	285.0	n.d.	n.d.	n.d.
JAZ2	0.9	n.d.	n.d.	524.1	13.0	n.d.	6718	n.d.	1878	64.8	n.d.	n.d.	142.4	n.d.	n.d.	n.d.
JAZ3	1.4	n.d.	n.d.	172.2	10.4	n.d.	n.d.	n.d.	2071	85.0	n.d.	n.d.	31.7	n.d.	n.d.	n.d.
JAZ4	9.0	n.d.	n.d.	2758	19.2	n.d.	n.d.	n.d.	1369	203.0	n.d.	n.d.	623.0	n.d.	n.d.	n.d.
JAZ5/6	21.3	n.d.	n.d.	n.d.	78.6	n.d.	n.d.	n.d.	4764	1196	n.d.	n.d.	499.9	n.d.	n.d.	n.d.
JAZ9	0.3	n.d.	n.d.	59.6	2.8	1222	1242	n.d.	200.7	3.4	n.d.	n.d.	73.0	n.d.	n.d.	n.d.
JAZ10	2.4	n.d.	n.d.	538.0	12.5	n.d.	17849	n.d.	2515	60.8	n.d.	n.d.	161.4	n.d.	n.d.	n.d.
JAZ11	5.9	n.d.	n.d.	1066	11.4	n.d.	n.d.	n.d.	4881	102.9	n.d.	n.d.	375.5	n.d.	n.d.	n.d.
JAZ12	9.1	n.d.	n.d.	308.4	19.7	n.d.	4912	n.d.	1570	35.7	n.d.	n.d.	83.2	n.d.	n.d.	n.d.

Supplementary Fig. 5. The K_d values (nM) of each stereoisomer for possible *At*COI1-*At*JAZ co-receptor. "n.d." means that the K_d value could not be determined from the obtained dose-response curve.

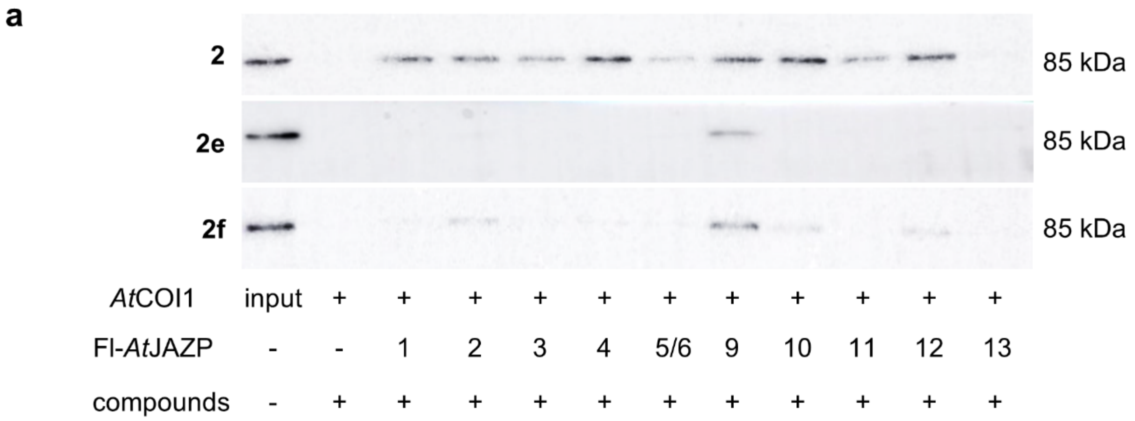

Supplementary Fig. 6. Pull-down assay of purified GST-*At*COI1 (5 nM) with Fl-*At*JAZPs (10 nM) in the presence of 5 µM of 2, 2e, and 2f. (**a**) Cropped and (**b** to **d**) uncropped images of pull-down assay. HRP-conjugated anti-GST antibody was used to detect GST-COI1 (1/5000 dilution). COI1-**2f**-JAZ1 pair was not detected in the pull-down assay because the affinity of **2f** against COI1-JAZ1 pairs was considerably weak (ca. 20,000 nM; shown in Supplementary Fig. 5), and washing procedures removed the weak interaction. (**b**), (**c**), and (**d**) are obtained by three independent experiments.

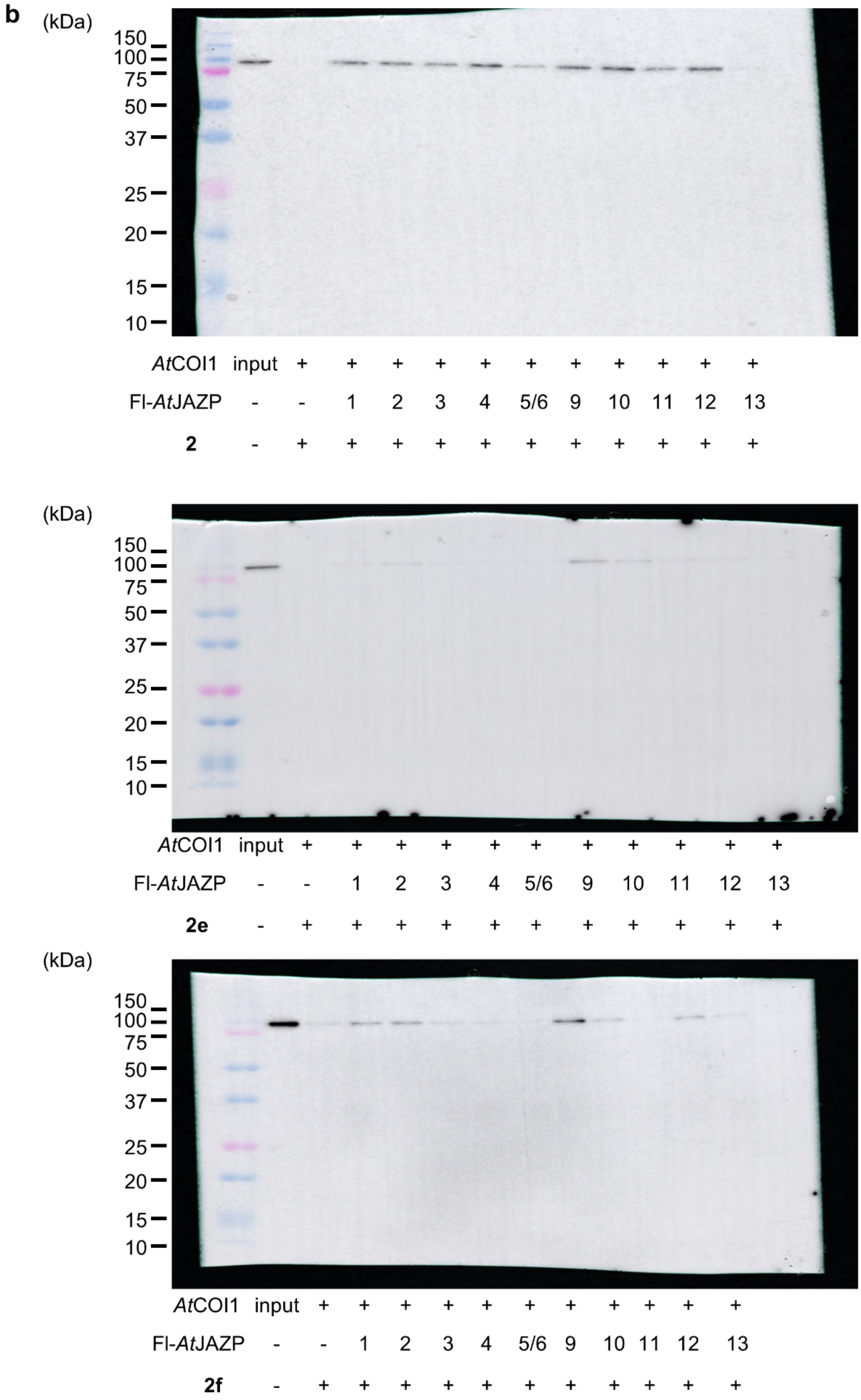

Supplementary Fig. 6. (continued) (**b**) uncropped images of pull-down assay.

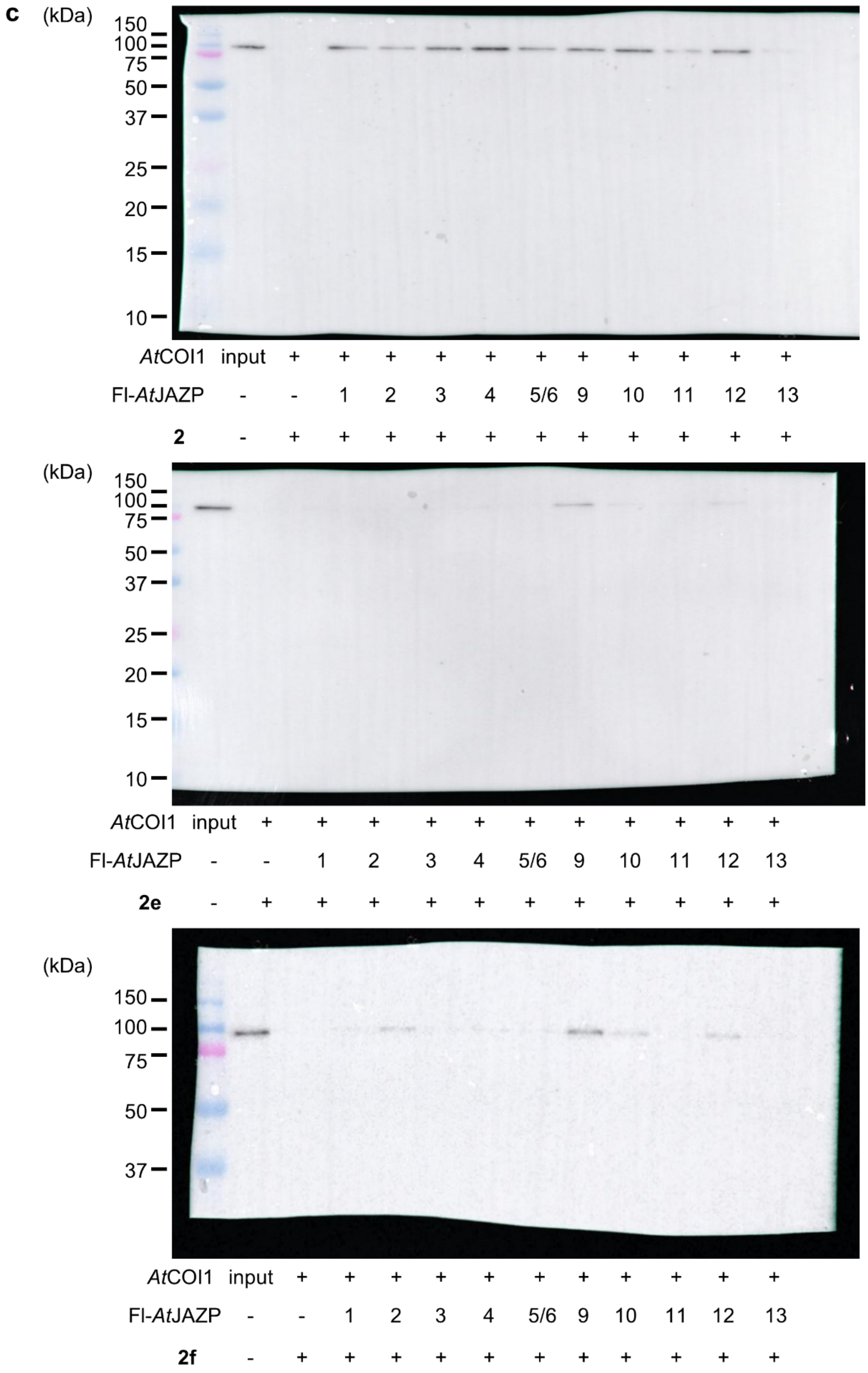

Supplementary Fig. 6. (continued) (**c**) uncropped images of pull-down assay.

d

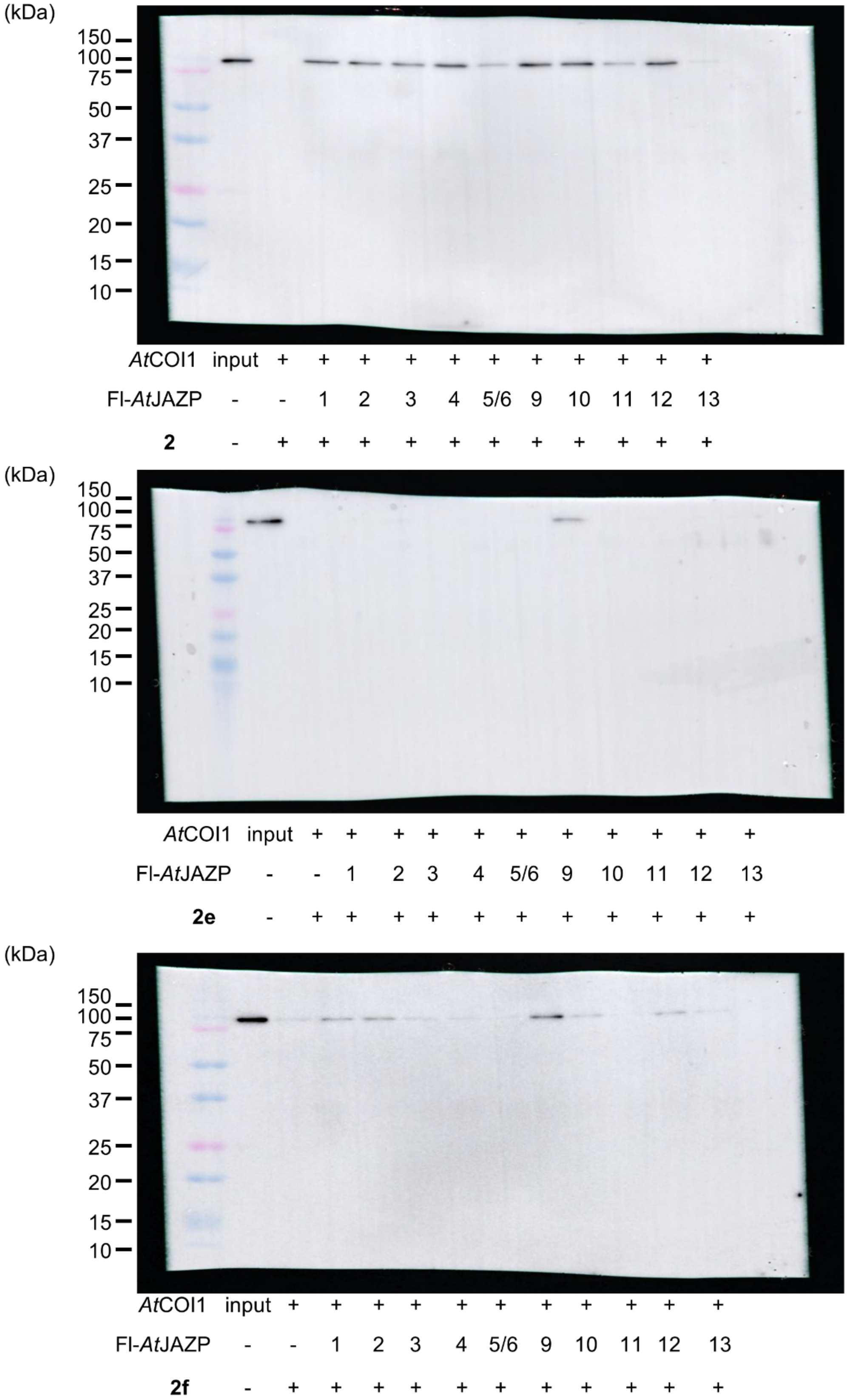

Supplementary Fig. 6. (continued) (**d**) uncropped images of pull-down assay.

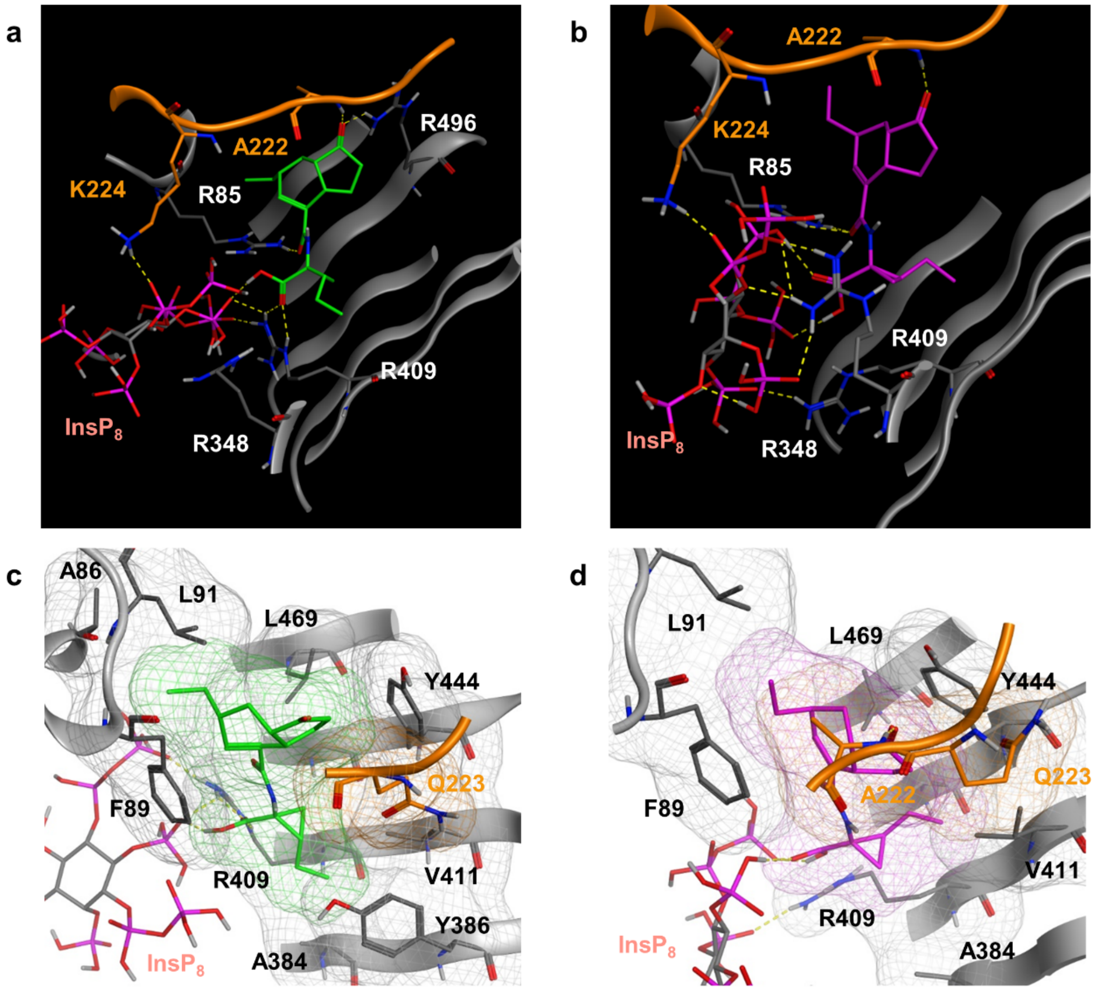

e

	2	2e
	Hydrogen bond	
O_amide	R85^{COI1}	R85^{COI1}
O_ketone	R496^{COI1},A222^{JAZ9}	A222^{JAZ9}
O_COOH	R85^{COI1},R409^{COI1},PO$_4$	R85^{COI1},R409^{COI1},PO$_4$
	Hydrophobic interaction	
CFA moiety	A86^{COI1},F89^{COI1},L91^{COI1} Y444^{COI1},L469^{COI1},Q221^{JAZ9}	F89^{COI1},L91^{COI1},Y444^{COI1} L469^{COI1},Q221^{JAZ9},A222^{JAZ9}
CMA moiety	A384^{COI1},Y386^{COI1} R409^{COI1},V411^{COI1},A443^{COI1}	A384^{COI1},R409^{COI1},V411^{COI1},A443^{COI1}

Supplementary Fig. 7. *In silico* analysis of 2 and 2e in COI1-InsP$_8$-JAZ9 complex. To obtain the binding model of **2** and **2e** with *At*COI1-InsP8-*At*JAZ9, we performed docking and subsequent *in silico* molecular dynamics (MD) simulations of (**a**, **c**) *At*COI1 (gray)-**2** (green)-*At*JAZ9 (orange) and (**b**, **d**) *At*COI1-**2e** (magenta)-*At*JAZ9 complex according to previous study.[4, 14, 15] As shown in Supplementary Fig. 7 **a-d**, **2e** and **2** can be accommodated in the binding pocket of *At*COI1-*At*JAZ9. Pink-colored molecule is

PO4 in ligand binding pocket. (**e**) Hydrogen bonds (yellow dotted lines) between *At*COI1-*At*JAZ9 and **2e** are well conserved. In contrast, hydrophobic interaction by Y386 in *At*COI1-**2**-*At*JAZ9 was lost in *At*COI1-**2e**-*At*JAZ9, explaining the weaker affinity of **2e** (K_d 1.2 μM vs. K_d 0.3 nM of **2**) with *At*COI1-*At*JAZ9. Hydrogen bonds were determined by average distances (at least 3.5 Å) calculated with radial distribution function (RDF) analyses between the atoms in each ligand and nitrogen and oxygen atoms in *At*COI1, *At*JAZ9, and PO4.

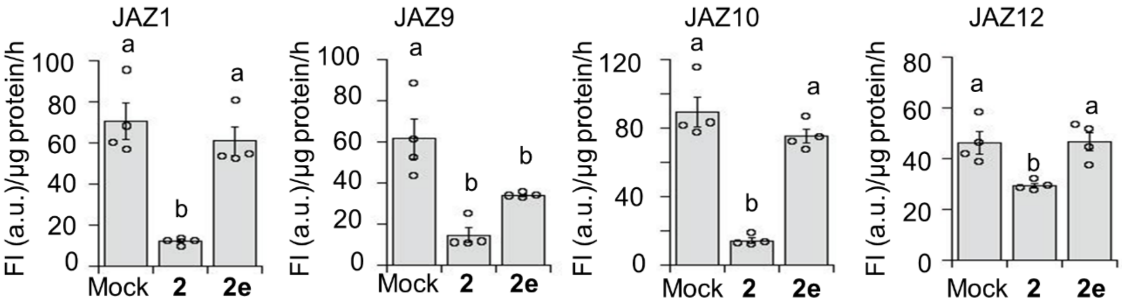

Supplementary Fig. 8. GUS quantification assay of compounds-treated transgenic plants. Evaluation of GUS activity in the roots of 4-day-old *35S: JAZ1-GUS*, *35S: JAZ9-GUS*, *35S: JAZ10-GUS*, and *35S: JAZ12-GUS* seedlings (n=4). Quantification of GUS activity was assessed by fluorescence of hydrolyzed methylumbelliferyl-β-D-glucuronide (Ex. / Em. = 365 / 460 nm). Similar results were obtained in three independent experiments. Significant differences were evaluated by one-way ANOVA/Tukey HSD post hoc test ($p < 0.05$). Values are mean ± SE.

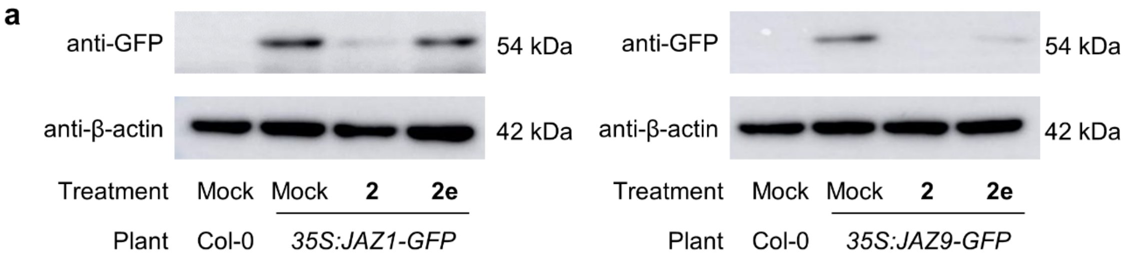

Supplementary Fig. 9. *In vivo* degradation assay using *35S: JAZ1/9-GFP* lines. (**a**) Cropped and (**b** to **d**) uncropped images of *in vivo* degradation assay. 4-day-old seedlings were treated with 1 μM of **2** and **2e** for 2 h. HRP-conjugated anti-GFP antibody was used to detect JAZ1/9-GFP (1/5,000 dilution). Anti-β-actin antibody and HRP-conjugated anti-mouse IgM antibody were used to detect β-actin (1/1,000 and 1/20,000 dilution, respectively). (**b**), (**c**), and (**d**) are obtained by three independent experiments. *: JAZ1- or JAZ9-GFP, **: unknown protein, ***: β-actin monomer.

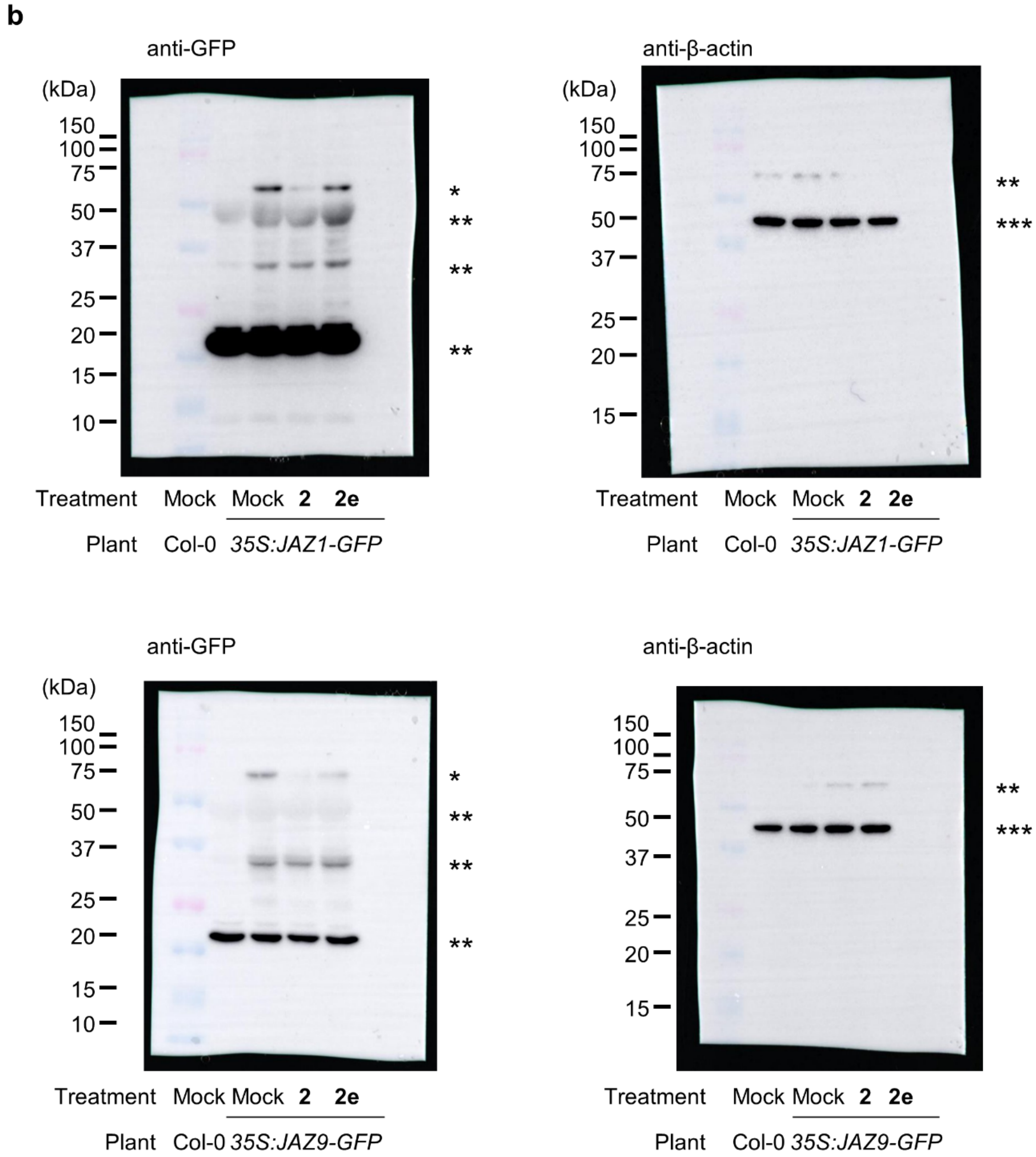

Supplementary Fig. 9. (continued) (**b**) uncropped images of *in vivo* degradation assay.

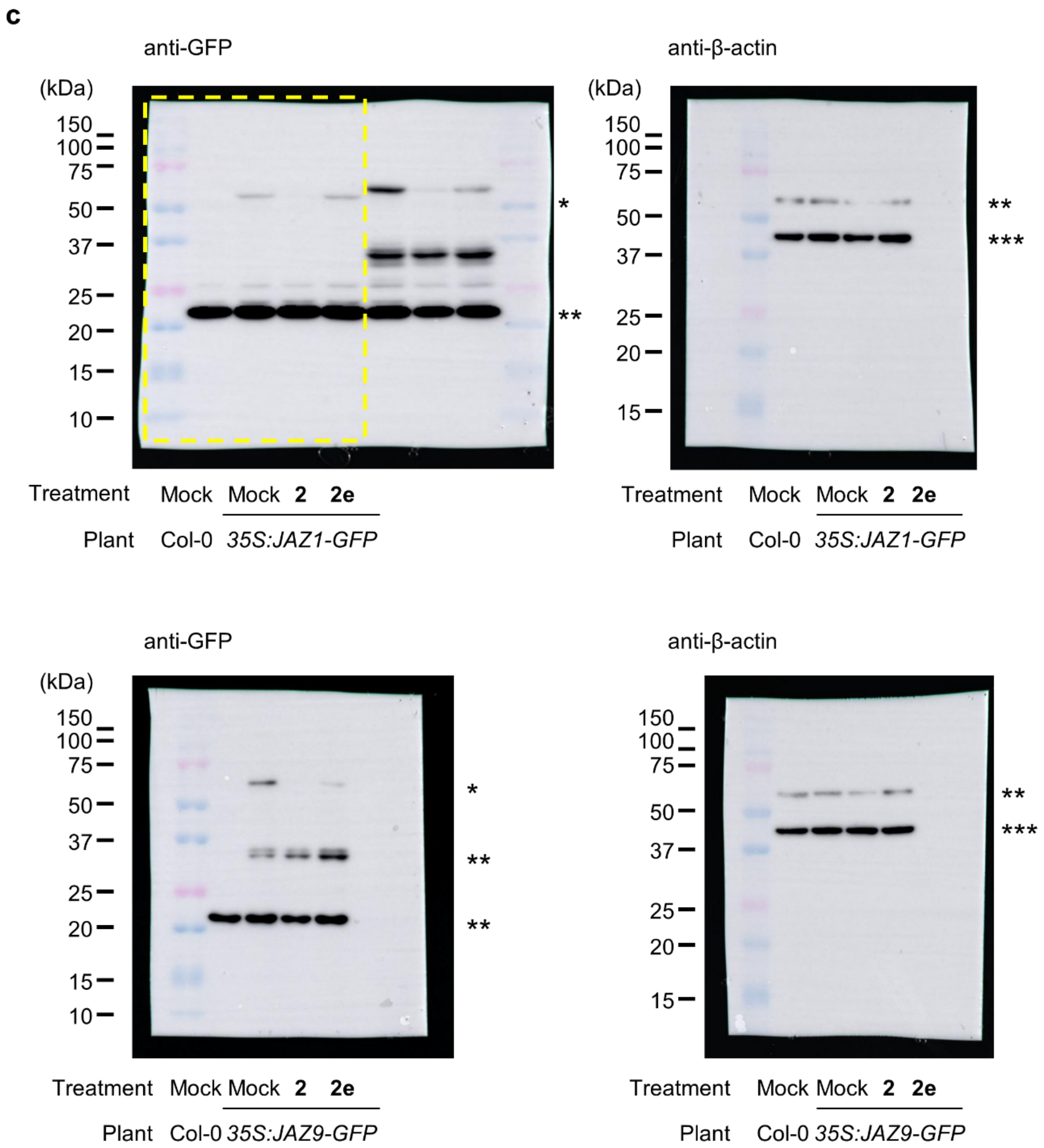

Supplementary Fig. 9. (continued) (**c**) uncropped images of *in vivo* degradation assay.

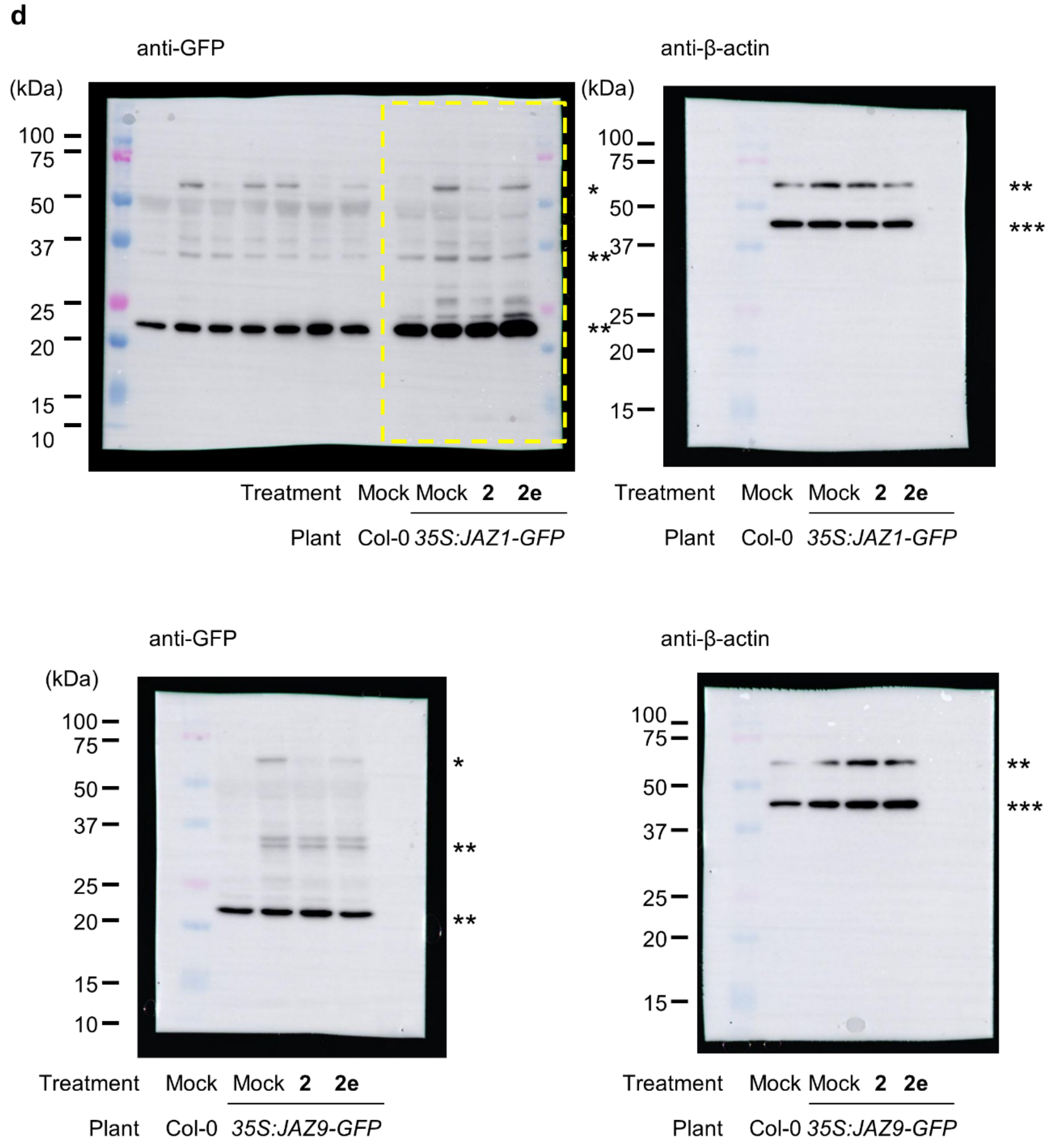

Supplementary Fig. 9. (continued) (**d**) uncropped images of *in vivo* degradation assay.

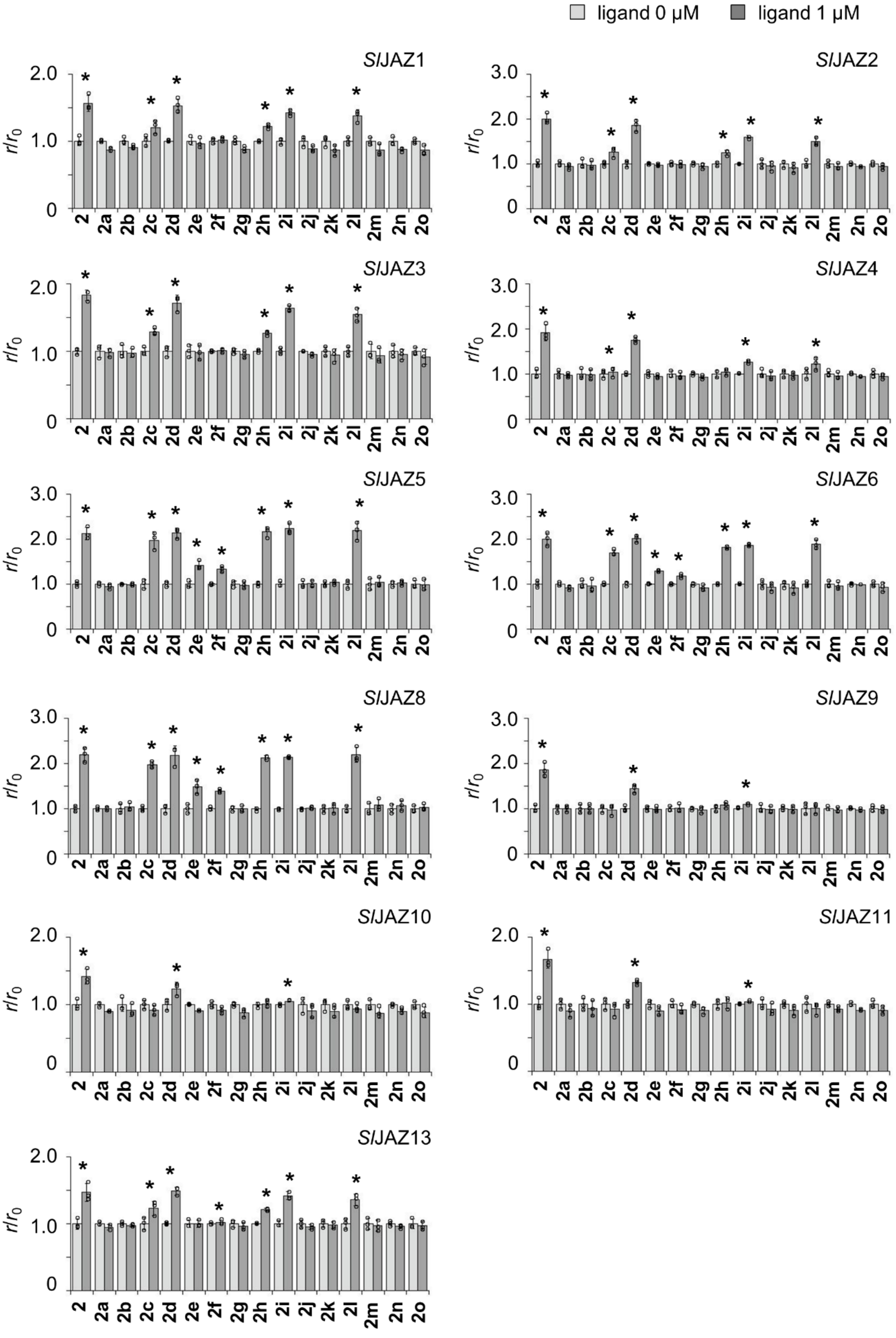

ligand 0 μM
ligand 1 μM
SlJAZ1
SlJAZ2
SlJAZ3
SlJAZ4
SlJAZ5
SlJAZ6
SlJAZ8
SlJAZ9
SlJAZ10
SlJAZ11
SlJAZ13
r/r0
2 2a 2b 2c 2d 2e 2f 2g 2h 2i 2j 2k 2l 2m 2n 2o

Supplementary Fig. 10. FA change (r/r_0) of Fl-*Sl*JAZPs (100 nM) with GST-*Sl*COI1 (100 nM) in the absence or presence of each stereoisomer (1 μM). Values are mean ± SD of three replicates. Significant differences between FA values in the absence or presence of the indicated ligands (shown as asterisk) were evaluated by a Student's t-test ($p < 0.05$).

*Sl*JAZ (nM)	**2**	**2a**	**2b**	**2c**	**2d**	**2e**	**2f**	**2g**	**2h**	**2i**	**2j**	**2k**	**2l**	**2m**	**2n**	**2o**
JAZ1	1.5	n.d.	n.d.	769.0	39.2	n.d.	n.d.	n.d.	663.7	79.0	n.d.	n.d.	162.6	n.d.	n.d.	n.d.
JAZ2	2.0	n.d.	n.d.	1228	174.3	n.d.	n.d.	n.d.	1282	268.7	n.d.	n.d.	513.1	n.d.	n.d.	n.d.
JAZ3	2.4	n.d.	n.d.	1445	147.2	n.d.	n.d.	n.d.	1114	203.6	n.d.	n.d.	368.7	n.d.	n.d.	n.d.
JAZ4	18.1	n.d.	n.d.	16178	83.5	n.d.	n.d.	n.d.	n.d.	1226	n.d.	n.d.	1837	n.d.	n.d.	n.d.
JAZ5	0.1	n.d.	n.d.	94.6	40.0	1564	1888	n.d.	99.3	14.5	n.d.	n.d.	26.9	n.d.	n.d.	n.d.
JAZ6	0.3	n.d.	n.d.	75.0	28.2	2390	4213	n.d.	130.0	12.0	n.d.	n.d.	33.9	n.d.	n.d.	n.d.
JAZ8	2.3	n.d.	n.d.	100.5	18.3	1353	1855	n.d.	92.3	10.5	n.d.	n.d.	21.5	n.d.	n.d.	n.d.
JAZ9	42.0	n.d.	n.d.	n.d.	532.6	n.d.	n.d.	n.d.	n.d.	4572	n.d.	n.d.	n.d.	n.d.	n.d.	n.d.
JAZ10	27.8	n.d.	n.d.	n.d.	391.6	n.d.	n.d.	n.d.	n.d.	13431	n.d.	n.d.	n.d.	n.d.	n.d.	n.d.
JAZ11	42.3	n.d.	n.d.	n.d.	620.2	n.d.	n.d.	n.d.	n.d.	39643	n.d.	n.d.	n.d.	n.d.	n.d.	n.d.
JAZ13	1.9	n.d.	n.d.	906.4	125.7	n.d.	40531	n.d.	904.7	111.3	n.d.	n.d.	283.5	n.d.	n.d.	n.d.

Supplementary Fig. 11. The K_d values (nM) of each stereoisomer for possible *Sl*COI1-*Sl*JAZ co-receptor. "n.d." means that the K_d value could not be determined from the obtained dose-response curve.

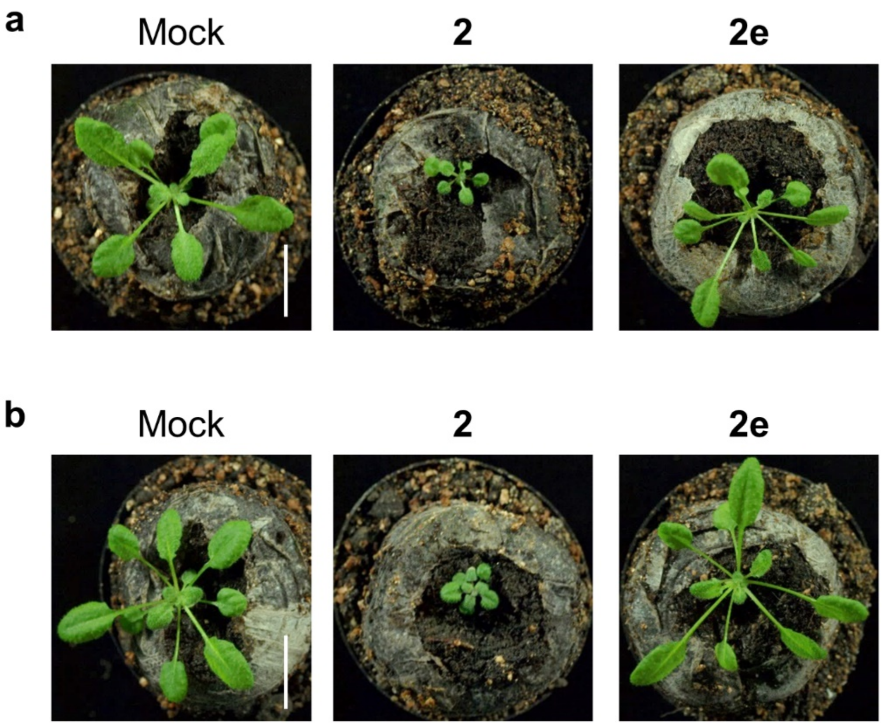

Supplementary Fig. 12. Growth inhibition assay using ligand-treated 4-week-old plants. Photographs in (**a**), (**b**), and Fig. 3a are obtained by three independent experiments. Scale bar: 1.0 cm.

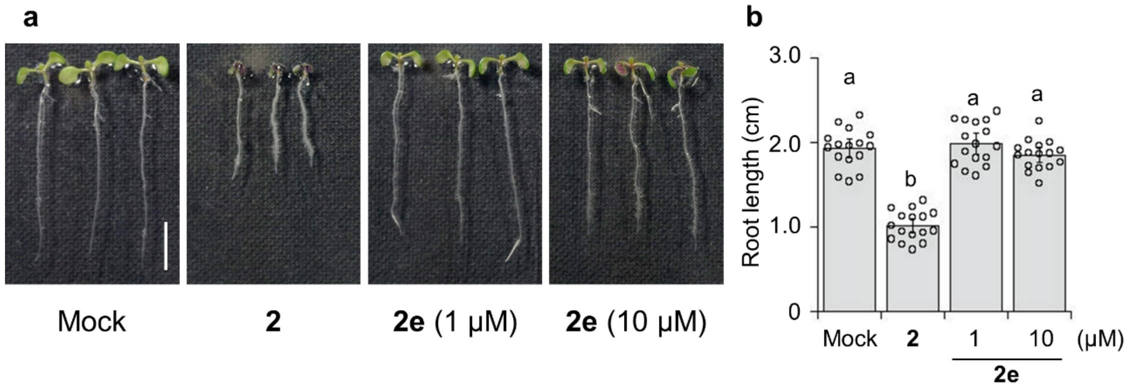

Supplementary Fig. 13. Growth inhibition assay using ligand-treated seedlings. (**a**) Images of 6-day-old **2** (1 μM)- or **2e** (1 or 10 μM)-treated seedlings. (**b**) Quantification of root length of the ligand-treated seedlings (n=8). Significant differences were evaluated by one-way ANOVA/Tukey HSD post hoc test ($p < 0.05$). Values are mean ± SE. Scale bar: 0.5 cm.

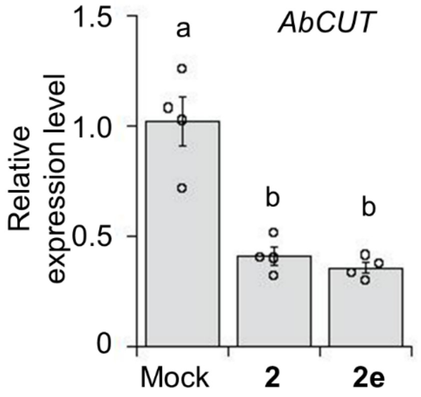

Supplementary Fig. 14. Quantification of fungal growth by RT-qPCR analysis of *A. brassicicola* gene (*AbCUT*) in compound-treated plants. ~~RT-qPCR~~Quantitative PCR analysis was performed by using *A. brassicicola* genomic DNA extracted from ~~bacteria~~fungus-infected leaves shown in Fig. 3c. *AbACT8* was used as a reference gene. Significant differences were evaluated by one-way ANOVA/Tukey HSD post hoc test ($p < 0.05$). Values are mean ± SE.

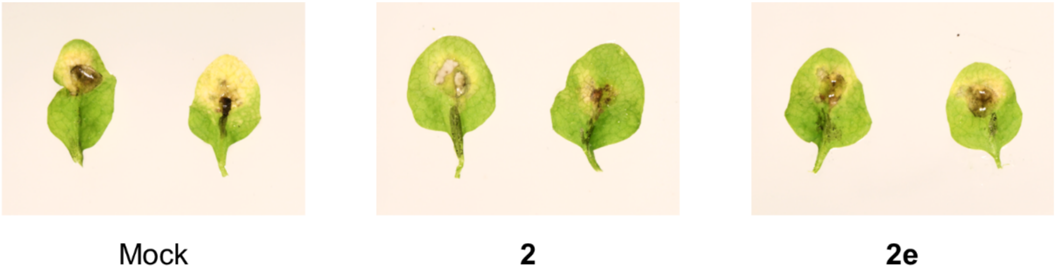

Supplementary Fig. 15. Representative images of *jaz9-1* leaves infected with *A. brassicicola*. Box plots of quantification of fungal spores are shown in Fig. 3e.

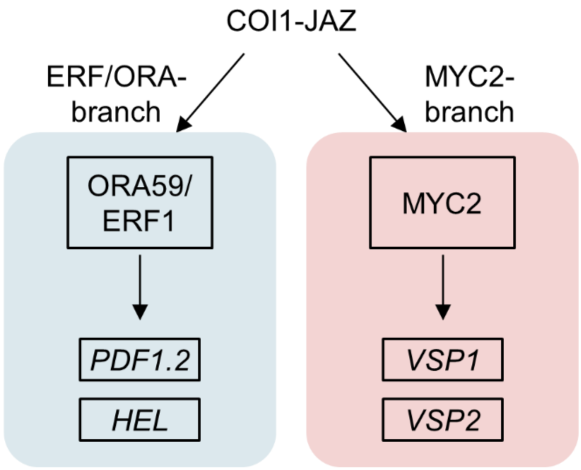

Supplementary Fig. 16. Two signaling branches of downstream JA signaling. *ORA59, ERF1*, *PDF1.2,* and *HEL* belong to the ERF/ORA-branch, and *MYC2* and *VSP1/2* belong to the MYC2-branch.

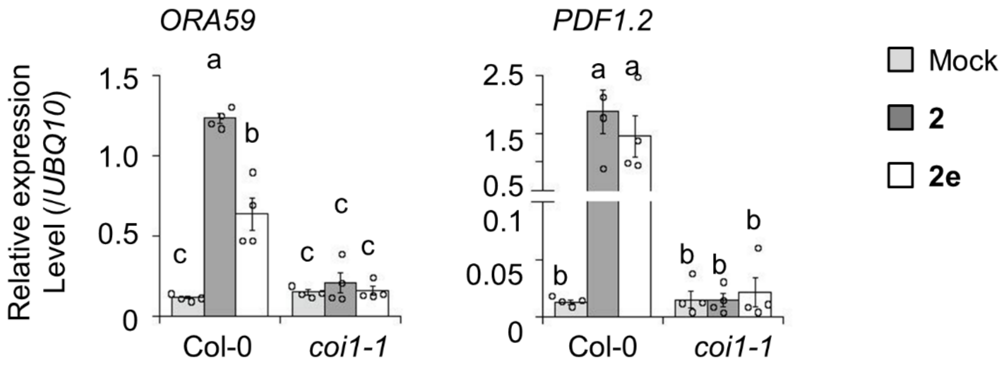

Supplementary Fig. 17. COI1-dependent Mode-of-action of 2e. Gene expression analyses of marker genes belonging to the ERF/ORA branch by RT-qPCR in Col-0 and *coi1-1* seedling (n=4) with or without compound treatment (1 μM) for 2 h (*ORA59*) or 8 h (*PDF1.2*). Similar results were obtained in three independent experiments. Significant differences were evaluated by one-way ANOVA/Tukey HSD post hoc test ($p < 0.05$). Values are mean ± SE.

AT1G03940	TIFY7	JAZ7	HMT3	RAP2.9	PLP5	UMAMIT42
AT1G05575	JAZ6	AT2G34810	AT3G23550	TPS13	AT4G37409	AOS
JAZ1	AT1G73325	AT2G34930	AOC1	RTFL12	AT5G02170	AT5G42930
CLH1	MKK9	ANNAT4	AOC2	ATCSLA15	AT5G02940	AT5G43570
OPCL1	AT1G76590	COR15A	AIG2	BAM5	PAI2	PDF1.2
AT1G23850	AT2G18210	WRKY45	NIT2	CYP705A1	AT5G05600	PDF1.2c
CSLA10	PDF1.3	GSTF11	LOX2	HPL1	PGIP2	AT5G47980
STZ	PDF1.2b	AT3G10930	LSU3	ERF-1	AT5G13210	CYP96A4
JAZ8	AT2G26370	SCPL15	DIN11	NRT1.8	KIN1	UGT76E1
MYC2	AR781	AT3G14260	AT3G55310	AT4G21903	AT5G19100	UGT76E2
AT1G59950	CYP94C1	AT3G15356	JRG21	AT4G22610	AT5G19110	AT5G62360
DIR5	GSTU4	NAC3	AT3G56200	CORI3	SQP2	CYP94B1
ENDO2	CIPK13	AT3G22275	AT3G59340	AT4G24350	CYP81D1	AT5G66650

Supplementary Fig. 18. List of 91 genes upregulated by 2e. List of up-regulated 91 genes in intersection of **2**- or **2e**-treated Col-0, and not in mock-treated *jaz9-1,* in Venn diagram shown in Fig. 4b.

a

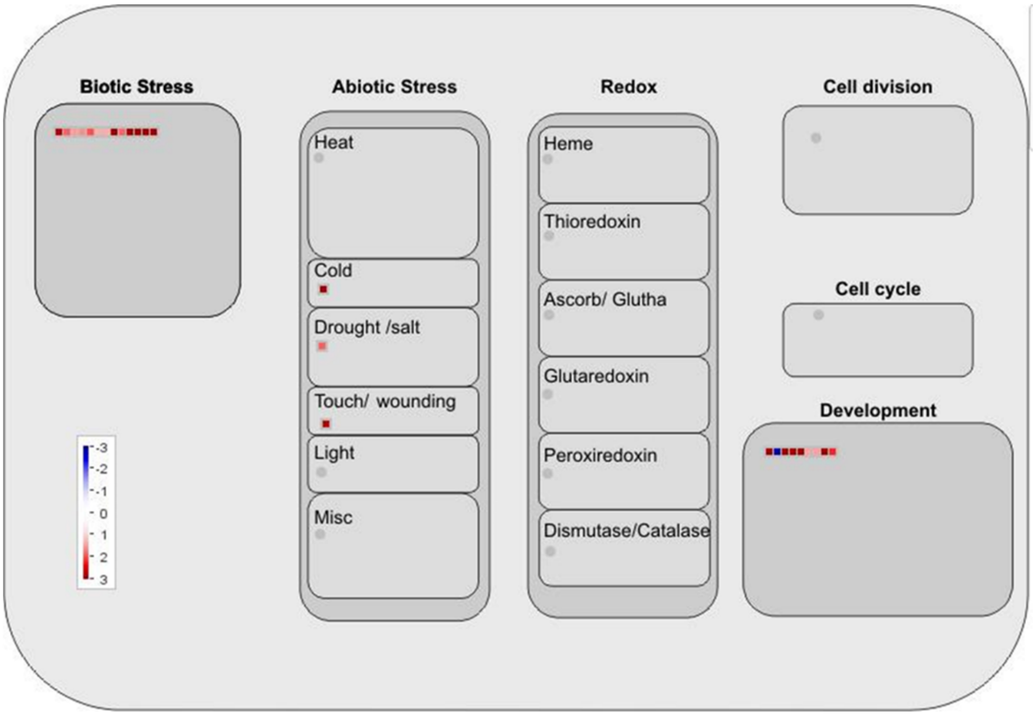

b

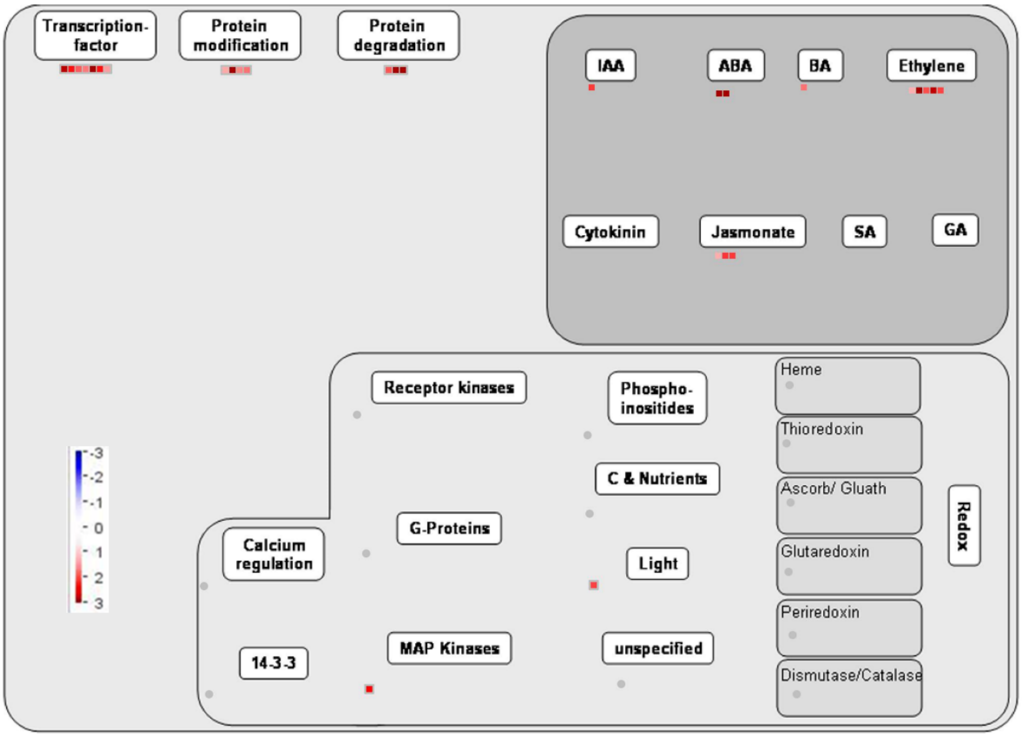

Supplementary Fig. 19. Overview of RNAseq. Changes in response to treatment with **2e**. (**a**) Cellular response, (**b**) regulation overview. Expression data are represented as log_2 fold changes compared to mock-treated seedlings. Little gene downregulations were observed in the **2e**-treated seedlings (**a**–**b**). Expression data are represented as log_2 fold changes compared to mock-treated seedlings.

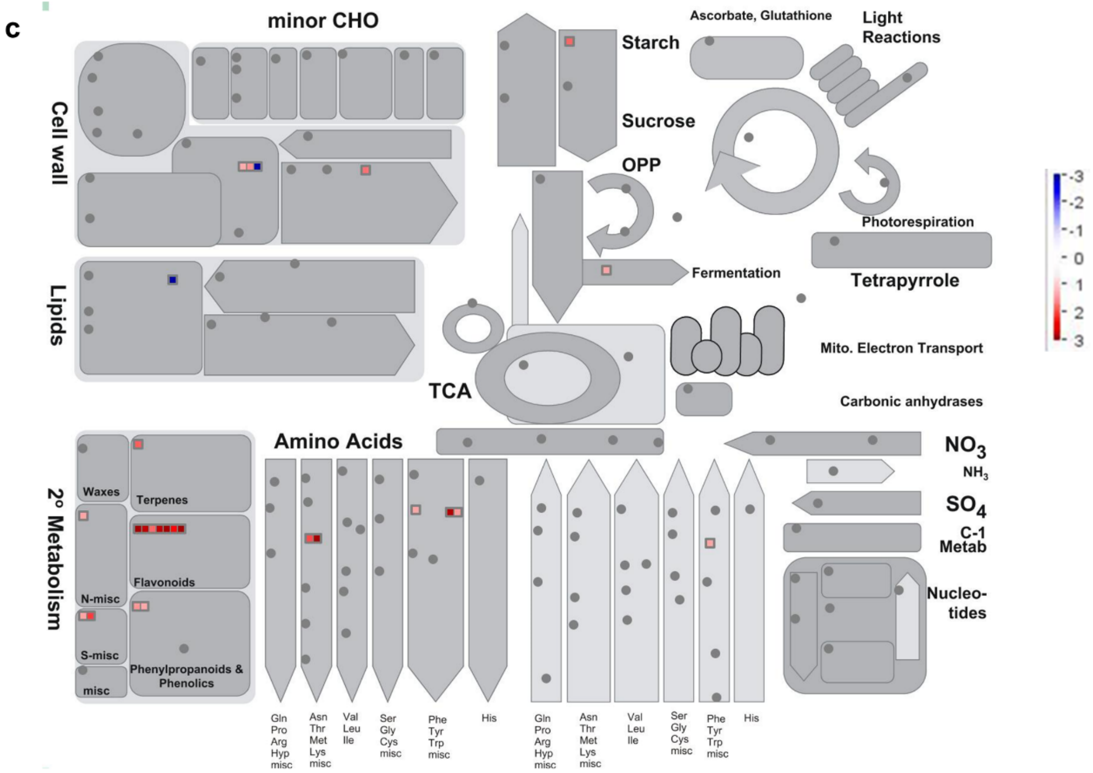

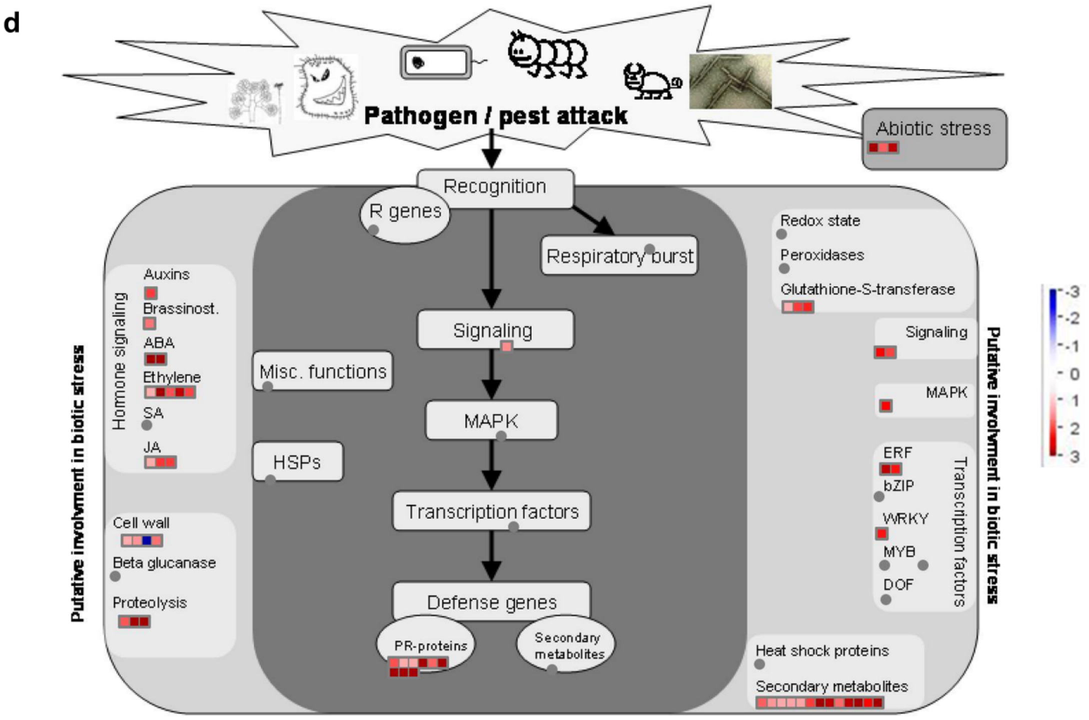

Supplementary Fig. 19. (continued) Changes in response to treatment with **2e**. (**c**) Metabolism overview, (**d**) biotic stress. Expression data are represented as $\log_2$ fold changes compared to mock-treated seedlings. Little gene downregulations were observed in the **2e**-treated seedlings (**c**–**d**). Expression data are represented as $\log_2$ fold changes compared to mock-treated seedlings.

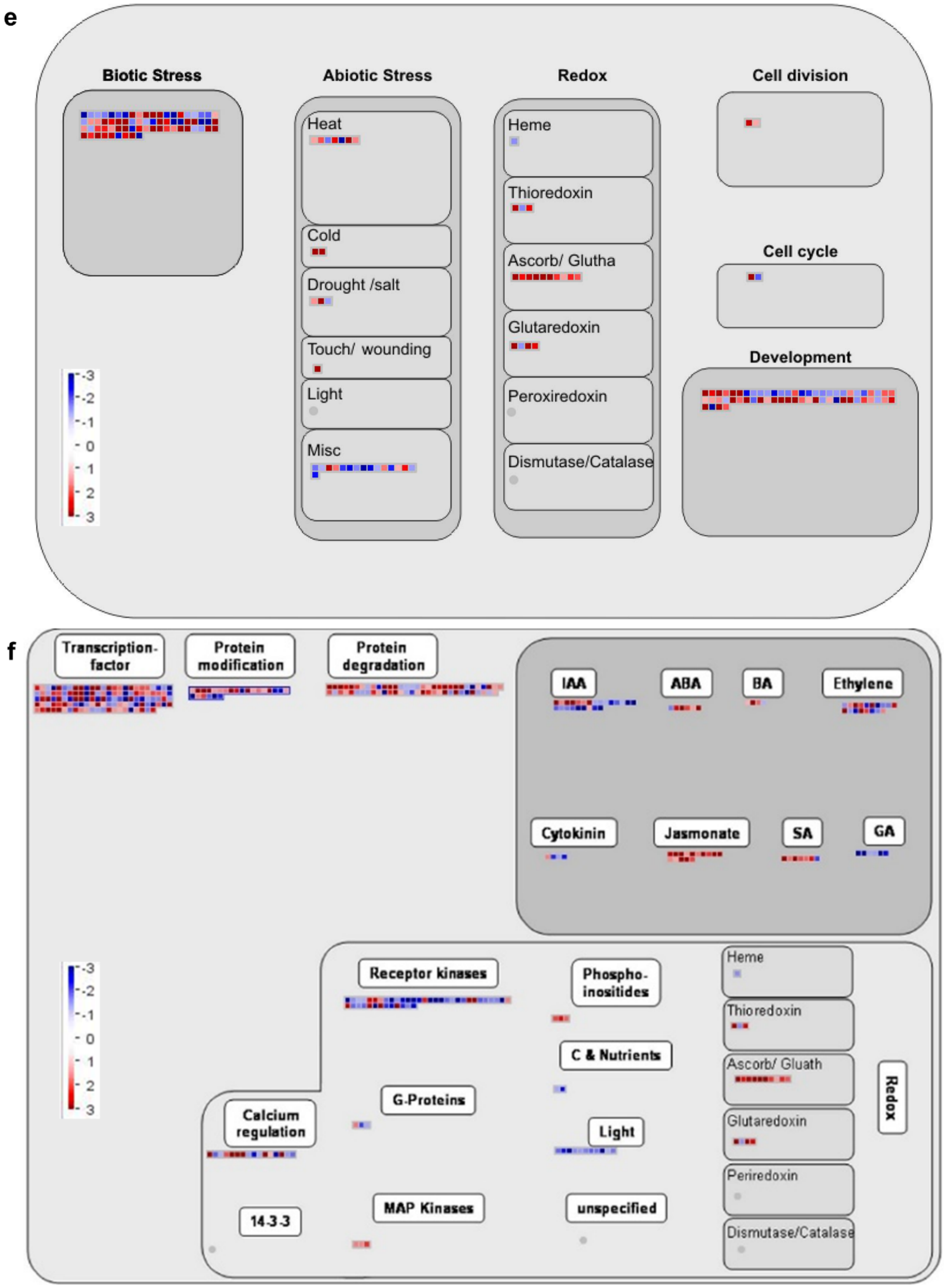

Supplementary Fig. 19. (continued) Changes in metabolism-related genes in response to treatment with **2**. (**e**) Cellular response, (**f**) regulation overview.

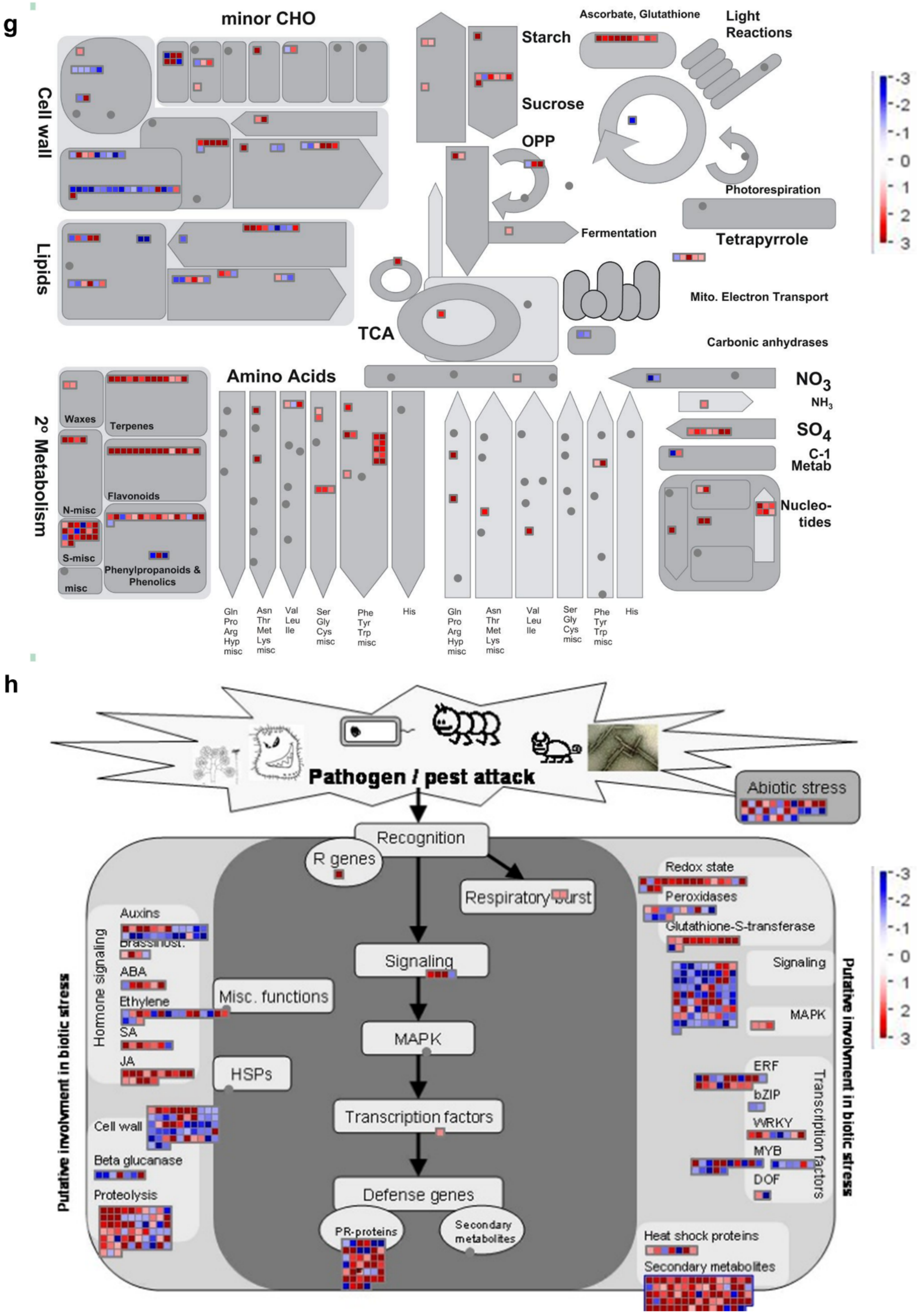

Supplementary Fig. 19. (continued) Changes in metabolism-related genes in response to treatment with **2**. (**g**) Metabolism overview, (**h**) biotic stress.

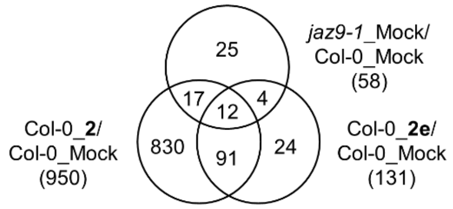

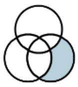

GO biological process complete	*P*-value
cellular response to ethylene stimulus (GO:0071369)	1.89E-06
response to ethylene (GO:0009723)	1.90E-06
response to chemical (GO:0042221)	2.36E-05
ethylene-activated signaling pathway (GO:0009873)	1.63E-04
cellular response to chemical stimulus (GO:0070887)	5.31E-04
phosphorelay signal transduction system (GO:0000160)	7.87E-04
response to organic substance (GO:0010033)	1.19E-03
response to stress (GO:0006950)	1.70E-03
response to chitin (GO:0010200)	5.61E-03
response to stimulus (GO:0050896)	7.89E-03
response to external biotic stimulus (GO:0043207)	1.00E-02
response to other organism (GO:0051707)	1.00E-02
response to biotic stimulus (GO:0009607)	1.01E-02
biological process involved in interspecies interaction between organisms (GO:0044419)	1.17E-02
response to jasmonic acid (GO:0009753)	1.41E-02
response to fatty acid (GO:0070542)	1.54E-02
response to hormone (GO:0009725)	2.34E-02
response to endogenous stimulus (GO:0009719)	2.73E-02
cellular response to hormone stimulus (GO:0032870)	3.80E-02

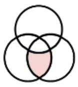

GO biological process complete (ID)	*P*-value
response to wounding (GO:0009611)	1.55E-21
response to jasmonic acid (GO:0009753)	6.13E-12
response to fatty acid (GO:0070542)	8.22E-12
response to stress (GO:0006950)	3.67E-11
response to stimulus (GO:0050896)	3.77E-11
regulation of jasmonic acid mediated signaling pathway (GO:2000022)	1.43E-08
response to chemical (GO:0042221)	4.58E-08
response to lipid (GO:0033993)	3.72E-07
response to oxygen-containing compound (GO:1901700)	2.34E-05
response to hormone (GO:0009725)	7.11E-05
jasmonic acid metabolic process (GO:0009694)	8.04E-05
regulation of defense response (GO:0031347)	9.00E-05
response to endogenous stimulus (GO:0009719)	1.03E-04
jasmonic acid biosynthetic process (GO:0009695)	1.35E-04
regulation of response to stress (GO:0080134)	3.43E-04
response to toxic substance (GO:0009636)	5.40E-04
regulation of defense response to insect (GO:2000068)	5.64E-04
response to external biotic stimulus (GO:0043207)	2.22E-03
response to other organism (GO:0051707)	2.22E-03
response to biotic stimulus (GO:0009607)	2.27E-03
biological process involved in interspecies interaction between organisms (GO:0044419)	2.58E-03
response to organic substance (GO:0010033)	4.98E-03
cellular response to jasmonic acid stimulus (GO:0071395)	2.00E-02
cellular response to fatty acid (GO:0071398)	2.54E-02
response to external stimulus (GO:0009605)	3.44E-02
detoxification (GO:0098754)	4.45E-02

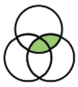

No enrichment

Supplementary Fig. 20. Venn diagram and GO enrichment analysis of up-regulated genes at least 2.5-fold change (FDR<0.1) in response to different treatments. The Gene Ontology (GO) analysis of these 91 genes showed that **2e** induced the jasmonate signaling (ID: 0009611, 0009753, and 0043207), including defense responses (ID: 0031347, and 0051707). Furthermore, 16 genes overlapped **2e**-treated Col-0, and mock-treated *jaz9-1* represented no enrichment of GO terms.

a

AT1G03940	MYC2	CYP94C1	HMT3	TPS13	AT4G24350	CYP81D1
ORA59	DIR5	JAZ7	AT3G23550	RTFL12	SEN1	AOS
AT1G15125	AT1G73325	AT2G34810	AOC1	BAM5	AT5G02940	AT5G43570
JAZ1	MKK9	ANNAT4	AOC2	HPL1	AT5G05600	PDF1.2
CLH1	PDF1.3	AT2G43590	LOX2	ERF1	PGIP2	CYP96A4
CSLA10	AT2G26370	AT3G15356	LSU3	AT4G21903	AT5G19100	UGT76E1
STZ	AR781	NAC3	DIN11	AT4G22610	SQP2	AACT1

b

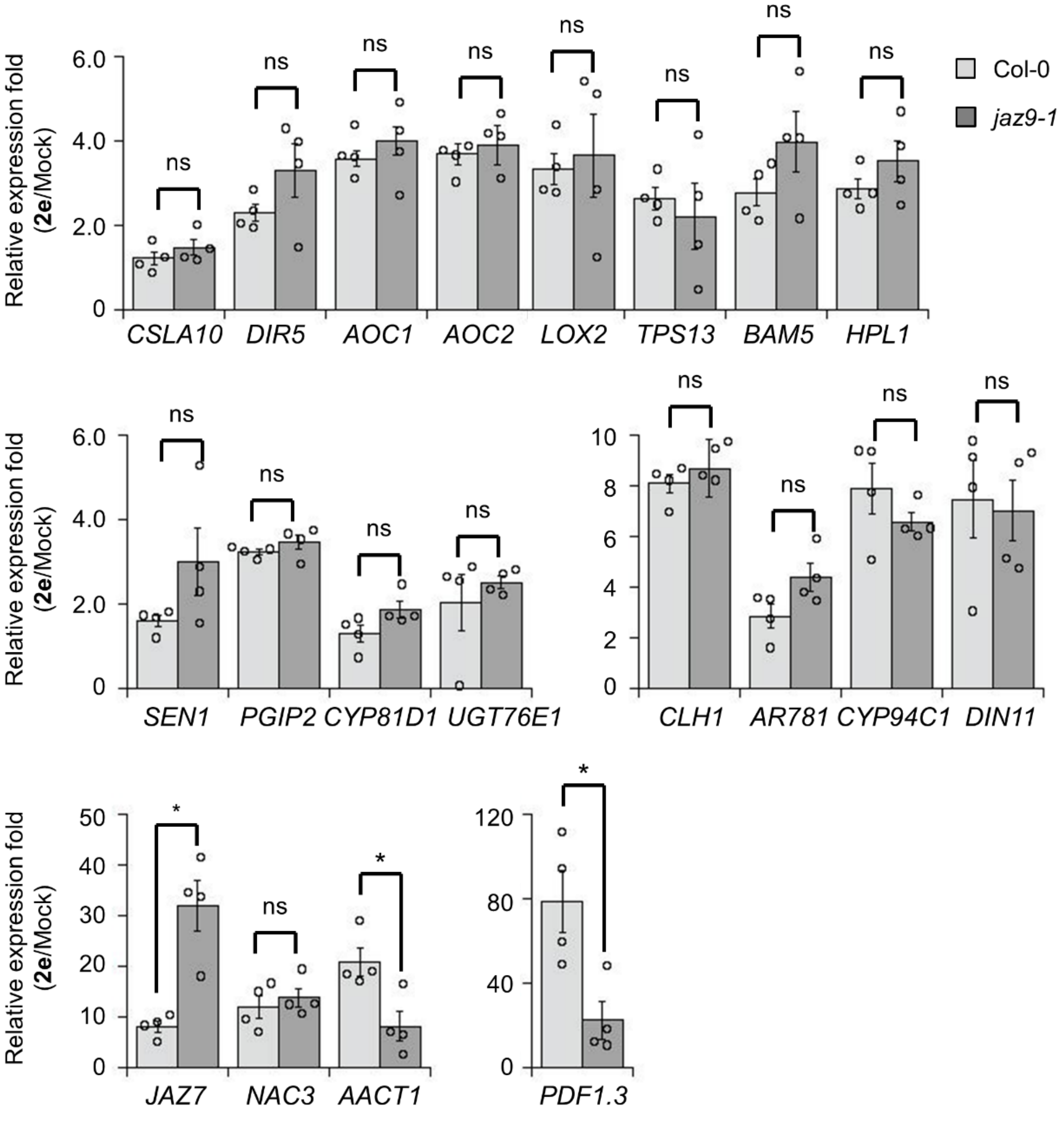

Supplementary Fig. 21. (**a**) List of up-regulated 49 genes in intersection of **2e**-treated Col-0 and *jaz9-1* not in mock-treated *jaz9-1* in Venn diagram shown in Fig. 4f. (**b**) RT-qPCR analyses in Col-0 and *jaz9-1* seedling (n=4) with or without compound treatment (1 μM). Each relative expression fold was calculated using an average expression level in Mock- or **2e**-treated Col-0 or *jaz9-1* seedlings. RT-qPCR data of *ORA59*, *JAZ1*, *MYC2*, *ERF1*, *AOS*, and *PDF1.2* are presented in Fig. 4g. The expressions

of *PDF1.3* and *AACT1* were suppressed in *jaz9-1*. Similar results were obtained in three independent experiments. Similar results were obtained in three independent experiments. Significant differences were evaluated by Student's t-test (**p < 0.01, *p < 0.05; ns, not significant). Values are mean ± SE.

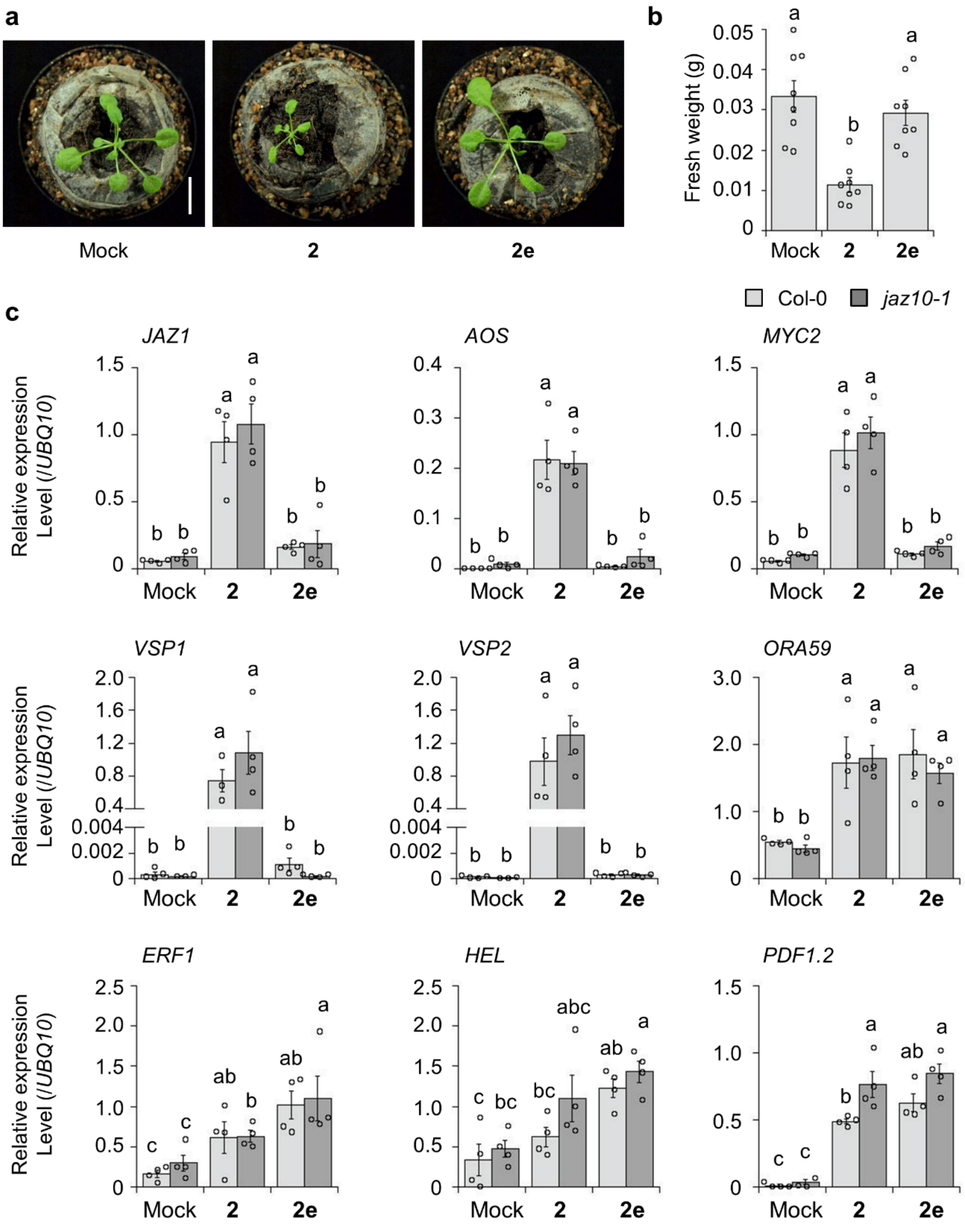

Supplementary Fig. 22. ***In planta*** **analyses of stereoisomers in** ***jaz10-1*****.** (**a** and **b**) Growth inhibition assay using ligand-treated adult plants. (**a**) Representative images of the **2**- (50 µM) and **2e**-treated (50 µM) adult plants. Similar results were obtained in three independent experiments. Scale bar: 1.0 cm. (**b**) Quantification of fresh weight of aerial

part of the ligand-treated plants (n=8). Significant differences were evaluated by one-way ANOVA/Tukey HSD post hoc test ($p < 0.05$). Values are mean ± SE. (**c**) Gene expression analyses of JA marker genes, genes belonging to MYC-branch, and belonging to ERF/ORA branch by RT-qPCR in Col-0 and *jaz10-1* seedling (n=4) with or without compound treatment (1 μM) for 2 h (*JAZ1*, *AOS*, *MYC2*, and *ORA59*) or 8 h (*VSP1*, *VSP2*, *ERF1*, *HEL*, and *PDF1.2*). Similar results were obtained in three independent experiments. Significant differences were evaluated by one-way ANOVA/Tukey HSD post hoc test ($p < 0.05$). Values are mean ± SE.

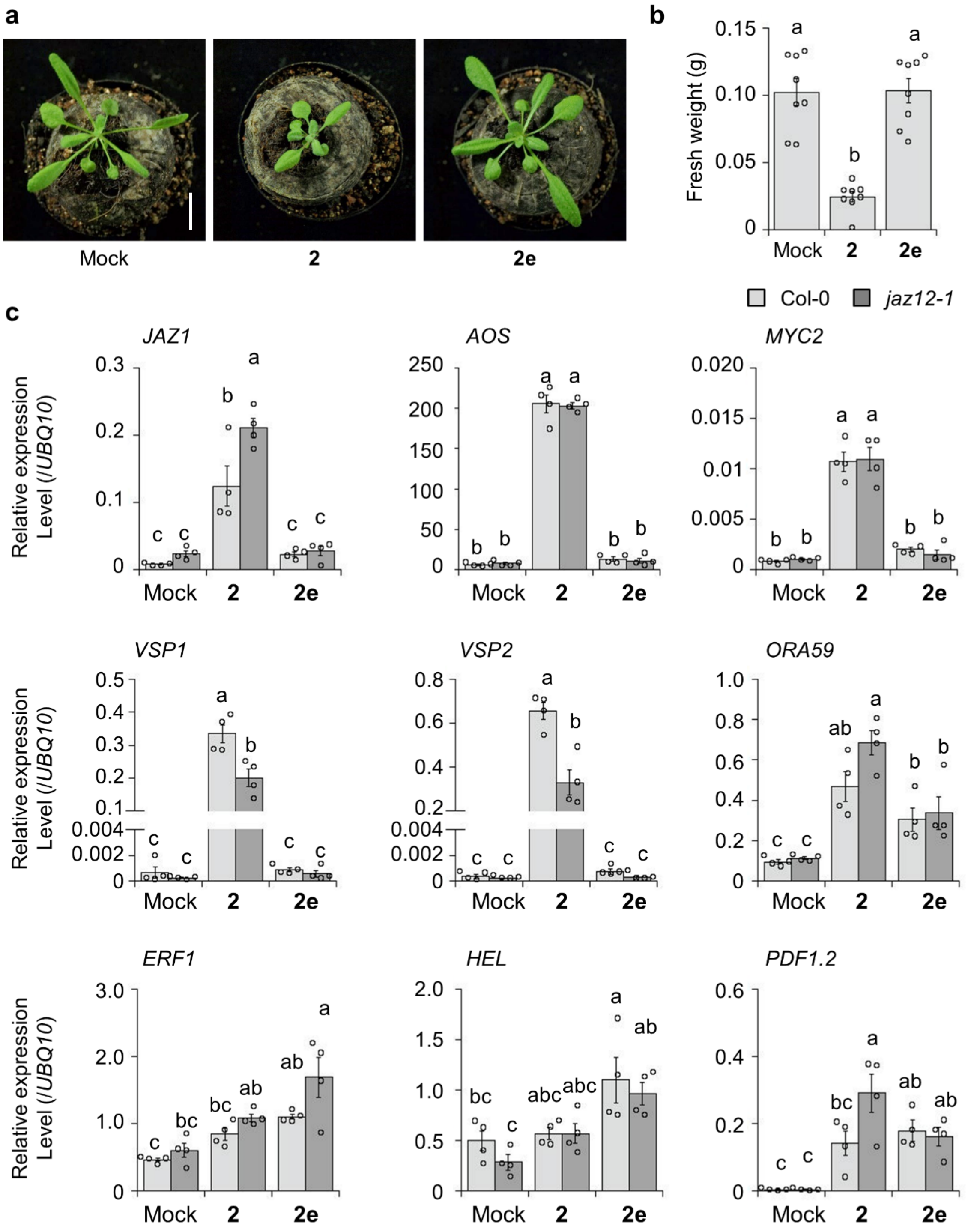

Supplementary Fig. 23. ***In planta*** **analyses of stereoisomers in** ***jaz12-1*****.** (**a** and **b**) Growth inhibition assay using ligand-treated adult plants. (**a**) Representative images of the **2**- (50 µM) and **2e**-treated (50 µM) adult plants. Similar results were obtained in three independent experiments. Scale bar: 1.0 cm. (**b**) Quantification of fresh weight of aerial part of the ligand-treated plants (n=8). Significant differences were evaluated by one-way

ANOVA/Tukey HSD post hoc test ($p < 0.05$). Values are mean ± SE. (**c**) Gene expression analyses of JA marker genes, genes belonging to MYC-branch, and belonging to ERF/ORA branch by RT-qPCR in Col-0 and *jaz12-1* seedling (n=4) with or without compound treatment (1 μM) for 2 h (*JAZ1*, *AOS*, *MYC2*, and *ORA59*) or 8 h (*VSP1*, *VSP2*, *ERF1*, *HEL*, and *PDF1.2*). Similar results were obtained in three independent experiments. Significant differences were evaluated by one-way ANOVA/Tukey HSD post hoc test ($p < 0.05$). Values are mean ± SE.

~~Legend of Dataset S1.~~ Legend of Supplementary Data 1

RNAseq data through quantile normalization and log 2 transformations. One-way ANOVA was performed, and only genes with FDR values less than 0.1 were shown. Further, Tuckey's HSD test with FDR was performed for individual combinations.

~~Legend of Dataset S2.~~ Legend of Supplementary Data 2

Primer sequences were used in this study.

Legend of Supplementary Data 3

Source data of all bar and box plots in the main text.

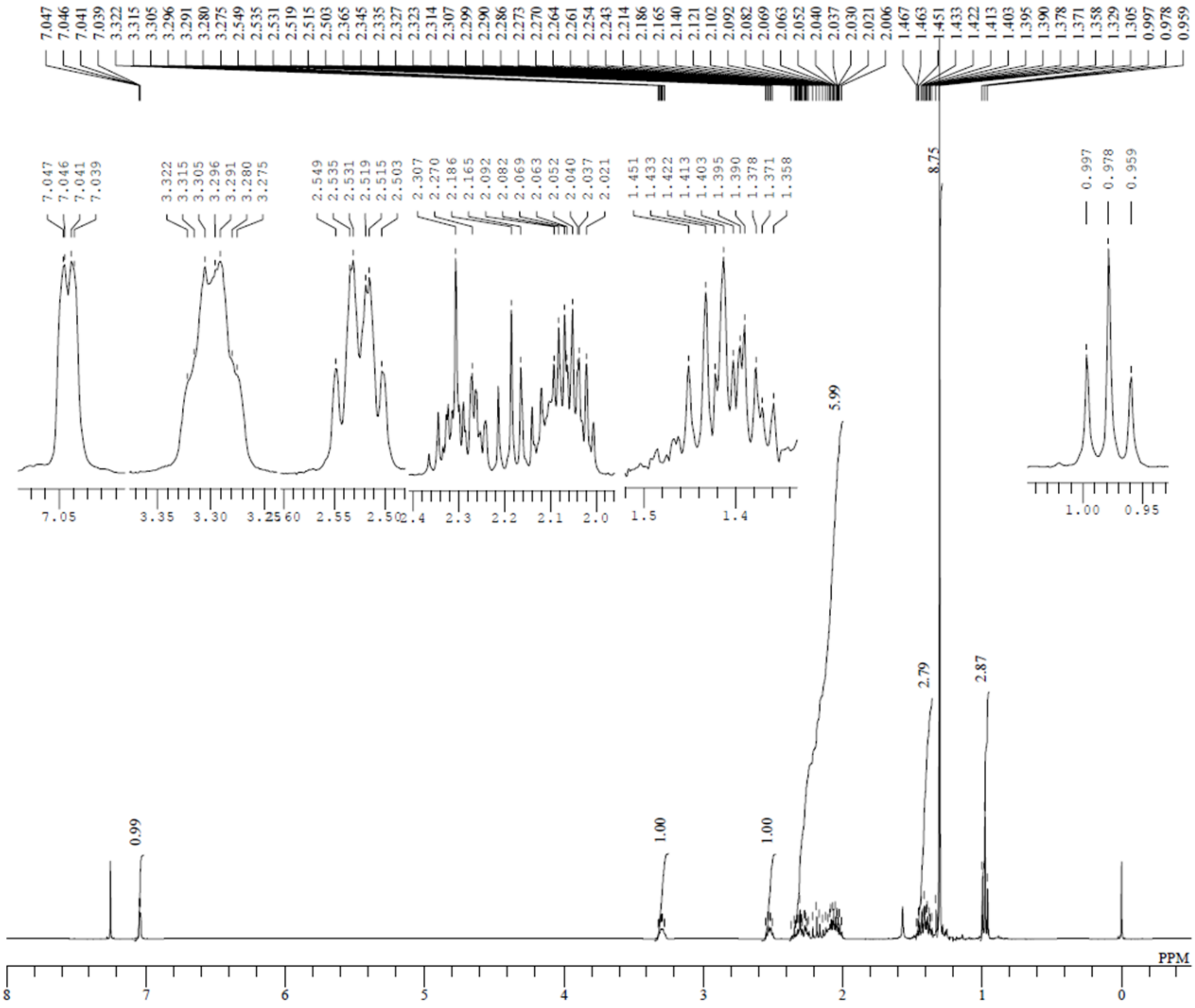

DFILE	07019p2-1.als
COMNT	single_pulse
DATIM	20-04-2020 20:28:45
OBNUC	1H
EXMOD	proton.jxp
OBFRQ	399.78 MHz
OBSET	4.19 KHz
OBFIN	7.29 Hz
POINT	13120
FREQU	6002.40 Hz
SCANS	8
ACQTM	2.1837 sec
PD	5.0000 sec
PW1	2.95 usec
IRNUC	1H
CTEMP	23.4 c
SLVNT	CDCL3
EXREF	0.00 ppm
BF	0.25 Hz
RGAIN	48

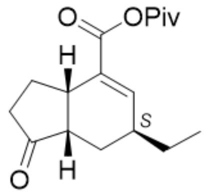

(+)-**10**

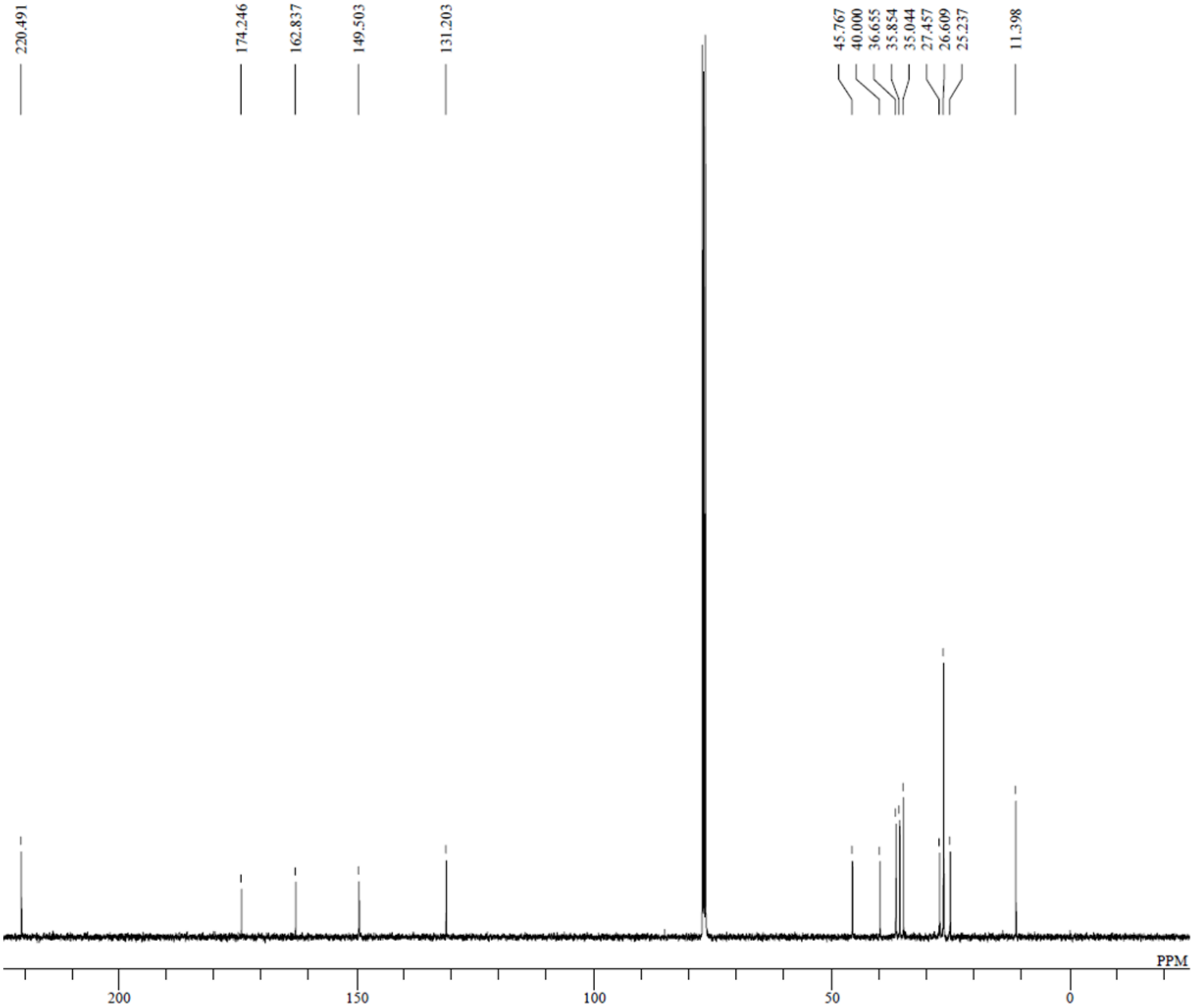

DFILE	3-201-3_Carbon-1-1.als
COMNT	single pulse decoupled gated N
DATIM	06-11-2021 20:32:46
OBNUC	13C
EXMOD	carbon.jxp
OBFRQ	100.53 MHz
OBSET	5.35 KHz
OBFIN	5.86 Hz
POINT	32780
FREQU	31407.04 Hz
SCANS	1793
ACQTM	1.0433 sec
PD	2.0000 sec
PW1	3.37 usec
IRNUC	1H
CTEMP	21.5 c
SLVNT	CDCL3
EXREF	77.00 ppm
BF	0.25 Hz
RGAIN	50

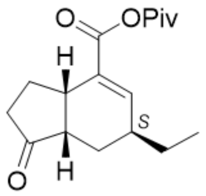

(+)-**10**

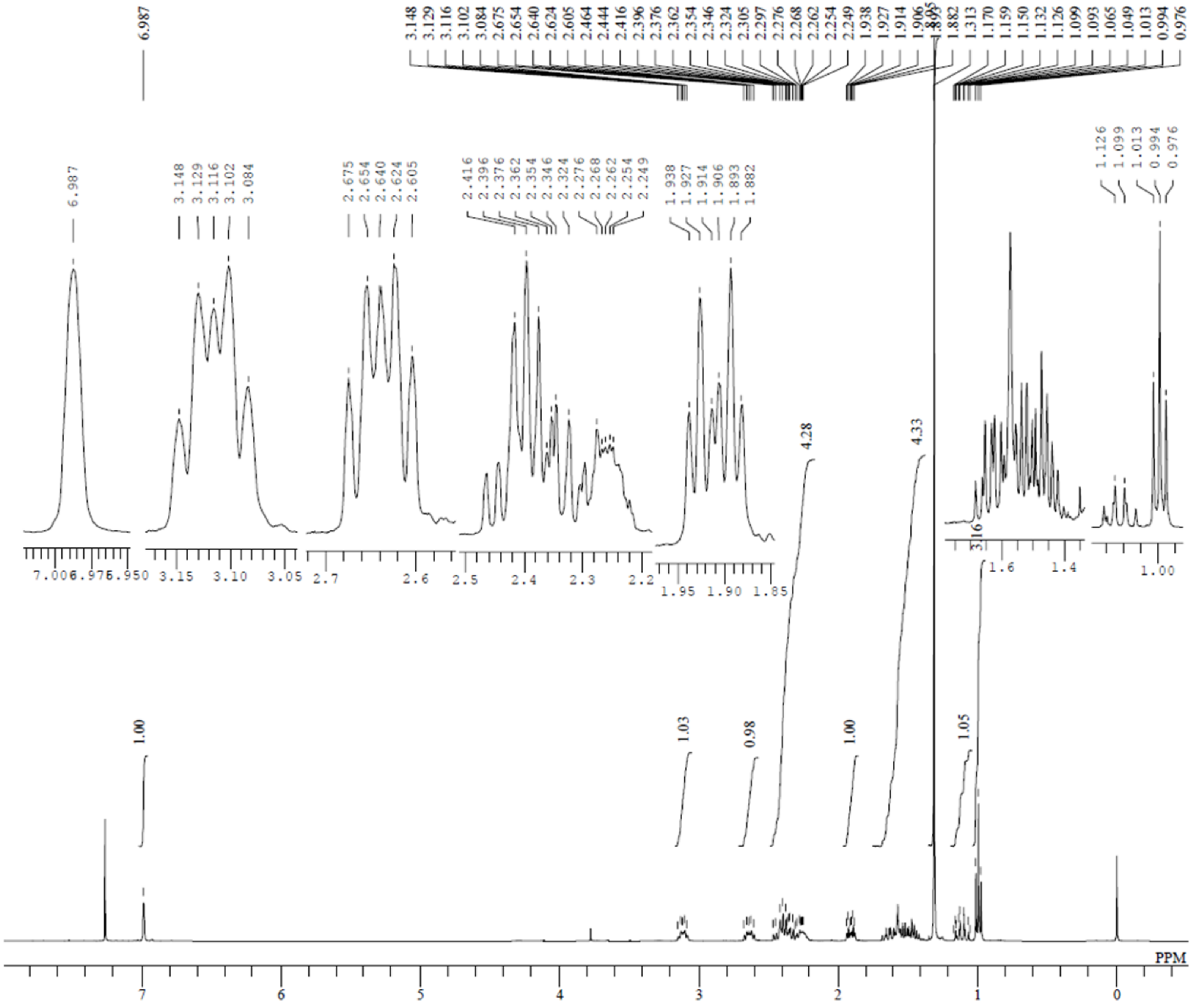

DFILE 3-204-13_Proton-1-1.als
COMNT single_pulse
DATIM 23-11-2021 21:44:31
OBNUC 1H
EXMOD proton.jxp
OBFRQ 399.78 MHz
OBSET 4.19 KHz
OBFIN 7.29 Hz
POINT 16384
FREQU 7503.00 Hz
SCANS 32
ACQTM 2.1837 sec
PD 5.0000 sec
PW1 2.95 usec
IRNUC 1H
CTEMP 21.3 c
SLVNT CDCL3
EXREF 0.00 ppm
BF 0.25 Hz
RGAIN 60

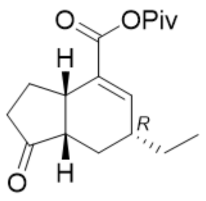

6*R*-isomer

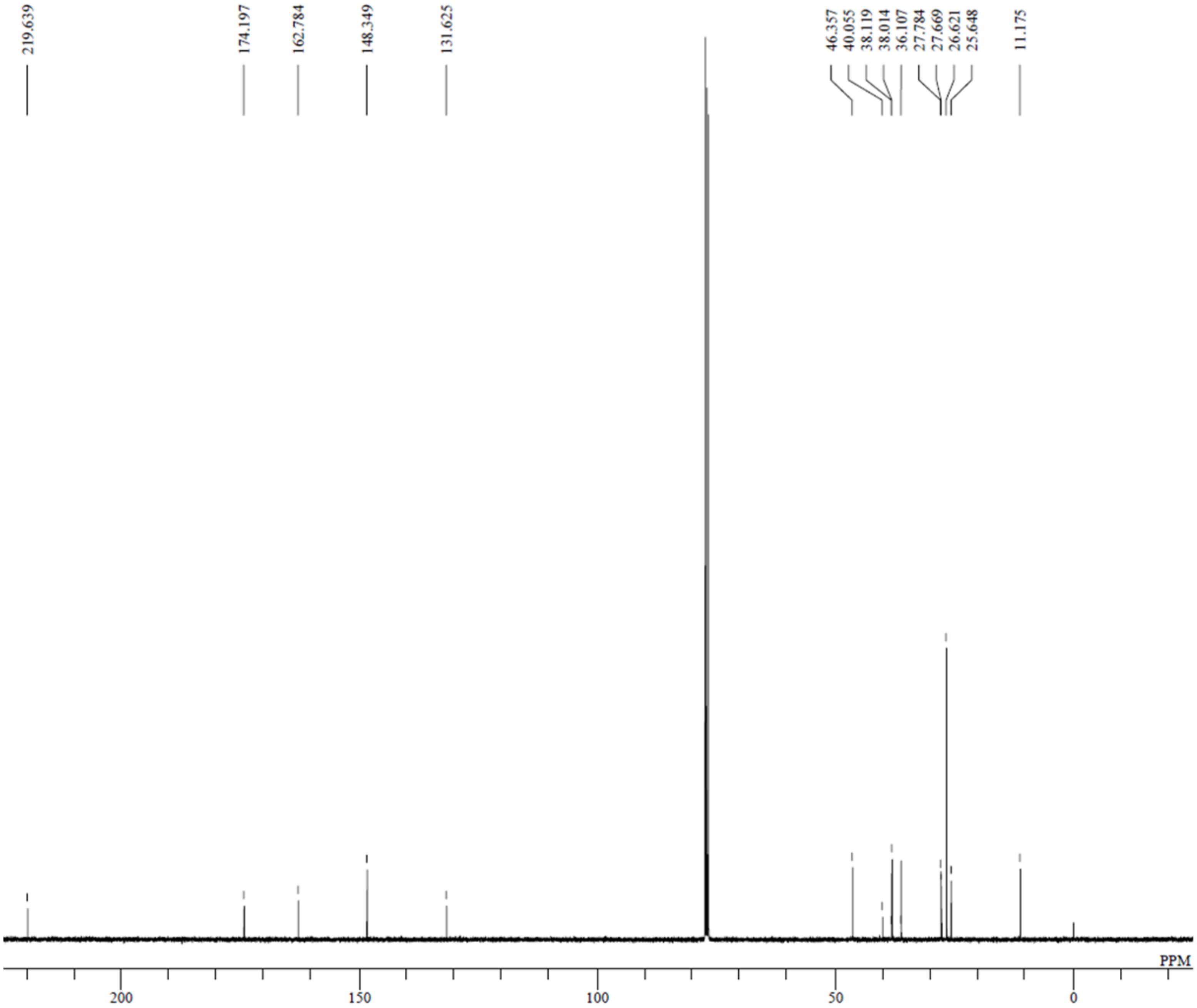

DFILE	211129_3-204-13ON_Carbon-
COMNT	single pulse decoupled gated N
DATIM	30-11-2021 00:00:33
OBNUC	13C
EXMOD	carbon.jxp
OBFRQ	100.53 MHz
OBSET	5.35 KHz
OBFIN	5.86 Hz
POINT	32767
FREQU	31407.04 Hz
SCANS	8192
ACQTM	1.0433 sec
PD	2.0000 sec
PW1	3.37 usec
IRNUC	1H
CTEMP	23.6 c
SLVNT	CDCL3
EXREF	0.00 ppm
BF	0.25 Hz
RGAIN	50

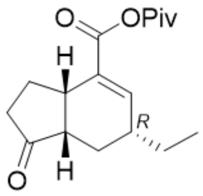

6*R*-isomer

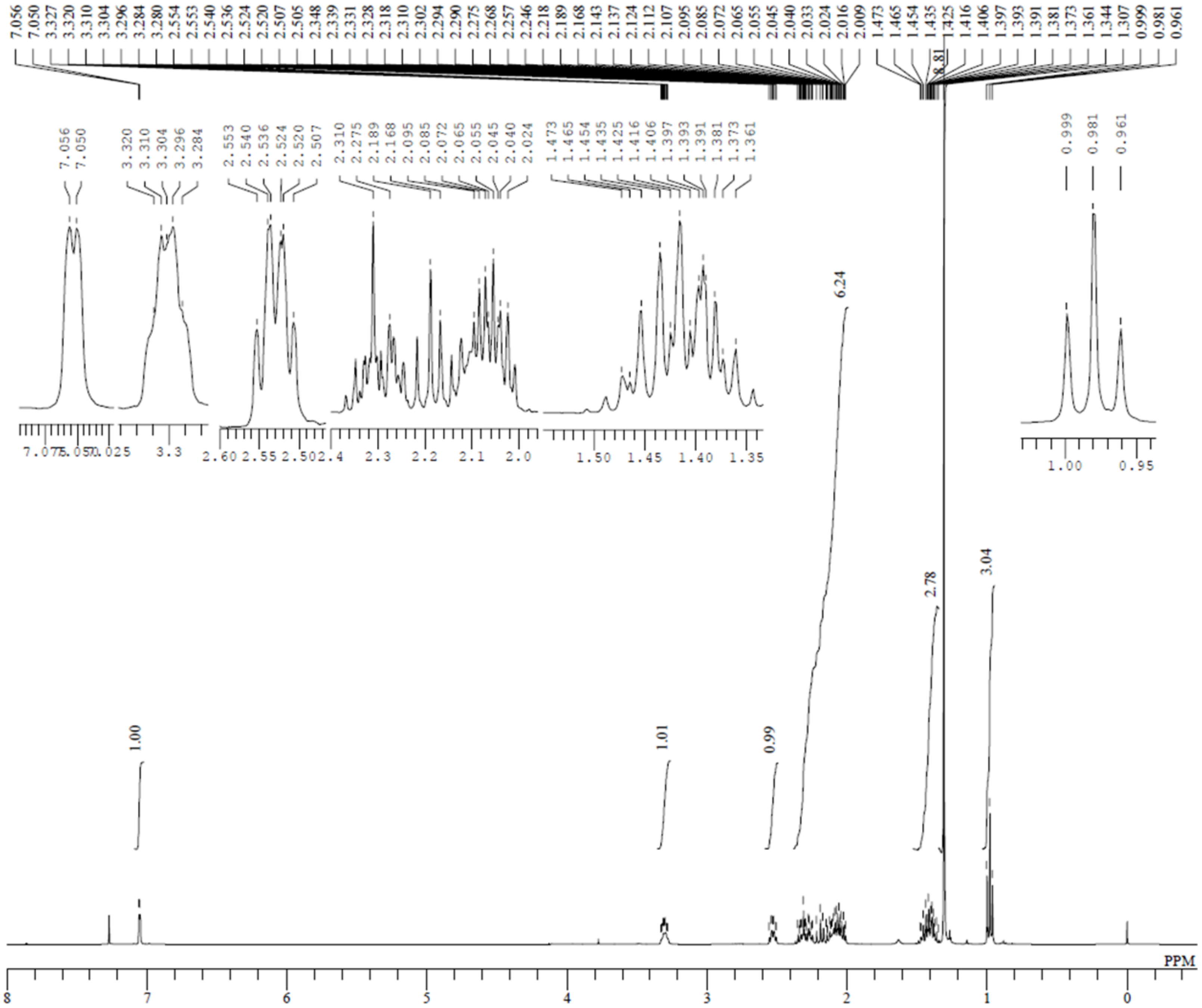

DFILE 3-206-9_Proton-1-1.als
COMNT single_pulse
DATIM 08-12-2021 22:12:11
OBNUC 1H
EXMOD proton.jxp
OBFRQ 399.78 MHz
OBSET 4.19 KHz
OBFIN 7.29 Hz
POINT 16384
FREQU 7503.00 Hz
SCANS 8
ACQTM 2.1837 sec
PD 5.0000 sec
PW1 2.95 usec
IRNUC 1H
CTEMP 21.6 c
SLVNT CDCL3
EXREF 0.00 ppm
BF 0.25 Hz
RGAIN 54

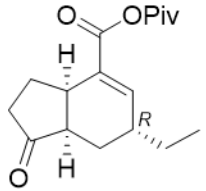

(−)-**10**

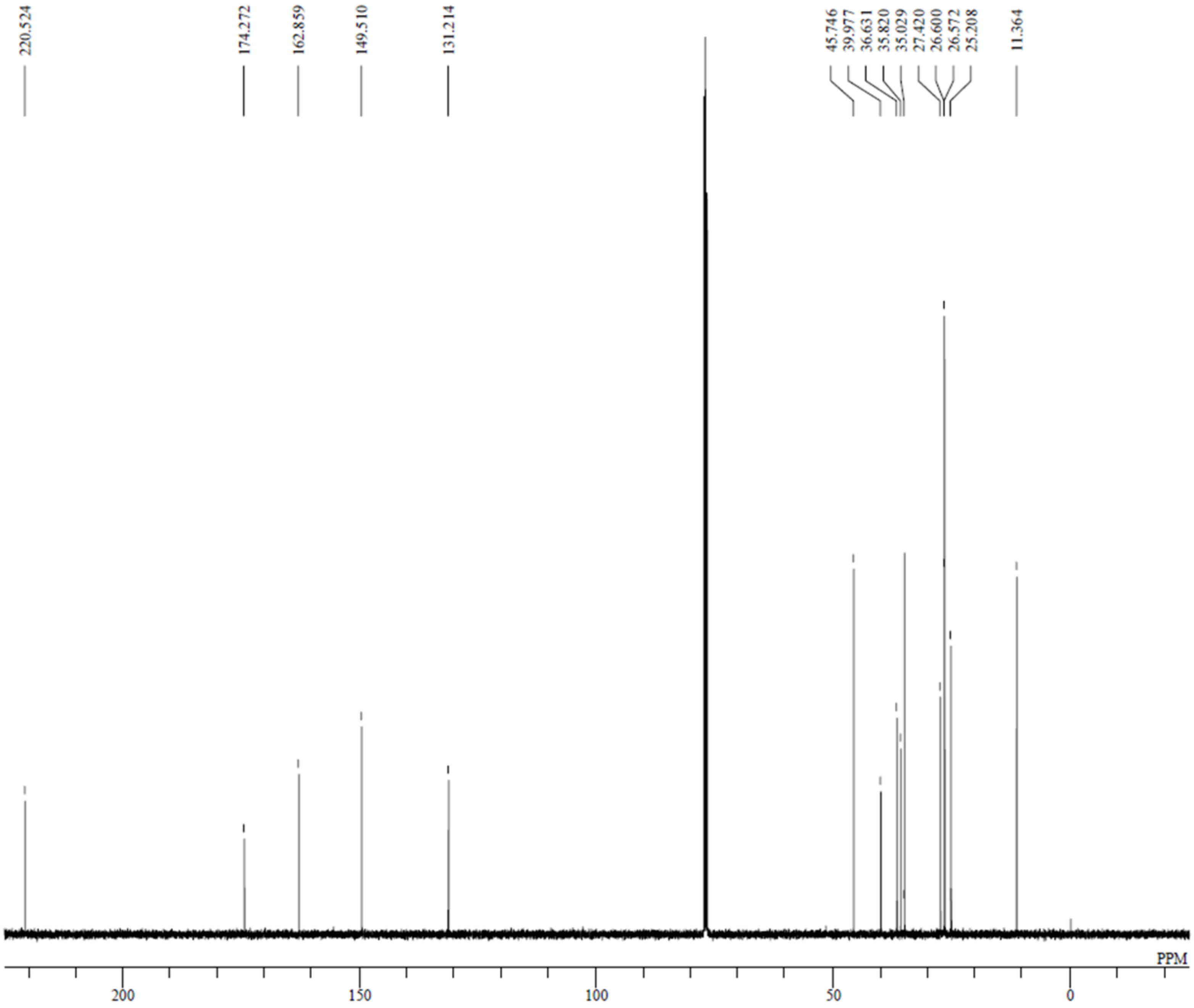

DFILE 3-206-9_Carbon-1-1.als
COMNT single pulse decoupled gated
DATIM 08-12-2021 22:55:29
OBNUC 13C
EXMOD carbon.jxp
OBFRQ 100.53 MHz
OBSET 5.35 KHz
OBFIN 5.86 Hz
POINT 32767
FREQU 31407.04 Hz
SCANS 2048
ACQTM 1.0433 sec
PD 2.0000 sec
PW1 3.37 usec
IRNUC 1H
CTEMP 21.3 c
SLVNT CDCL3
EXREF 77.00 ppm
BF 0.25 Hz
RGAIN 50

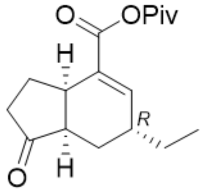

(−)-**10**

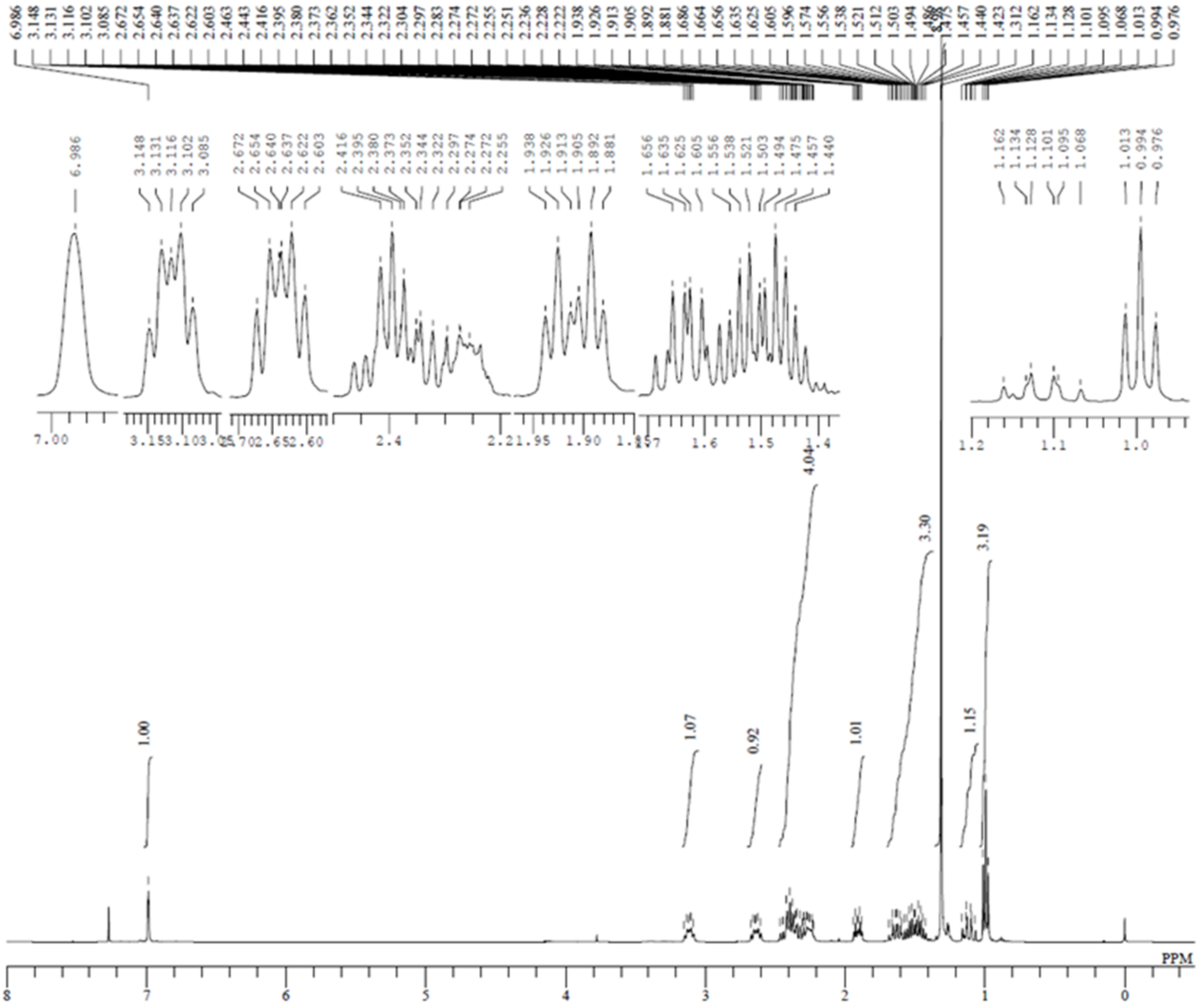
DFILE 3-206-6_Proton-1-1.als
COMNT single_pulse
DATIM 07-12-2021 15:18:44
OBNUC 1H
EXMOD proton.jxp
OBFRQ 399.78 MHz
OBSET 4.19 KHz
OBFIN 7.29 Hz
POINT 16384
FREQU 7503.00 Hz
SCANS 8
ACQTM 2.1837 sec
PD 5.0000 sec
PW1 2.95 usec
IRNUC 1H
CTEMP 24.4 c
SLVNT CDCL3
EXREF 0.00 ppm
BF 0.25 Hz
RGAIN 50
1.00
1.07
0.92
4.04
1.01
3.30
1.15
3.19
PPM

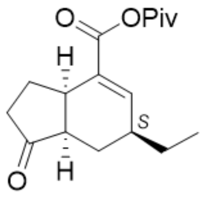
OPiv
H
H
S

6*S*-isomer

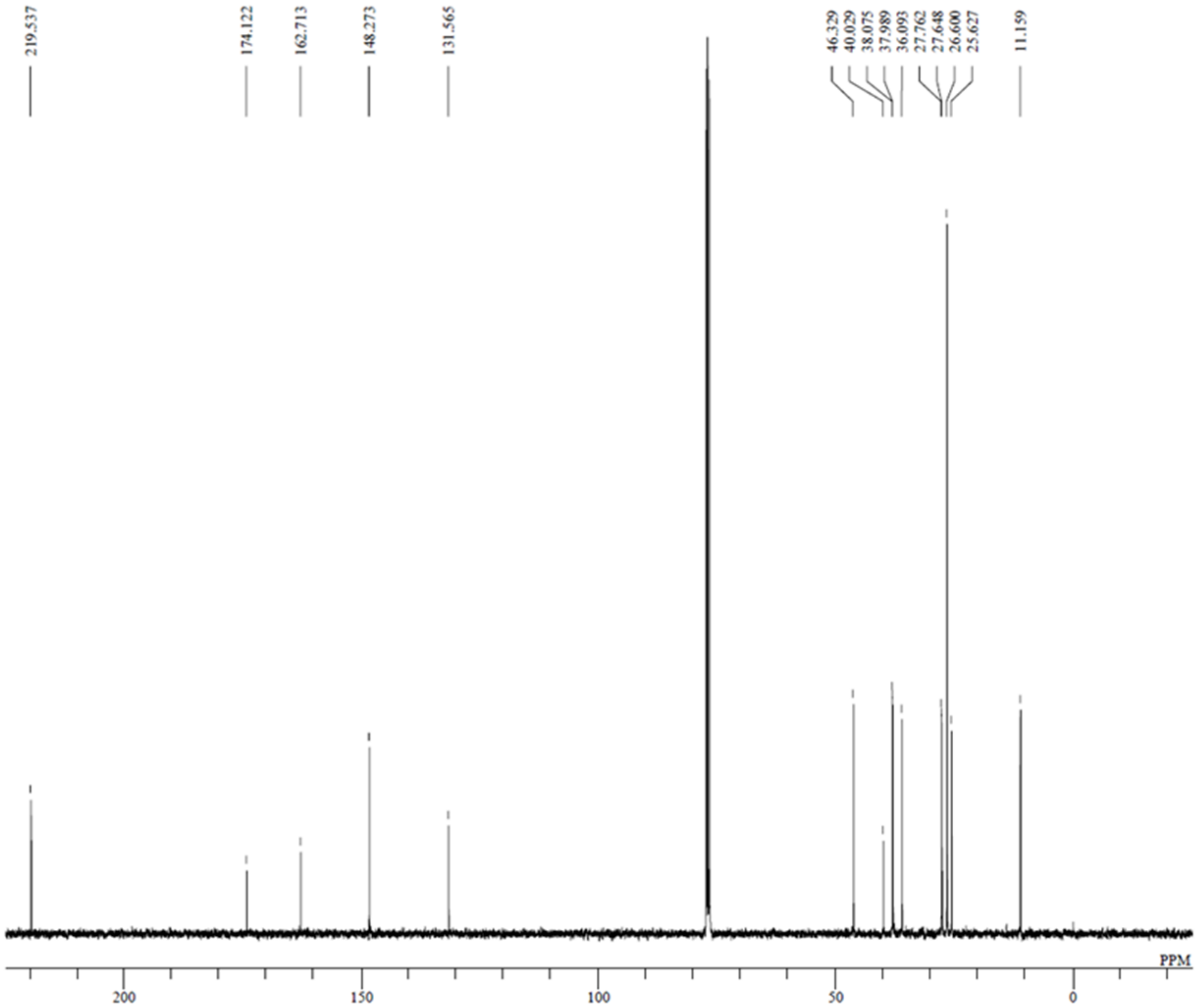

DFILE	3-206-6-save_Carbon-1-1.als
COMNT	single pulse decoupled gated N
DATIM	07-12-2021 15:49:32
OBNUC	13C
EXMOD	carbon.jxp
OBFRQ	100.53 MHz
OBSET	5.35 KHz
OBFIN	5.86 Hz
POINT	32780
FREQU	31407.04 Hz
SCANS	1184
ACQTM	1.0433 sec
PD	2.0000 sec
PW1	3.37 usec
IRNUC	1H
CTEMP	24.5 c
SLVNT	CDCL3
EXREF	77.00 ppm
BF	0.25 Hz
RGAIN	50

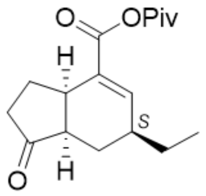

6*S*-isomer

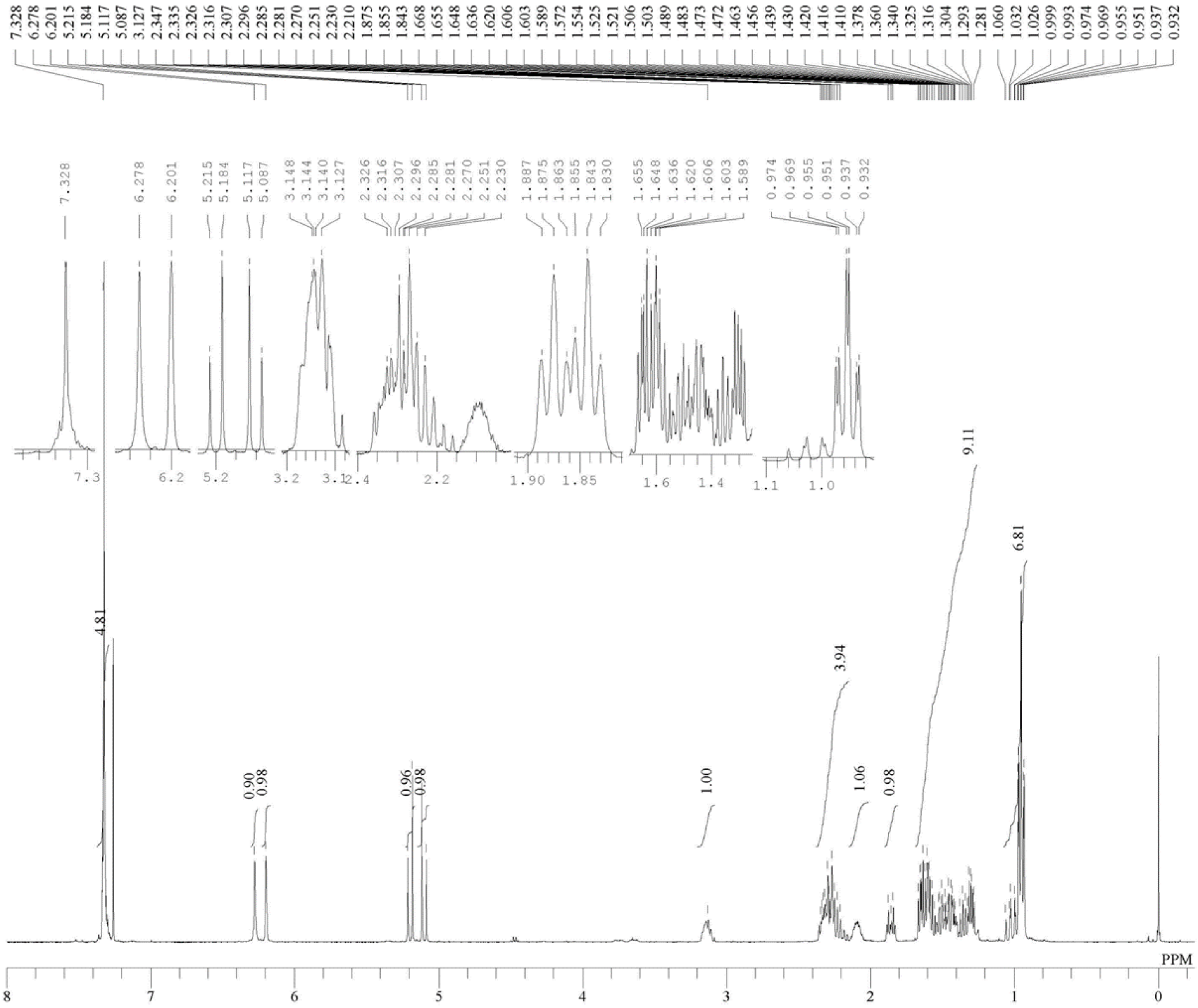

DFILE	3-150-1_Proton-1-1.als
COMNT	single_pulse
DATIM	21-12-2018 17:10:24
OBNUC	1H
EXMOD	proton.jxp
OBFRQ	399.78 MHz
OBSET	4.19 KHz
OBFIN	7.29 Hz
POINT	16384
FREQU	7503.00 Hz
SCANS	8
ACQTM	2.1837 sec
PD	5.0000 sec
PW1	2.95 usec
IRNUC	1H
CTEMP	20.7 c
SLVNT	CDCL3
EXREF	0.00 ppm
BF	0.10 Hz
RGAIN	56

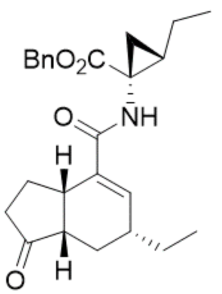

11

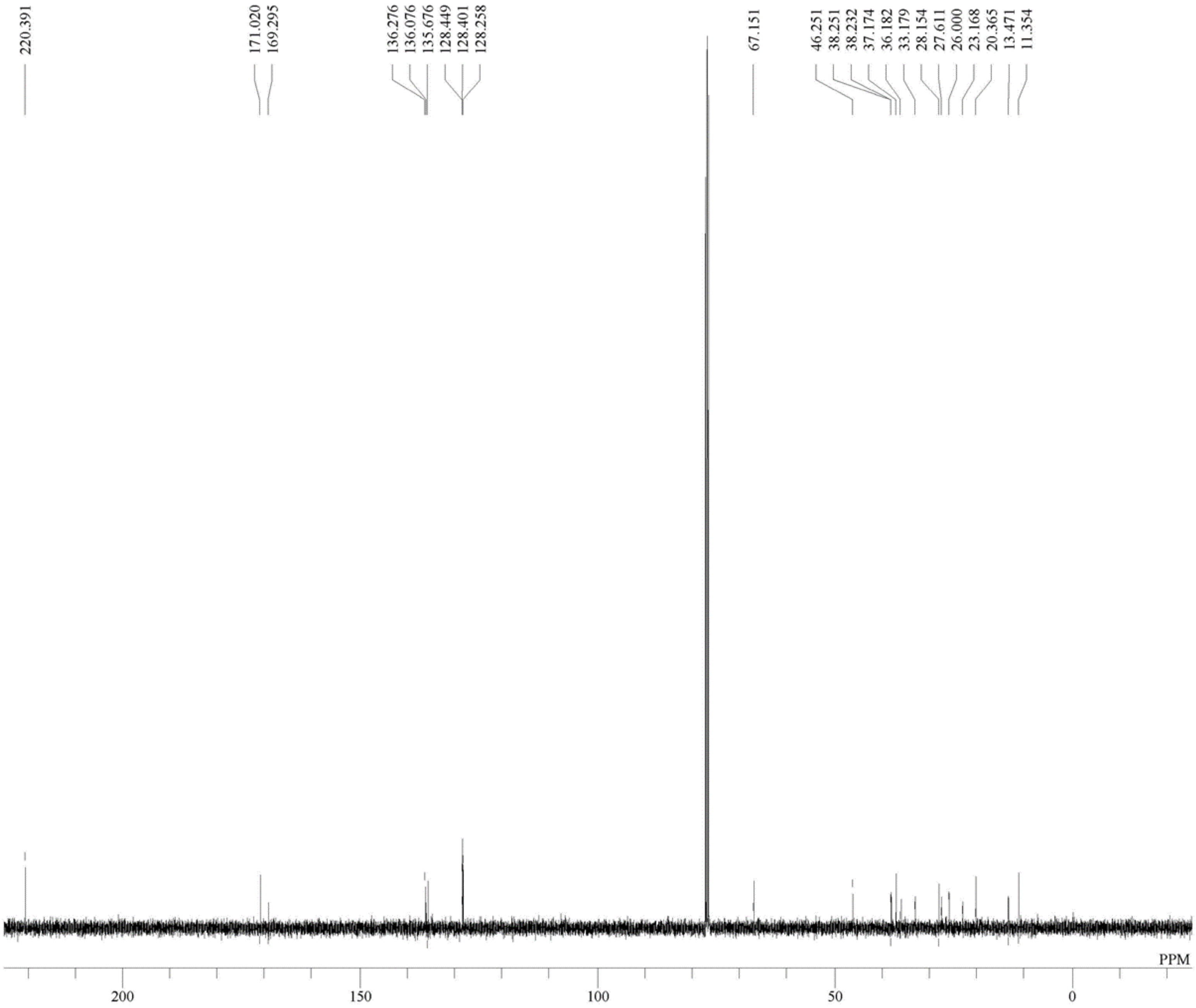

DFILE	3-150-1_Carbon-1-1.als
COMNT	single pulse decoupled gated N
DATIM	21-12-2018 17:11:42
OBNUC	13C
EXMOD	carbon.jxp
OBFRQ	100.53 MHz
OBSET	5.35 KHz
OBFIN	5.86 Hz
POINT	32767
FREQU	31407.04 Hz
SCANS	745
ACQTM	1.0433 sec
PD	2.0000 sec
PW1	3.37 usec
IRNUC	1H
CTEMP	20.8 c
SLVNT	CDCL3
EXREF	77.00 ppm
BF	0.10 Hz
RGAIN	50

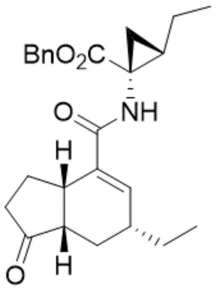

11

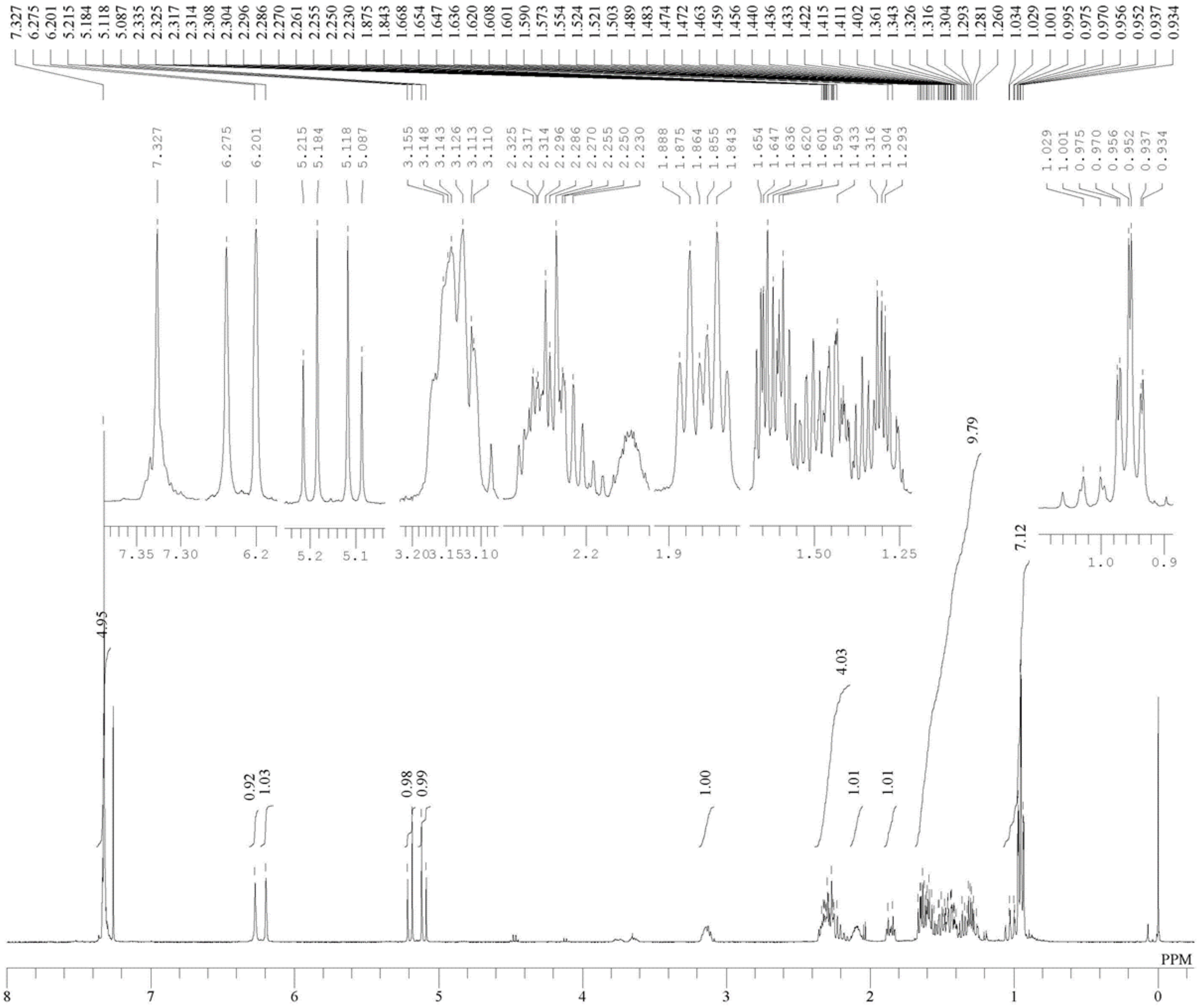

DFILE	3-138-7_Proton-1-1.als
COMNT	single_pulse
DATIM	29-11-2018 21:40:23
OBNUC	1H
EXMOD	proton.jxp
OBFRQ	399.78 MHz
OBSET	4.19 KHz
OBFIN	7.29 Hz
POINT	16384
FREQU	7503.00 Hz
SCANS	8
ACQTM	2.1837 sec
PD	5.0000 sec
PW1	2.95 usec
IRNUC	1H
CTEMP	22.1 c
SLVNT	CDCL3
EXREF	0.00 ppm
BF	0.10 Hz
RGAIN	56

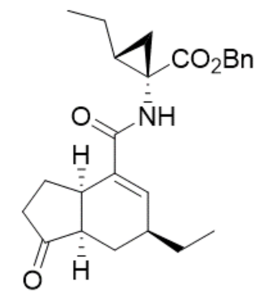

11a

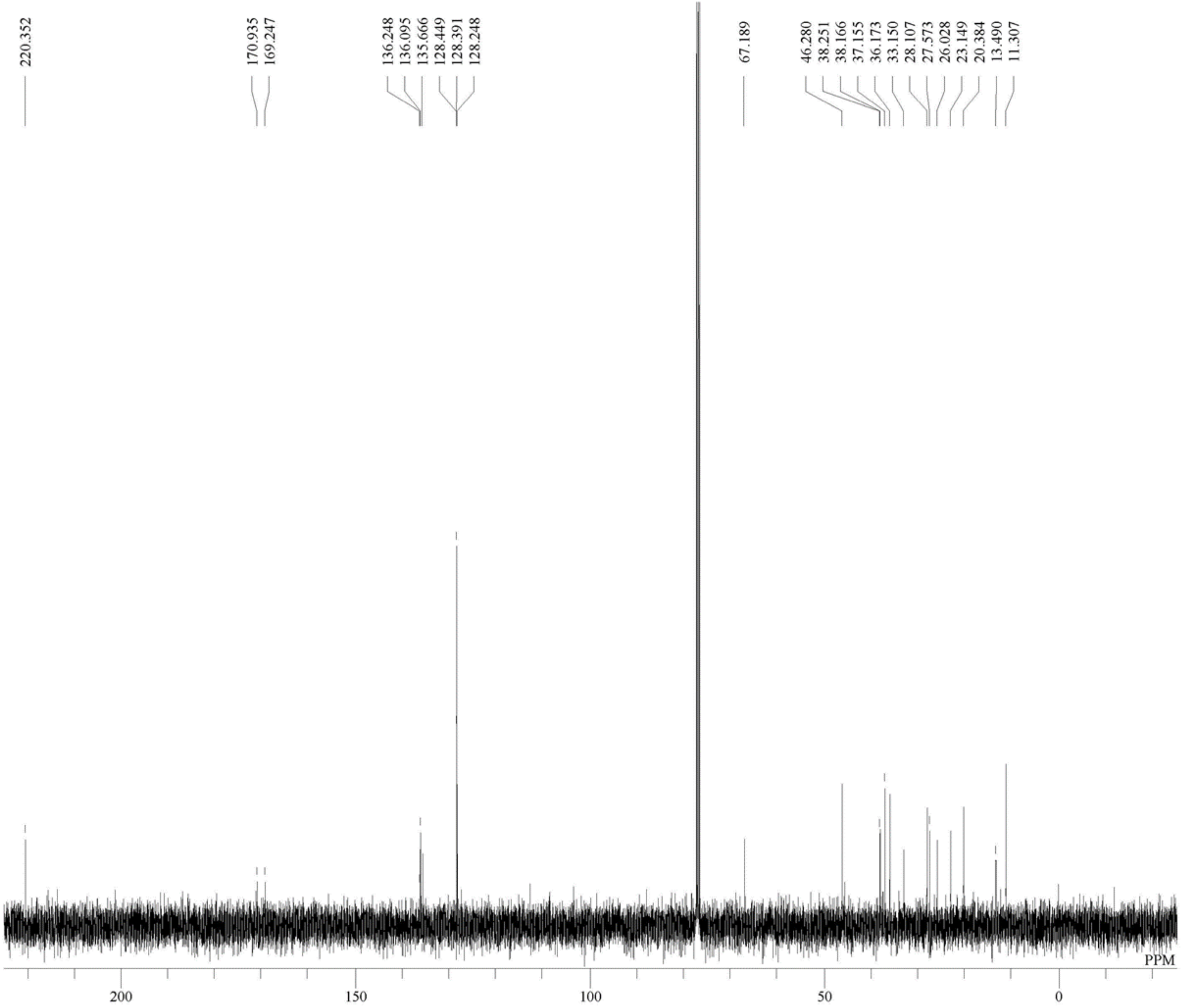

DFILE 3-138-7_Carbon-1-1.als
COMNT single pulse decoupled gated N
DATIM 29-11-2018 21:41:37
OBNUC 13C
EXMOD carbon.jxp
OBFRQ 100.53 MHz
OBSET 5.35 KHz
OBFIN 5.86 Hz
POINT 32767
FREQU 31407.04 Hz
SCANS 497
ACQTM 1.0433 sec
PD 2.0000 sec
PW1 3.37 usec
IRNUC 1H
CTEMP 22.1 c
SLVNT CDCL3
EXREF 77.00 ppm
BF 0.10 Hz
RGAIN 50

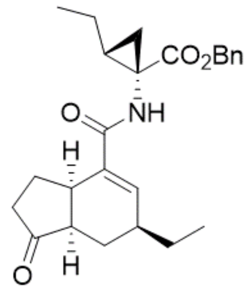

11a

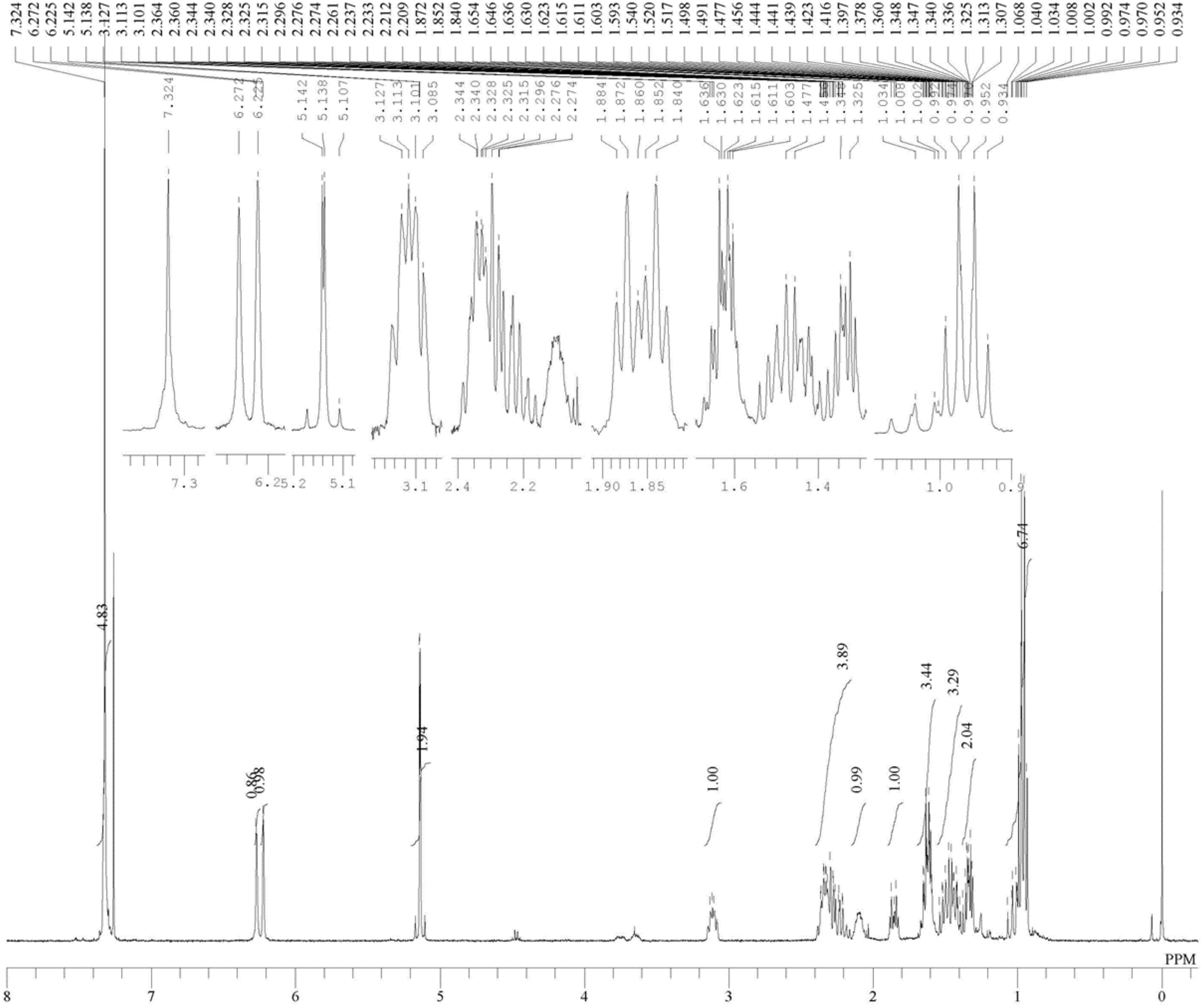

DFILE	3-138-5_Proton-1-1.als
COMNT	single_pulse
DATIM	29-11-2018 20:39:18
OBNUC	1H
EXMOD	proton.jxp
OBFRQ	399.78 MHz
OBSET	4.19 KHz
OBFIN	7.29 Hz
POINT	16384
FREQU	7503.00 Hz
SCANS	8
ACQTM	2.1837 sec
PD	5.0000 sec
PW1	2.95 usec
IRNUC	1H
CTEMP	22.0 c
SLVNT	CDCL3
EXREF	0.00 ppm
BF	0.10 Hz
RGAIN	62

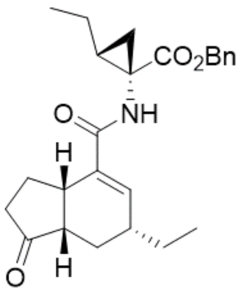

11b

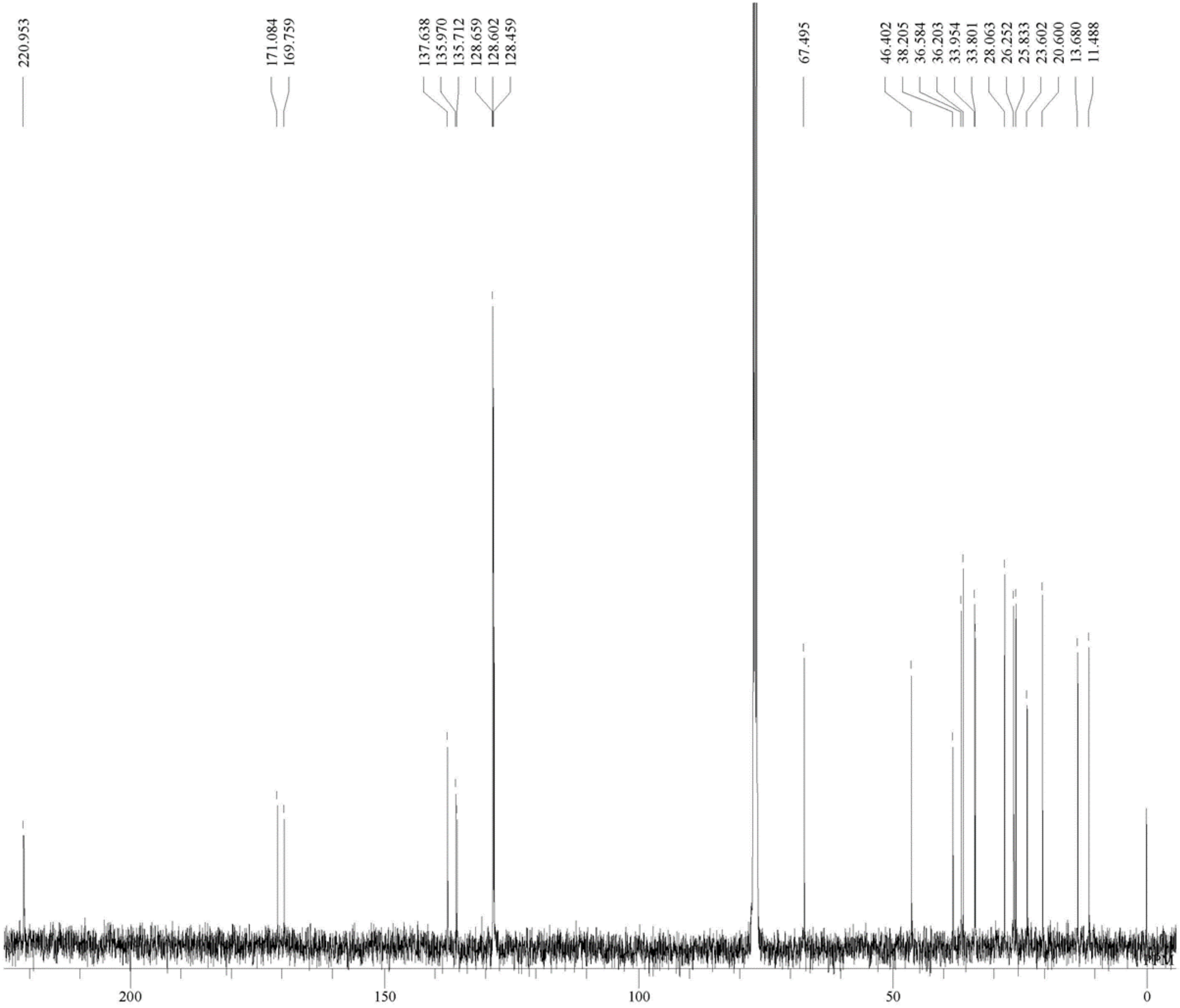

DFILE	12b_Carbon-1-1.als
COMNT	st_2_104p_C6+eCMA
DATIM	07/Jun/2017 00:40:24
OBNUC	13C
EXMOD	carbon.jxp
OBFRQ	100.53 MHz
OBSET	-5.00 KHz
OBFIN	304.55 Hz
POINT	26224
FREQU	25124.29 Hz
SCANS	9517
ACQTM	1.0438 sec
PD	2.0000 sec
PW1	3.37 usec
IRNUC	NUL
CTEMP	27.3 c
SLVNT	CHLOROFORM
EXREF	77.16 ppm
BF	0.25 Hz
RGAIN	50

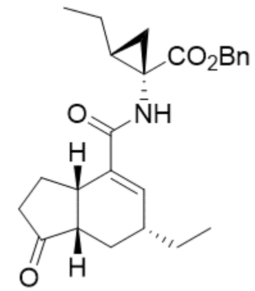

11b

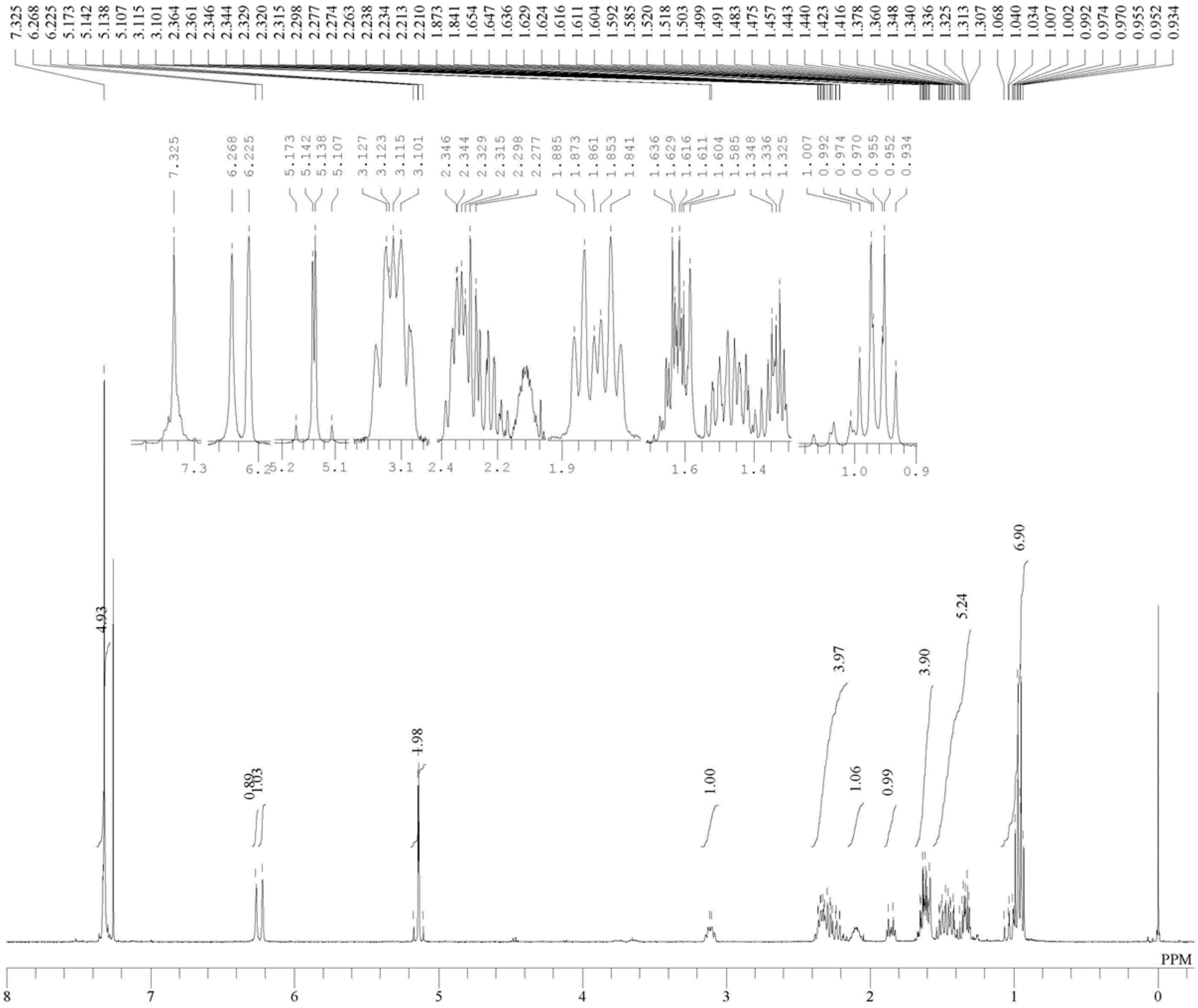

DFILE 3-150-3_Proton-1-1.als
COMNT single_pulse
DATIM 21-12-2018 18:02:31
OBNUC 1H
EXMOD proton.jxp
OBFRQ 399.78 MHz
OBSET 4.19 KHz
OBFIN 7.29 Hz
POINT 16384
FREQU 7503.00 Hz
SCANS 8
ACQTM 2.1837 sec
PD 5.0000 sec
PW1 2.95 usec
IRNUC 1H
CTEMP 20.8 c
SLVNT CDCL3
EXREF 0.00 ppm
BF 0.10 Hz
RGAIN 60

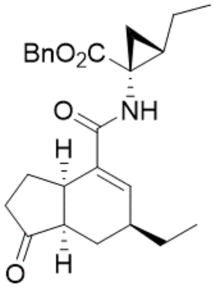

11c

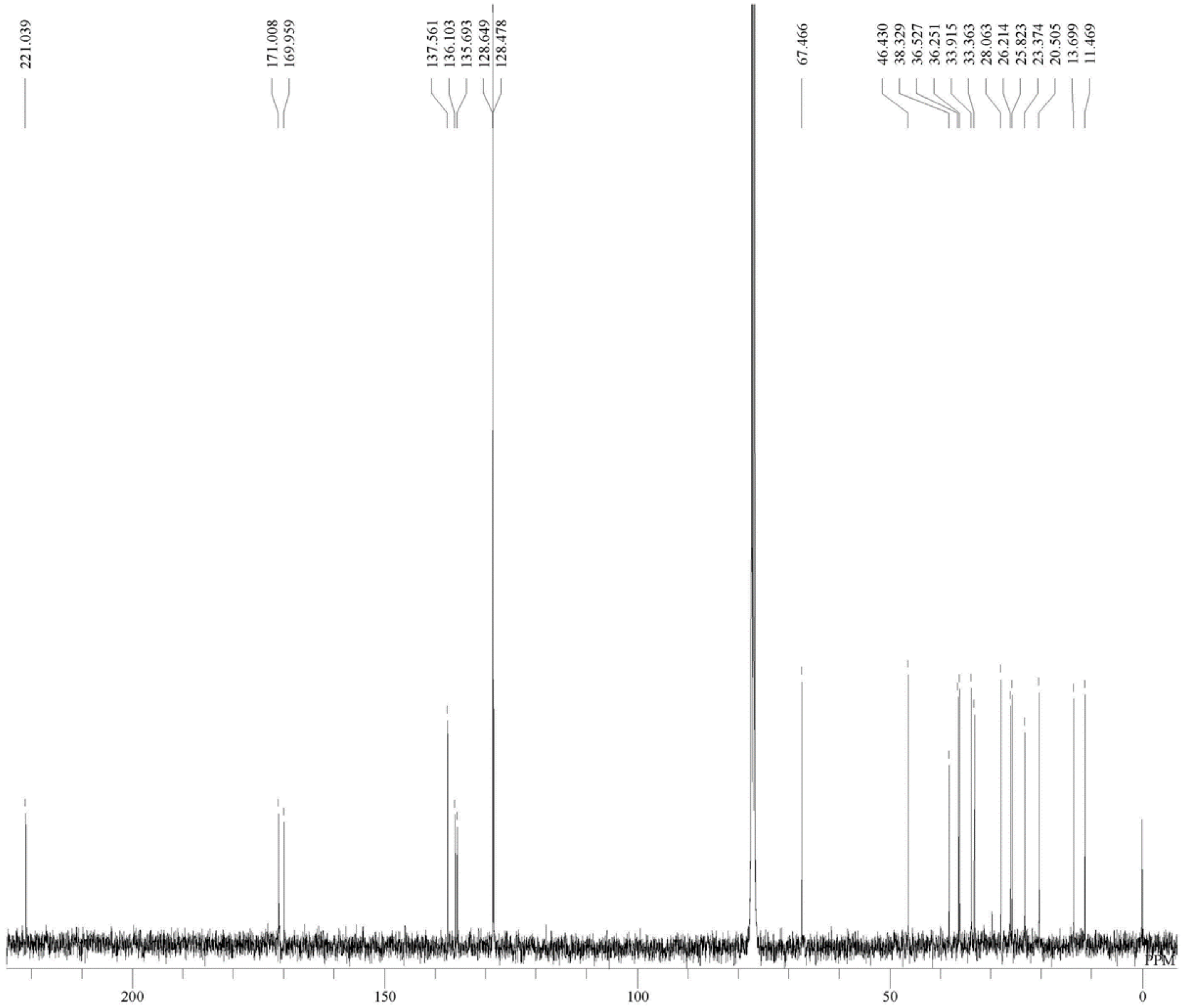

DFILE	12c_Carbon-1-1.als
COMNT	st_2_100_C6+nCMA-Bn
DATIM	06/Jun/2017 01:15:06
OBNUC	13C
EXMOD	carbon.jxp
OBFRQ	100.53 MHz
OBSET	-5.00 KHz
OBFIN	304.55 Hz
POINT	26224
FREQU	25124.29 Hz
SCANS	10699
ACQTM	1.0438 sec
PD	2.0000 sec
PW1	3.37 usec
IRNUC	NUL
CTEMP	24.1 c
SLVNT	CHLOROFORM
EXREF	77.16 ppm
BF	0.25 Hz
RGAIN	50

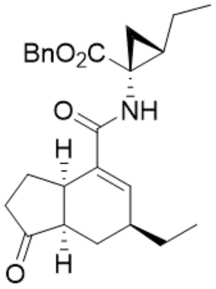

11c

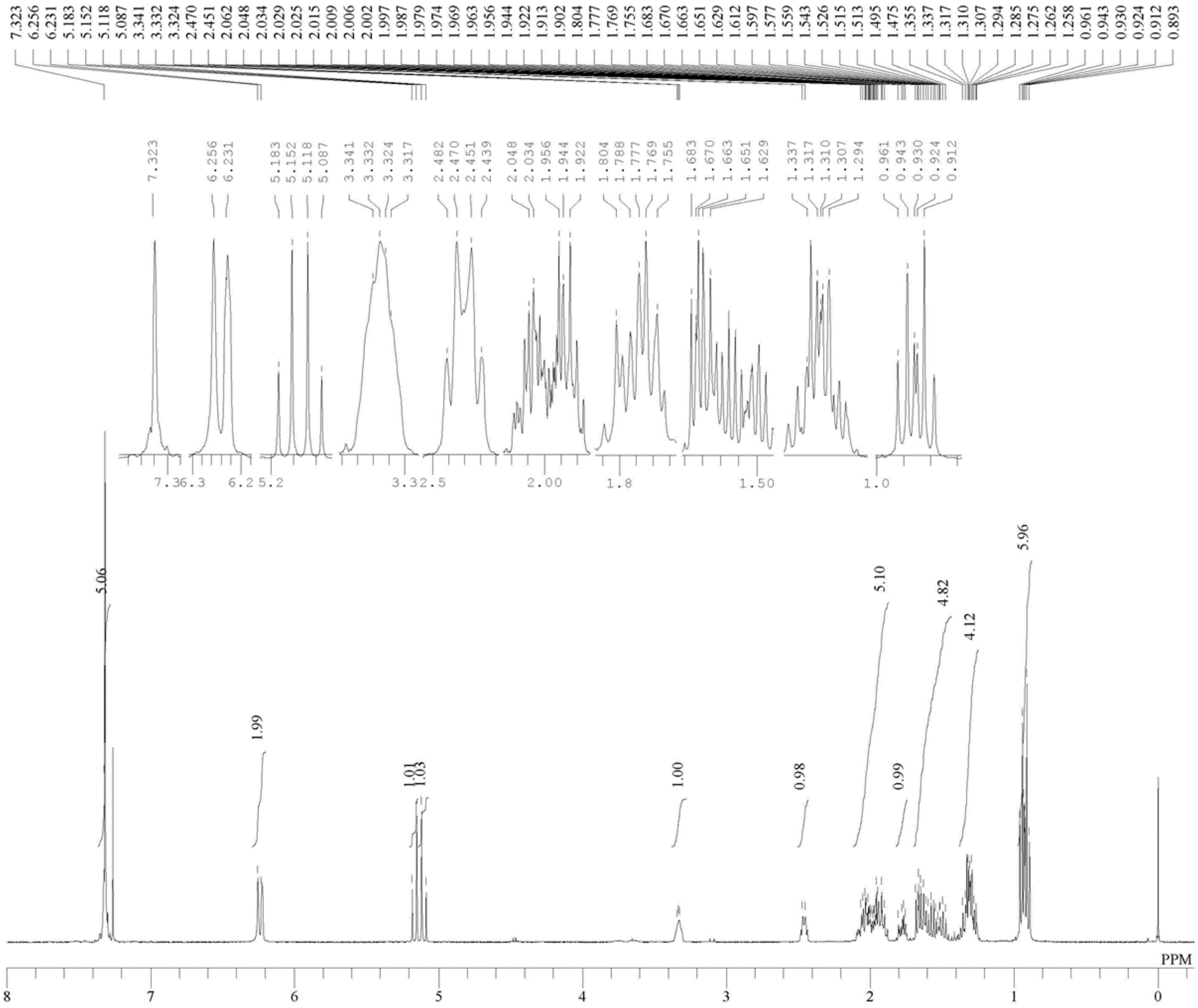

DFILE	3-150-2_Proton-1-1.als
COMNT	single_pulse
DATIM	21-12-2018 17:55:23
OBNUC	1H
EXMOD	proton.jxp
OBFRQ	399.78 MHz
OBSET	4.19 KHz
OBFIN	7.29 Hz
POINT	16384
FREQU	7503.00 Hz
SCANS	8
ACQTM	2.1837 sec
PD	5.0000 sec
PW1	2.95 usec
IRNUC	1H
CTEMP	20.8 c
SLVNT	CDCL3
EXREF	0.00 ppm
BF	0.10 Hz
RGAIN	54

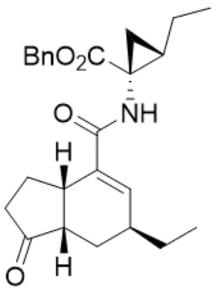

11d

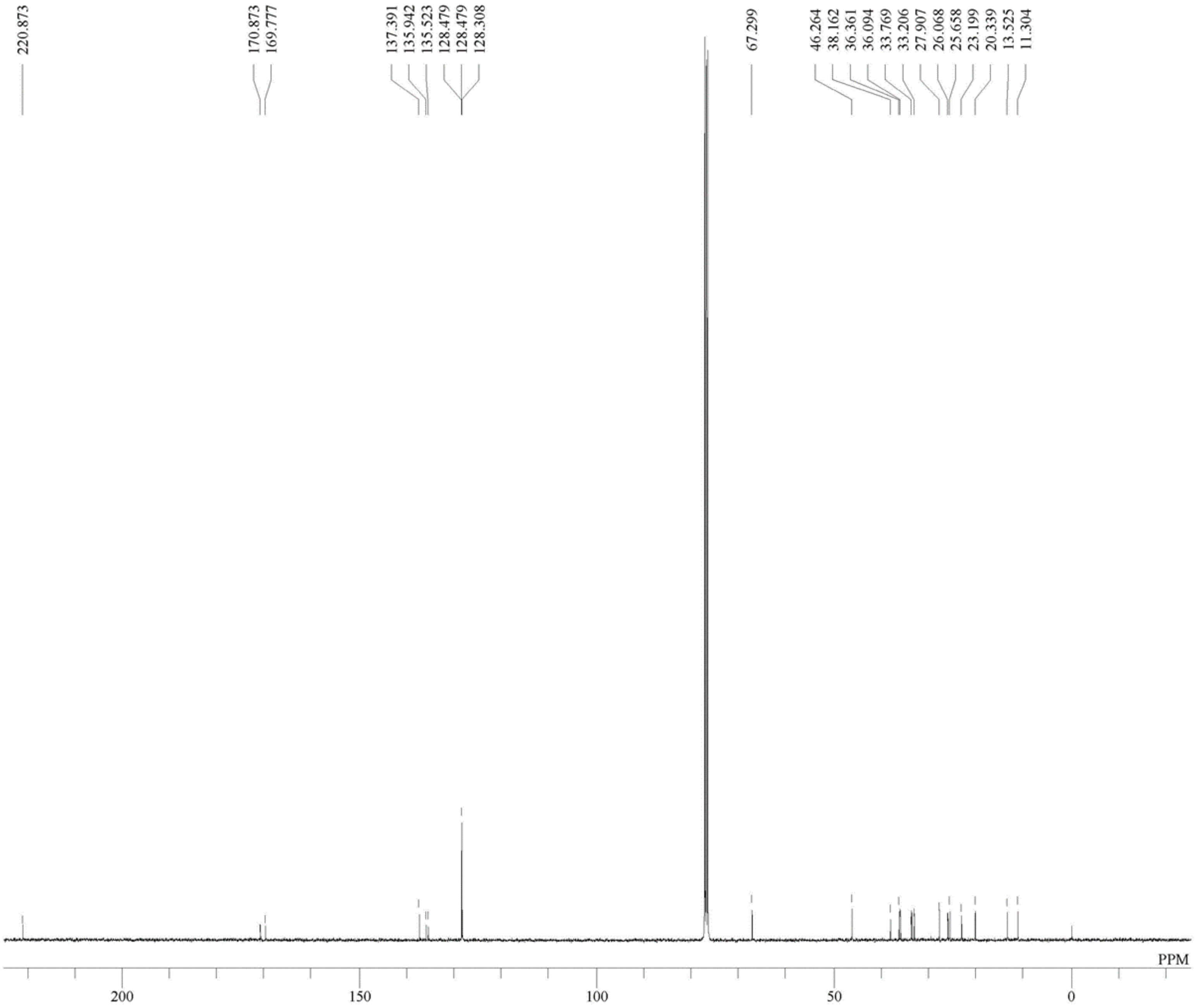

DFILE st_2_100_C6+nCMA-Bn_Carb
COMNT single pulse decoupled gated N
DATIM 06-06-2017 00:56:59
OBNUC 13C
EXMOD carbon.jxp
OBFRQ 100.53 MHz
OBSET 5.35 KHz
OBFIN 5.86 Hz
POINT 32780
FREQU 31407.04 Hz
SCANS 10699
ACQTM 1.0433 sec
PD 2.0000 sec
PW1 3.37 usec
IRNUC 1H
CTEMP 24.1 c
SLVNT CDCL3
EXREF 0.00 ppm
BF 0.10 Hz
RGAIN 50

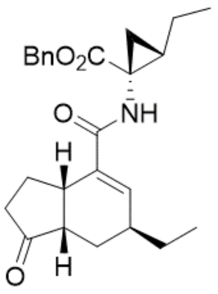

11d

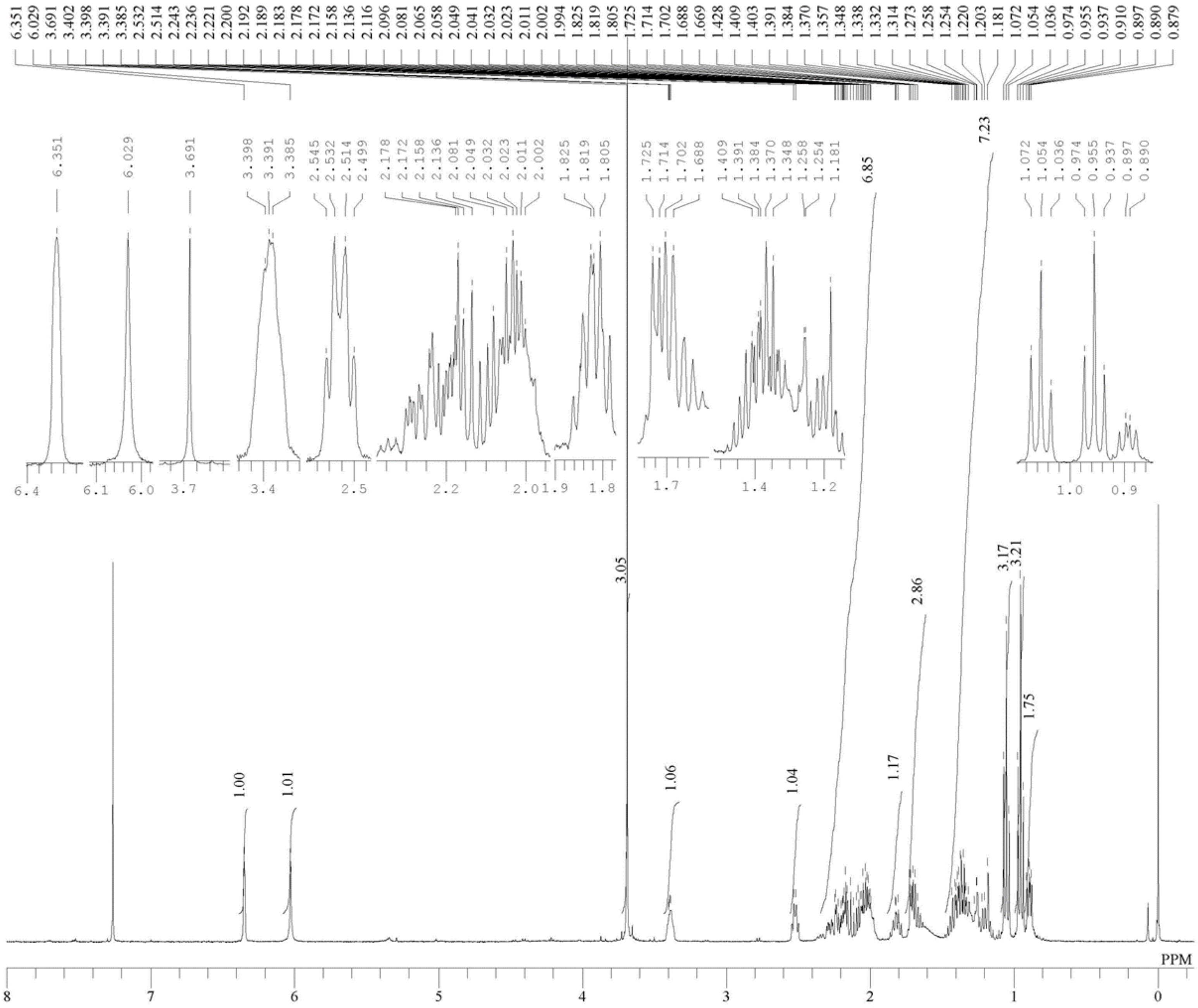

DFILE	3-144-1_Proton-1-1.als
COMNT	single_pulse
DATIM	12-12-2018 15:10:31
OBNUC	1H
EXMOD	proton.jxp
OBFRQ	399.78 MHz
OBSET	4.19 KHz
OBFIN	7.29 Hz
POINT	16384
FREQU	7503.00 Hz
SCANS	8
ACQTM	2.1837 sec
PD	5.0000 sec
PW1	2.95 usec
IRNUC	1H
CTEMP	20.4 c
SLVNT	CDCL3
EXREF	0.00 ppm
BF	0.10 Hz
RGAIN	60

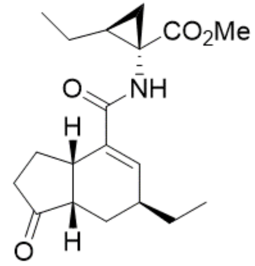

11e

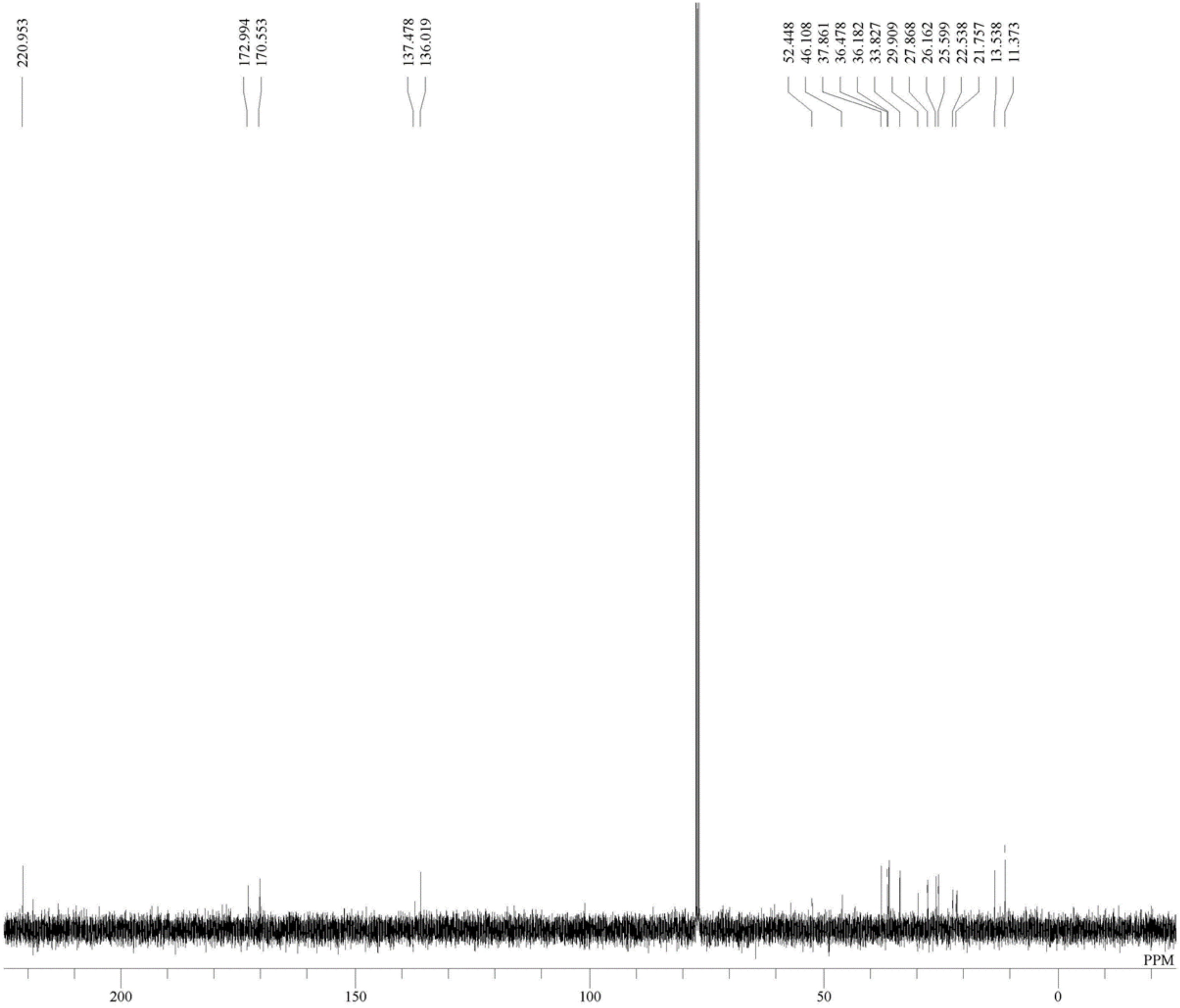

DFILE 3-144-1_Carbon-1-1.als
COMNT single pulse decoupled gated N
DATIM 12-12-2018 15:11:49
OBNUC 13C
EXMOD carbon.jxp
OBFRQ 100.53 MHz
OBSET 5.35 KHz
OBFIN 5.86 Hz
POINT 32767
FREQU 31407.04 Hz
SCANS 1024
ACQTM 1.0433 sec
PD 2.0000 sec
PW1 3.37 usec
IRNUC 1H
CTEMP 20.4 c
SLVNT CDCL3
EXREF 77.00 ppm
BF 0.10 Hz
RGAIN 50

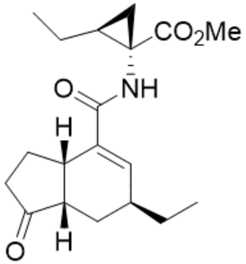

11e

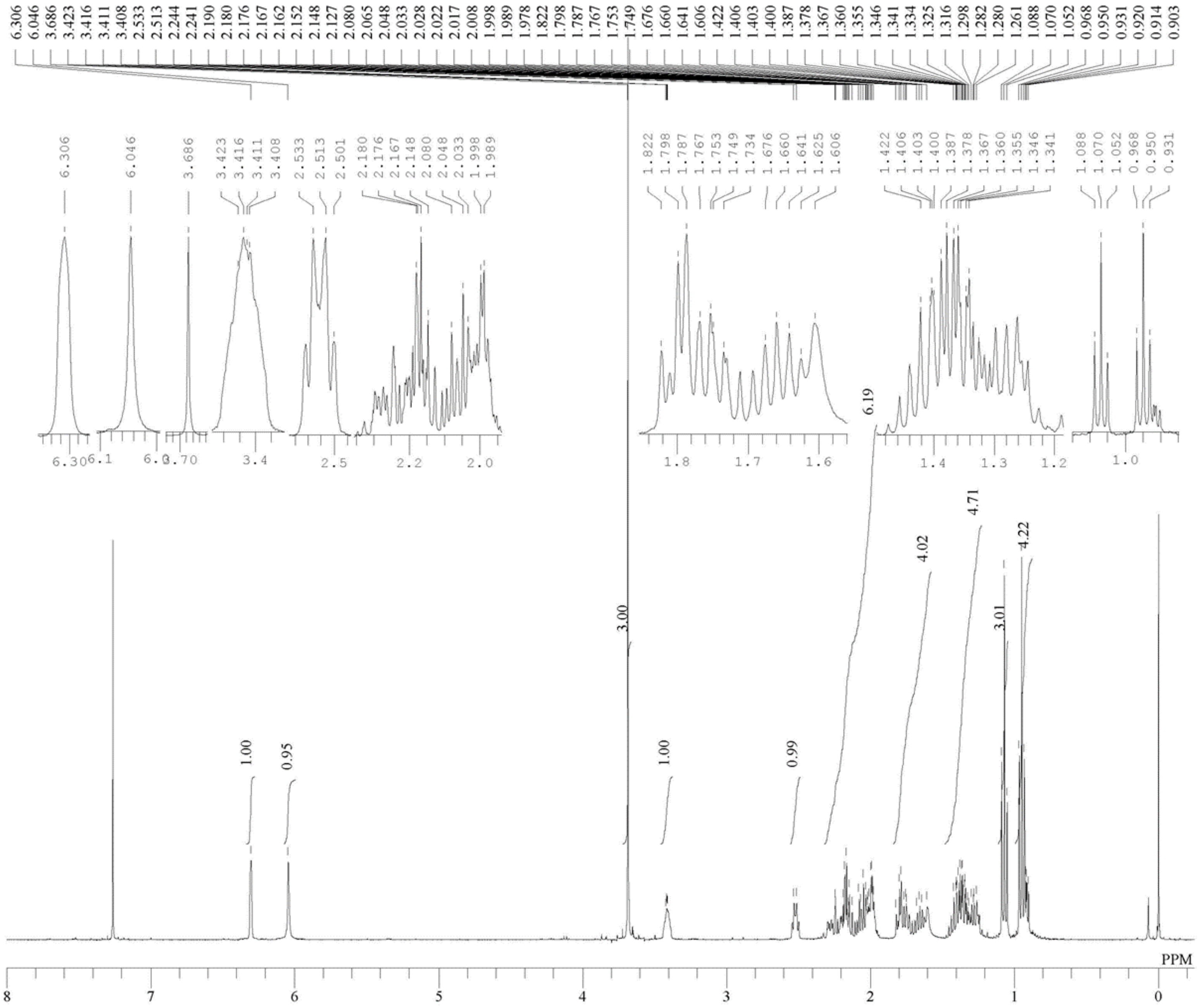

DFILE	3-144-2_Proton-1-1.als
COMNT	single_pulse
DATIM	12-12-2018 21:08:06
OBNUC	1H
EXMOD	proton.jxp
OBFRQ	399.78 MHz
OBSET	4.19 KHz
OBFIN	7.29 Hz
POINT	16384
FREQU	7503.00 Hz
SCANS	8
ACQTM	2.1837 sec
PD	5.0000 sec
PW1	2.95 usec
IRNUC	1H
CTEMP	20.3 c
SLVNT	CDCL3
EXREF	0.00 ppm
BF	0.10 Hz
RGAIN	56

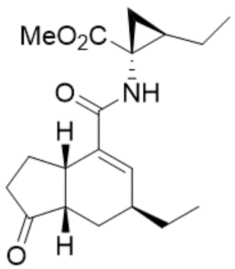

11f

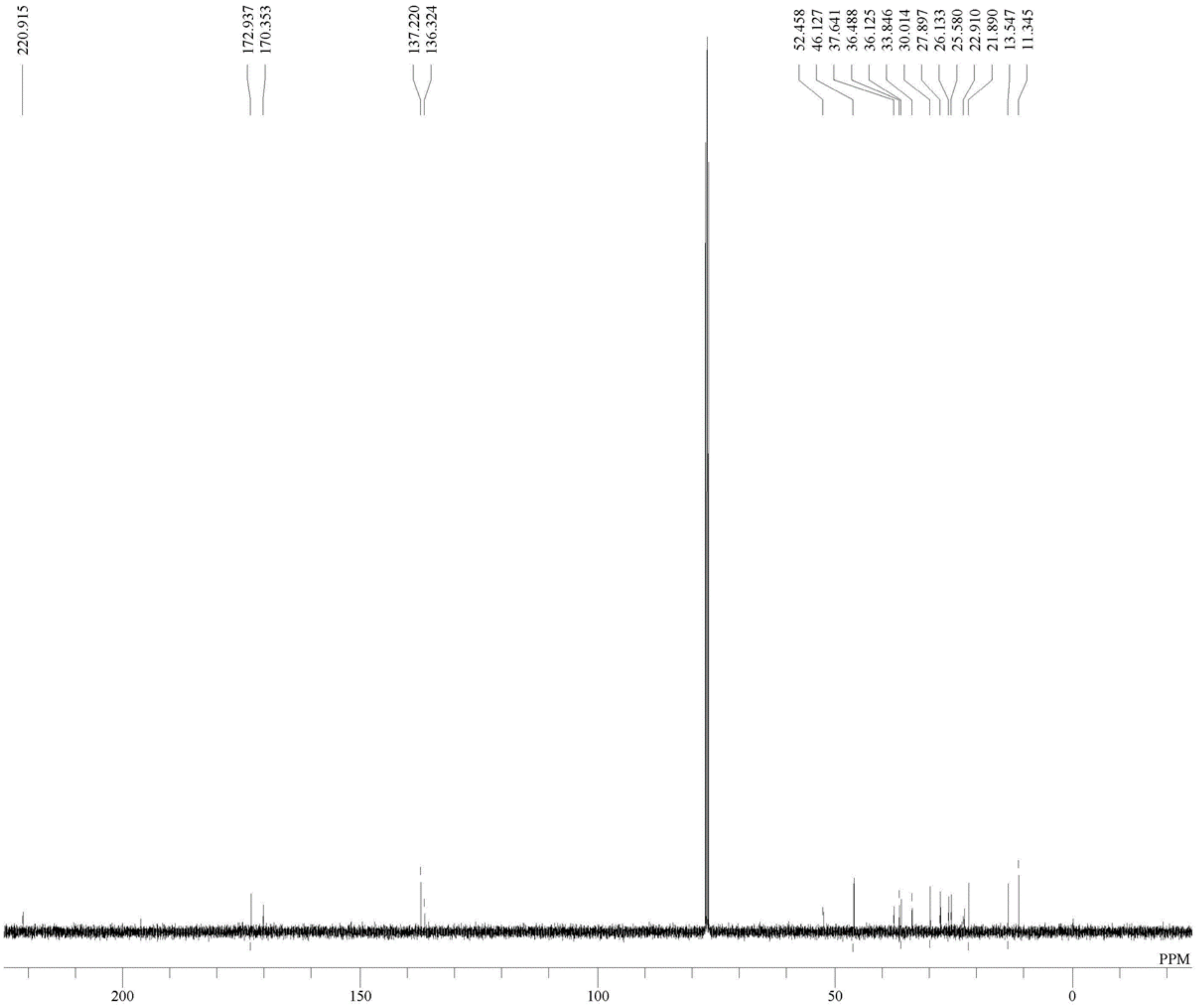

DFILE 3-144-2_Carbon-1-1.als
COMNT single pulse decoupled gated N
DATIM 12-12-2018 21:09:19
OBNUC 13C
EXMOD carbon.jxp
OBFRQ 100.53 MHz
OBSET 5.35 KHz
OBFIN 5.86 Hz
POINT 32767
FREQU 31407.04 Hz
SCANS 1184
ACQTM 1.0433 sec
PD 2.0000 sec
PW1 3.37 usec
IRNUC 1H
CTEMP 20.4 c
SLVNT CDCL3
EXREF 77.00 ppm
BF 0.10 Hz
RGAIN 50

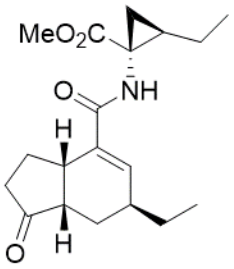

11f

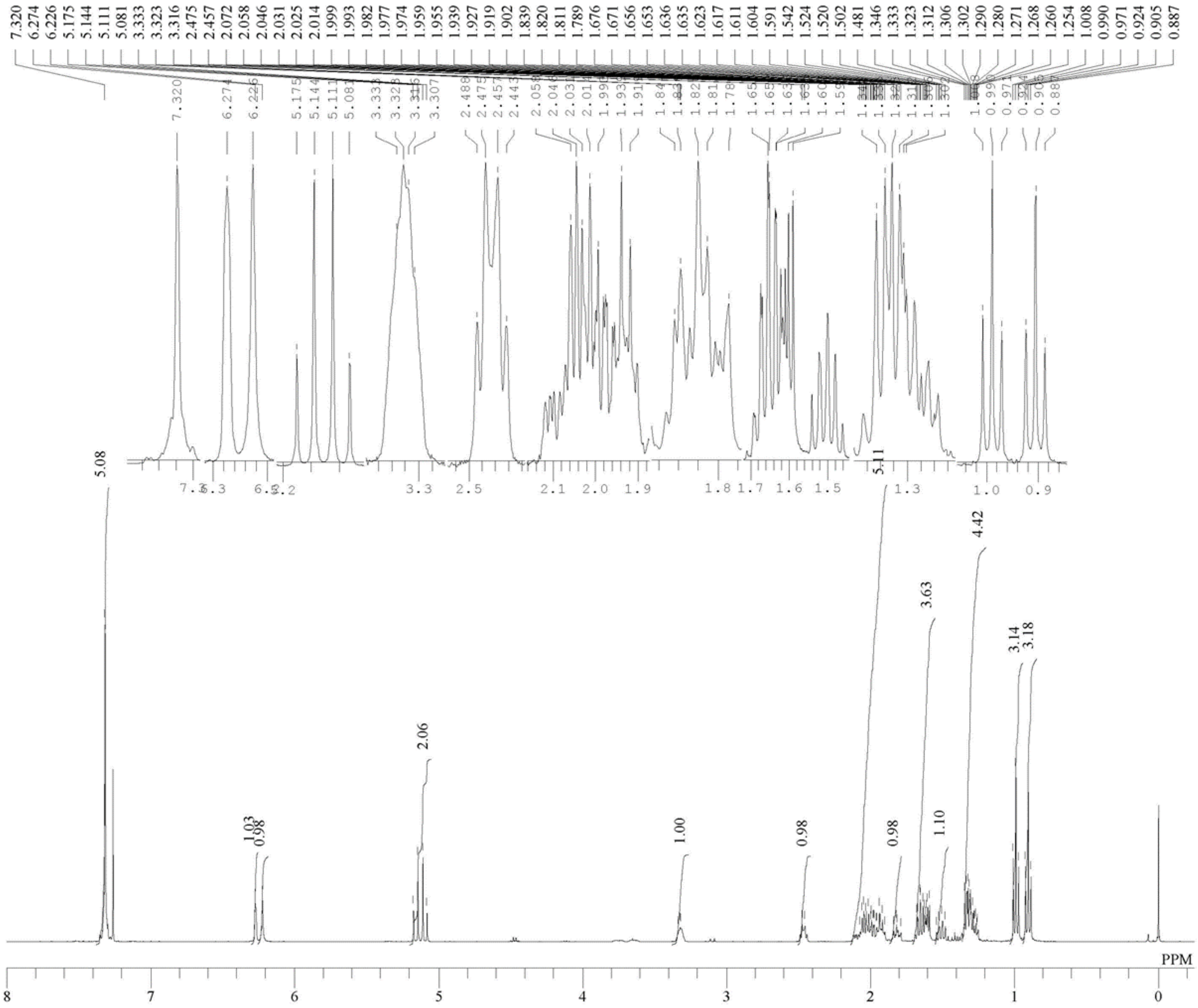

DFILE	3-138-6_Proton-1-1.als
COMNT	single_pulse
DATIM	29-11-2018 22:39:00
OBNUC	1H
EXMOD	proton.jxp
OBFRQ	399.78 MHz
OBSET	4.19 KHz
OBFIN	7.29 Hz
POINT	16384
FREQU	7503.00 Hz
SCANS	8
ACQTM	2.1837 sec
PD	5.0000 sec
PW1	2.95 usec
IRNUC	1H
CTEMP	22.1 c
SLVNT	CDCL3
EXREF	0.00 ppm
BF	0.10 Hz
RGAIN	56

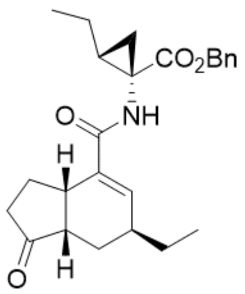

11g

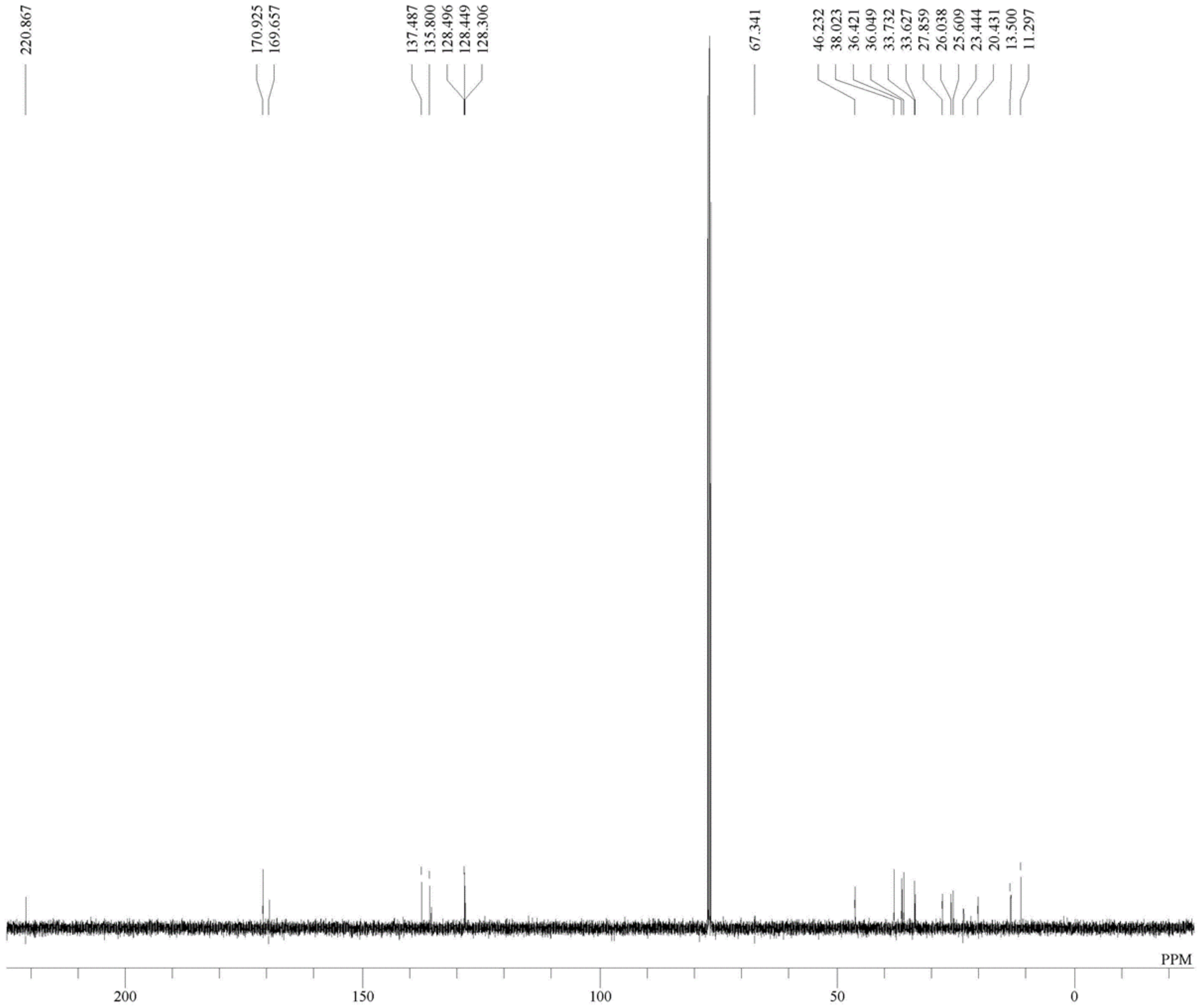

DFILE 3-138-6_Carbon-1-1.als
COMNT single pulse decoupled gated N
DATIM 29-11-2018 22:40:14
OBNUC 13C
EXMOD carbon.jxp
OBFRQ 100.53 MHz
OBSET 5.35 KHz
OBFIN 5.86 Hz
POINT 32767
FREQU 31407.04 Hz
SCANS 1024
ACQTM 1.0433 sec
PD 2.0000 sec
PW1 3.37 usec
IRNUC 1H
CTEMP 21.9 c
SLVNT CDCL3
EXREF 77.00 ppm
BF 0.10 Hz
RGAIN 50

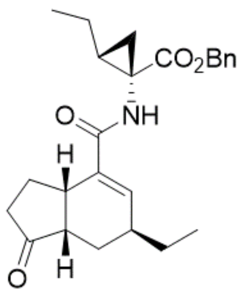

11g

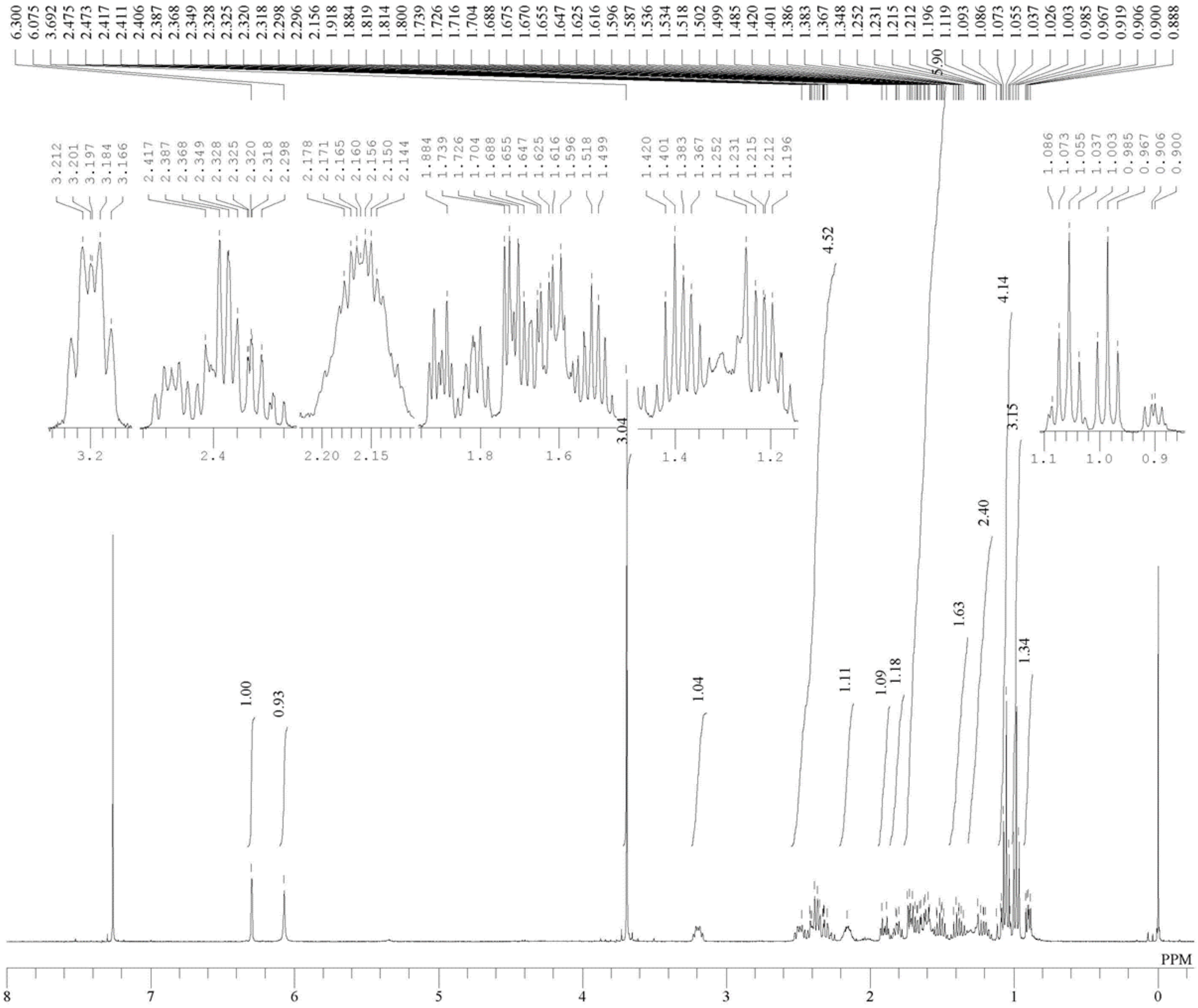

DFILE	3-156-1_Proton-1-1.jdf
COMNT	single_pulse
DATIM	04-01-2019 18:17:33
OBNUC	1H
EXMOD	proton.jxp
OBFRQ	399.78 MHz
OBSET	4.19 KHz
OBFIN	7.29 Hz
POINT	16384
FREQU	7503.00 Hz
SCANS	8
ACQTM	2.1837 sec
PD	5.0000 sec
PW1	2.95 usec
IRNUC	1H
CTEMP	17.6 c
SLVNT	CDCL3
EXREF	0.00 ppm
BF	0.10 Hz
RGAIN	60

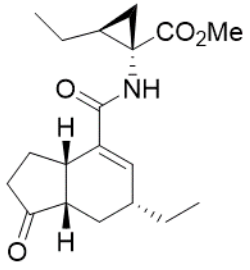

11h

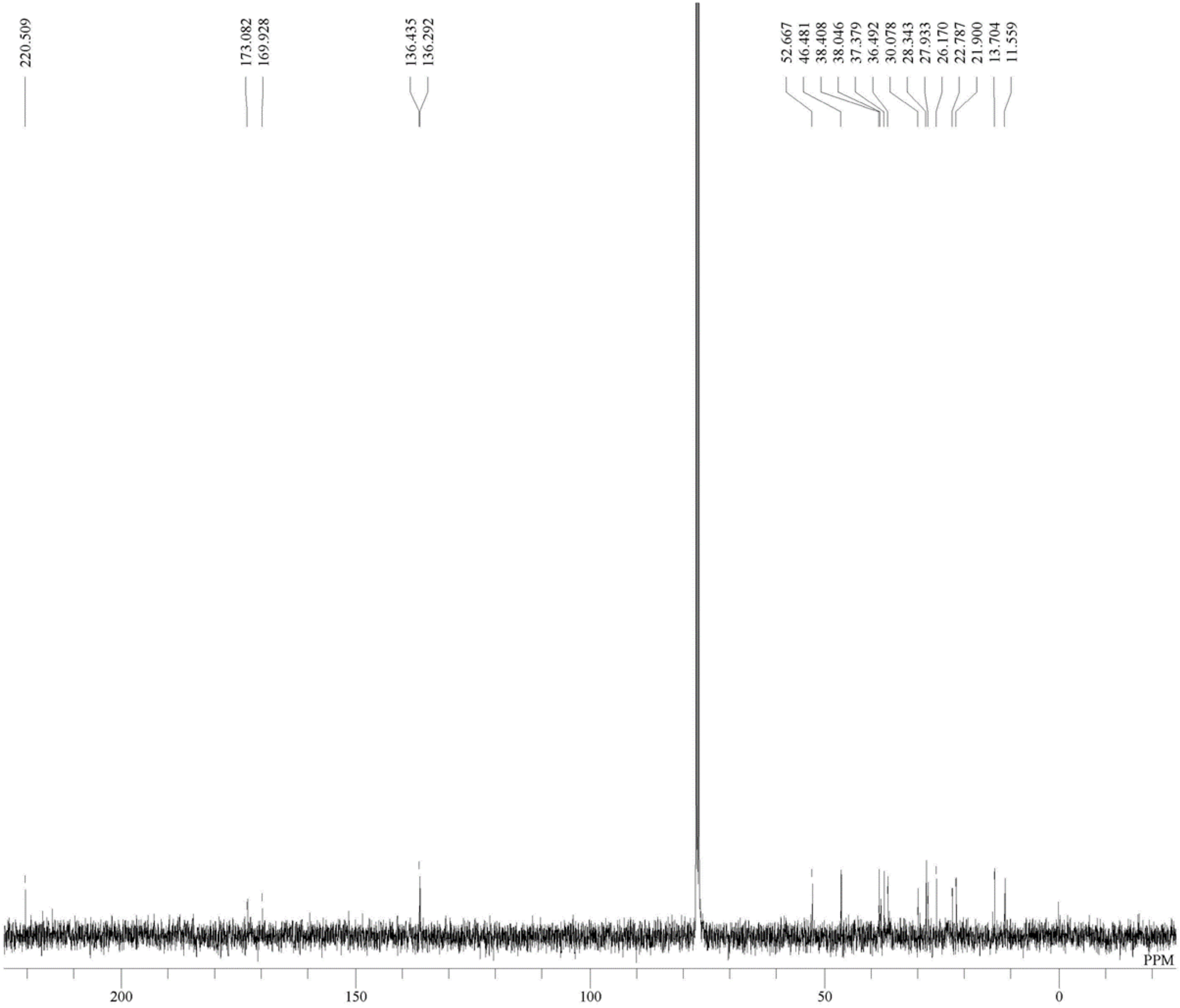

DFILE 3-156-1_Carbon-1-1.als
COMNT single pulse decoupled gated N
DATIM 04-01-2019 18:18:46
OBNUC 13C
EXMOD carbon.jxp
OBFRQ 100.53 MHz
OBSET 5.35 KHz
OBFIN 5.86 Hz
POINT 26224
FREQU 25125.63 Hz
SCANS 884
ACQTM 1.0433 sec
PD 2.0000 sec
PW1 3.37 usec
IRNUC 1H
CTEMP 17.6 c
SLVNT CDCL3
EXREF 224.97 ppm
BF 0.10 Hz
RGAIN 50

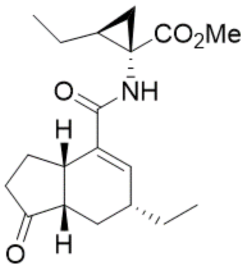

11h

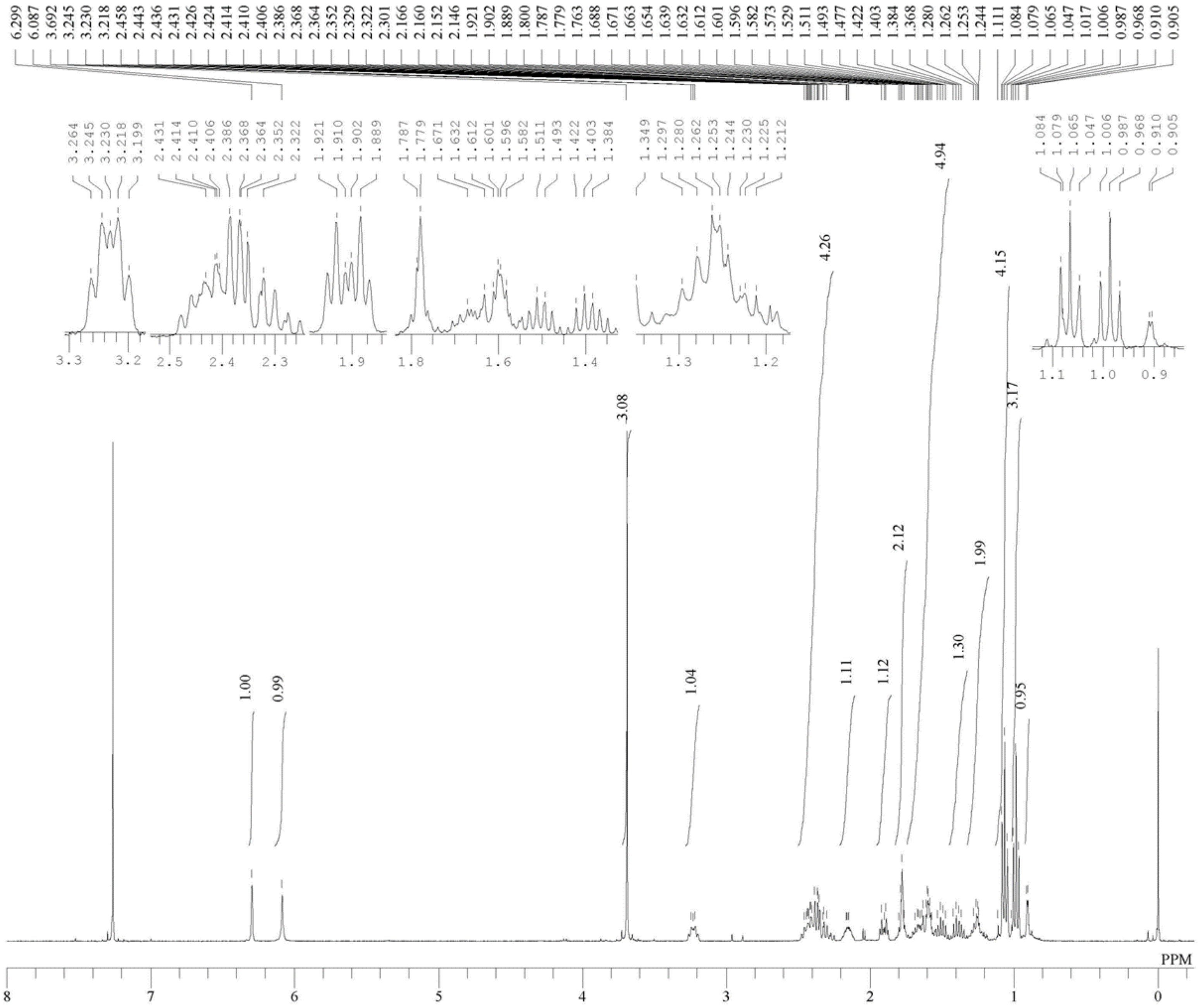

DFILE	3-156-2_Proton-1-1.jdf
COMNT	single_pulse
DATIM	04-01-2019 22:53:60
OBNUC	1H
EXMOD	proton.jxp
OBFRQ	399.78 MHz
OBSET	4.19 KHz
OBFIN	7.29 Hz
POINT	16384
FREQU	7503.00 Hz
SCANS	8
ACQTM	2.1837 sec
PD	5.0000 sec
PW1	2.95 usec
IRNUC	1H
CTEMP	17.5 c
SLVNT	CDCL3
EXREF	0.00 ppm
BF	0.10 Hz
RGAIN	60

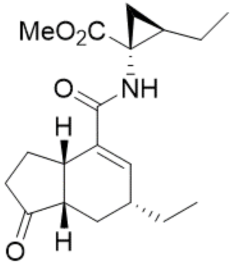

11i

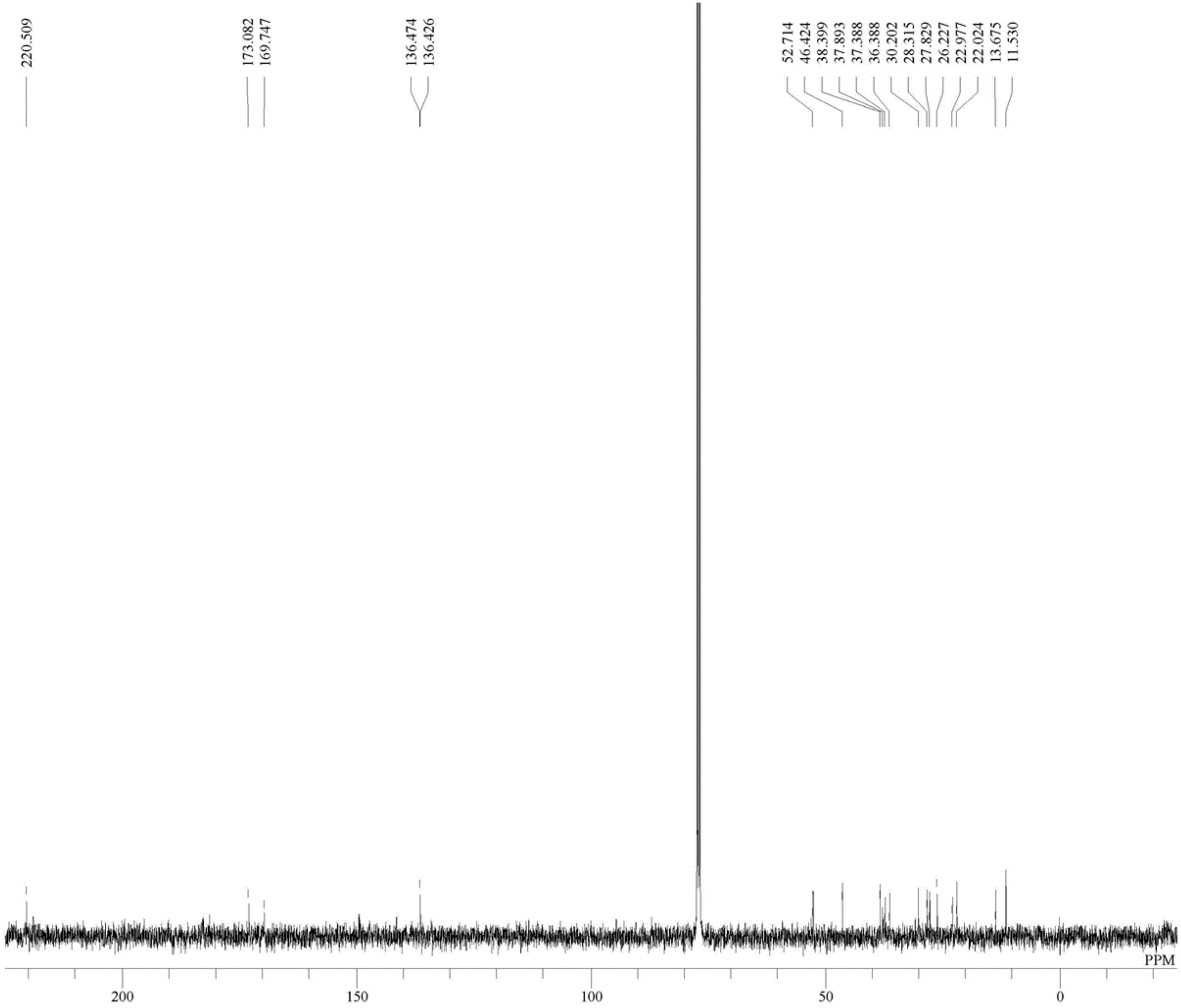

DFILE	3-156-2_Carbon-1-1.als
COMNT	single pulse decoupled gated N
DATIM	04-01-2019 19:10:15
OBNUC	13C
EXMOD	carbon.jxp
OBFRQ	100.53 MHz
OBSET	5.35 KHz
OBFIN	5.86 Hz
POINT	32780
FREQU	31407.04 Hz
SCANS	1056
ACQTM	1.0433 sec
PD	2.0000 sec
PW1	3.37 usec
IRNUC	1H
CTEMP	17.6 c
SLVNT	CDCL3
EXREF	224.97 ppm
BF	0.10 Hz
RGAIN	50

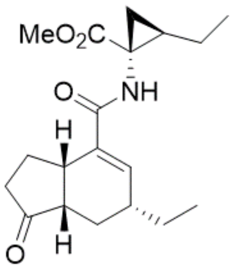

11i

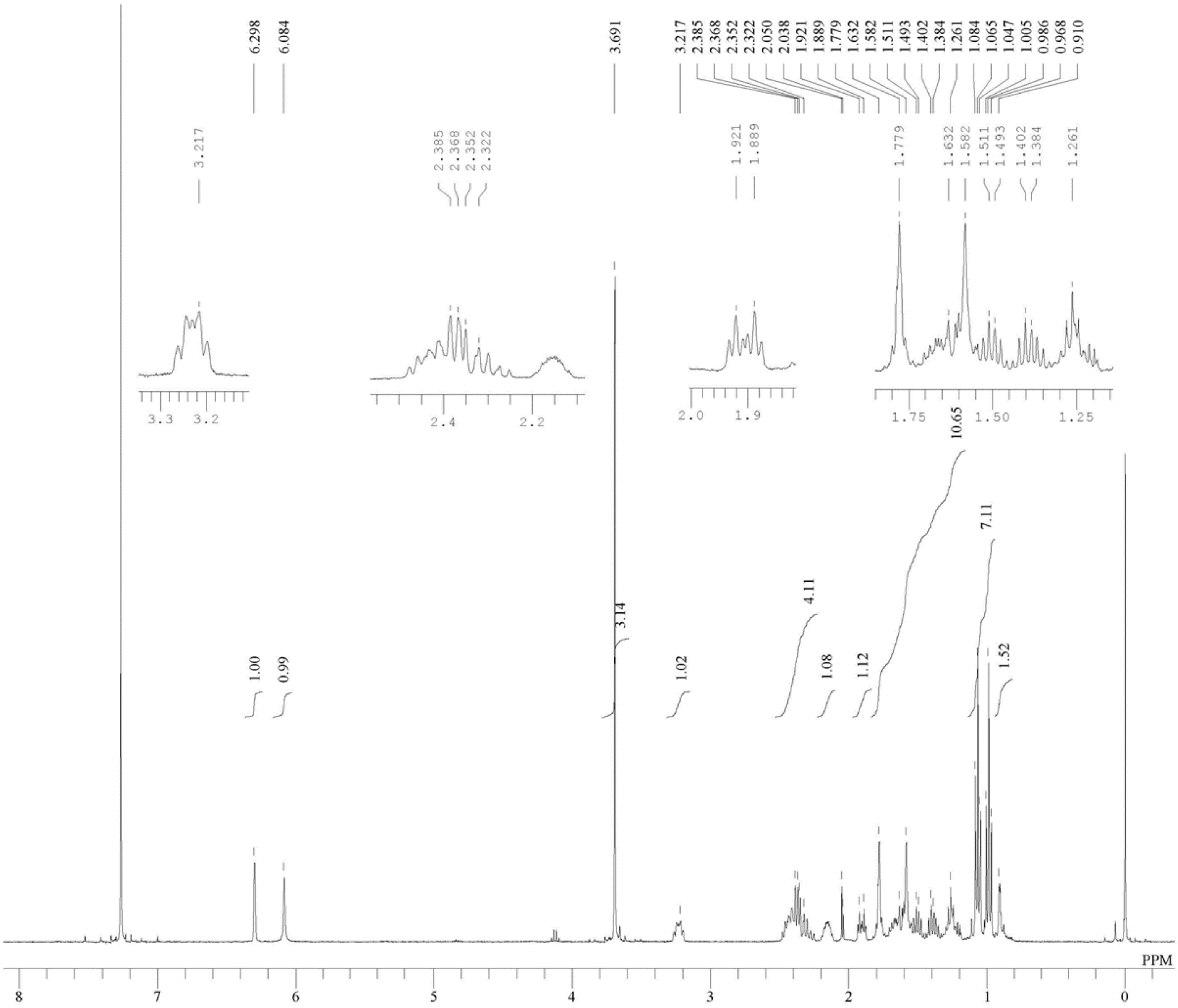

DFILE	3-156-3_Proton-1-1.jdf
COMNT	single_pulse
DATIM	04-01-2019 23:00:40
OBNUC	1H
EXMOD	proton.jxp
OBFRQ	399.78 MHz
OBSET	4.19 KHz
OBFIN	7.29 Hz
POINT	16384
FREQU	7503.00 Hz
SCANS	8
ACQTM	2.1837 sec
PD	5.0000 sec
PW1	2.95 usec
IRNUC	1H
CTEMP	17.4 c
SLVNT	CDCL3
EXREF	0.00 ppm
BF	0.25 Hz
RGAIN	62

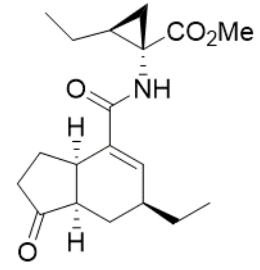

11j

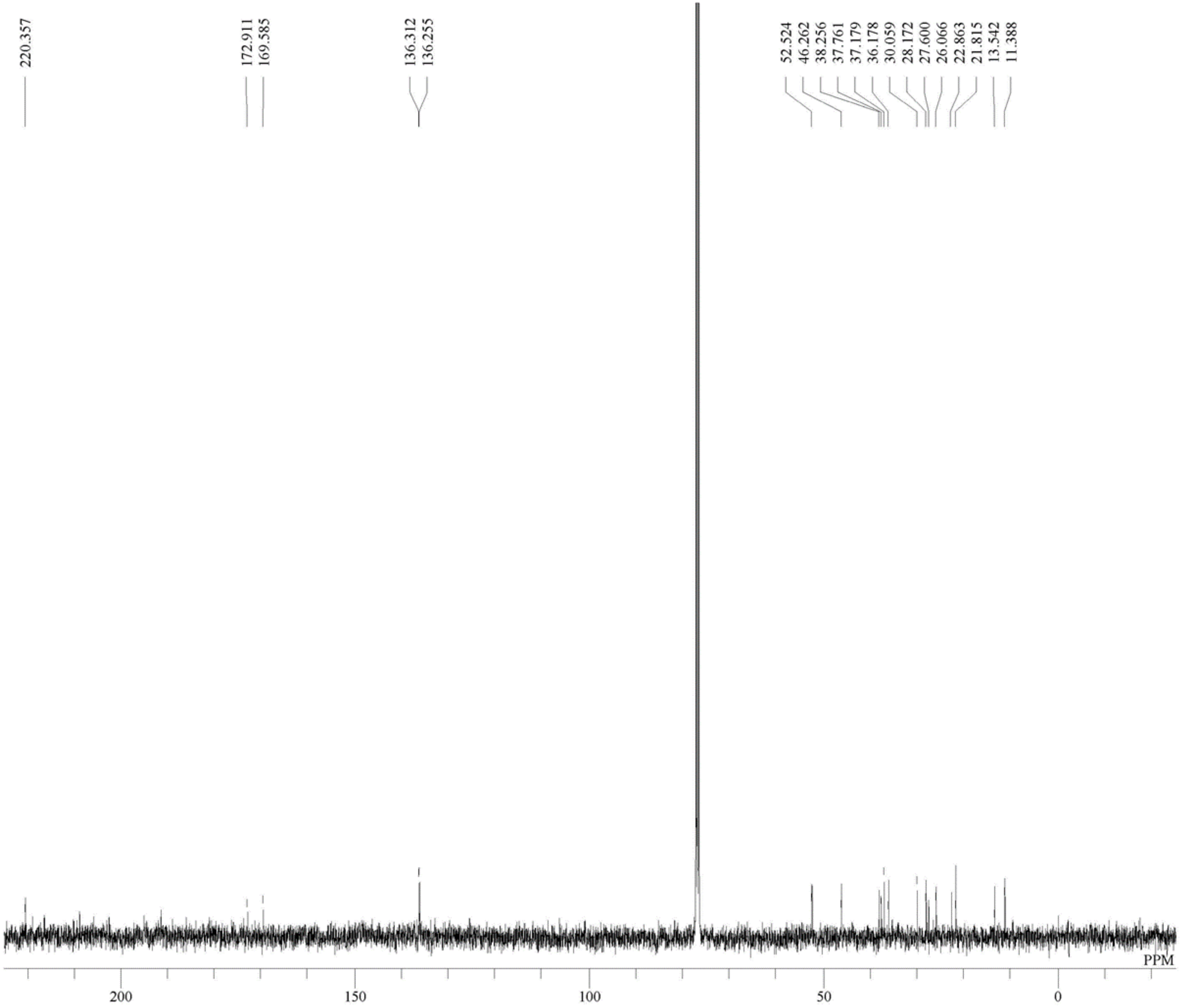

DFILE	3-156-3_Carbon-1-1.jdf
COMNT	single pulse decoupled gated N
DATIM	04-01-2019 20:11:14
OBNUC	13C
EXMOD	carbon.jxp
OBFRQ	100.53 MHz
OBSET	5.35 KHz
OBFIN	5.86 Hz
POINT	32780
FREQU	31407.04 Hz
SCANS	1091
ACQTM	1.0433 sec
PD	2.0000 sec
PW1	3.37 usec
IRNUC	1H
CTEMP	17.5 c
SLVNT	CDCL3
EXREF	77.00 ppm
BF	0.10 Hz
RGAIN	50

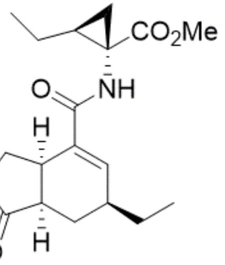

11j

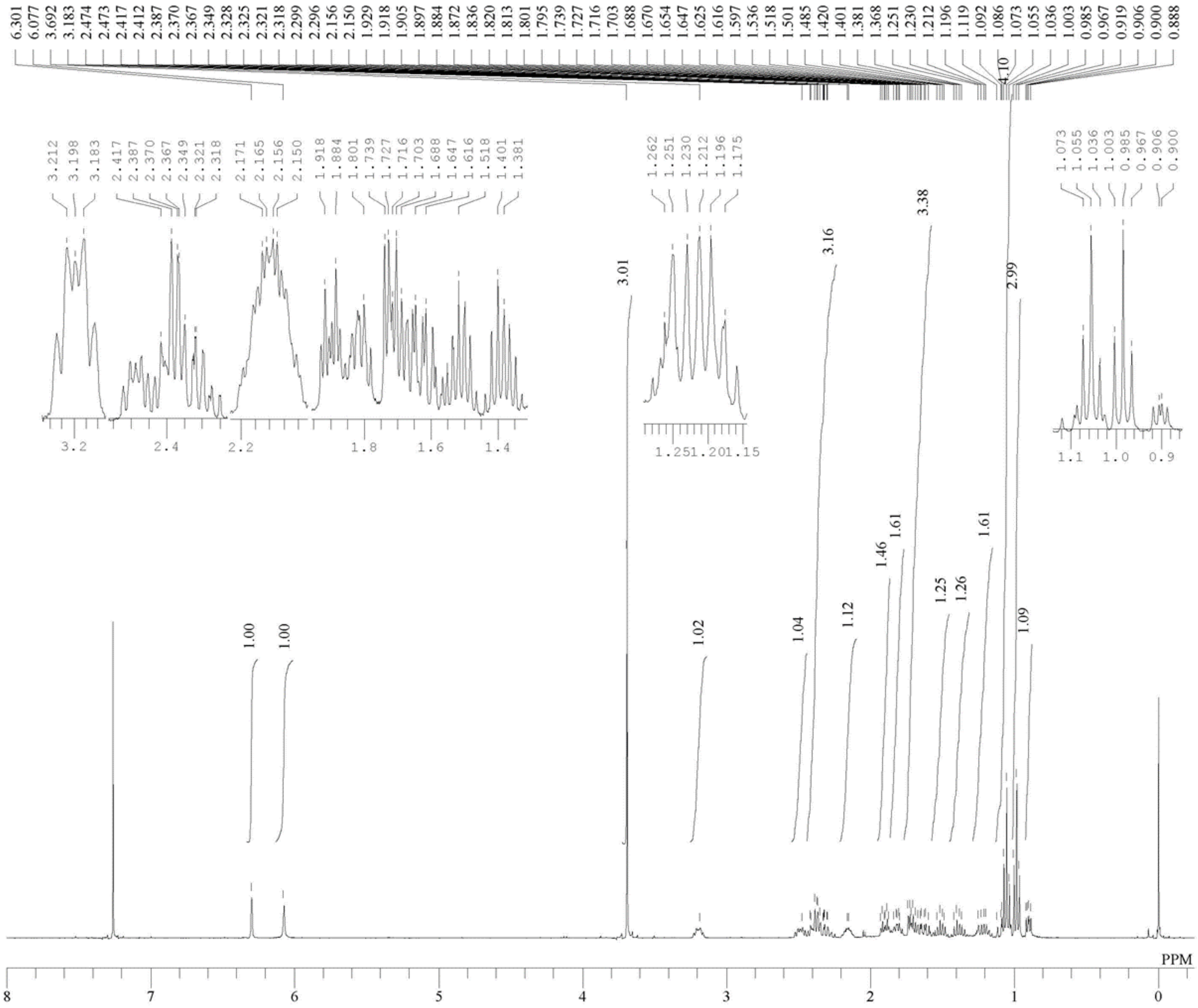

DFILE	3-156-4_Proton-1-1.jdf
COMNT	single_pulse
DATIM	04-01-2019 21:11:49
OBNUC	1H
EXMOD	proton.jxp
OBFRQ	399.78 MHz
OBSET	4.19 KHz
OBFIN	7.29 Hz
POINT	16384
FREQU	7503.00 Hz
SCANS	8
ACQTM	2.1837 sec
PD	5.0000 sec
PW1	2.95 usec
IRNUC	1H
CTEMP	17.4 c
SLVNT	CDCL3
EXREF	0.00 ppm
BF	0.10 Hz
RGAIN	62

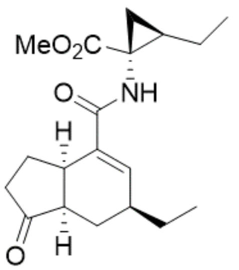

11k

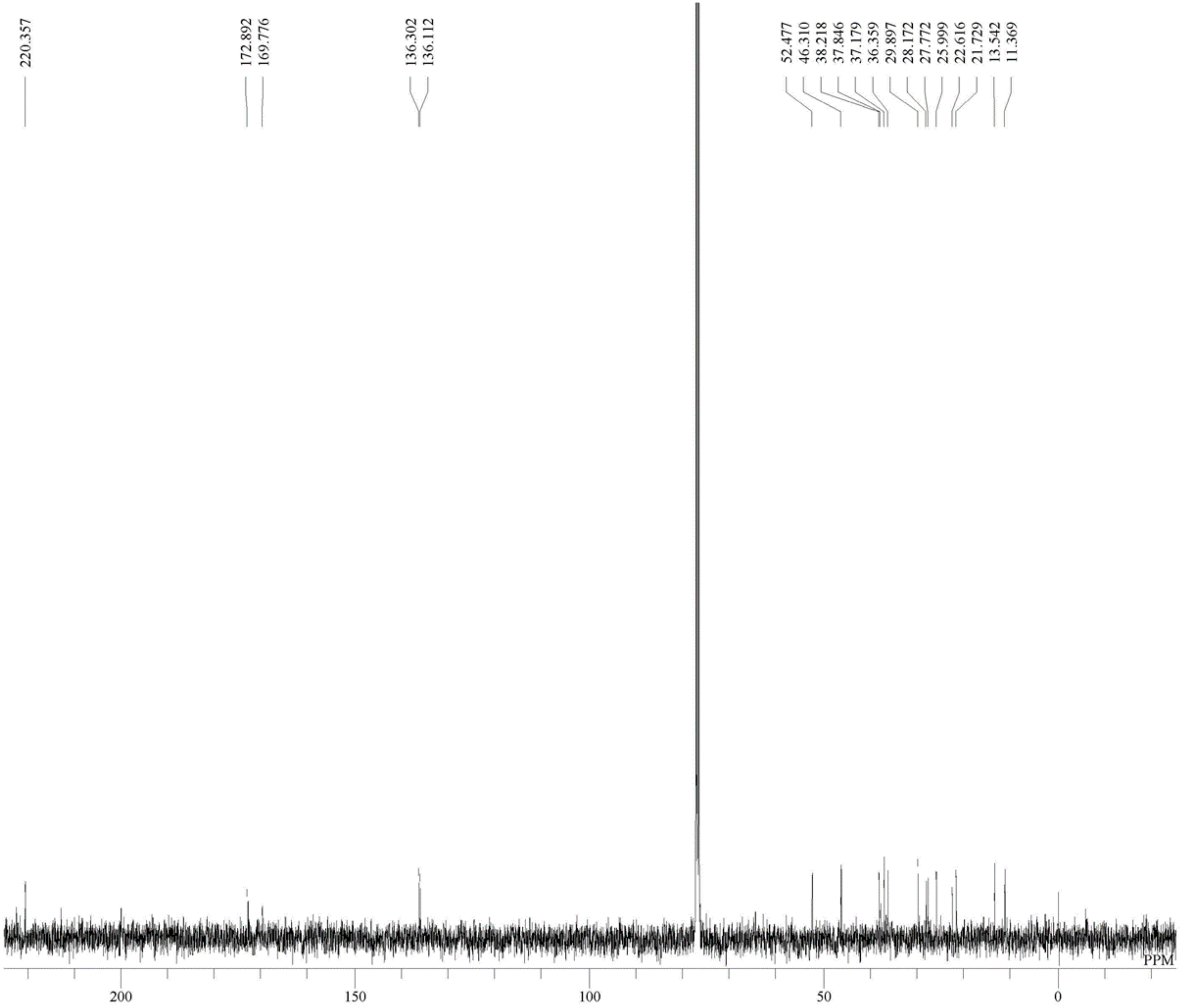

DFILE	3-156-4_Carbon-1-1.jdf
COMNT	single pulse decoupled gated N
DATIM	04-01-2019 21:13:02
OBNUC	13C
EXMOD	carbon.jxp
OBFRQ	100.53 MHz
OBSET	5.35 KHz
OBFIN	5.86 Hz
POINT	32780
FREQU	31407.04 Hz
SCANS	1337
ACQTM	1.0433 sec
PD	2.0000 sec
PW1	3.37 usec
IRNUC	1H
CTEMP	17.4 c
SLVNT	CDCL3
EXREF	77.00 ppm
BF	0.10 Hz
RGAIN	50

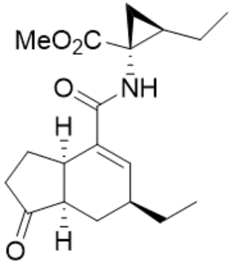

11k

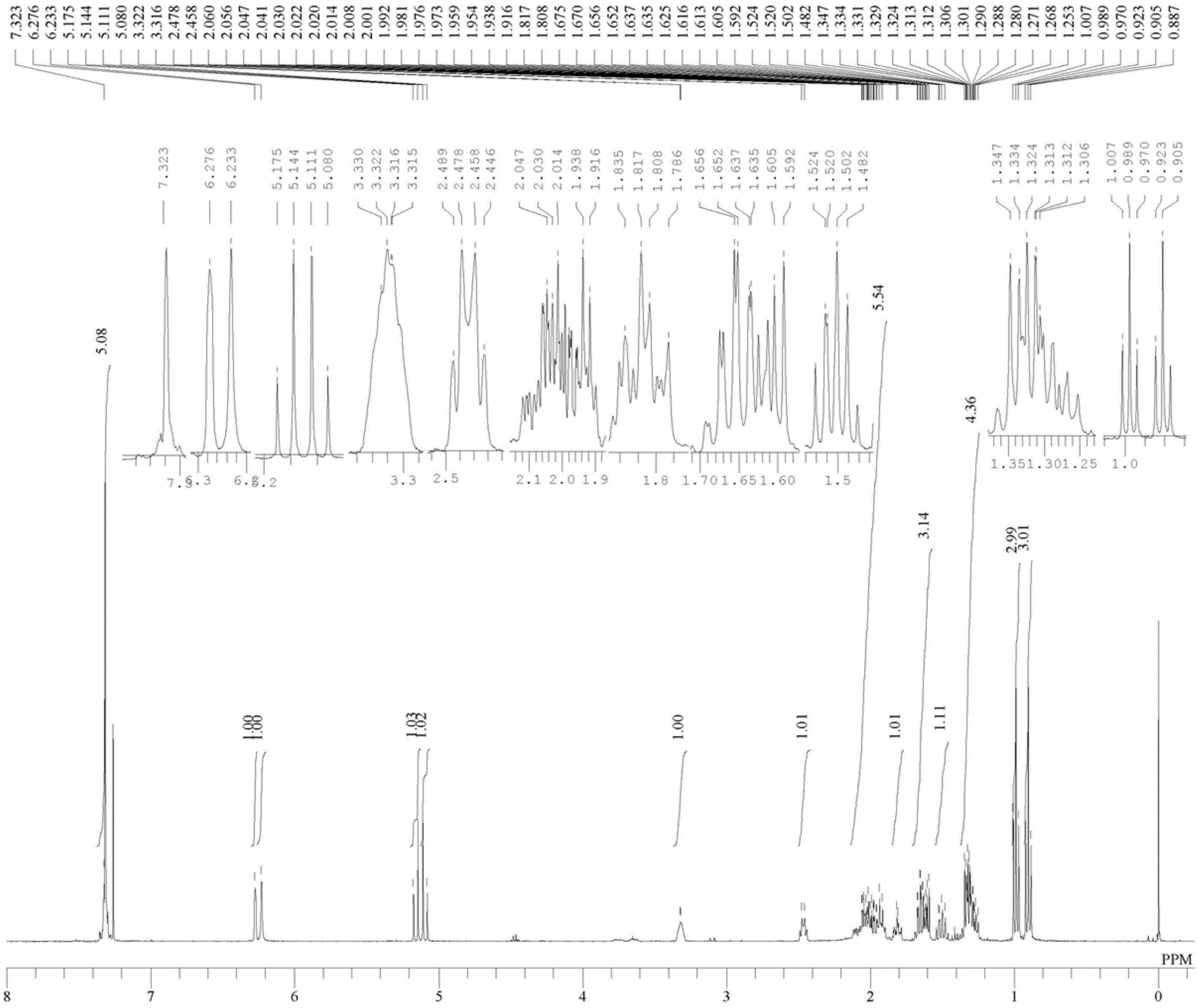

DFILE	3-150-4_Proton-1-1.als
COMNT	single_pulse
DATIM	21-12-2018 19:30:39
OBNUC	1H
EXMOD	proton.jxp
OBFRQ	399.78 MHz
OBSET	4.19 KHz
OBFIN	7.29 Hz
POINT	16384
FREQU	7503.00 Hz
SCANS	8
ACQTM	2.1837 sec
PD	5.0000 sec
PW1	2.95 usec
IRNUC	1H
CTEMP	20.8 c
SLVNT	CDCL3
EXREF	0.00 ppm
BF	0.10 Hz
RGAIN	56

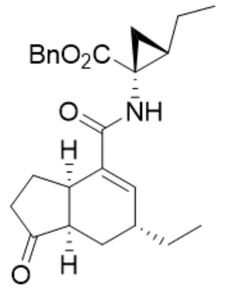

11l

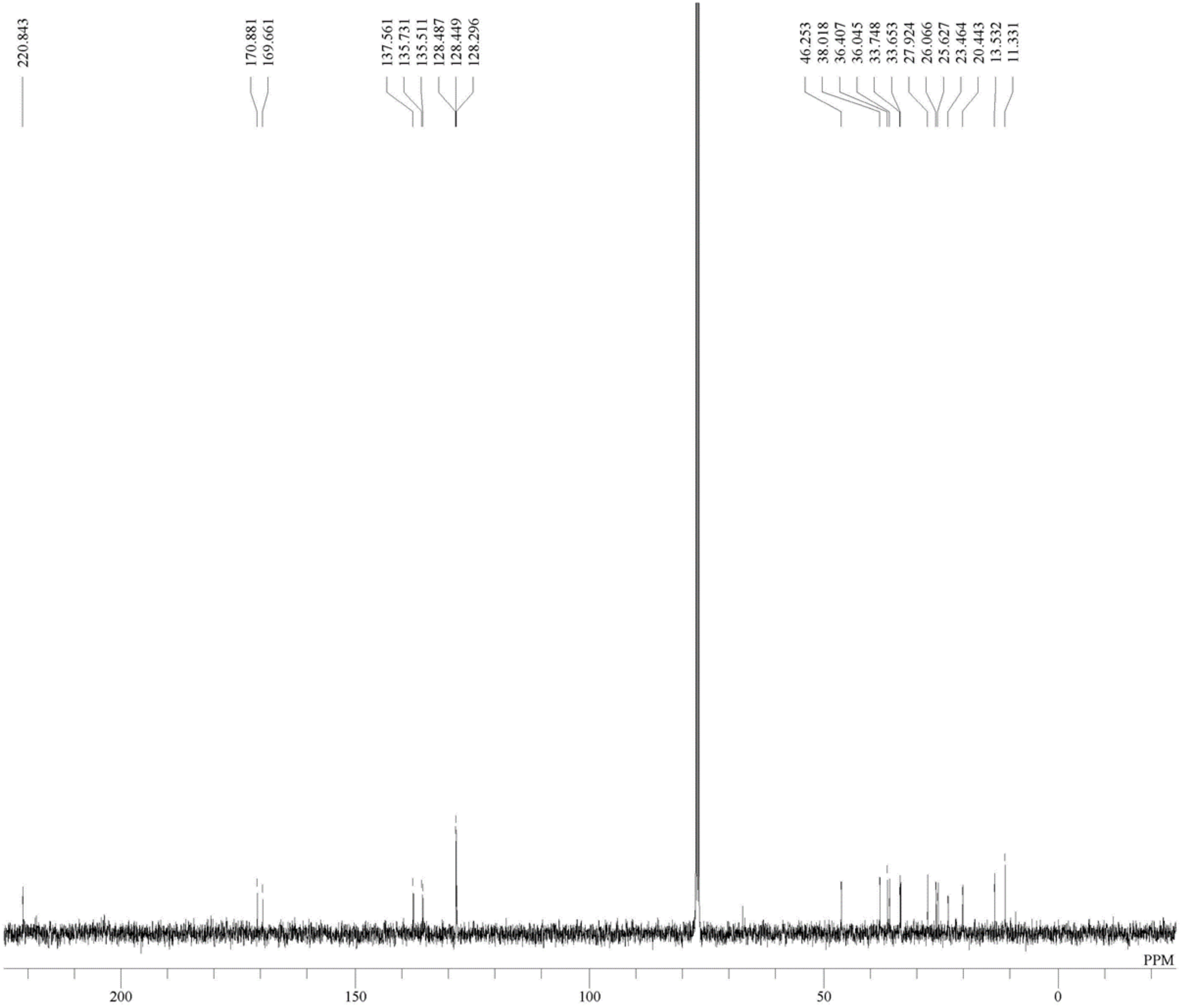

DFILE 3-150-4_Carbon-1-1.jdf
COMNT single pulse decoupled gated N[illegible]
DATIM 21-12-2018 20:25:42
OBNUC 13C
EXMOD carbon.jxp
OBFRQ 100.53 MHz
OBSET 5.35 KHz
OBFIN 5.86 Hz
POINT 32780
FREQU 31407.04 Hz
SCANS 712
ACQTM 1.0433 sec
PD 2.0000 sec
PW1 3.37 usec
IRNUC 1H
CTEMP 20.9 c
SLVNT CDCL3
EXREF 77.00 ppm
BF 0.10 Hz
RGAIN 50

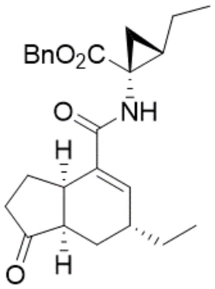

11l

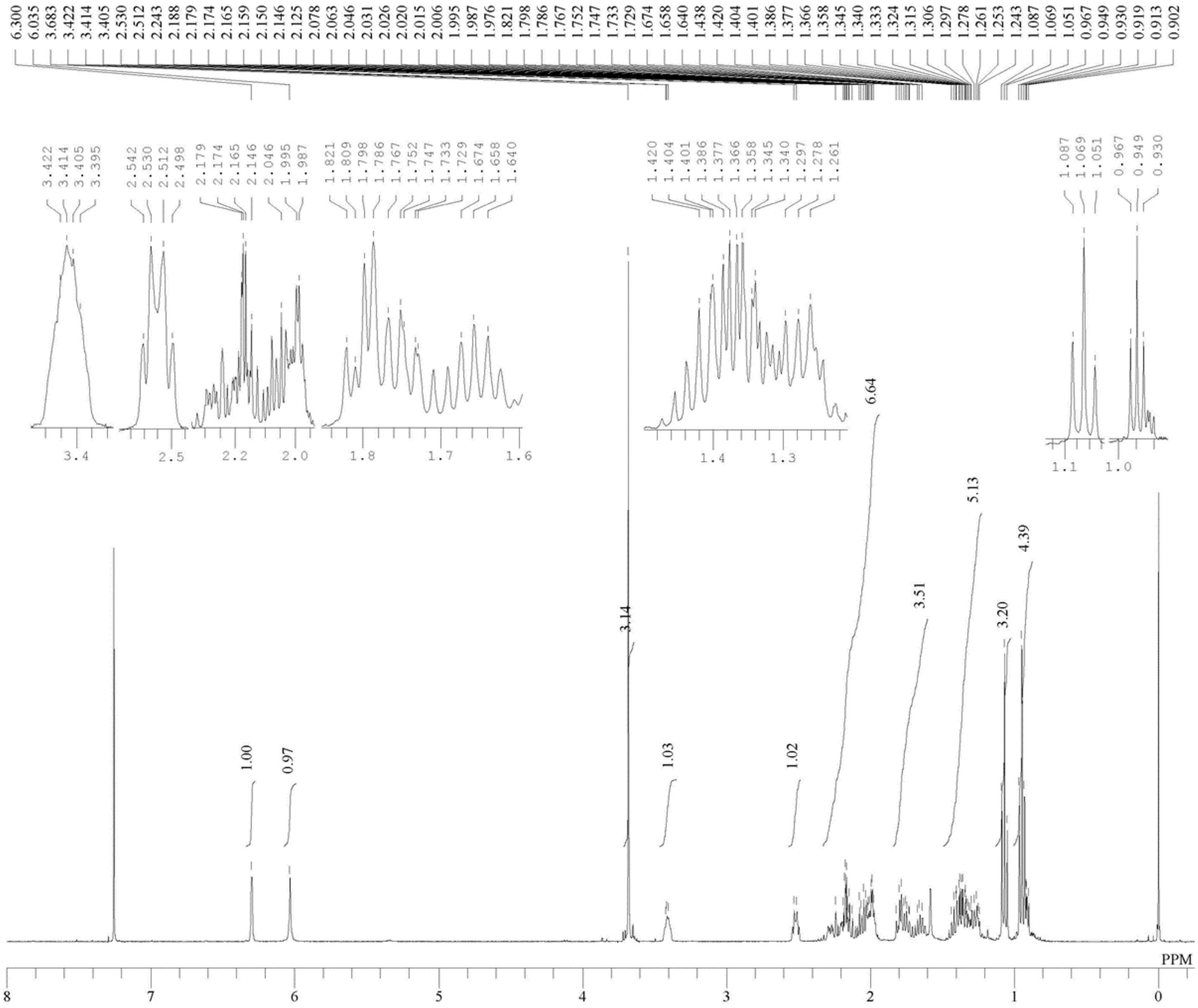

DFILE rw_2_192_Proton-3-1.jdf
COMNT single_pulse
DATIM 13-12-2018 01:58:33
OBNUC 1H
EXMOD proton.jxp
OBFRQ 399.78 MHz
OBSET 4.19 KHz
OBFIN 7.29 Hz
POINT 16400
FREQU 7503.00 Hz
SCANS 8
ACQTM 2.1837 sec
PD 5.0000 sec
PW1 2.95 usec
IRNUC 1H
CTEMP 19.7 c
SLVNT CDCL3
EXREF 0.00 ppm
BF 0.10 Hz
RGAIN 58

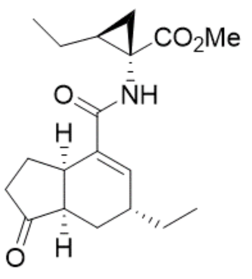

11m

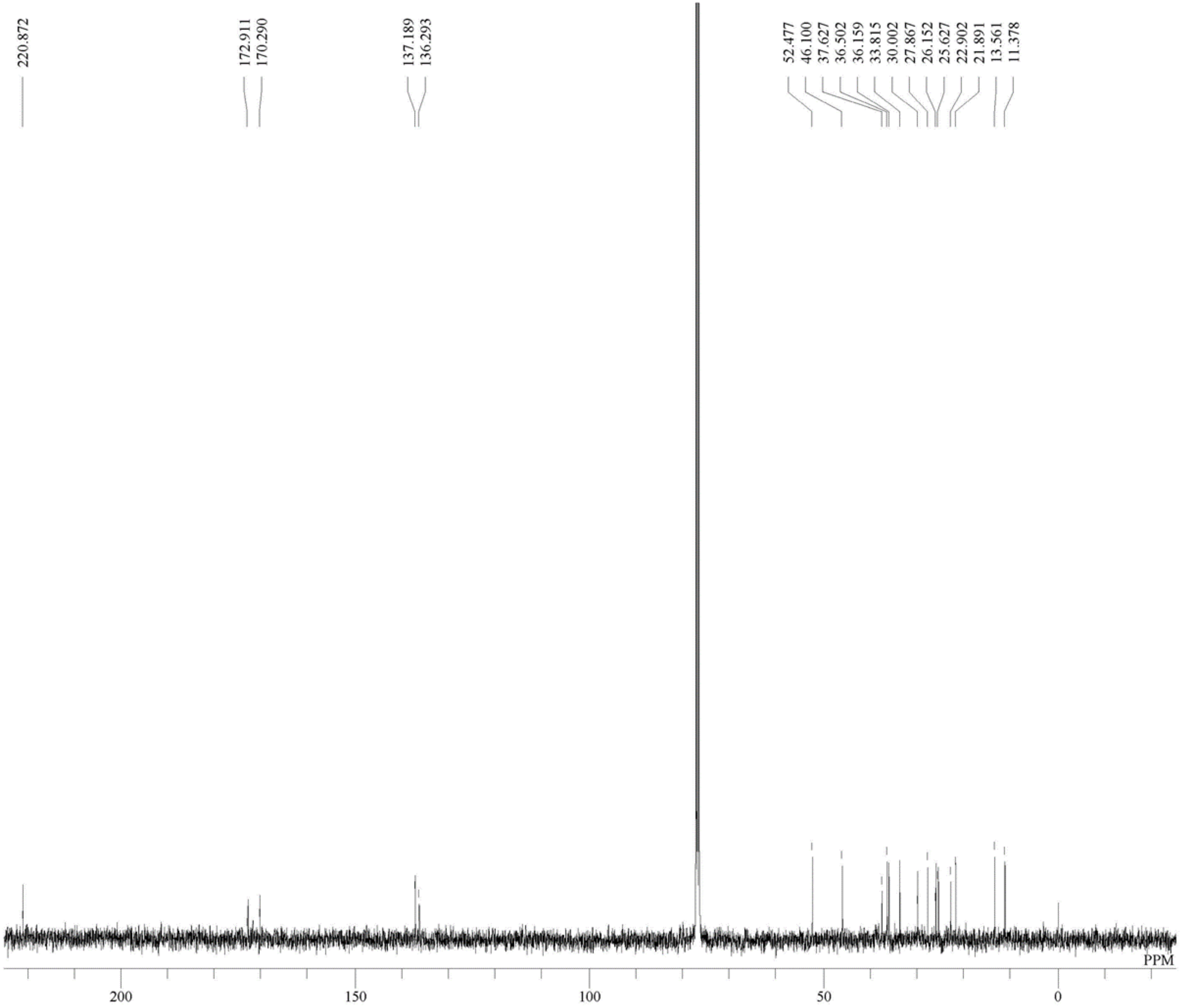

DFILE rw_2_192_Carbon-1-1.als
COMNT single pulse decoupled gated N
DATIM 13-12-2018 02:01:15
OBNUC 13C
EXMOD carbon.jxp
OBFRQ 100.53 MHz
OBSET 5.35 KHz
OBFIN 5.86 Hz
POINT 32780
FREQU 31407.04 Hz
SCANS 2048
ACQTM 1.0433 sec
PD 2.0000 sec
PW1 3.37 usec
IRNUC 1H
CTEMP 19.6 c
SLVNT CDCL3
EXREF 77.00 ppm
BF 0.10 Hz
RGAIN 50

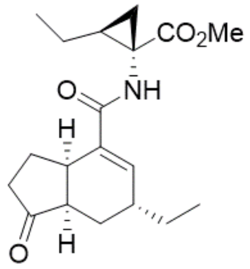

11m

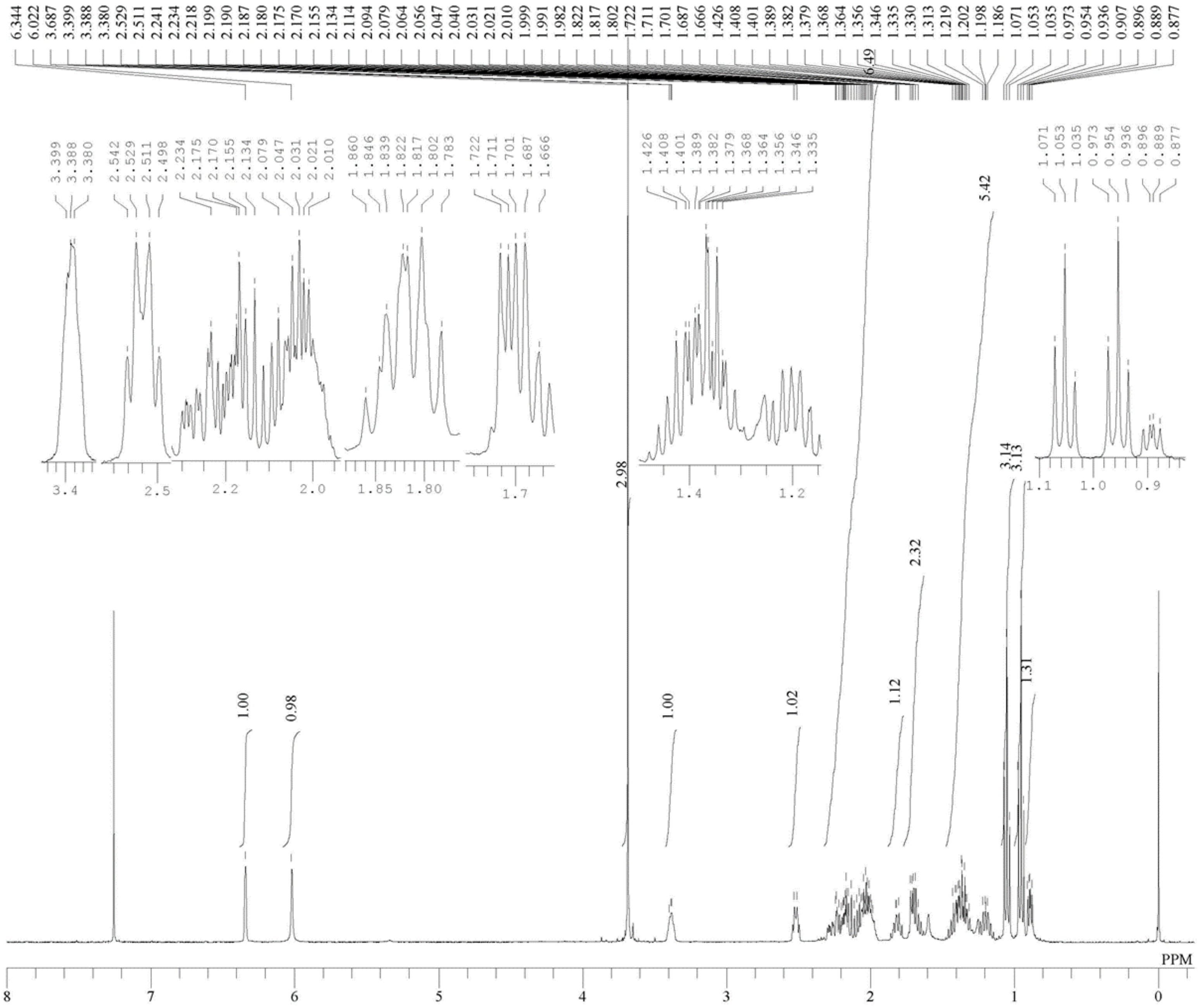

DFILE rw_2_194_Proton-1-1.als
COMNT single_pulse
DATIM 12-12-2018 23:35:51
OBNUC 1H
EXMOD proton.jxp
OBFRQ 399.78 MHz
OBSET 4.19 KHz
OBFIN 7.29 Hz
POINT 16400
FREQU 7503.00 Hz
SCANS 8
ACQTM 2.1837 sec
PD 5.0000 sec
PW1 2.95 usec
IRNUC 1H
CTEMP 20.2 c
SLVNT CDCL3
EXREF 0.00 ppm
BF 0.10 Hz
RGAIN 56

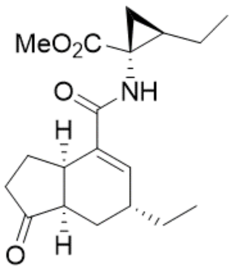

11n

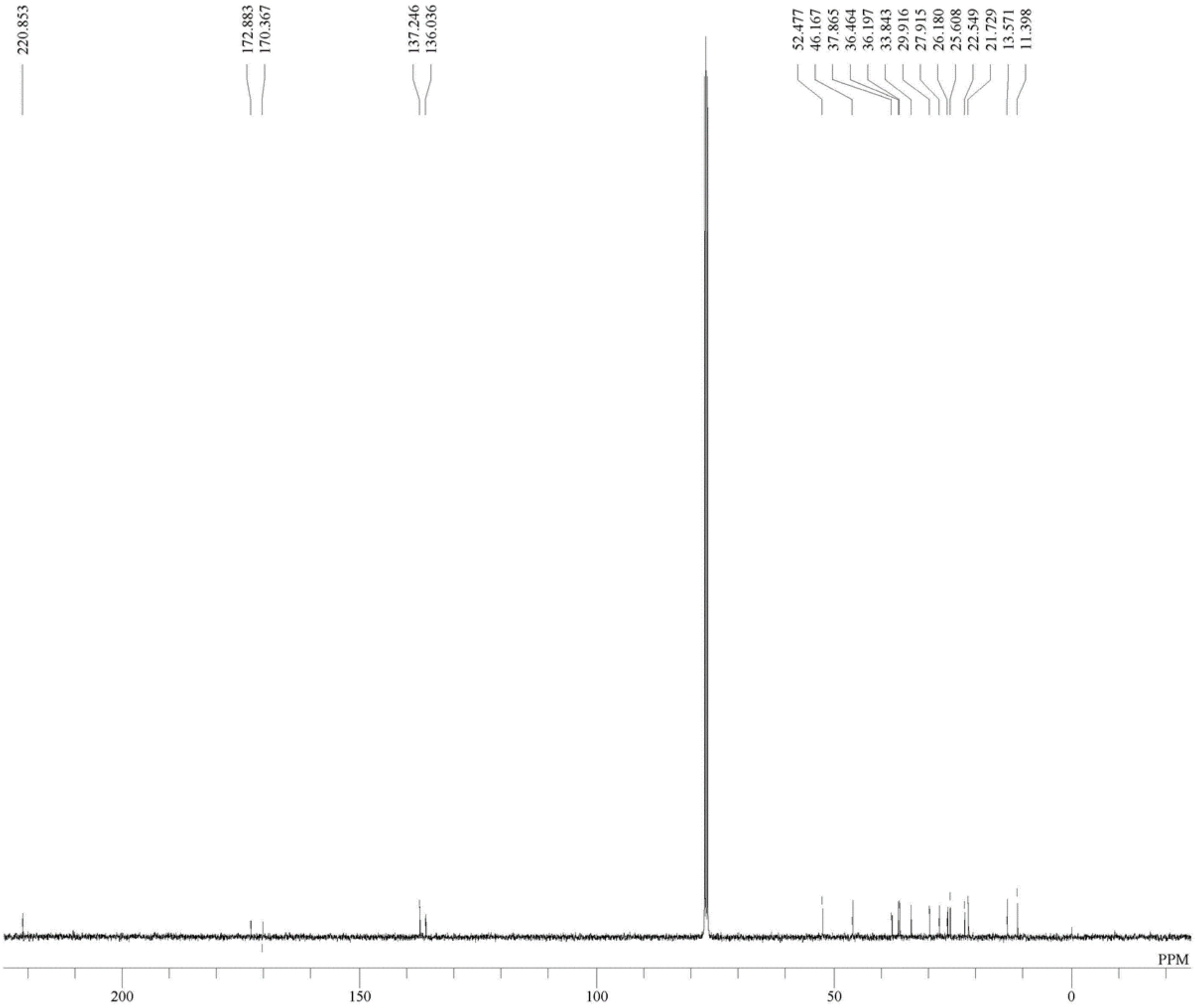

DFILE	rw_2_194_Carbon-1-1.als
COMNT	single pulse decoupled gated N
DATIM	12-12-2018 23:39:30
OBNUC	13C
EXMOD	carbon.jxp
OBFRQ	100.53 MHz
OBSET	5.35 KHz
OBFIN	5.86 Hz
POINT	32780
FREQU	31407.04 Hz
SCANS	2048
ACQTM	1.0433 sec
PD	2.0000 sec
PW1	3.37 usec
IRNUC	1H
CTEMP	19.7 c
SLVNT	CDCL3
EXREF	77.00 ppm
BF	0.10 Hz
RGAIN	50

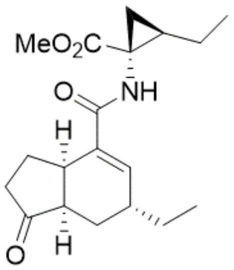

11n

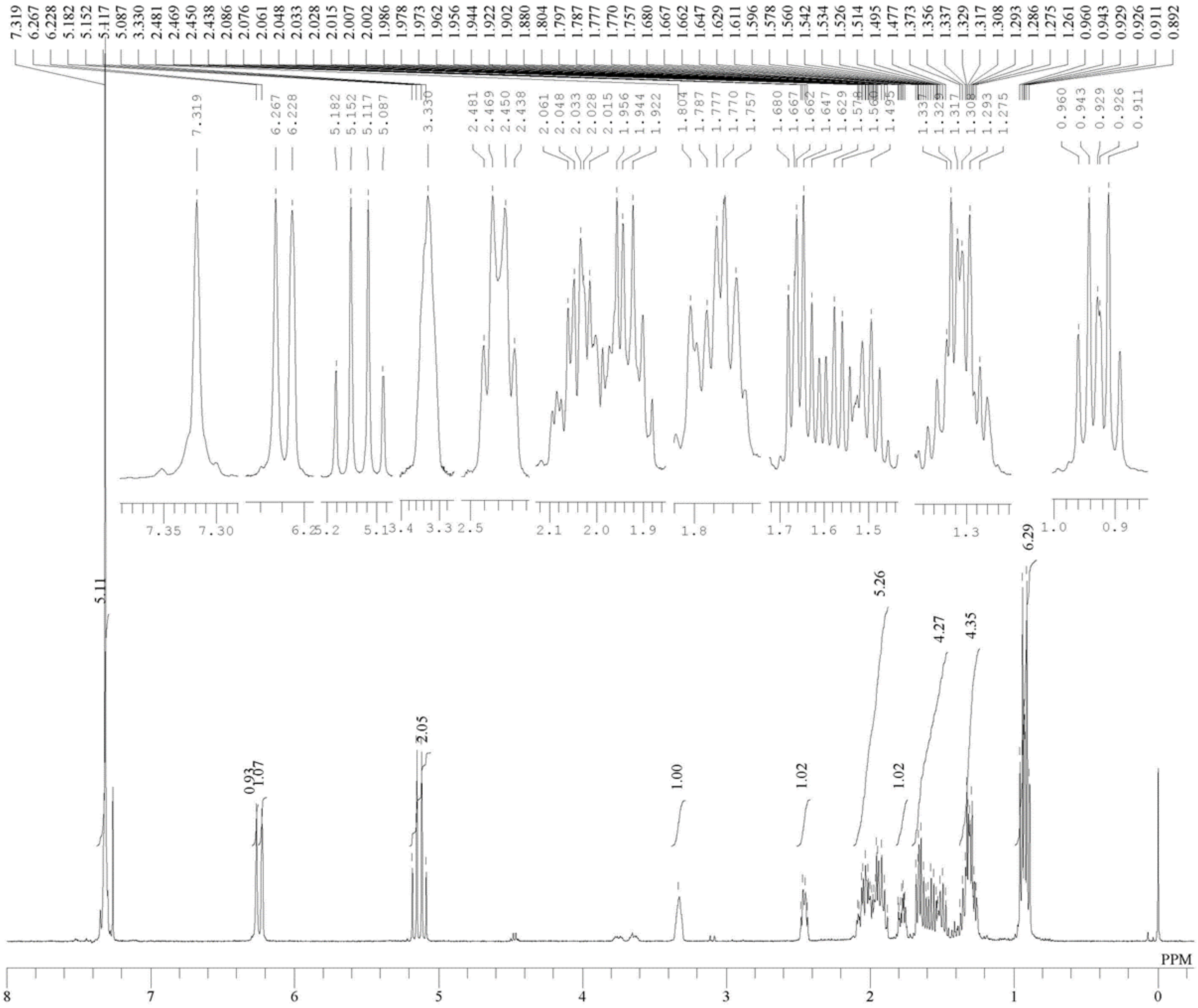

DFILE 3-138-8_Proton-1-1.als
COMNT single_pulse
DATIM 29-11-2018 21:13:57
OBNUC 1H
EXMOD proton.jxp
OBFRQ 399.78 MHz
OBSET 4.19 KHz
OBFIN 7.29 Hz
POINT 16384
FREQU 7503.00 Hz
SCANS 8
ACQTM 2.1837 sec
PD 5.0000 sec
PW1 2.95 usec
IRNUC 1H
CTEMP 22.0 c
SLVNT CDCL3
EXREF 0.00 ppm
BF 0.10 Hz
RGAIN 56

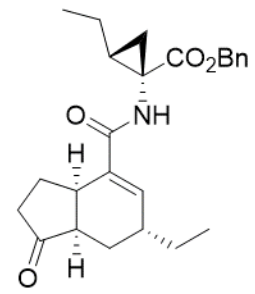

11o

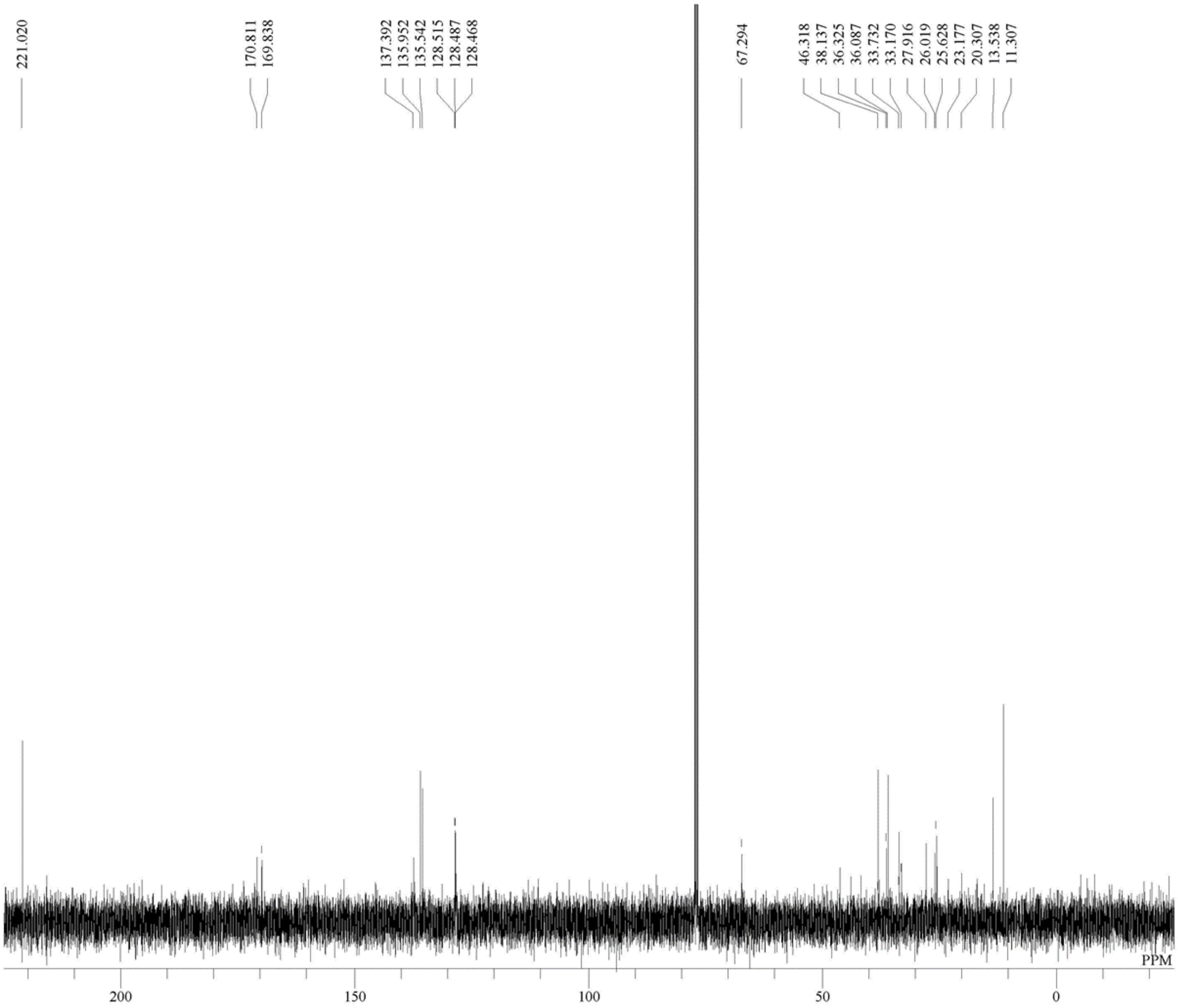

DFILE	3-138-8_Carbon-1-1.als
COMNT	single pulse decoupled gated N
DATIM	29-11-2018 21:15:09
OBNUC	13C
EXMOD	carbon.jxp
OBFRQ	100.53 MHz
OBSET	5.35 KHz
OBFIN	5.86 Hz
POINT	32767
FREQU	31407.04 Hz
SCANS	370
ACQTM	1.0433 sec
PD	2.0000 sec
PW1	3.37 usec
IRNUC	1H
CTEMP	22.1 c
SLVNT	CDCL3
EXREF	77.00 ppm
BF	0.10 Hz
RGAIN	50

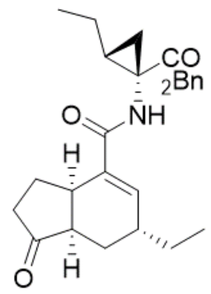

11o

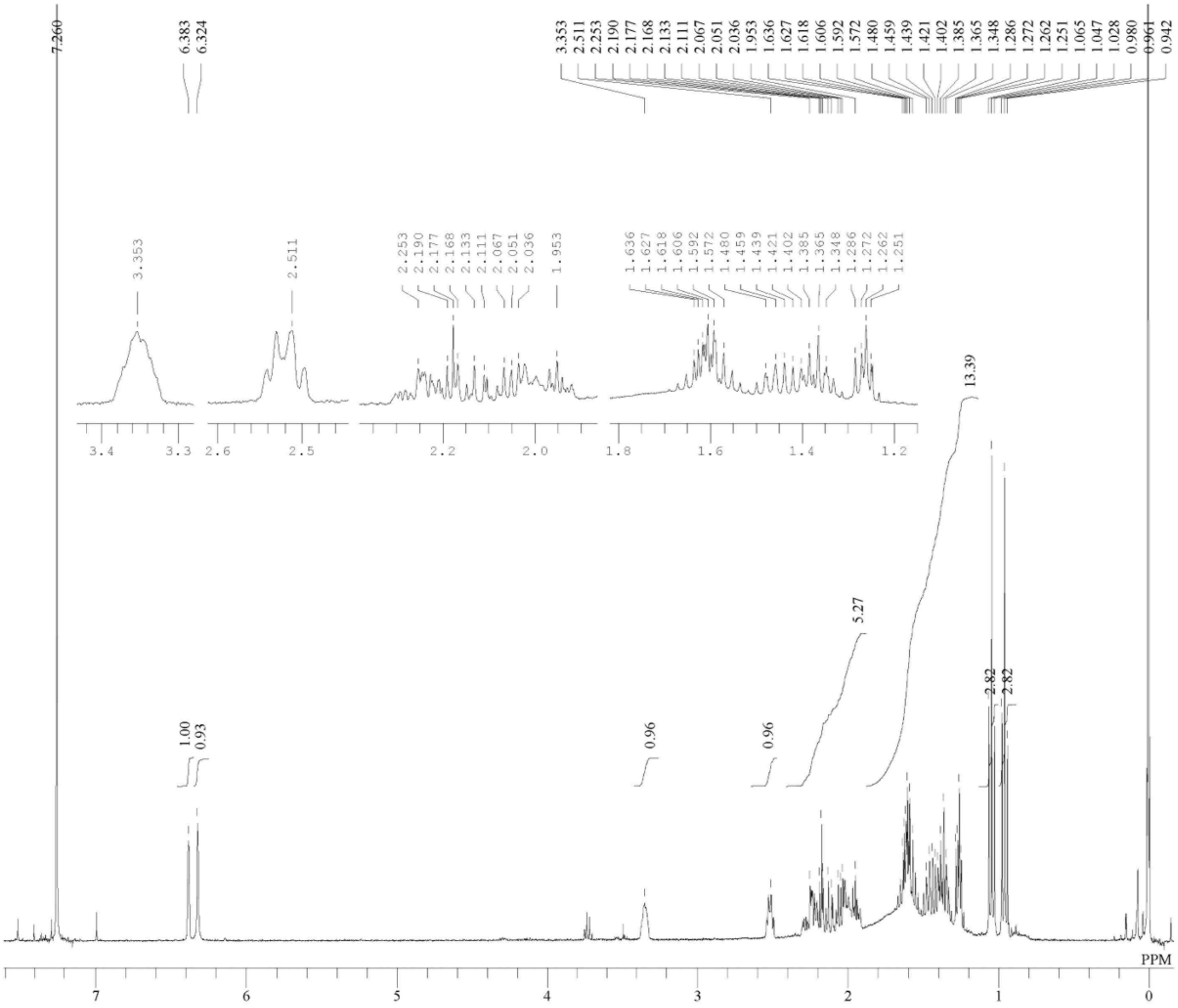
7.260
6.383
6.324
3.353
2.511
2.253
2.190
2.177
2.168
2.133
2.111
2.067
2.051
2.036
1.953
1.636
1.627
1.618
1.606
1.592
1.572
1.480
1.459
1.439
1.421
1.402
1.385
1.365
1.348
1.286
1.272
1.262
1.251
1.065
1.047
1.028
0.980
0.961
0.942
1.00
0.93
0.96
0.96
5.27
13.39
2.82
2.82
PPM
DFILE 2d_Proton-1-1.als
COMNT st_2_112_n_p1
DATIM 11/Jun/2017 05:48:13
OBNUC NUL
EXMOD proton.jxp
OBFRQ 399.78 MHz
OBSET 2.00 KHz
OBFIN 199.60 Hz
POINT 13120
FREQU 6001.85 Hz
SCANS 32
ACQTM 2.1860 sec
PD 5.0000 sec
PW1 2.95 usec
IRNUC NUL
CTEMP 23.9 c
SLVNT CHLOROFORM
EXREF 7.26 ppm
BF 0.25 Hz
RGAIN 58

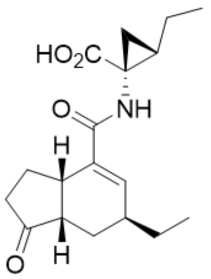
HO2C
NH
O
H
H
O

2d

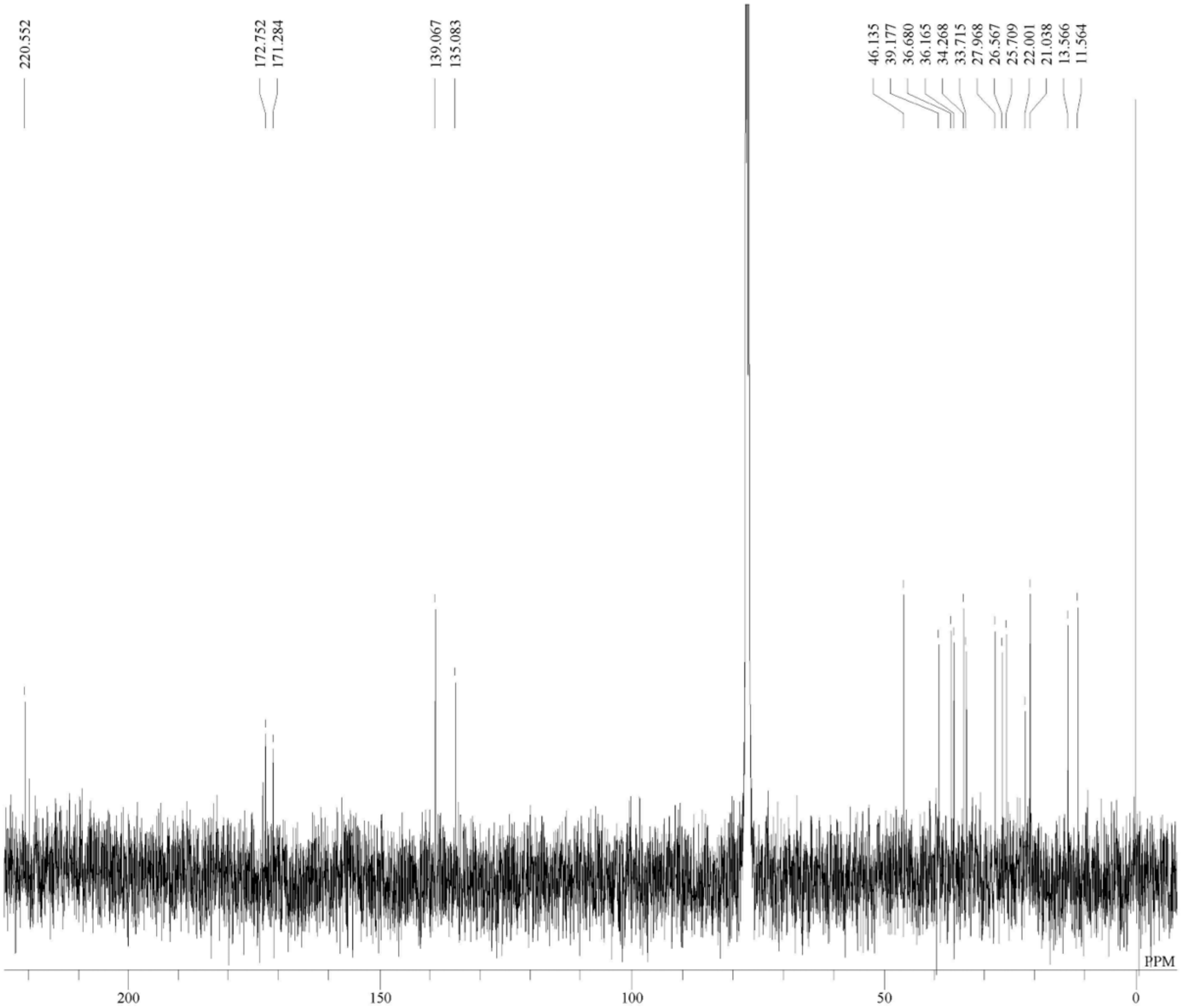

DFILE 2d_Carbon-1-1.als
COMNT st_2_110_n
DATIM 12/Jun/2017 00:49:41
OBNUC 13C
EXMOD carbon.jxp
OBFRQ 100.53 MHz
OBSET -5.00 KHz
OBFIN 304.55 Hz
POINT 26224
FREQU 25124.29 Hz
SCANS 11000
ACQTM 1.0438 sec
PD 2.0000 sec
PW1 3.37 usec
IRNUC NUL
CTEMP 24.0 c
SLVNT CHLOROFORM
EXREF 77.16 ppm
BF 0.25 Hz
RGAIN 50

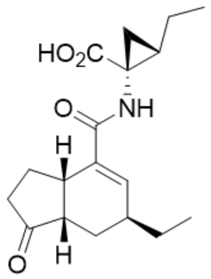

2d

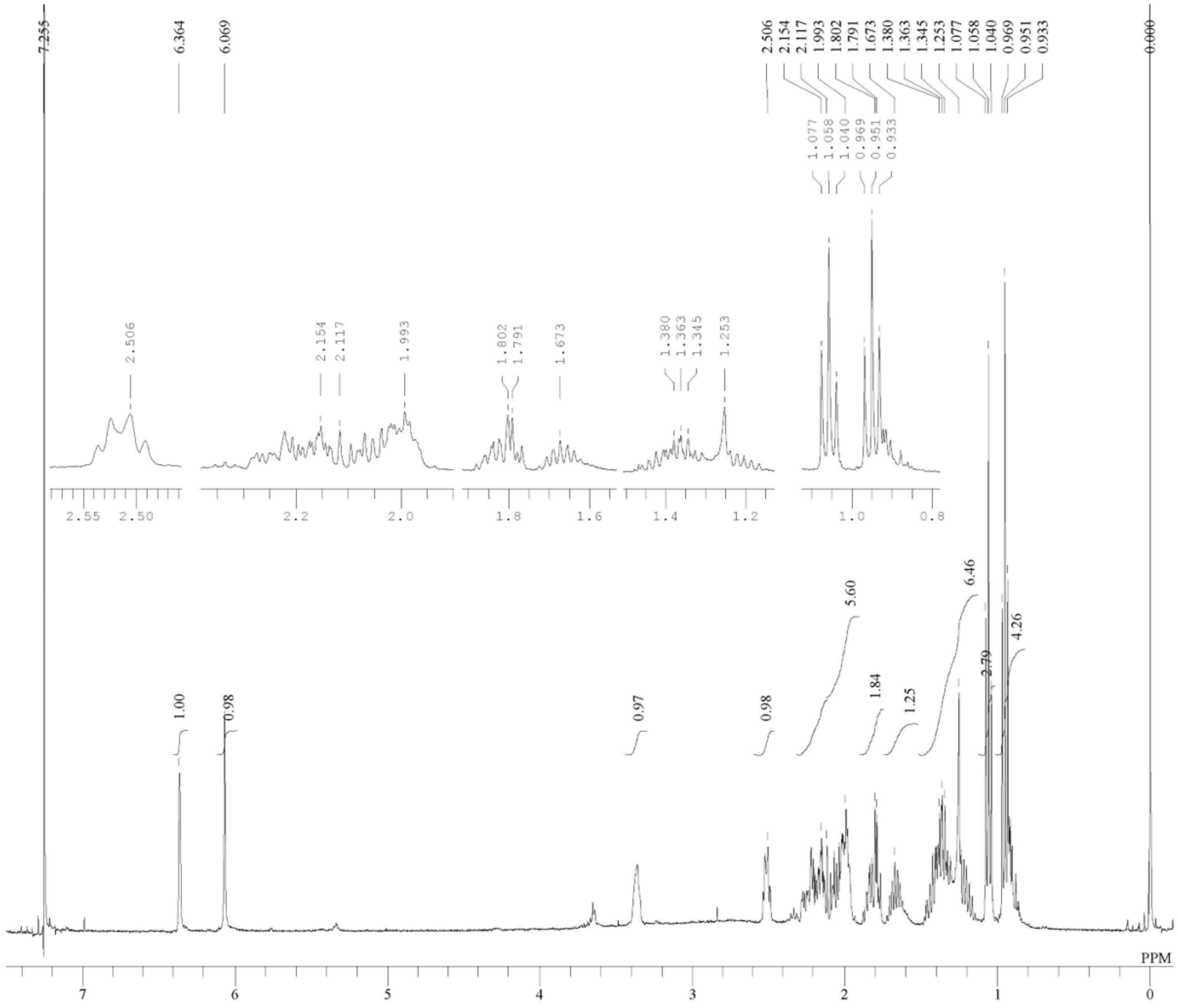

DFILE 2e_Proton-1-1.als
COMNT st_2_98p
DATIM 28/May/2017 13:23:06
OBNUC NUL
EXMOD proton.jxp
OBFRQ 399.78 MHz
OBSET 2.00 KHz
OBFIN 199.60 Hz
POINT 13120
FREQU 6001.85 Hz
SCANS 8
ACQTM 2.1860 sec
PD 5.0000 sec
PW1 2.95 usec
IRNUC NUL
CTEMP 23.9 c
SLVNT CHLOROFORM
EXREF 0.00 ppm
BF 0.25 Hz
RGAIN 54

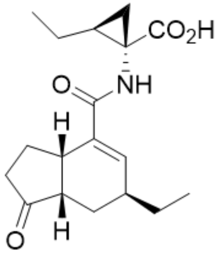

2e

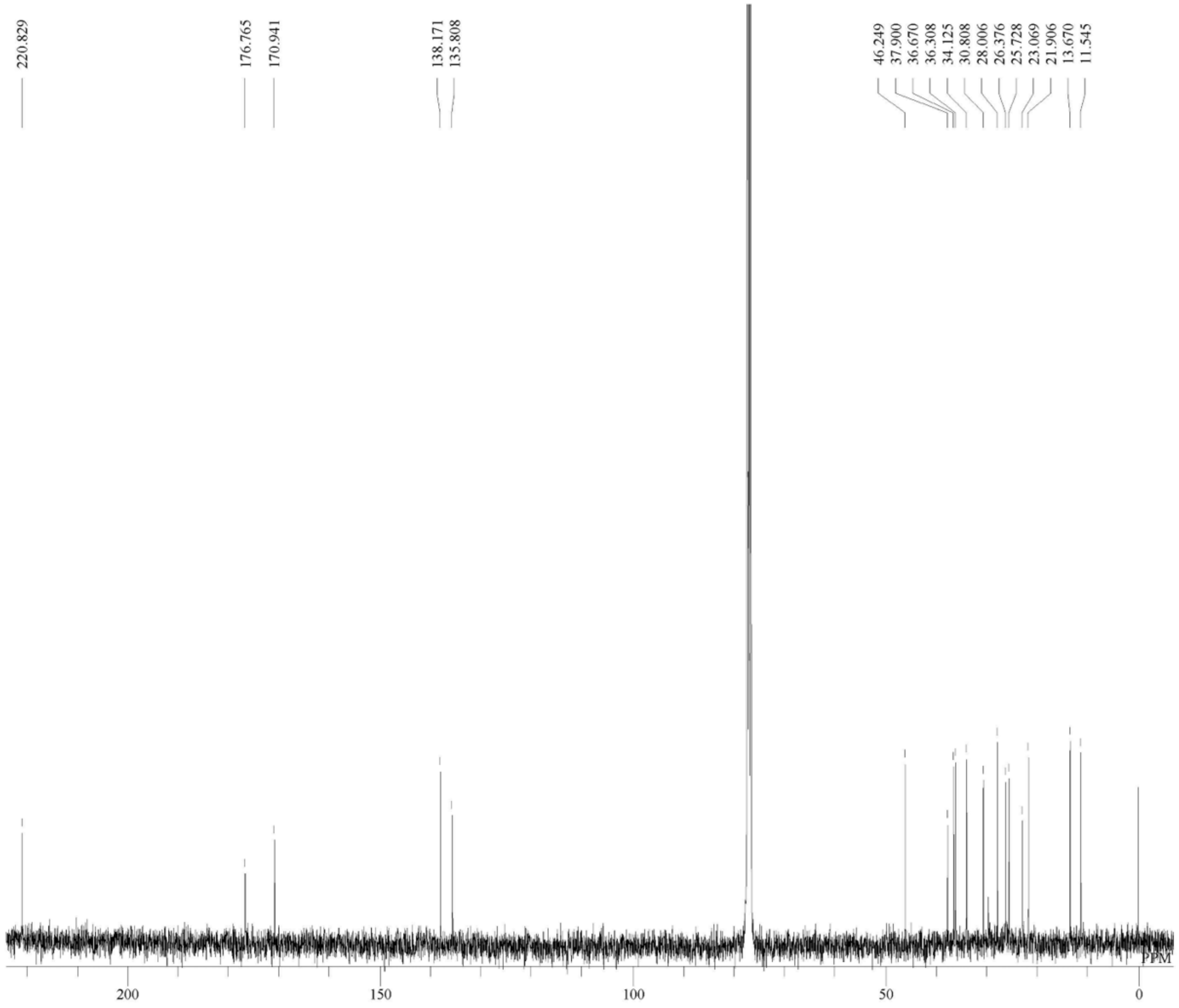

DFILE 2e_Carbon-1-1.als
COMNT st_2_98p
DATIM 29/May/2017 02:07:28
OBNUC 13C
EXMOD carbon.jxp
OBFRQ 100.53 MHz
OBSET -5.00 KHz
OBFIN 304.55 Hz
POINT 26224
FREQU 25124.29 Hz
SCANS 12895
ACQTM 1.0438 sec
PD 2.0000 sec
PW1 3.37 usec
IRNUC NUL
CTEMP 23.8 c
SLVNT CHLOROFORM
EXREF 77.16 ppm
BF 0.25 Hz
RGAIN 50

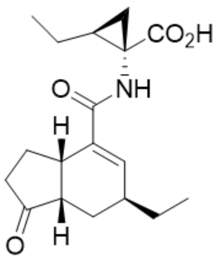

2e

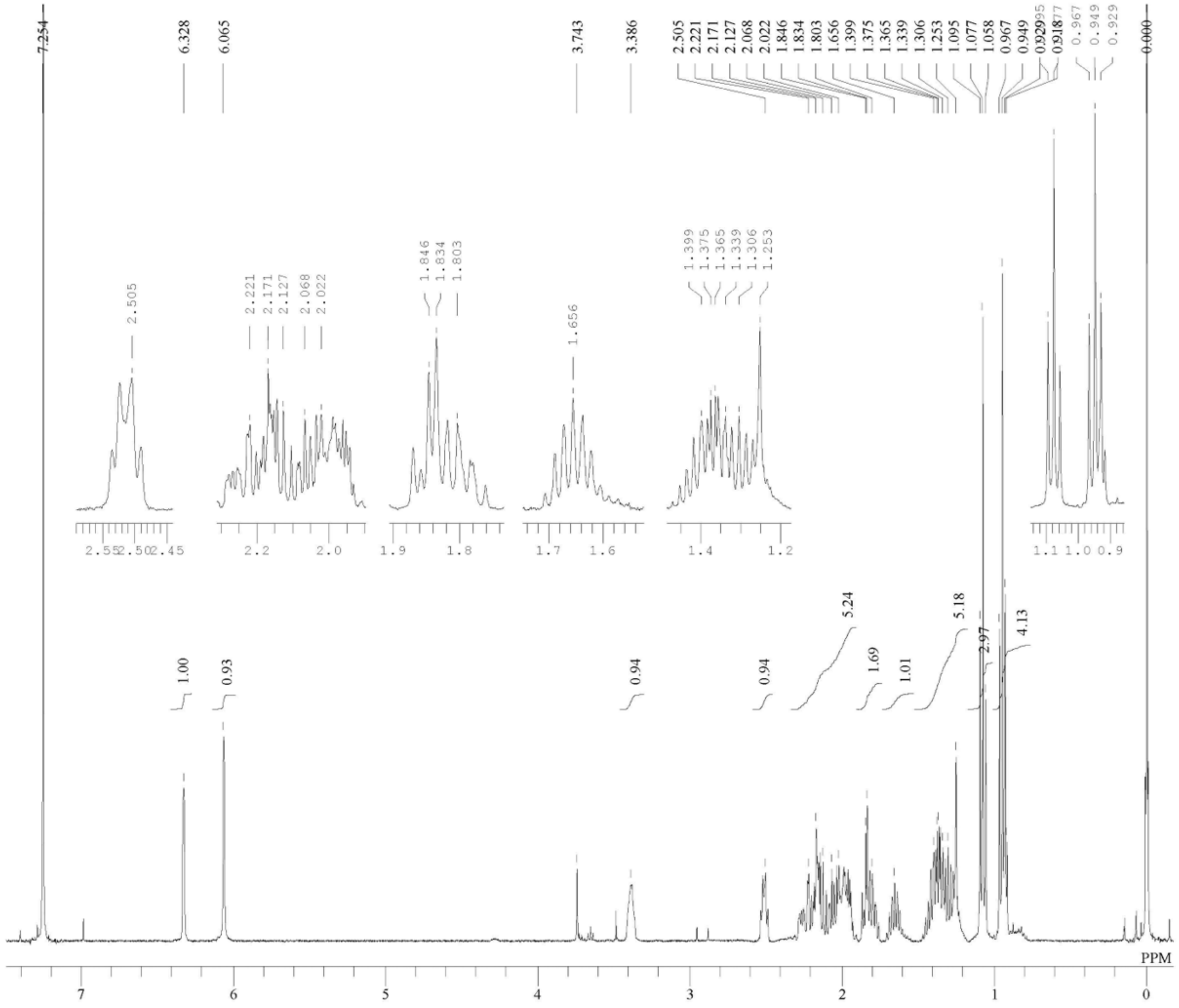

DFILE 2f_Proton-1-1.als
COMNT st_2_112_C2_p1
DATIM 11/Jun/2017 05:37:16
OBNUC NUL
EXMOD proton.jxp
OBFRQ 399.78 MHz
OBSET 2.00 KHz
OBFIN 199.60 Hz
POINT 13120
FREQU 6001.85 Hz
SCANS 32
ACQTM 2.1860 sec
PD 5.0000 sec
PW1 2.95 usec
IRNUC NUL
CTEMP 23.9 c
SLVNT CHLOROFORM
EXREF 0.00 ppm
BF 0.25 Hz
RGAIN 56

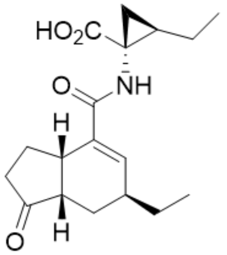

2f

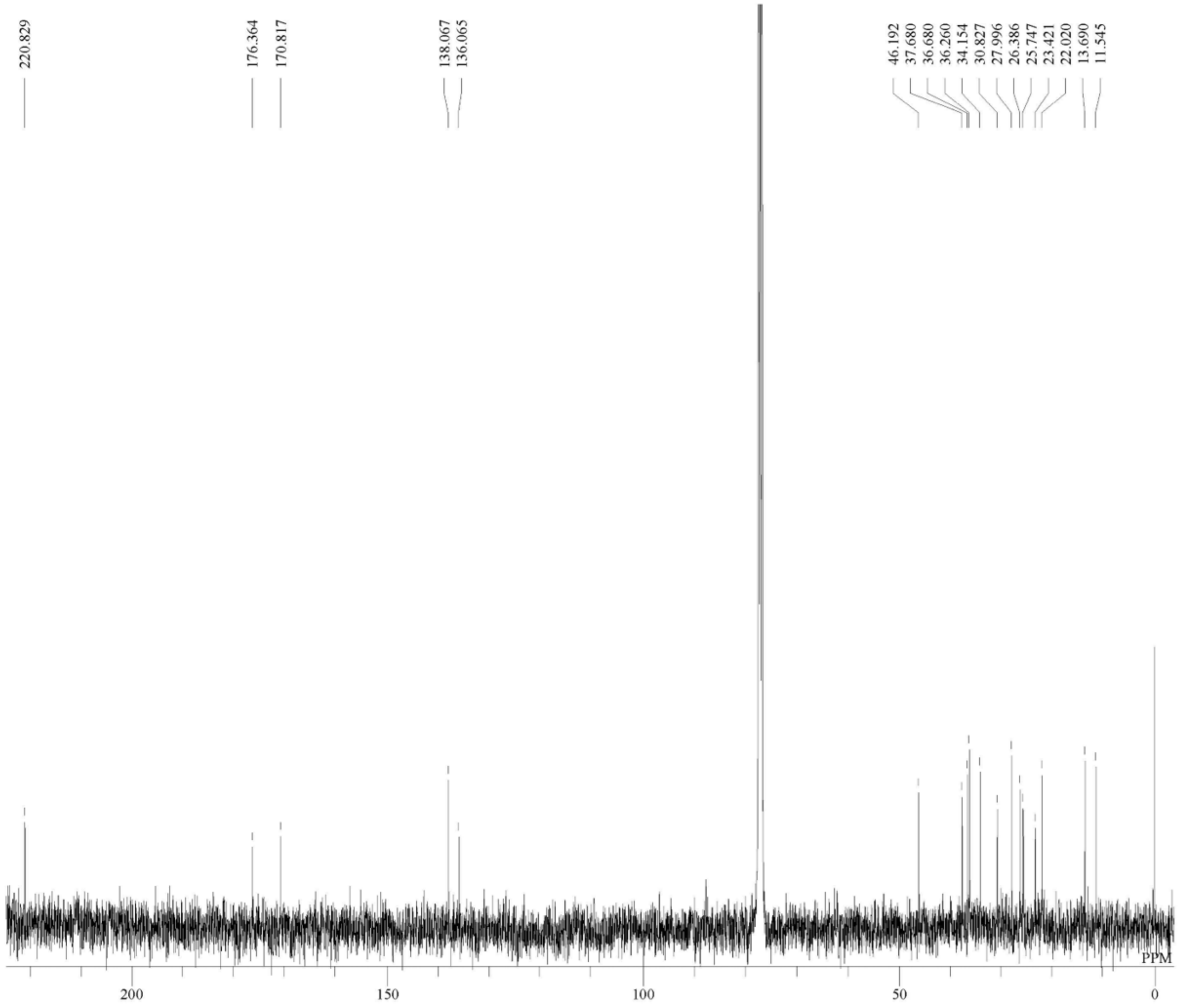

DFILE 2f_Carbon-1-1.als
COMNT st_2_112_C2
DATIM 11/Jun/2017 15:50:14
OBNUC 13C
EXMOD carbon.jxp
OBFRQ 100.53 MHz
OBSET -5.00 KHz
OBFIN 304.55 Hz
POINT 26224
FREQU 25124.29 Hz
SCANS 10502
ACQTM 1.0438 sec
PD 2.0000 sec
PW1 3.37 usec
IRNUC NUL
CTEMP 23.8 c
SLVNT CHLOROFORM
EXREF 77.16 ppm
BF 0.25 Hz
RGAIN 50

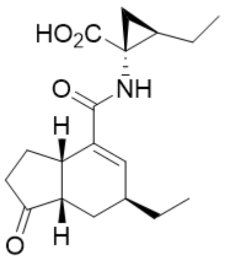

2f

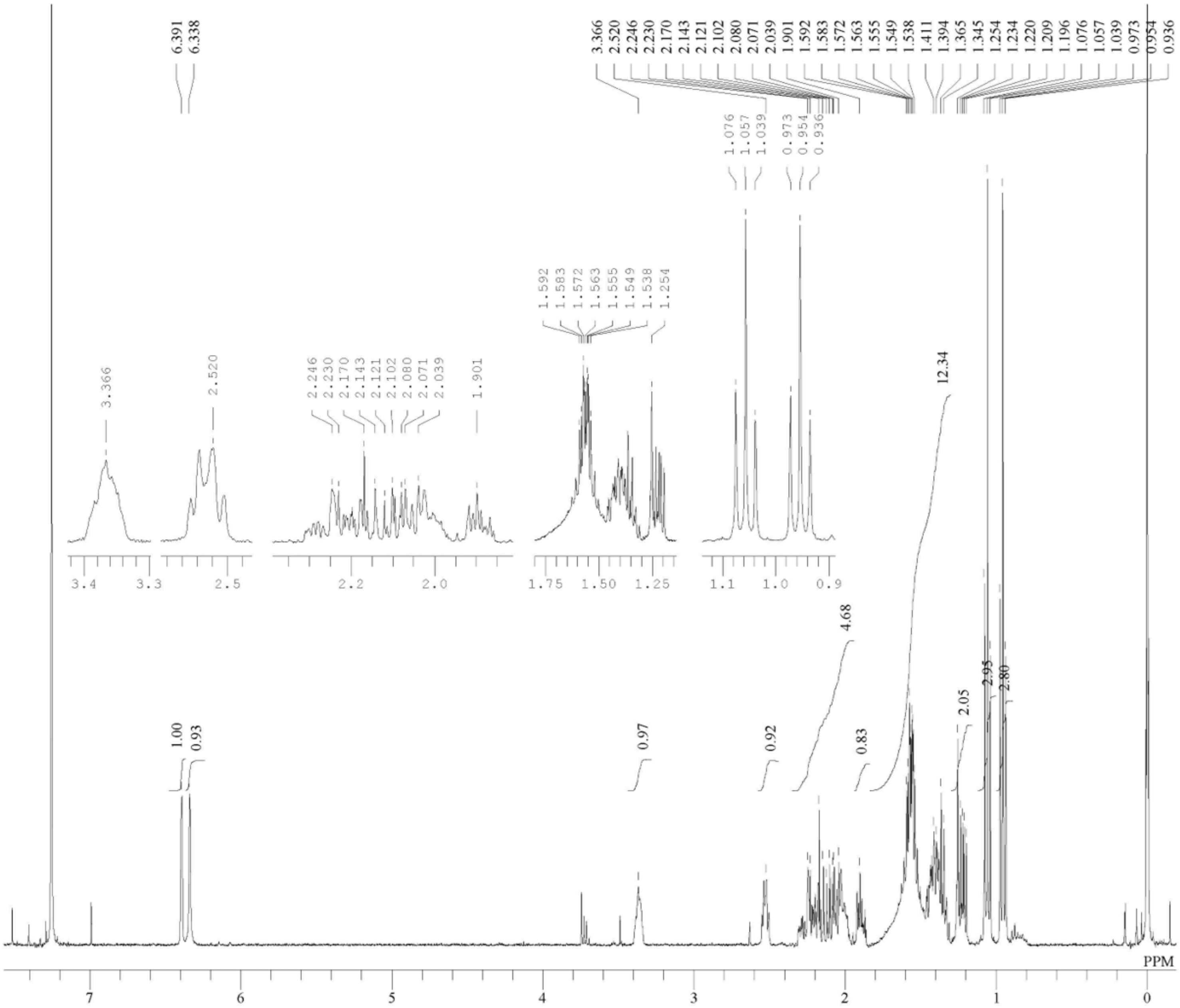

DFILE 2g_Proton-1-1.als
COMNT st_2_112_e_p1
DATIM 11/Jun/2017 05:58:14
OBNUC NUL
EXMOD proton.jxp
OBFRQ 399.78 MHz
OBSET 2.00 KHz
OBFIN 199.60 Hz
POINT 13120
FREQU 6001.85 Hz
SCANS 32
ACQTM 2.1860 sec
PD 5.0000 sec
PW1 2.95 usec
IRNUC NUL
CTEMP 23.9 c
SLVNT CHLOROFORM
EXREF 0.00 ppm
BF 0.25 Hz
RGAIN 58

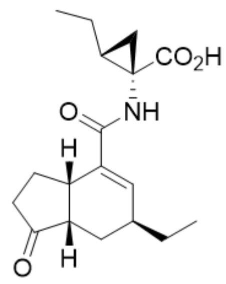

2g

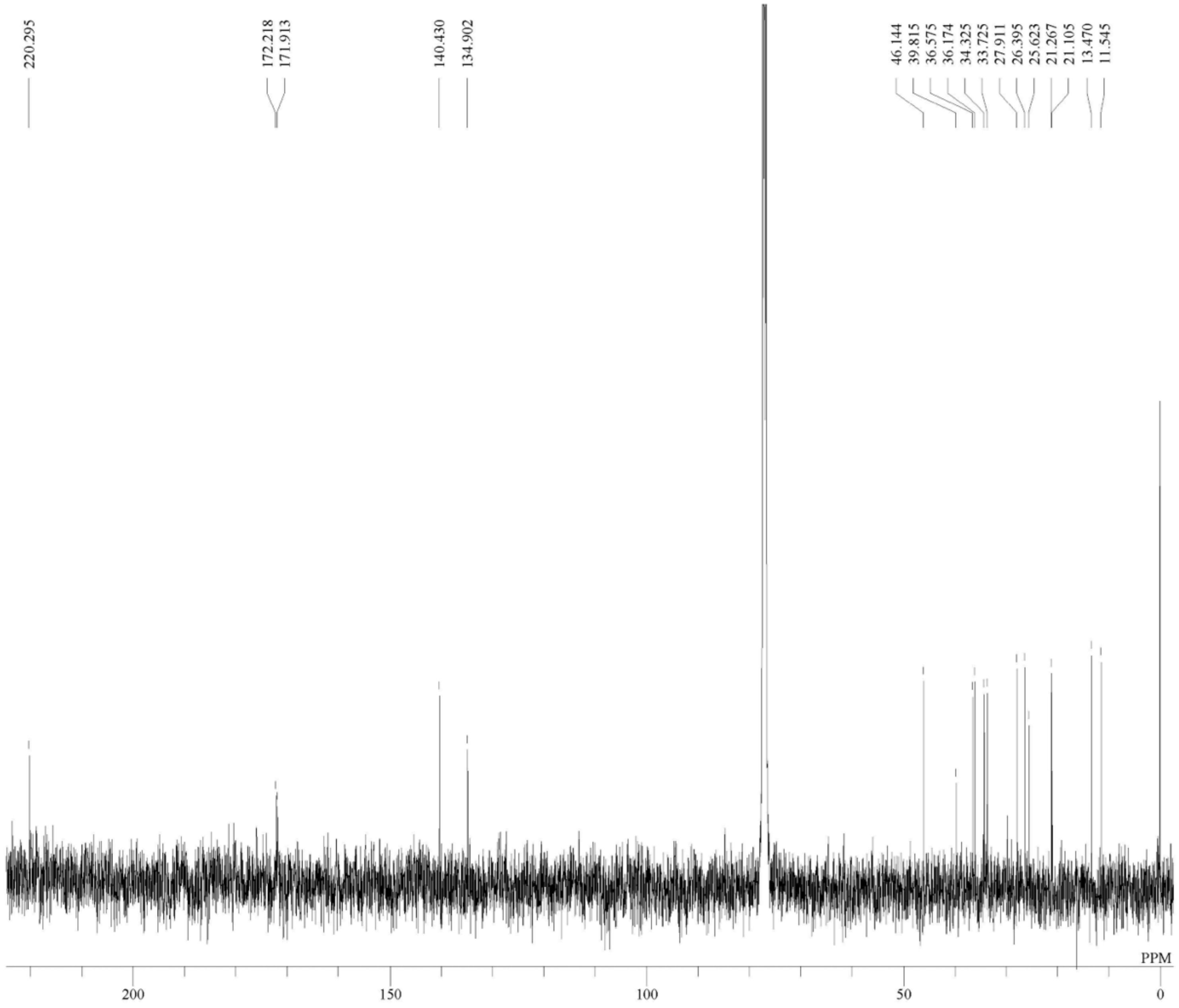

DFILE 2g_Carbon-1-1.als
COMNT st_2_111_e
DATIM 13/Jun/2017 01:05:48
OBNUC 13C
EXMOD carbon.jxp
OBFRQ 100.53 MHz
OBSET -5.00 KHz
OBFIN 304.55 Hz
POINT 26224
FREQU 25124.29 Hz
SCANS 10845
ACQTM 1.0438 sec
PD 2.0000 sec
PW1 3.37 usec
IRNUC NUL
CTEMP 23.6 c
SLVNT CHLOROFORM
EXREF 77.16 ppm
BF 0.25 Hz
RGAIN 50

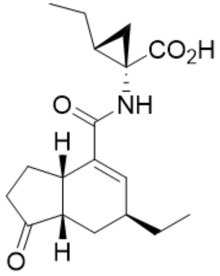

2g

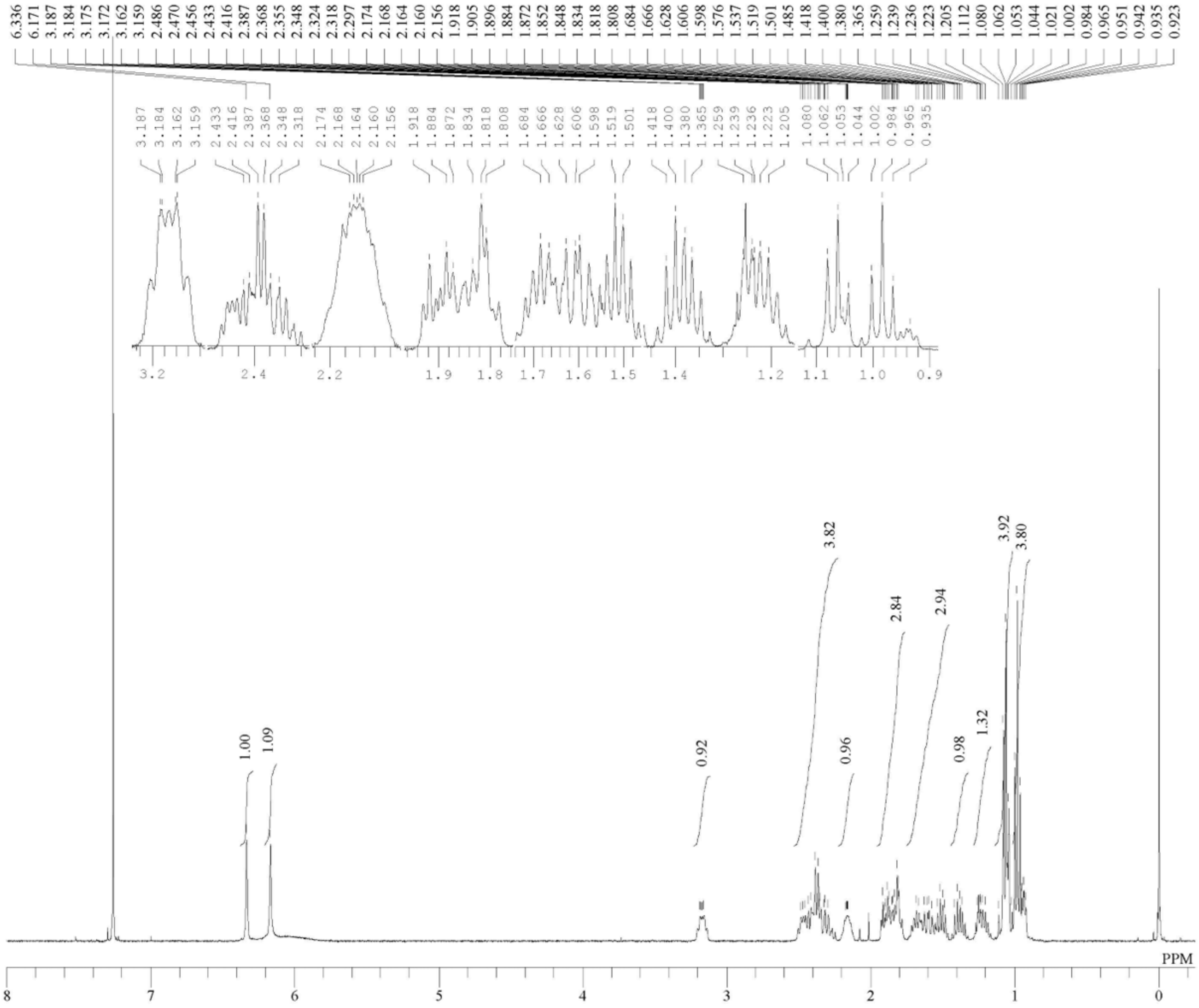

DFILE	3-162-1_Proton-1-1.jdf
COMNT	single_pulse
DATIM	17-01-2019 11:31:16
OBNUC	1H
EXMOD	proton.jxp
OBFRQ	399.78 MHz
OBSET	4.19 KHz
OBFIN	7.29 Hz
POINT	16384
FREQU	7503.00 Hz
SCANS	8
ACQTM	2.1837 sec
PD	5.0000 sec
PW1	2.95 usec
IRNUC	1H
CTEMP	17.6 c
SLVNT	CDCL3
EXREF	0.00 ppm
BF	0.10 Hz
RGAIN	62

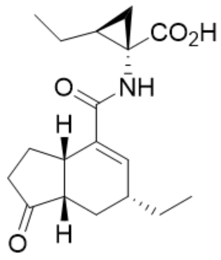

2h

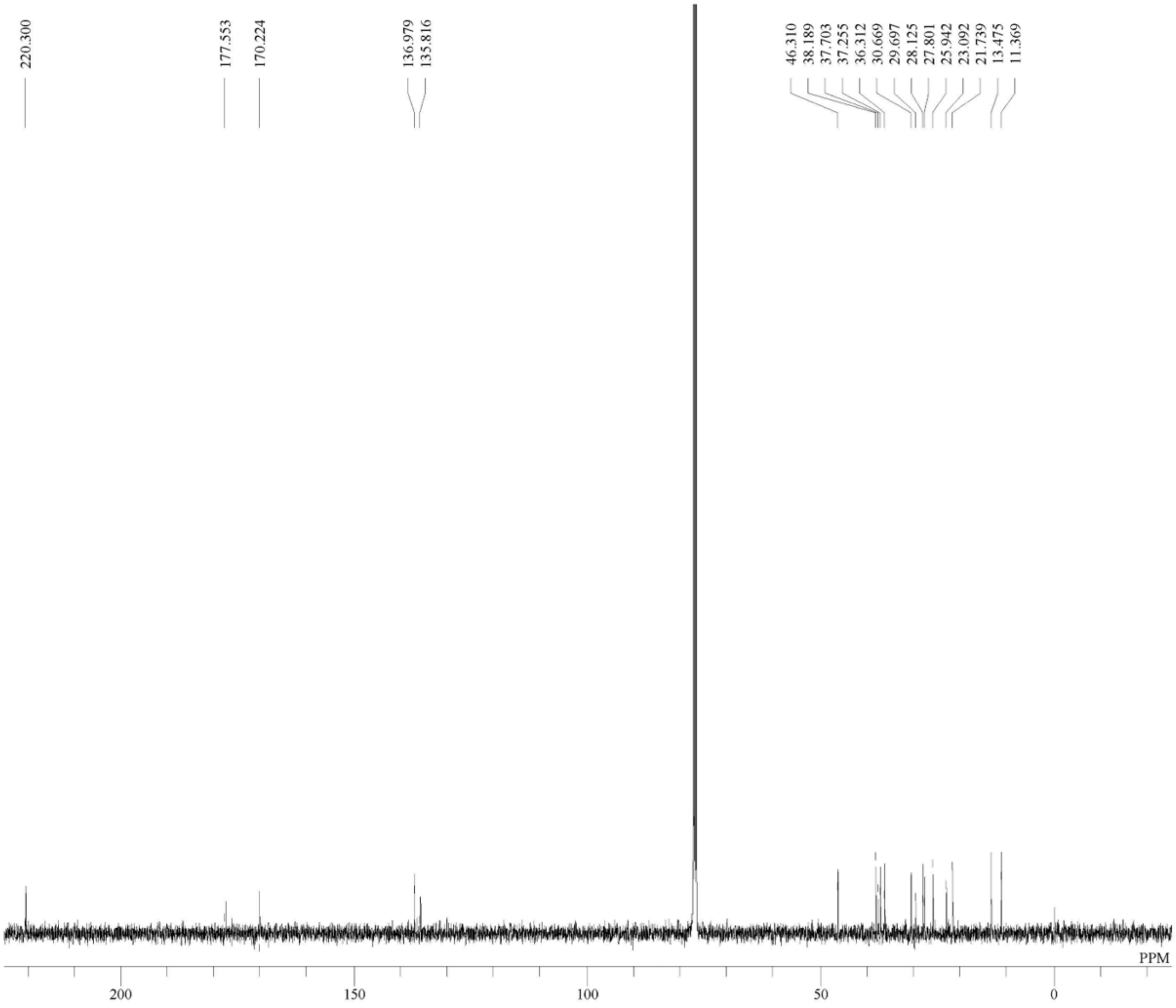

DFILE	3-158-1_Carbon-1-1.jdf
COMNT	single pulse decoupled gated N[illegible]
DATIM	15-01-2019 22:37:28
OBNUC	13C
EXMOD	carbon.jxp
OBFRQ	100.53 MHz
OBSET	5.35 KHz
OBFIN	5.86 Hz
POINT	32780
FREQU	31407.04 Hz
SCANS	1373
ACQTM	1.0433 sec
PD	2.0000 sec
PW1	3.37 usec
IRNUC	1H
CTEMP	18.5 c
SLVNT	CDCL3
EXREF	77.00 ppm
BF	0.10 Hz
RGAIN	50

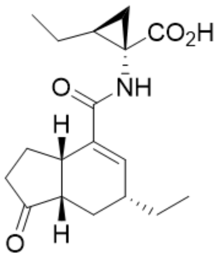

2h

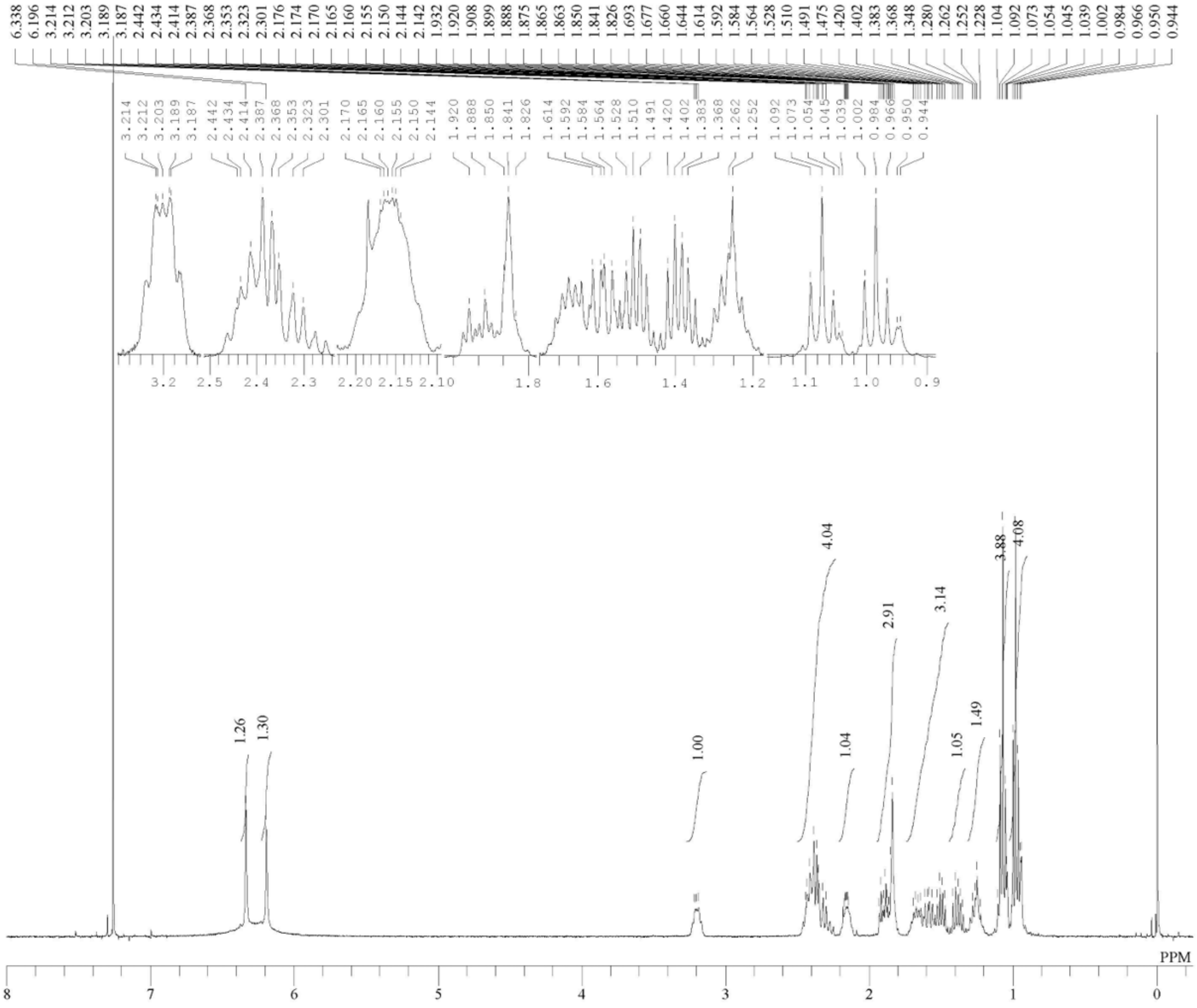

DFILE 3-162-2_Proton-1-1.jdf
COMNT single_pulse
DATIM 17-01-2019 16:32:10
OBNUC 1H
EXMOD proton.jxp
OBFRQ 399.78 MHz
OBSET 4.19 KHz
OBFIN 7.29 Hz
POINT 16384
FREQU 7503.00 Hz
SCANS 8
ACQTM 2.1837 sec
PD 5.0000 sec
PW1 2.95 usec
IRNUC 1H
CTEMP 18.5 c
SLVNT CDCL3
EXREF 0.00 ppm
BF 0.10 Hz
RGAIN 58

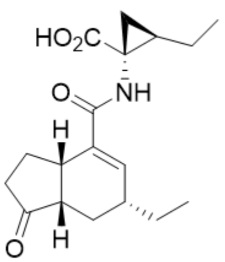

2i

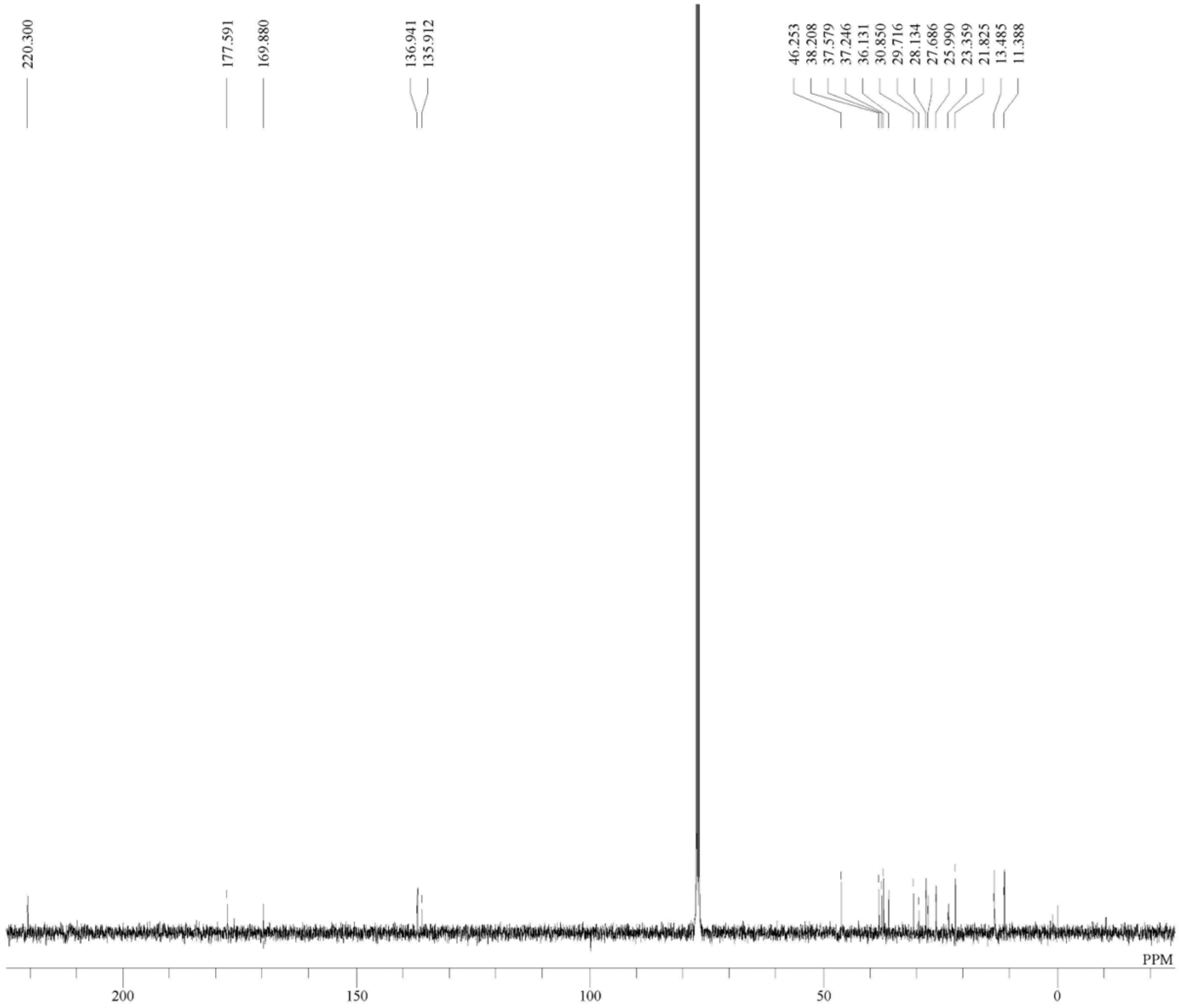

DFILE	3-158-2_Carbon-1-1.jdf
COMNT	single pulse decoupled gated N
DATIM	15-01-2019 23:53:43
OBNUC	13C
EXMOD	carbon.jxp
OBFRQ	100.53 MHz
OBSET	5.35 KHz
OBFIN	5.86 Hz
POINT	32780
FREQU	31407.04 Hz
SCANS	1177
ACQTM	1.0433 sec
PD	2.0000 sec
PW1	3.37 usec
IRNUC	1H
CTEMP	18.3 c
SLVNT	CDCL3
EXREF	77.00 ppm
BF	0.10 Hz
RGAIN	50

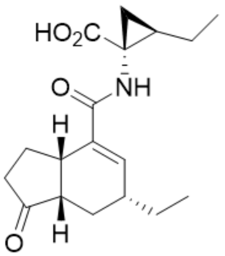

2i

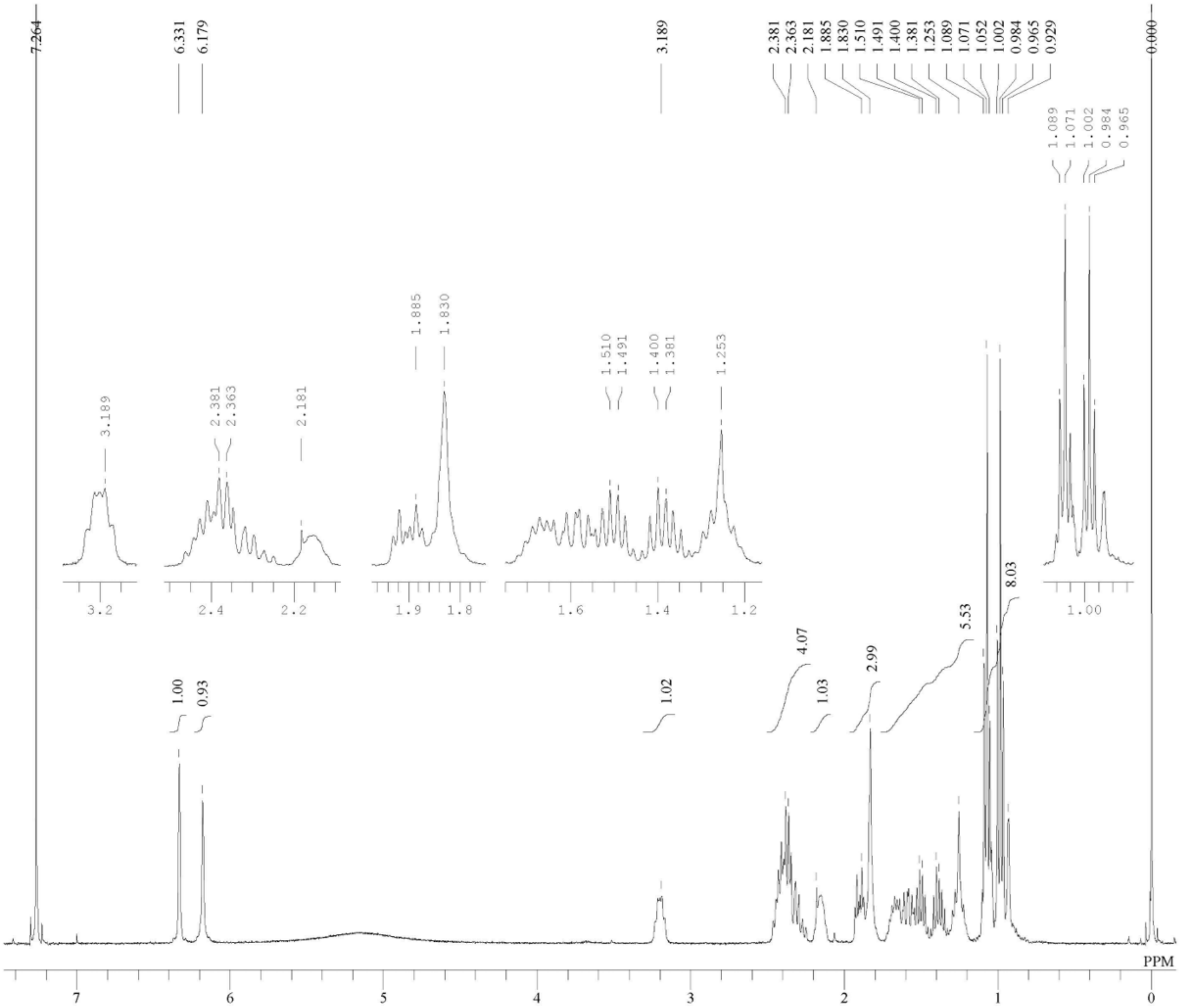

DFILE	2j_Proton-1-1.jdf
COMNT	single_pulse
DATIM	17-01-2019 16:38:27
OBNUC	1H
EXMOD	proton.jxp
OBFRQ	399.78 MHz
OBSET	4.19 KHz
OBFIN	7.29 Hz
POINT	16384
FREQU	7503.00 Hz
SCANS	8
ACQTM	2.1837 sec
PD	5.0000 sec
PW1	2.95 usec
IRNUC	1H
CTEMP	18.5 c
SLVNT	CDCL3
EXREF	0.00 ppm
BF	0.25 Hz
RGAIN	60

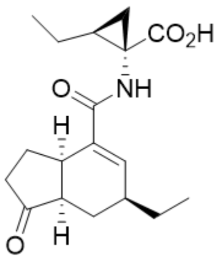

2j

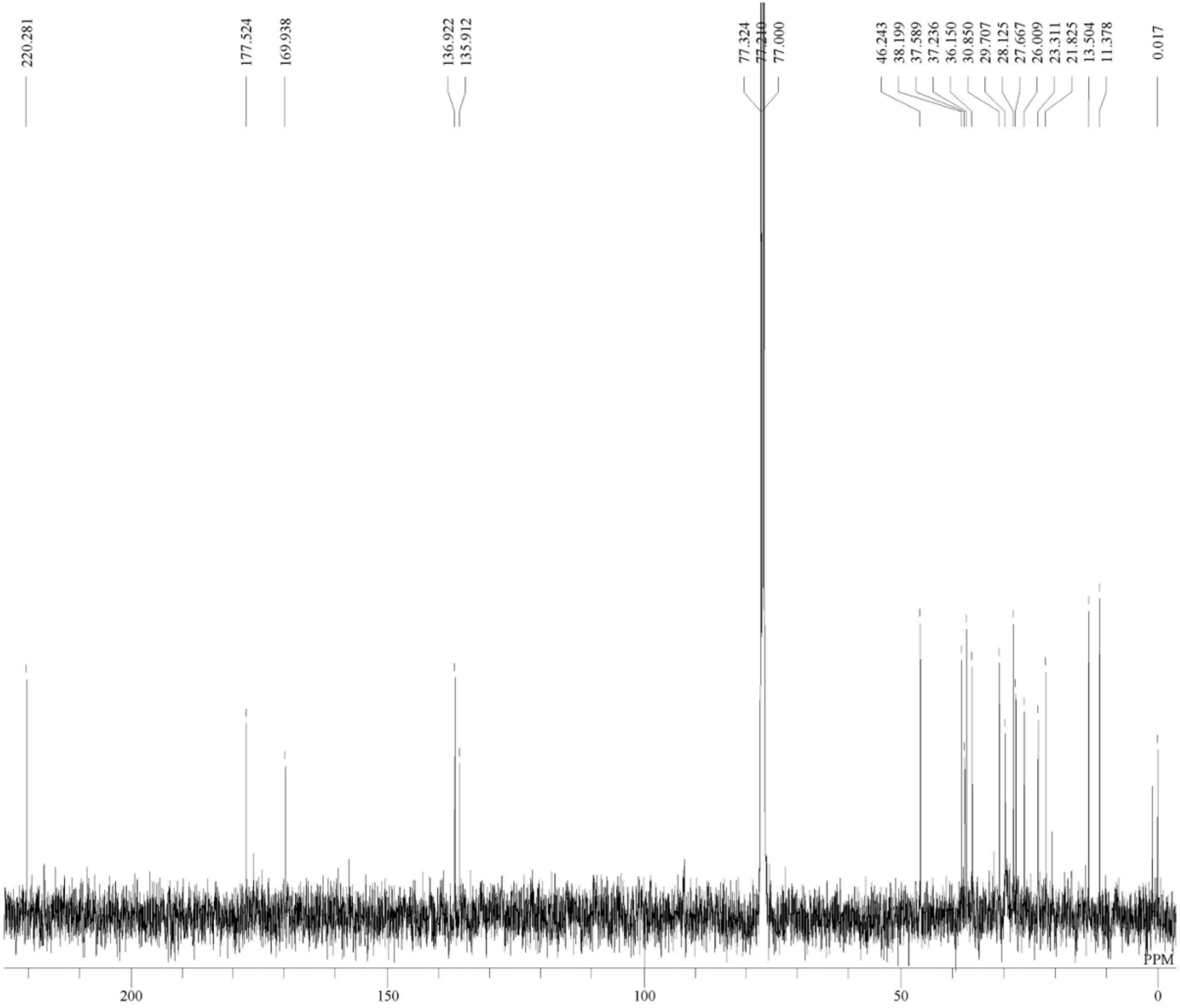

DFILE 2j_Carbon-1-1.jdf
COMNT single pulse decoupled gated N[illegible]
DATIM 16-01-2019 01:00:37
OBNUC 13C
EXMOD carbon.jxp
OBFRQ 100.53 MHz
OBSET 5.35 KHz
OBFIN 5.86 Hz
POINT 32780
FREQU 31407.04 Hz
SCANS 4096
ACQTM 1.0433 sec
PD 2.0000 sec
PW1 3.37 usec
IRNUC 1H
CTEMP 18.1 c
SLVNT CDCL3
EXREF 77.00 ppm
BF 0.25 Hz
RGAIN 50

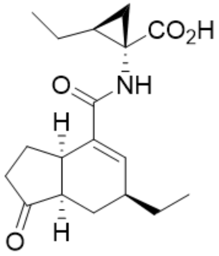

2j

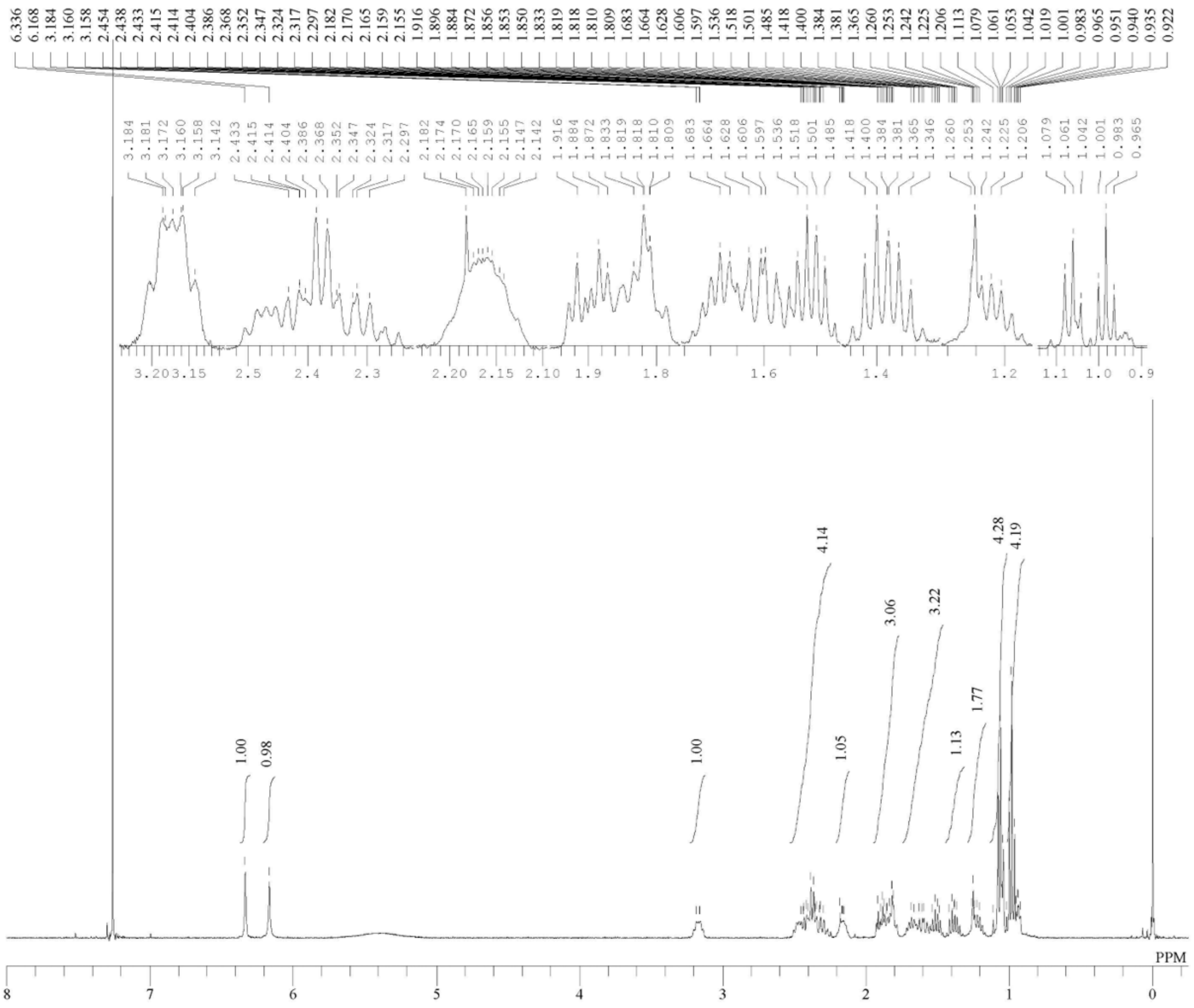

DFILE	3-162-4_Proton-1-1.jdf
COMNT	single_pulse
DATIM	17-01-2019 17:00:07
OBNUC	1H
EXMOD	proton.jxp
OBFRQ	399.78 MHz
OBSET	4.19 KHz
OBFIN	7.29 Hz
POINT	16384
FREQU	7503.00 Hz
SCANS	8
ACQTM	2.1837 sec
PD	5.0000 sec
PW1	2.95 usec
IRNUC	1H
CTEMP	18.6 c
SLVNT	CDCL3
EXREF	0.00 ppm
BF	0.10 Hz
RGAIN	58

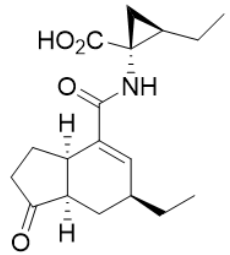

2k

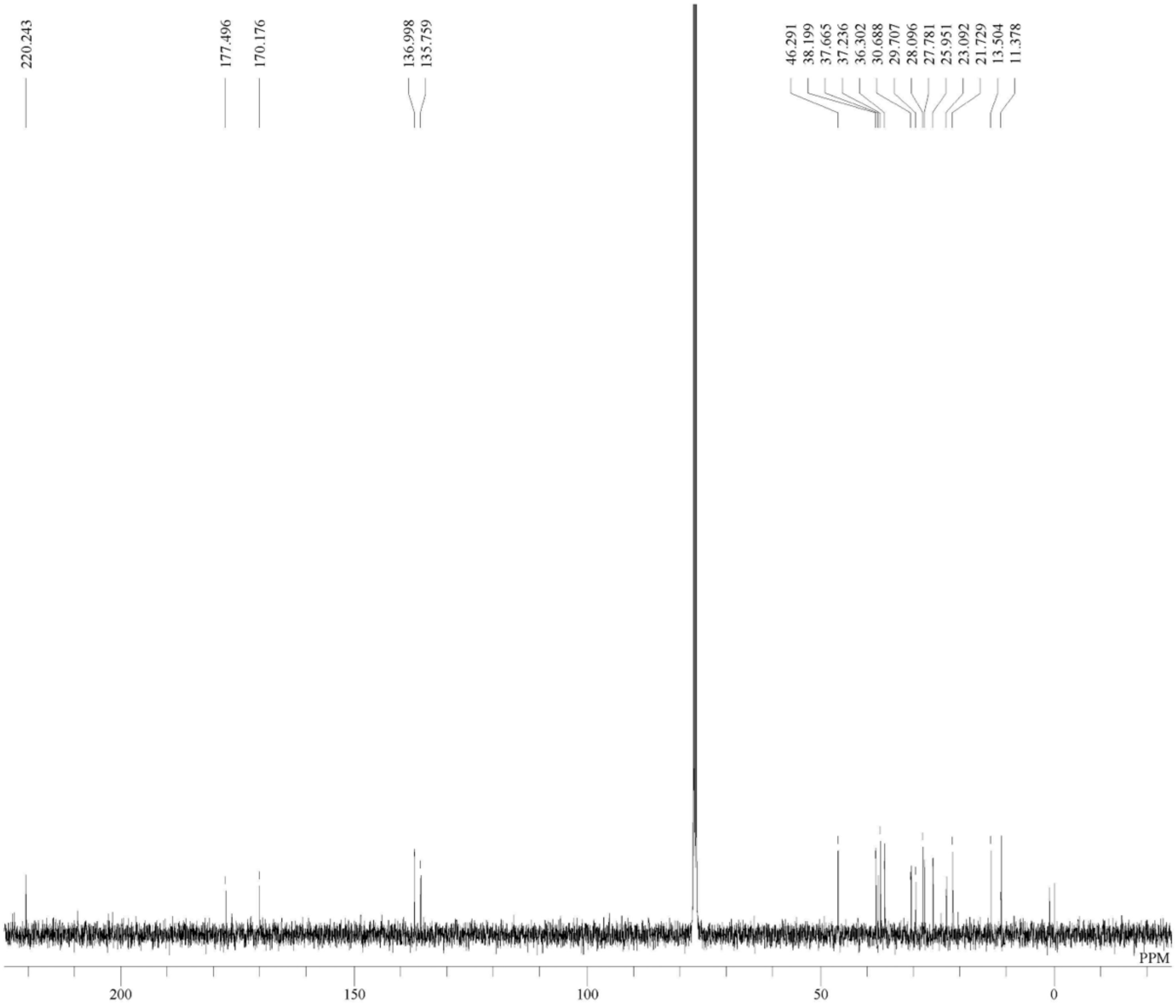

DFILE	3-158-4_Carbon-1-1.jdf
COMNT	single pulse decoupled gated N
DATIM	16-01-2019 11:33:08
OBNUC	13C
EXMOD	carbon.jxp
OBFRQ	100.53 MHz
OBSET	5.35 KHz
OBFIN	5.86 Hz
POINT	32780
FREQU	31407.04 Hz
SCANS	1480
ACQTM	1.0433 sec
PD	2.0000 sec
PW1	3.37 usec
IRNUC	1H
CTEMP	18.2 c
SLVNT	CDCL3
EXREF	77.00 ppm
BF	0.10 Hz
RGAIN	50

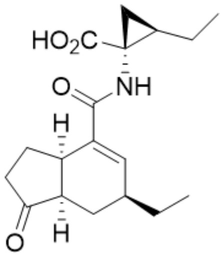

2k

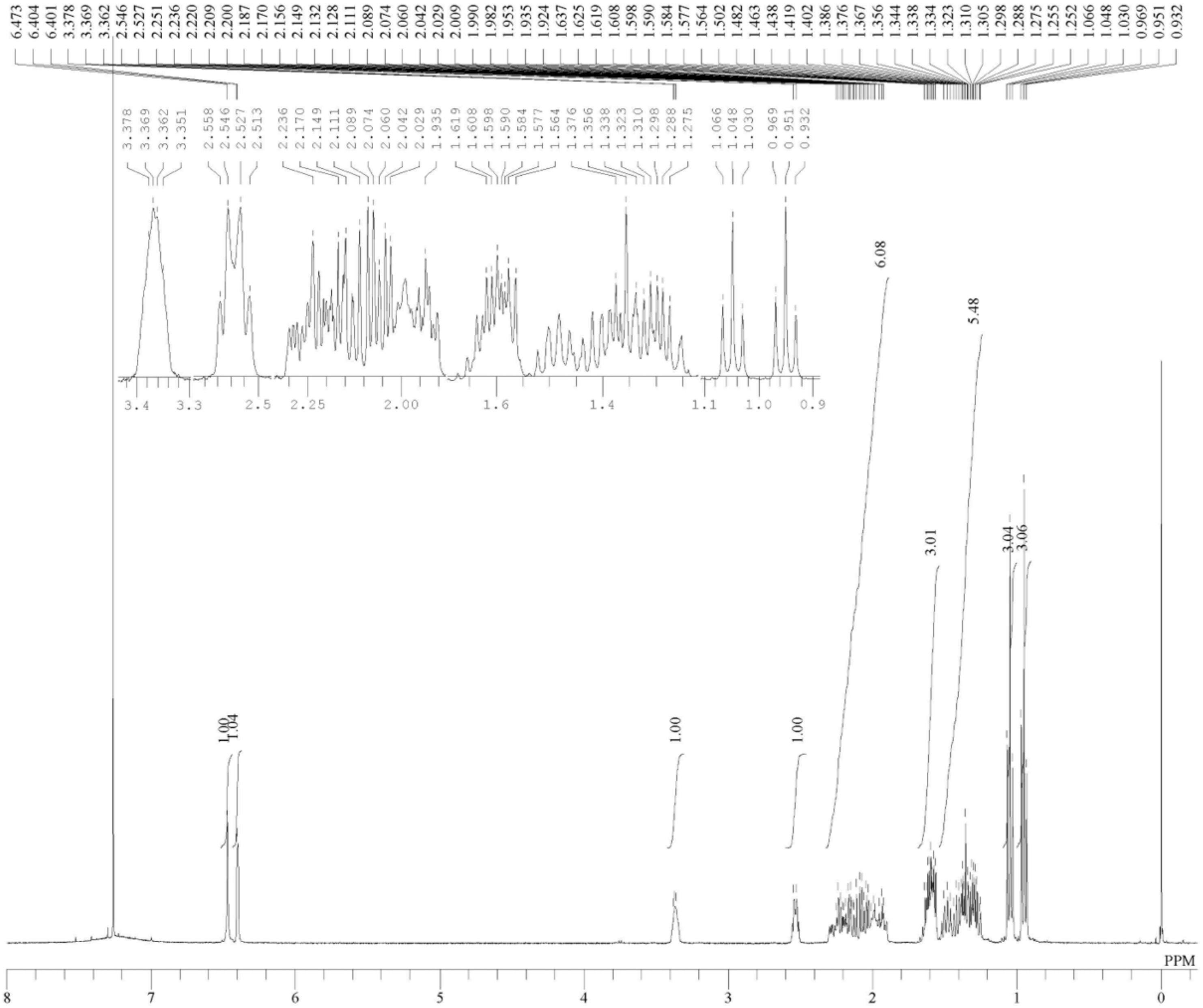

DFILE 3-153-4_Proton-1-1.als
COMNT single_pulse
DATIM 02-01-2019 21:31:55
OBNUC 1H
EXMOD proton.jxp
OBFRQ 399.78 MHz
OBSET 4.19 KHz
OBFIN 7.29 Hz
POINT 16384
FREQU 7503.00 Hz
SCANS 8
ACQTM 2.1837 sec
PD 5.0000 sec
PW1 2.95 usec
IRNUC 1H
CTEMP 16.9 c
SLVNT CDCL3
EXREF 0.00 ppm
BF 0.10 Hz
RGAIN 60

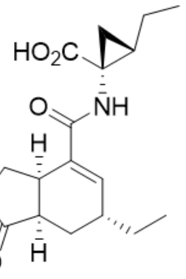

2l

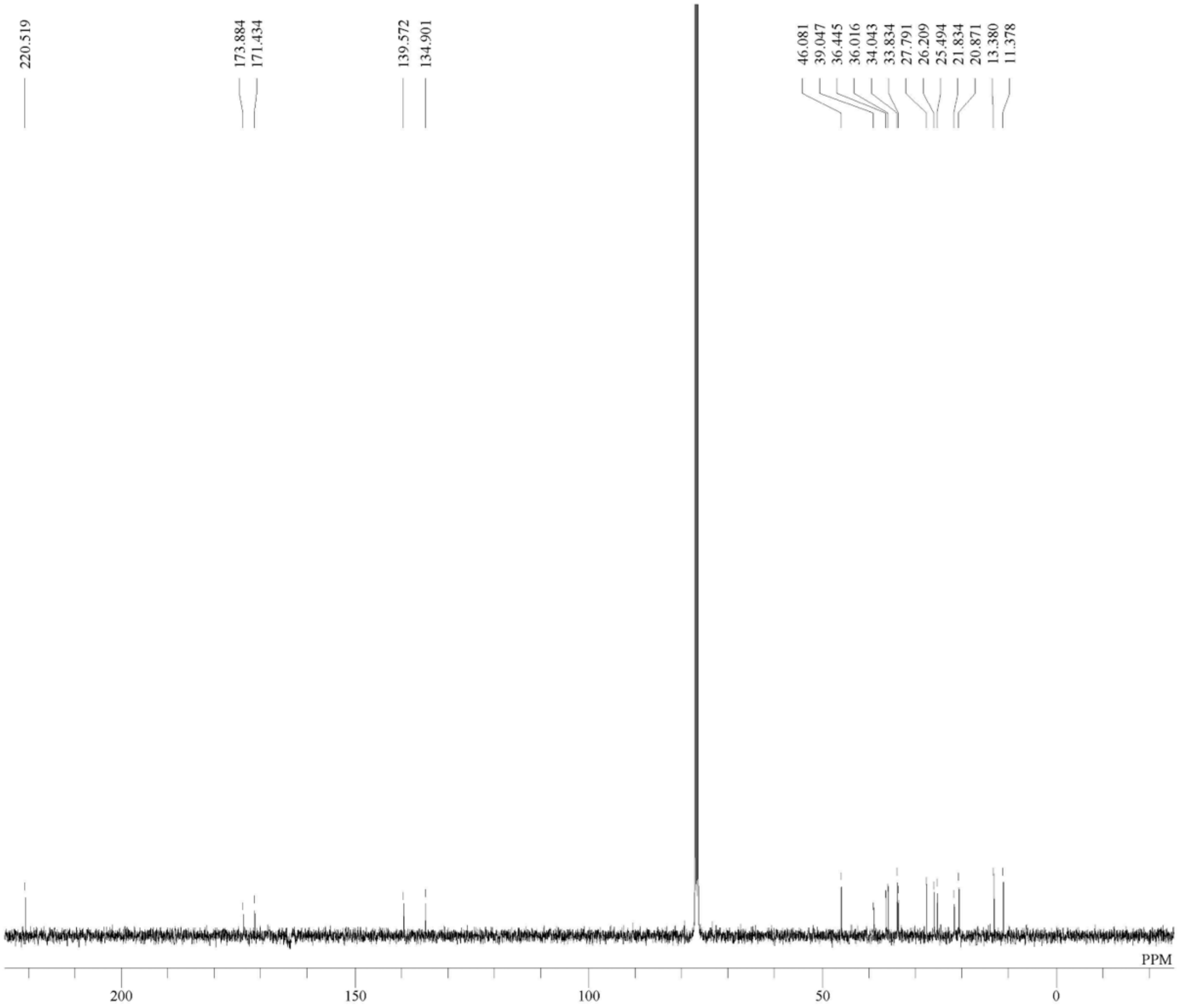

DFILE	3-152-4_Carbon-1-1.jdf
COMNT	single pulse decoupled gated N[illegible]
DATIM	25-12-2018 21:28:05
OBNUC	13C
EXMOD	carbon.jxp
OBFRQ	100.53 MHz
OBSET	5.35 KHz
OBFIN	5.86 Hz
POINT	32780
FREQU	31407.04 Hz
SCANS	1555
ACQTM	1.0433 sec
PD	2.0000 sec
PW1	3.37 usec
IRNUC	1H
CTEMP	20.5 c
SLVNT	CDCL3
EXREF	77.00 ppm
BF	0.10 Hz
RGAIN	50

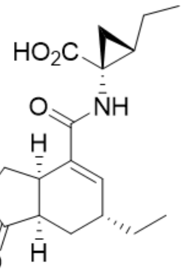

2l

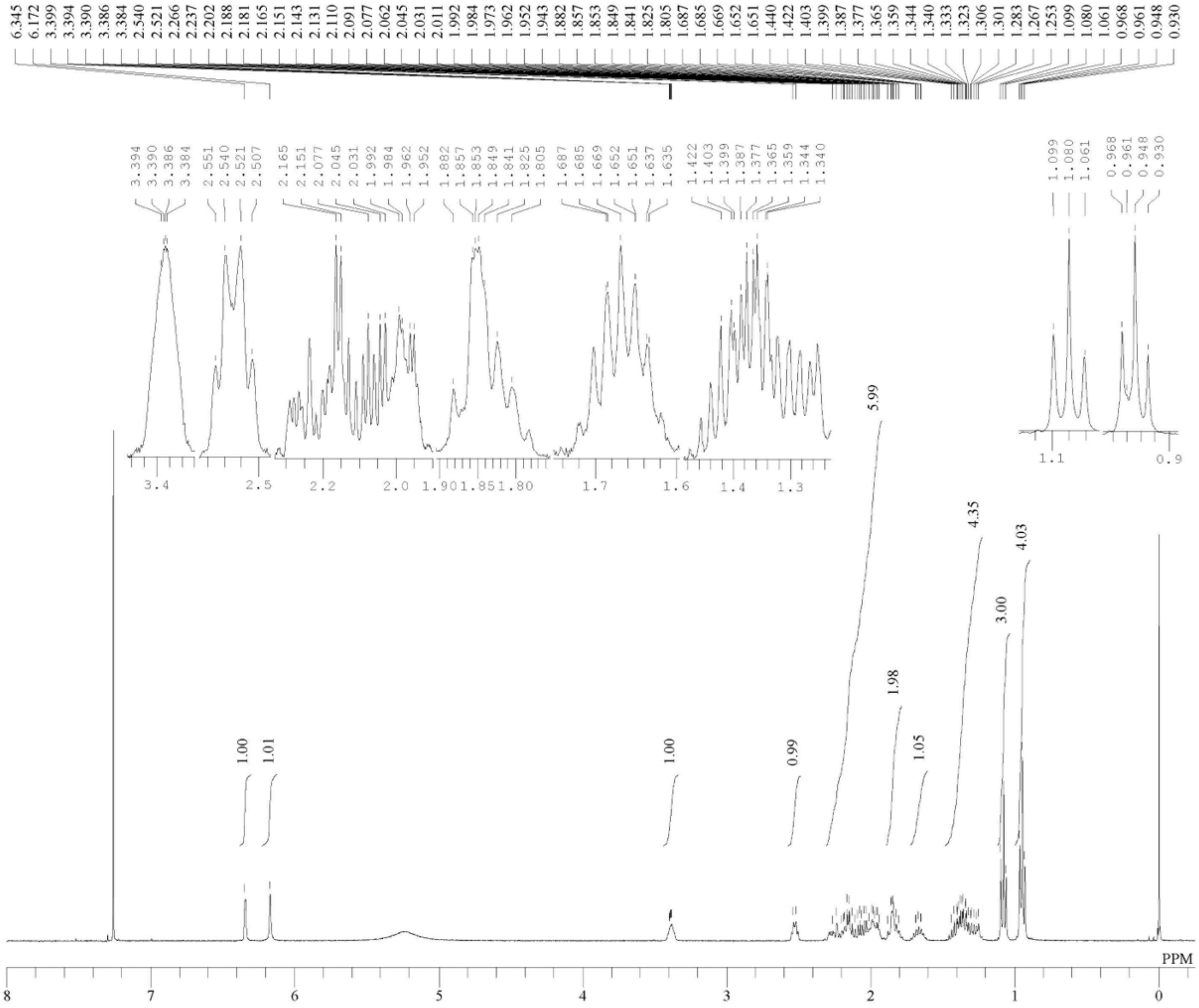

DFILE 3-147-3_Proton-1-1.als
COMNT single_pulse
DATIM 15-12-2018 15:04:39
OBNUC 1H
EXMOD proton.jxp
OBFRQ 399.78 MHz
OBSET 4.19 KHz
OBFIN 7.29 Hz
POINT 16384
FREQU 7503.00 Hz
SCANS 8
ACQTM 2.1837 sec
PD 5.0000 sec
PW1 2.95 usec
IRNUC 1H
CTEMP 19.8 c
SLVNT CDCL3
EXREF 0.00 ppm
BF 0.10 Hz
RGAIN 62

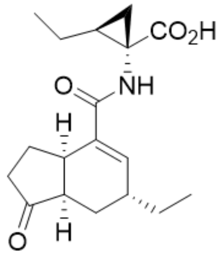

2m

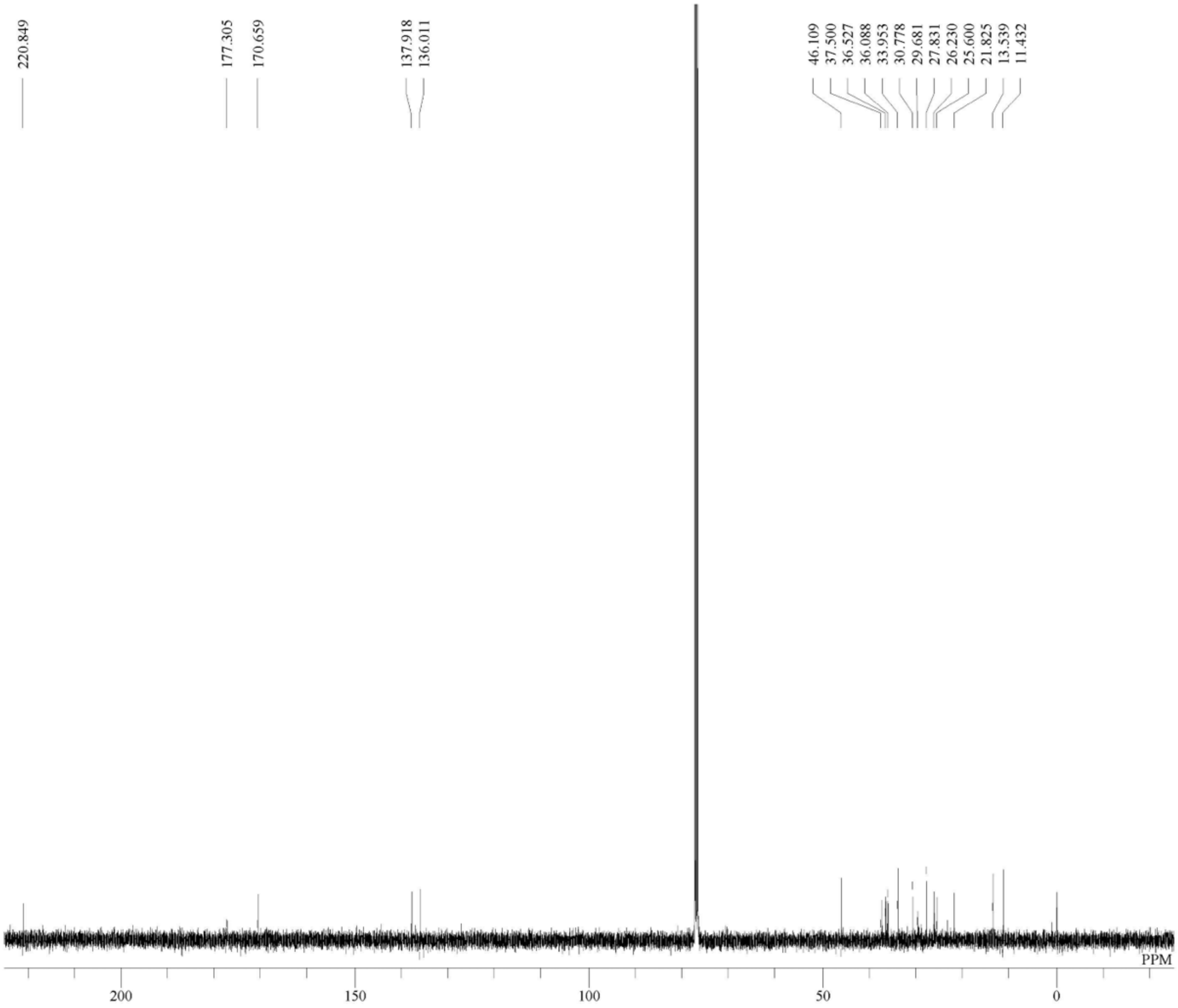

DFILE 3-146-3_Carbon-1-1.als
COMNT single pulse decoupled gated N[illegible]
DATIM 13-12-2018 23:47:07
OBNUC 13C
EXMOD carbon.jxp
OBFRQ 100.53 MHz
OBSET 5.35 KHz
OBFIN 5.86 Hz
POINT 32767
FREQU 31407.04 Hz
SCANS 12252
ACQTM 1.0433 sec
PD 2.0000 sec
PW1 3.37 usec
IRNUC 1H
CTEMP 19.7 c
SLVNT CDCL3
EXREF 0.00 ppm
BF 0.10 Hz
RGAIN 50

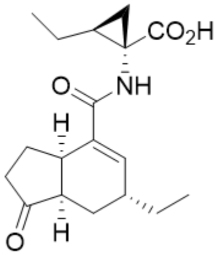

2m

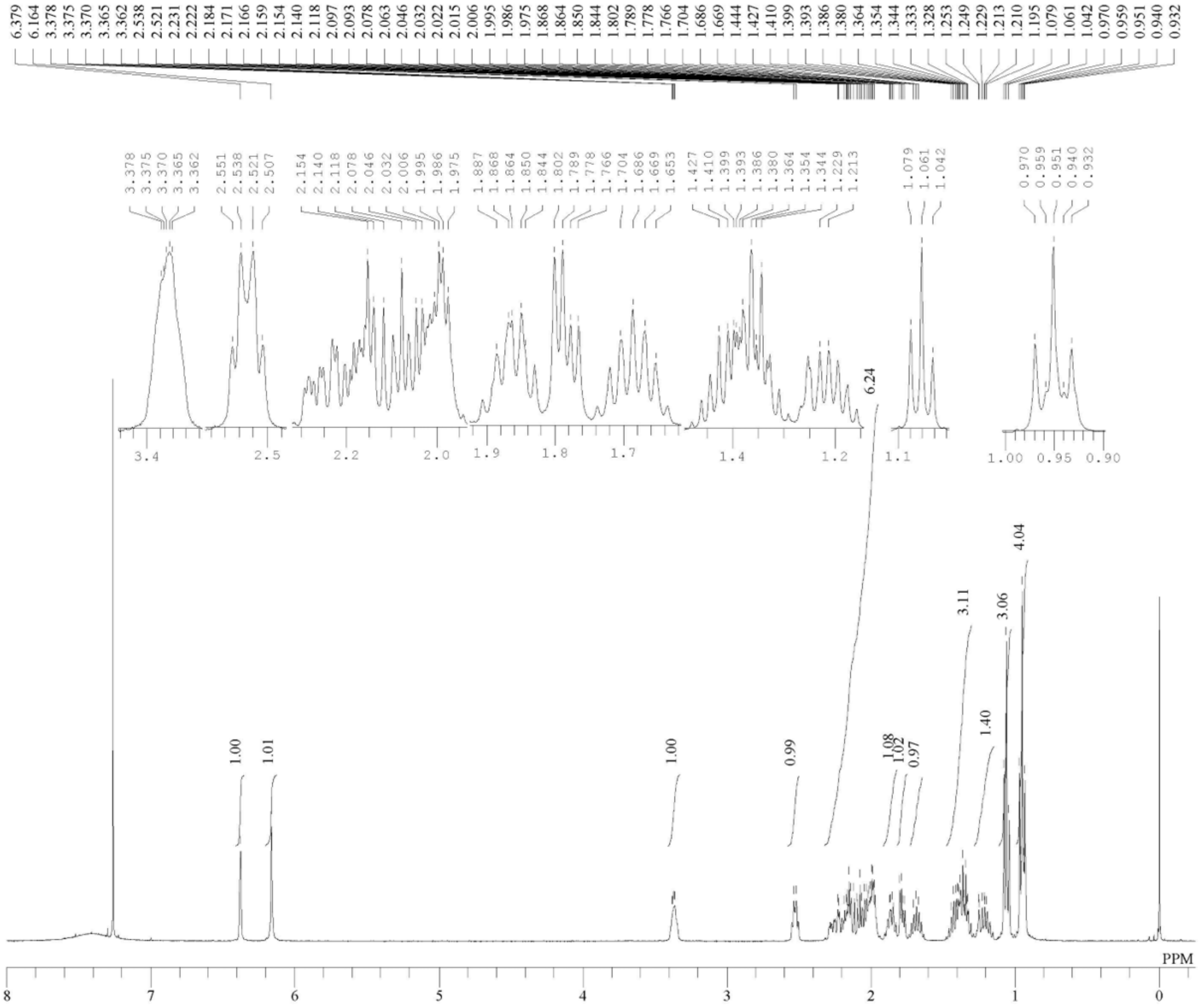

DFILE 3-162-5_Proton-1-1.jdf
COMNT single_pulse
DATIM 18-01-2019 15:19:01
OBNUC 1H
EXMOD proton.jxp
OBFRQ 399.78 MHz
OBSET 4.19 KHz
OBFIN 7.29 Hz
POINT 16384
FREQU 7503.00 Hz
SCANS 8
ACQTM 2.1837 sec
PD 5.0000 sec
PW1 2.95 usec
IRNUC 1H
CTEMP 18.4 c
SLVNT CDCL3
EXREF 0.00 ppm
BF 0.10 Hz
RGAIN 54

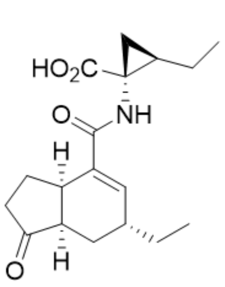

2n

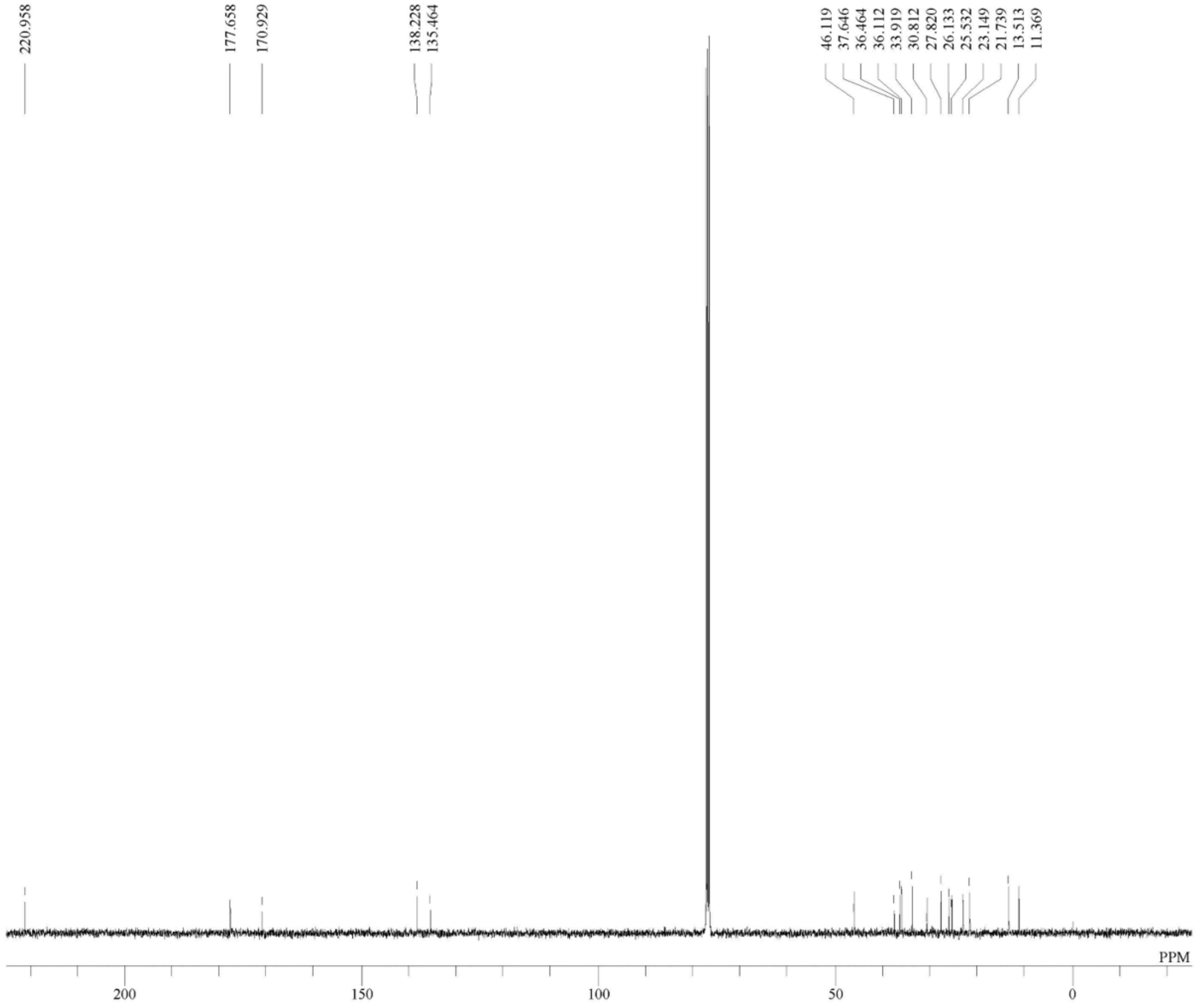

DFILE 3-162-5_Carbon-1-1.jdf
COMNT single pulse decoupled gated N
DATIM 18-01-2019 15:20:15
OBNUC 13C
EXMOD carbon.jxp
OBFRQ 100.53 MHz
OBSET 5.35 KHz
OBFIN 5.86 Hz
POINT 32780
FREQU 31407.04 Hz
SCANS 1220
ACQTM 1.0433 sec
PD 2.0000 sec
PW1 3.37 usec
IRNUC 1H
CTEMP 18.6 c
SLVNT CDCL3
EXREF 77.00 ppm
BF 0.10 Hz
RGAIN 50

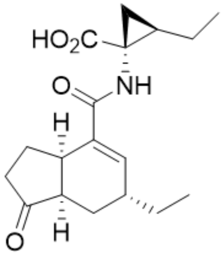

2n

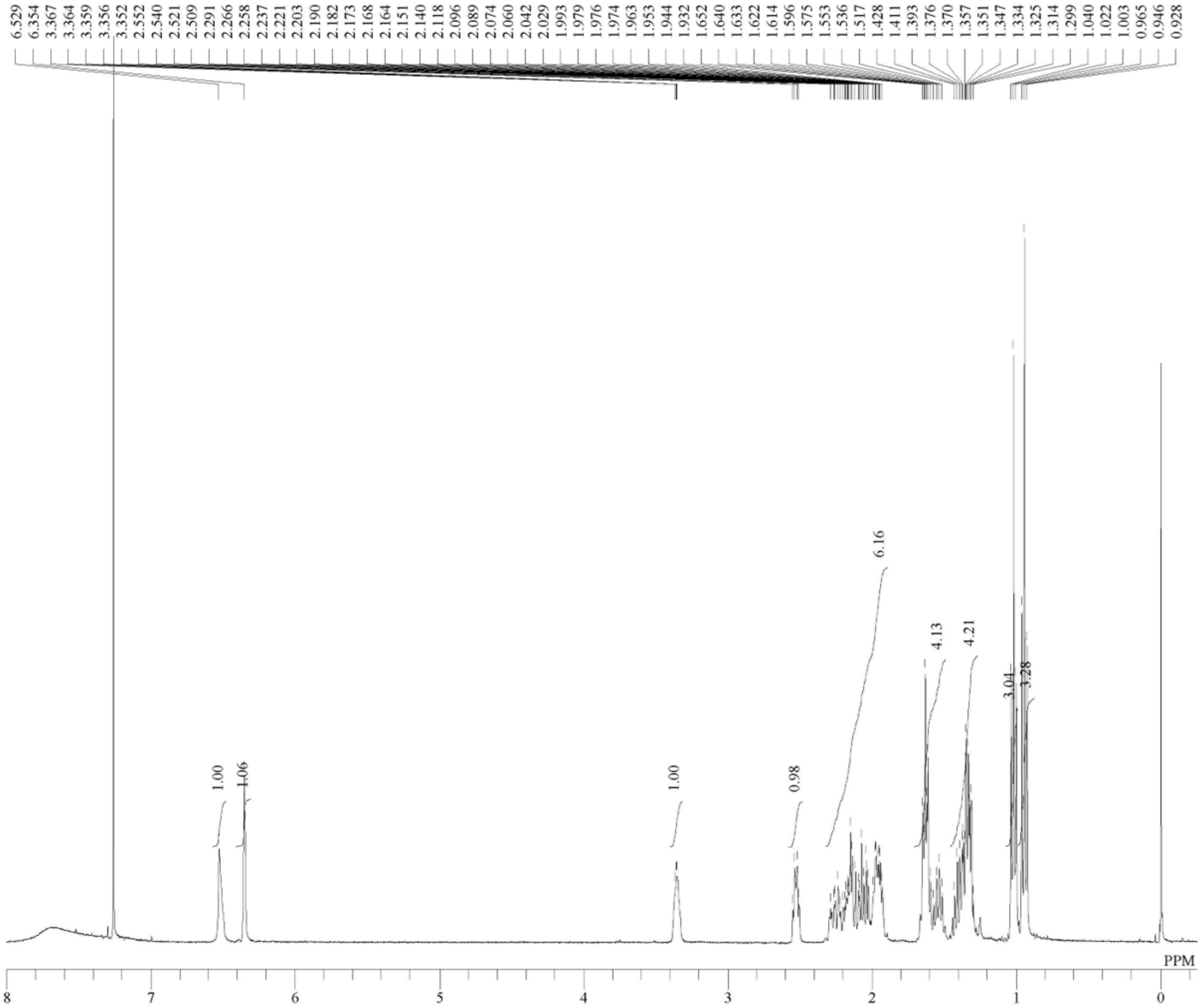

DFILE 3-141-5_Proton-1-1.als
COMNT single_pulse
DATIM 08-12-2018 21:31:59
OBNUC 1H
EXMOD proton.jxp
OBFRQ 399.78 MHz
OBSET 4.19 KHz
OBFIN 7.29 Hz
POINT 16384
FREQU 7503.00 Hz
SCANS 8
ACQTM 2.1837 sec
PD 5.0000 sec
PW1 2.95 usec
IRNUC 1H
CTEMP 20.9 c
SLVNT CDCL3
EXREF 0.00 ppm
BF 0.10 Hz
RGAIN 60

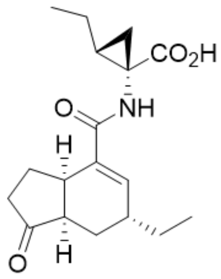

2o

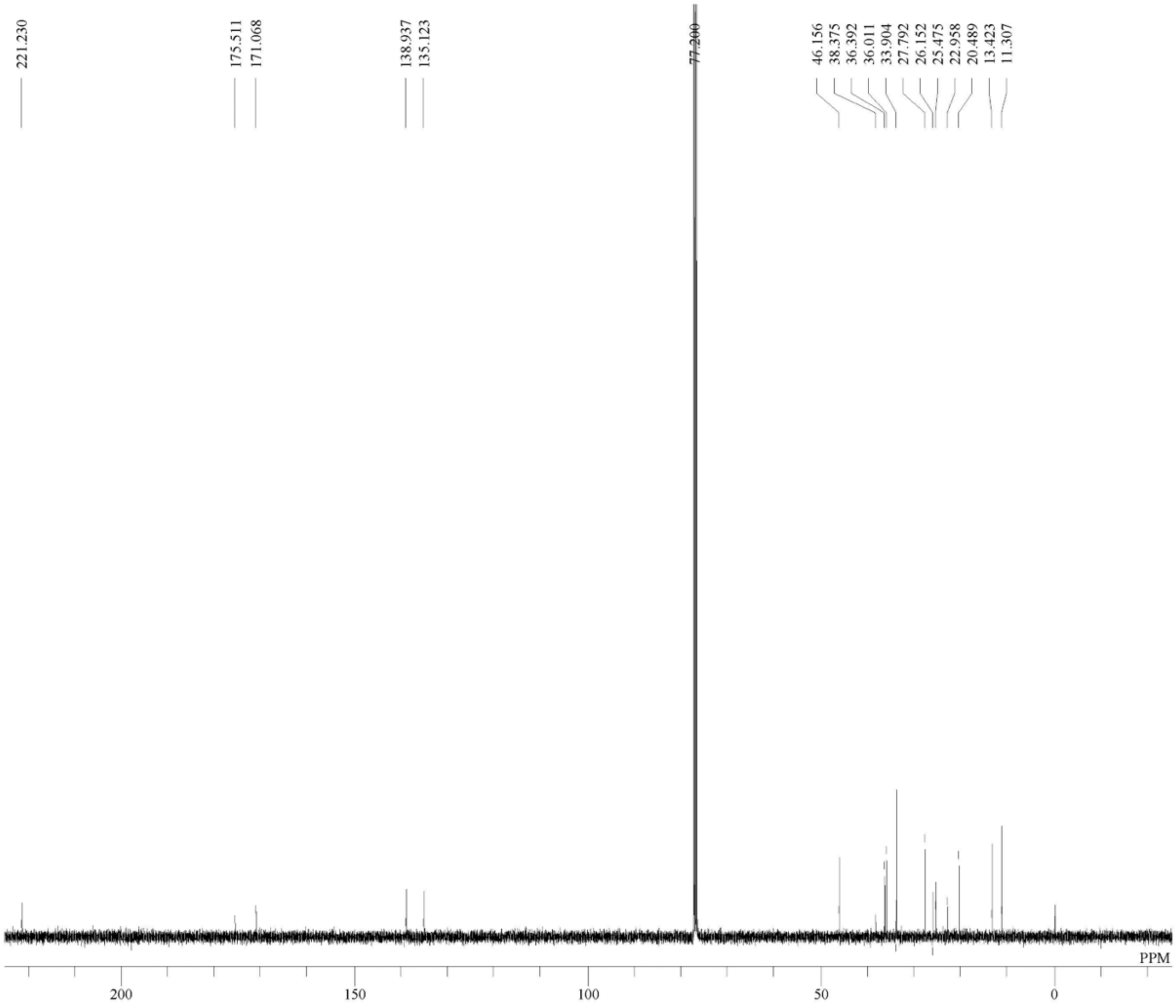

DFILE	3-141-5_Carbon-1-1.als
COMNT	single pulse decoupled gated N[illegible]
DATIM	08-12-2018 21:33:13
OBNUC	13C
EXMOD	carbon.jxp
OBFRQ	100.53 MHz
OBSET	5.35 KHz
OBFIN	5.86 Hz
POINT	32767
FREQU	31407.04 Hz
SCANS	8192
ACQTM	1.0433 sec
PD	2.0000 sec
PW1	3.37 usec
IRNUC	1H
CTEMP	20.8 c
SLVNT	CDCL3
EXREF	77.00 ppm
BF	0.10 Hz
RGAIN	50

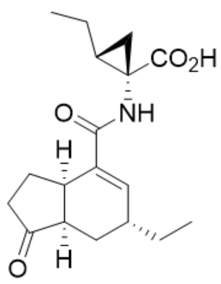

2o

Supplementary References

1. Kato, N. *et al.* A scalable synthesis of (+)-coronafacic acid. *Chirality* **32**, 423-430 (2020).
2. Watanabe, R. *et al.* Stereoselective Syntheses of all the Possible Stereoisomers of Coronafacic Acid. *ChemistryOpen* **9**, 1008-1017 (2020).
3. Okada, M. *et al.* Total syntheses of coronatines by exo-selective Diels–Alder reaction and their biological activities on stomatal opening. *Organic & Biomolecular Chemistry* **7**, 3065-3065 (2009).
4. Takaoka, Y. *et al.* A rationally designed JAZ subtype-selective agonist of jasmonate perception. *Nat Commun* **9**, 3654 (2018).
5. Parrinello, M. & Rahman, A. Polymorphic Transitions in Single-Crystals - a New Molecular-Dynamics Method. *Journal of Applied Physics* **52**, 7182-7190 (1981).
6. Hoover, W.G. Canonical dynamics: Equilibrium phase-space distributions. *Phys Rev A Gen Phys* **31**, 1695-1697 (1985).
7. Huang, J. & MacKerell, A.D., Jr. CHARMM36 all-atom additive protein force field: validation based on comparison to NMR data. *J Comput Chem* **34**, 2135-2145 (2013).
8. Vanommeslaeghe, K. *et al.* CHARMM general force field: A force field for drug-like molecules compatible with the CHARMM all-atom additive biological force fields. *J Comput Chem* **31**, 671-690 (2010).
9. Jorgensen, W.L., Chandrasekhar, J., Madura, J.D., Impey, R.W. & Klein, M.L. Comparison of simple potential functions for simulating liquid water. *The Journal of Chemical Physics* **79**, 926-935 (1983).
10. Darden, T., York, D. & Pedersen, L. Particle mesh Ewald: An N · log(N) method for Ewald sums in large systems. *J Chem Phys* **98**, 10089-10092 (1993).
11. Littleson, M.M. *et al.* Scalable total synthesis and comprehensive structure-activity relationship studies of the phytotoxin coronatine. *Nat Commun* **9**, 1105 (2018).
12. Miyamoto, K. *et al.* Facile preparation of optically active jasmonates and their biological activities in rice. *Bioscience, biotechnology, and biochemistry*, 1-6 (2019).
13. Saitou, N. & Nei, M. The neighbor-joining method: a new method for

reconstructing phylogenetic trees. *Mol Biol Evol* **4**, 406-425 (1987).

14. Cui, M., Du, J. & Yao, X. The Binding Mechanism Between Inositol Phosphate (InsP) and the Jasmonate Receptor Complex: A Computational Study. *Front Plant Sci* **9**, 963 (2018).
15. Cui, M., Zhang, K., Wu, R. & Du, J. Exploring the interaction mechanism between antagonist and the jasmonate receptor complex by molecular dynamics simulation. *J Comput Aided Mol Des* (2022).
