# Supplementary material for: Subtype-selective agonists of plant hormone co-receptor COI1-JAZs identified from the stereoisomers of coronatine
Source: Commun Biol. 2023 Mar 25;6:320. doi: 10.1038/s42003-023-04709-1 (PMC10039919; doi:10.1038/s42003-023-04709-1)
Supplement: Supplementary file 3 — Description of Additional Supplementary Files [file 42003_2023_4709_MOESM3_ESM.pdf]

Description of Additional Supplementary Files

File name: Supplementary Data 1
Description: The whole data of RNAseq analysis in the paper.

File name: Supplementary Data 2
Description: The primer sequences used in this paper.

File name: Supplementary Data 3
Description: The source data behind the graphs in the main text of this paper.
