# Supplementary material for: Subtype-selective agonists of plant hormone co-receptor COI1-JAZs identified from the stereoisomers of coronatine
Source: Commun Biol. 2023 Mar 25;6:320. doi: 10.1038/s42003-023-04709-1 (PMC10039919; doi:10.1038/s42003-023-04709-1)
Supplement: Supplementary file 7 — Reporting Summary [file 42003_2023_4709_MOESM7_ESM.pdf]

nature portfolio

Corresponding author(s): Minoru Ueda

Last updated by author(s): Mar 8, 2023

# Reporting Summary

Nature Portfolio wishes to improve the reproducibility of the work that we publish. This form provides structure for consistency and transparency in reporting. For further information on Nature Portfolio policies, see our Editorial Policies and the Editorial Policy Checklist.

## Statistics

For all statistical analyses, confirm that the following items are present in the figure legend, table legend, main text, or Methods section.

| n/a | Confirmed | |
|---|---|---|
| ☐ | ☒ | The exact sample size (*n*) for each experimental group/condition, given as a discrete number and unit of measurement |
| ☐ | ☒ | A statement on whether measurements were taken from distinct samples or whether the same sample was measured repeatedly |
| ☐ | ☒ | The statistical test(s) used AND whether they are one- or two-sided<br>*Only common tests should be described solely by name; describe more complex techniques in the Methods section.* |
| ☐ | ☒ | A description of all covariates tested |
| ☐ | ☒ | A description of any assumptions or corrections, such as tests of normality and adjustment for multiple comparisons |
| ☐ | ☒ | A full description of the statistical parameters including central tendency (e.g. means) or other basic estimates (e.g. regression coefficient) AND variation (e.g. standard deviation) or associated estimates of uncertainty (e.g. confidence intervals) |
| ☐ | ☒ | For null hypothesis testing, the test statistic (e.g. *F*, *t*, *r*) with confidence intervals, effect sizes, degrees of freedom and *P* value noted<br>*Give P values as exact values whenever suitable.* |
| ☒ | ☐ | For Bayesian analysis, information on the choice of priors and Markov chain Monte Carlo settings |
| ☒ | ☐ | For hierarchical and complex designs, identification of the appropriate level for tests and full reporting of outcomes |
| ☒ | ☐ | Estimates of effect sizes (e.g. Cohen's *d*, Pearson's *r*), indicating how they were calculated |

*Our web collection on statistics for biologists contains articles on many of the points above.*

## Software and code

Policy information about availability of computer code

| | |
|---|---|
| Data collection | JEOL Delta v5.0.4 was used for NMR analysis. micrOTOF control Version 3.0 was used for mass spectrometry. EnVision Manager Version 1.13.3009.1409 was used for fluorescence anisotropy. StepOne Software Version 2.2.2 was used for quantitative RTPCR. |
| Data analysis | Microsoft Excel 2019 and KaleidaGraph were used to analyze the data in this study. MOE 2020.0901 was used to create images of ligand binding structure. |

For manuscripts utilizing custom algorithms or software that are central to the research but not yet described in published literature, software must be made available to editors and reviewers. We strongly encourage code deposition in a community repository (e.g. GitHub). See the Nature Portfolio guidelines for submitting code & software for further information.

## Data

Policy information about availability of data

All manuscripts must include a data availability statement. This statement should provide the following information, where applicable:

- Accession codes, unique identifiers, or web links for publicly available datasets
- A description of any restrictions on data availability
- For clinical datasets or third party data, please ensure that the statement adheres to our policy

The data supporting the findings in this study are available in the paper and its Supplimentary Information. All data generated or analyzed are available from the correspondind author on reasonable request. The raw RNAseq data can be downloaded from the Gene Expression Omnibus repository (GSE184730).

## Human research participants

Policy information about studies involving human research participants and Sex and Gender in Research.

| | |
|---|---|
| Reporting on sex and gender | Not applicable. |
| Population characteristics | Not applicable |
| Recruitment | Not applicable |
| Ethics oversight | Not apllicable |

Note that full information on the approval of the study protocol must also be provided in the manuscript.

# Field-specific reporting

Please select the one below that is the best fit for your research. If you are not sure, read the appropriate sections before making your selection.

☒ Life sciences ☐ Behavioural & social sciences ☐ Ecological, evolutionary & environmental sciences

For a reference copy of the document with all sections, see nature.com/documents/nr-reporting-summary-flat.pdf

# Life sciences study design

All studies must disclose on these points even when the disclosure is negative.

| | |
|---|---|
| Sample size | Sample size was chosen by according to previous reports using similar experimental condition. All biochemical experiments were performed in at least three independent experiments. All biological experiments were performed in at least three independent experiments. Sample size of each experiment was indicated in each figure legend or method. |
| Data exclusions | No data was excluded from experiments. |
| Replication | All experiments were reproduced at least 3 times. |
| Randomization | No randomization was used. |
| Blinding | Investigators were not blinded during data collection and analysis. qRT-PCR was performed to validate the RNAseq in some JA marker genes. |

# Reporting for specific materials, systems and methods

We require information from authors about some types of materials, experimental systems and methods used in many studies. Here, indicate whether each material, system or method listed is relevant to your study. If you are not sure if a list item applies to your research, read the appropriate section before selecting a response.

### Materials & experimental systems

| n/a | Involved in the study | |
|---|---|---|
| ☐ | ☒ | Antibodies |
| ☒ | ☐ | Eukaryotic cell lines |
| ☒ | ☐ | Palaeontology and archaeology |
| ☒ | ☐ | Animals and other organisms |
| ☒ | ☐ | Clinical data |
| ☒ | ☐ | Dual use research of concern |

### Methods

| n/a | Involved in the study | |
|---|---|---|
| ☒ | ☐ | ChIP-seq |
| ☒ | ☐ | Flow cytometry |
| ☒ | ☐ | MRI-based neuroimaging |

## Antibodies

| | |
|---|---|
| Antibodies used | The following antibodies were used for western blot and pull-down assay: fluorescein (GENETEX, GTX26644, goat, 0.2 uL for each sample), GST (cytiva, RPN1236, goat, 1:5000), GFP (Miltenyi Biotec, 130-091-833, mouse, 1:5000), b-actin (proteintech, 60008-1-Ig, mouse, 1:1000), mouse IgM (TOKYO CHEMICAL INDUSTRY, G0417, goat, 1:20000). |
| Validation | Anti-fluorescein is a goat polyclonal IgG and suitable for WB and IP;<br>Anti-GST is a goat polyclonal IgG and conjugated with HRP. Anti-GST is recommended for detection of GST-conjugated proteins on |

the cytiva website;
Anti-GFP is a mouse monoclonal IgG1 and conjugated with HRP. Anti-GFP is recommended for detection of GFP-conjugated proteins on the Miltenyi Biotec website;
Anti-b-actin is a mouse monoclonal IgM and recommended for detection of b-actin of mammalian cells and plant tissue by Western Blotting on the TOKYO CHEMICAL INDUSTRY website;
Anti-mouse IgM is a goat polyclonal IgG and conjugated with HRP. Anti-mouse IgM is recommended for detection of mouse IgM by Western Blotting on the TOKYO CHEMICAL INDUSTRY website.
